# The first *de novo* transcriptome of pepino (*Solanum muricatum*): assembly, comprehensive analysis and comparison with the closely related species *S. caripense*, potato and tomato

**DOI:** 10.1186/s12864-016-2656-8

**Published:** 2016-05-04

**Authors:** Francisco J. Herraiz, José Blanca, Pello Ziarsolo, Pietro Gramazio, Mariola Plazas, Gregory J. Anderson, Jaime Prohens, Santiago Vilanova

**Affiliations:** Instituto de Conservación y Mejora de la Agrodiversidad Valenciana, Universitat Politècnica de València, Camino de Vera 14, 46022 Valencia Spain; Department of Ecology and Evolutionary Biology, University of Connecticut, Storrs, CT 06268-3043 USA

**Keywords:** *Solanum muricatum*, Transcriptome, *S. caripense*, Pepino, Potato, Tomato, Solanaceae, Functional annotation, Phylogeny, Candidate genes, Molecular markers

## Abstract

**Background:**

*Solanum* sect. *Basarthrum* is phylogenetically very close to potatoes (*Solanum* sect. *Petota*) and tomatoes (*Solanum* sect. *Lycopersicon*), two groups with great economic importance, and for which *Solanum* sect. *Basarthrum* represents a tertiary gene pool for breeding. This section includes the important regional cultigen, the pepino (*Solanum muricatum*), and several wild species. Among the wild species, *S. caripense* is prominent due to its major involvement in the origin of pepino and its wide geographical distribution. Despite the value of the pepino as an emerging crop, and the potential for gene transfer from both the pepino and *S. caripense* to potatoes and tomatoes, there has been virtually no genomic study of these species.

**Results:**

Using Illumina HiSeq 2000, RNA-Seq was performed with a pool of three tissues (young leaf, flowers in pre-anthesis and mature fruits) from *S. muricatum* and *S. caripense*, generating almost 111,000,000 reads among the two species. A high quality *de novo* transcriptome was assembled from *S. muricatum* clean reads resulting in 75,832 unigenes with an average length of 704 bp. These unigenes were functionally annotated based on similarity of public databases. We used Blast2GO, to conduct an exhaustive study of the gene ontology, including GO terms, EC numbers and KEGG pathways. Pepino unigenes were compared to both potato and tomato genomes in order to determine their estimated relative position, and to infer gene prediction models. Candidate genes related to traits of interest in other Solanaceae were evaluated by presence or absence and compared with *S. caripense* transcripts. In addition, by studying five genes, the phylogeny of pepino and five other members of the family, Solanaceae, were studied. The comparison of *S. caripense* reads against *S. muricatum* assembled transcripts resulted in thousands of intra- and interspecific nucleotide-level variants. In addition, more than 1000 SSRs were identified in the pepino transcriptome.

**Conclusions:**

This study represents the first genomic resource for the pepino. We suggest that the data will be useful not only for improvement of the pepino, but also for potato and tomato breeding and gene transfer. The high quality of the transcriptome presented here also facilitates comparative studies in the genus *Solanum*. The accurate transcript annotation will enable us to figure out the gene function of particular traits of interest. The high number of markers (SSR and nucleotide-level variants) obtained will be useful for breeding programs, as well as studies of synteny, diversity evolution, and phylogeny.

**Electronic supplementary material:**

The online version of this article (doi:10.1186/s12864-016-2656-8) contains supplementary material, which is available to authorized users.

## Background

The pepino (*Solanum muricatum* Aiton) is a neglected herbaceous domesticate native to the Andean region, where wild relatives (*Solanum* section *Basarthrum*) are naturally distributed [1, 2]. The pepino is a vegetatively propagated cultigen grown for its fruit. The fruits are juicy berries, of variable shape and color depending on the cultivar, which typically weighs between 100 and 400 g. The fruit has an attractive appearance, with most common cultivars producing fruits with a golden yellow skin covered with purple stripes. Nutritionally, the pepino has very high levels of potassium and vitamin C, and a low calorie content [3]. Apart from being cultivated in its region of origin, the pepino has been introduced in other countries like New Zealand, China and Turkey as a potential new horticultural crop [4, 5].

One of the most interesting features of the pepino is its phylogenetically close relationship with the potato and tomato [6, 7]. In fact, the pepino and its wild relatives in *Solanum* sect. *Basarthrum* are part of the tertiary gene pool of both potato and tomato [8, 9]. Cultivated potato, tomato and pepino share the same basic number of chromosomes (x = 12) [10, 11], although tomato and pepino are diploid and most cultivated potato cultivars are polyploid [12]. Importantly, the close phylogenetic relationship among these species allows the use of genomic resources from tomato and potato for pepino breeding, as has been demonstrated with the high transferability of tomato SSRs to the pepino [13]. Reciprocally, the close relationship may also facilitate the use of the pepino as a genetic source for tomato and potato breeding including resistance to several diseases in both crops, and the transfer of parthenocarpy and improved flavor in the tomato [3, 8, 14]. The first step in introgression of pepino traits into tomato, have been obtained via the construction of tomato-pepino somatic hybrids [9].

Wild relatives of domesticates are a source of variation for improving cultivated species [15] and for studying the domestication process [16]. In this context, *S. caripense* Dunal*,* locally known as *“*mamoncillo” or “tzimbalo”, is important: it is easily hybridized with the pepino, and hybrids are highly fertile [3]. AFLP and genic DNA sequence studies indicated that *S. caripense* is one of the species that has been involved in the origin and evolution of the cultivated pepino [17]. In the Andean region the widely distributed *S. caripense* and other cross-compatible wild relatives frequently grow in close proximity to the fields and gardens of the cultivated pepino; as a consequence, there is evidence of introgression and gene flow among them [17]. *Solanum caripense* is of particular interest because of traits for pepino breeding such as high levels of soluble solids content [18], or a high content of bioactive phenolic acids [19]. In addition, some accessions of *S. caripense* have displayed resistance to Tomato Mosaic Virus (ToMV) [20] and to *Phytophthora infestans* [21], the most important disease of potato [22], and could offer alternative sources of variation for breeding for resistance to these diseases.

Despite being an important crop in the Andean region during pre-Columbian times [23, 24] and despite its potential as a new crop for many areas with mild climates, there have been few molecular studies of, and few molecular tools developed for *S. muricatum* - the pepino. Neither the pepino or its significant wild relatives have been thoroughly studied at a genome-wide level in the context of molecular studies and tools. As of July 2015, only 126 nucleotide sequences had been deposited in the NCBI’s GenBank database, all of them resulting from a single study [17]. In addition, there are few studies of molecular markers and their application in pepinos. Some of the previous studies used cp-DNA restriction fragments length polymorphism (RFLP) [1], AFLP and gene sequence haplotypes [17], RAPDs [25] and EST-SSRs derived from tomato [13], to study diversity in the pepino and its wild relatives. Apart from these studies, an intra-specific low-density genetic map with SNPs taken from the sequencing of a set of COSII was produced in the pepino wild relative *S. caripense* with the aim of mapping the resistance to *Phytophthora infestans* [21].

High throughput sequencing of transcriptomes (RNA-Seq) has opened the way to study the genetic and functional information in neglected crops and species. RNA-Seq is genome-independent and is especially useful for analyzing the transcriptome of species without complete genome information or a reference genome [26, 27], as is the case of the pepino and wild relatives. In this context, RNA-Seq can be helpful for: (1) listing the transcripts and other RNAs from one or several tissues; (2) investigating the transcriptional structure of genes, splicing patterns, and gene isoforms; (3) studying post-transcriptional modification and mutations; and (4) quantifying gene expression [28]. The transcriptomics studied have provided a basis for: scanning the evolution of polyploidy in plants [29, 30], study of phylogenies in some families including the Solanaceae [31], comparing patterns associated with domestication [32], and finally for developing markers *en masse* [33–35].

In the present work, we used the Illumina pair-end sequencing technology to perform RNA-Seq of one modern cultivar of the pepino and of one accession of the pepino wild relative *S. caripense*. We obtained almost 111 million reads including sequencing of both species. Our transcriptome analysis included *de novo* assembly, structural and functional annotation and comparison with tomato and potato genomes [36, 37], providing us the opportunity to establish a dated phylogeny of the pepino compared with related species. Candidate genes, mainly from tomato agronomic traits of interest have also been found. These genes can provide us an effective comparative approximation of patterns of selection in domestication, and will allow us to identify genes useful for the genetic improvement of the pepino. Another important goal is the discovery of the high throughput markers (SSR and SNPs). These gene-derived markers are important functionally in that they can provide potential changes in the proteins expressed, and they offer an essential tool to be utilized in the construction of genetic maps, and they can be used in marker-assisted selection. The rest of the dataset will serve as a public information platform for gene expression and genomics in the pepino and their related species, particularly useful for future studies in pepino, potato, and tomato genomics and breeding. This is a seminal work that will pave the way to broader genomic studies in pepino, a neglected species with great interest for future development, and as a reservoir of important genes for tomato and potato improvement as well.

## Results

### Transcriptome sequencing (mRNA-Seq) output and assembly

We performed two Illumina Hiseq-2000 runs, one for the *S. muricatum* cultivar Sweet Long (SL) and one for *S. caripense* (EC-40) [1, 38]. The RNA sequencing of three tissues (young leaves, young flowers and mature fruits) from *S. muricatum* generated 58,327,154 raw paired-end reads, covering about 11.78 Gb of sequencing raw data (reads with a length of 101 bp). In the case of *S. caripense*, sequencing generated 52,646,045 reads and 10.63 Gb of raw data. Graphical representation of sequence quality is shown in Additional file 1: Figure S1, where the quality scores across all the bases is indicated. All these raw paired-end data have been deposited in the NCBI Sequence Read Archive. After initial trimming and quality filtering to remove adapters and low-quality data, 33,963,075 clean paired end reads were obtained, encompassing 6.86 Gb of sequencing data in *S. muricatum* and 36,228,181 clean reads and 7.32 Gb of sequencing data in *S. caripense* (Table 1).

**Table 1:** Summary of raw and clean reads after processing for *S. muricatum* and *S. caripense*

	*Solanum muricatum*	*Solanum caripense*
Total raw reads	58,327,154 × 2	52,646,045 × 2
Total raw reads data size (Gb)	11.78 Gb	10.63 Gb
% G/C	42.0	42.3
Total clean reads	33,963,075 × 2	36,228,181 × 2
Total clean reads data size (Gb)	6.86 Gb	7.32 Gb
% G/C	41.7	42.5

**Fig. 1:**
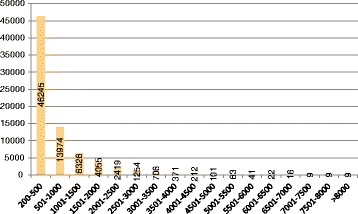
Length distribution of the pepino (*S. muricatum*) transcriptome most expressed transcripts. Horizontal axis represents the size range of each unigene. Vertical axis represents the number of unigenes for every interval

The high quality reads for *S. muricatum* were used to assemble the transcriptome using the Trinity software. The results of this assembly are shown in Table 2, and the length distribution of the transcripts after assembly is shown in Fig. 1. The total of 91,949 (Additional file 2) contigs were assembled with an average length of 895 bp. It should be noted that more than 63 % (58,465) of the transcripts are between 200 and 766 bp in length and only 1 % of them had a sequence length greater than 3500 bp. We have selected a subset of the transcripts, the selection based on the most expressed transcript of each Trinity transcript cluster. We obtained 75,832 of the most expressed transcripts (unigenes) (Additional file 3), with an average length of 704 bp. Additionally, the percentage of G/C of the clean reads, usually used as an indicator of closeness between species, was calculated. The value for *S. muricatum* was 41.7 % and for *S. caripense* it was 42.5 % (Table 1). These values are consistent with those reported for other *Solanum* species [31].

**Table 2:** Summary of the *Solanum muricatum* transcriptome assembly. After assembly in the first group (Transcripts), and after filtering by level of expression (Most expressed transcripts)

Transcripts	Number	91,949
	Total length	82,353,960
	Average length	895.65
	Maximum length	11,491
Most expressed transcripts	Number	75,832
	Total length	53,411,734
	Average length	704.34
	Maximum length	11,491

### Functional annotation

To identify *S. muricatum* transcripts potentially encoding proteins with a known function, a BLASTX analysis was performed sequentially using three protein databases [39]. The order of application of the databases used was: Swiss-Prot [40], ITAG2.4 from tomato [41] and UniRef90 [42], (e-value cut-off of 1e^−20^). More than 65.9 % of the transcripts (49,662) had at least one significant hit. Most of the transcripts with annotation had significant hits in Swiss-Prot (53.7 %), representing 35.2 % of the total of unigenes, an expected result for a manually curated database. The hits obtained in the rest of databases were: ITAG2.4 with 30.1 % of the sequences annotated and UniRef90 with 4.6 % of the sequences annotated. The results are presented in Table 3 and they are similar to those found in other studies [43–45]. To facilitate further study, the blast results are listed in an Additional file 4.

**Table 3:** Functional annotation summary of the pepino sequences over protein databases. First the most expressed transcripts were annotated in Swiss-Prot database. Then, unpaired transcripts in this annotation were evaluated in the next database, ITAG2.4. And finally, the unpaired at this level, were evaluated in the Uniref90 database

	Number of transcripts	% of total
Annotated in Swiss-Prot	26,688	53.7 %
Annotated in ITAG2.4	14,942	30.1 %
Annotated in UniRef90	2263	4.6 %
Total annotated in protein databases	43.893	
TOTAL Annotations	49,662	

Using Blast2GO against the NR database, we recovered gene ontology (GO) terms and enzyme commission numbers (EC) for the most expressed transcripts or unigenes in *S. muricatum*. A total of 197,221 GO terms were assigned to 37,031 transcripts. The distribution of unigenes relative to the number of GOs to which they were assigned is shown in Additional file 5: Figure S2. Slightly more than half of the unigenes (50.7 %) have between 1 and 5 GO terms, and 12 % have more than 10 GO terms. The maximum number of GO terms annotated in a unigene was 45. Among all the GO terms extracted, 89,060 (45.2 %) belong to the molecular function class (MF), 59,856 (30.3 %) to biological process class (BP) and 48,305 (24.5 %) to cellular components class (CC).

**Fig. 2:**
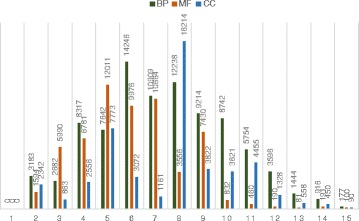
GO level distribution in each category for the annotated pepino unigenes. X axis represent the GO level and Y axis the number of annotated unigenes. BP = Biological Process, MF = Molecular Function, CC = Cellular Component

**Fig. 3:**
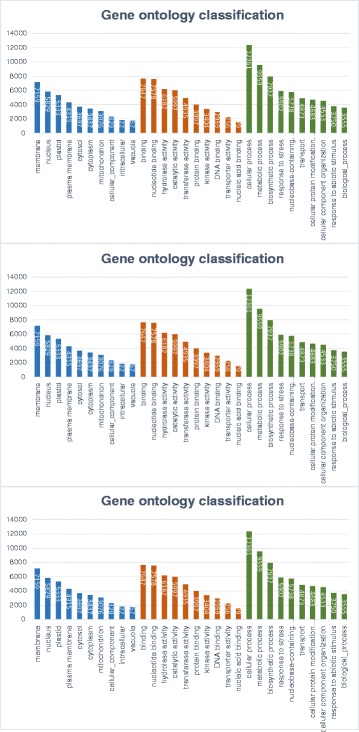
Gene ontology classification by plant slim term for level 2. The graphic indicates the number of transcripts for every process and functional category, cellular components (*left*), biological process (*middle*) and molecular function and (*right*)

The distribution of annotated transcripts under different GO levels shows a concentration, between levels 4–10 in the biological process, between levels 3–9 in molecular function and between levels 5–8 in cellular components (Fig. 2). The GO levels that ranged between 5 and 15, were 84.1 % for biological processes, 76.0 % for molecular function and 88.1 % for cellular components, indicating that the precision of the annotation was good (Fig. 2), and that a broad diversity of genes was sampled in our transcriptome. Most of the genes in the GO analysis were classified into biological process (BP) of cellular processes, metabolic processes, biosynthetic processes and response to stress (Fig. 3). Notably, many sequences were classified in the category of development for different plant structures, like embryo and flower development, pollen-pistil interaction and fruit ripening. All of these functions are related to the three tissues sampled (the full list of these annotation is shown in Additional file 6).

**Fig. 4:**
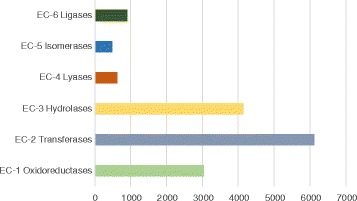
Number of unigenes for each enzyme commission (EC) category

The enzyme commission (EC) number is a codification for enzymes, based on the chemical reactions they catalyse [46]. We found a total of 15,337 annotations classified under this scheme involving 12,296 different unigenes (some unigenes have two or more EC annotations). The most prominent among the enzymes were: Transferases (EC-2) with 6127 sequences, hydrolases (EC-3) with 4126 sequences and oxidoreductases (EC-1) with 3046 sequences annotated (Fig. 4). Other enzyme classes like ligases, lyases and isomerases were represented to a lesser degree. The number of annotated sequences so recognized was greater than in other studies in the Solanaceae [47, 48]. The complete list of these EC numbers is included in the Additional file 6.

In order to understand the function of the unigenes in pepino, a BLASTX search against the KEGG protein database with a cut-off e value of 1e^−5^ was performed. Out of the 75,832 transcripts, 16,027 were annotated in the KEGG pathway database, and assigned to 144 unique pathways. These pathways include amino acid metabolism, sugar metabolism, fatty acid metabolism, as well as biosynthesis of secondary metabolites like flavonoids and terpenoids. Our results show that the largest three pathway groups were purine metabolism, starch and sucrose metabolism, and phenylalanine metabolism (see Additional file 7). Given that the pepino is largely a dessert fruit in which sugars and bioactive compounds are important for quality [3], we paid special attention to the pathways pertaining to starch and sucrose metabolism, and to biosynthesis of carotenoids, anthocyanins, and several vitamins. A considerable number of genes were related to relevant metabolic pathways, including starch and sucrose metabolism (map00500, 727 genes), carotenoid biosynthesis (map00906, 33 genes), anthocyanin biosynthesis (map00942, 31 genes), ascorbate and alderate metabolism (map00053, 123 genes), vitamin B6 metabolism (map00750, 28 genes), retinol (vitamin A) metabolism (map00830, 89 genes), thiamine (vitamin B1) metabolism (map00730, 325 genes), riboflavin (vitamin B2) metabolism (map00740, 117 genes), and biotin (vitamin H) metabolism (map00780, 98 genes). Finally, we compared the number of genes assigned for every KEGG pathway in our analysis with the analogous genes assigned in tomato and potato genomes. This comparison like other comparisons we made, indicated many similarities, and implies a not-surprising close relationship among the three species (Additional file 7). These data points also indicate that we have a good representation of the transcriptome. The number of genes annotated in the pepino was notably lower than in the tomato and potato in very few pathways. This is because some processes may not be properly represented in our samples because they derive from mRNA of three tissues, and we do not have a representation of the whole genome. Other processes instead, are better represented. The results of this comparison is presented in Additional file 7.

### Candidate genes

Several candidate genes related to traits of interest for domestication and breeding were studied. These include genes involved in the inflorescence type, fruit development [49], and synthesis of anthocyanins, chlorogenic acid, saponins, and sucroses. These genes were evaluated for presence or absence in our assembled transcriptome and compared with the transcripts of *S. caripense* (Table 4). The level of similarity given by the score and the e-value is also shown. The number of nucleotidic variants between *S. muricatum* and *S. caripense* was also indicated in order to compare differences between these closely related species.

**Table 4:** Candidate genes studied affecting traits of importance in different Solanaceae. Traits and genes affecting inflorescence, fruit stripes, fruit shape, anthocyanins route, chlorogenic acid pathway, saponines pathway, and sucrose accumulation are included. More information the Candidate genes section of Material and Methods and in Additional file 8: Table S1

Trait	Genes	Features
Inflorescence	Anantha	Gene - F-box protein
	Compound inflorescence	Transcription factor
Fruit stripes	Fruit Stripes	Transcription factor
Fruit shape	FAS	Intron-regulatory
	Fw2.2	Promoter-regulatory
	Fw3	Promoter-regulatory
	Tonneau	Gene - microtubule cytoskeleton organization
	Wuschel (LC)	SNP in downstream-regulatory
	OVATE	Premature stop
	POS1	Intron-regulatory
	SlCCS52A	Receptor activity
	Sl-IAA17	Gene
	SUN	Transposon insertion-regulatory
	Wee	Gene - Kinase
Anthocyanins pathway	F3’5’H	Gene - Hydroxylase
	Acyltransferase-like	Gene - Acyltransferase
	5GT	Gene - Glucosyltransferase
	ANS	Gene - Anthocyanidin synthase
	DFR	Gene - Dihydroflavonol 4-reductase
	F3H	Gene - Flavanone 3-hydroxylase
	CHI	Gene - Chalcone isomerase
	CHS2	Gene - Chalcone synthase
	CHS3	Gene - Chalcone synthase
	CHS1	Gene - Chalcone synthase
	Acyltransferase-like	Gene - Acyltransferase
Chlorogenic acid pathway	4CL	Gene - 4-Coumarate-CoA ligase
	C3H	Cytochrome P450
	HTC	Gene - Transferase
	HQT	Gene - Transferase
Saponines pathway	Egp#1–1	Gene - Glycosyltransferase
	Egp#1–4	Gene - Glycosyltransferase
	Ptt#1–53	Gene - Glycosyltransferase
	Ptt21	Gene - Glycosyltransferase
	Sgt1-1	Gene - Glycosyltransferase
	Ptt#5–30	Gene - Glycosyltransferase
	Sa#6–15	Gene - Glycosyltransferase
	Sk#7–4	Gene - Glycosyltransferase
	Sk#7–4	Gene - Glycosyltransferase
	Sk#7–5	Gene - Glycosyltransferase
	SaGT4A	Gene - Glycosyltransferase
	SaGT4R	Gene - Glycosyltransferase
	SaGT6	Gene - Glycosyltransferase
Sucrose accumulator	TIV1	Gene - Acid invertase

The majority of the genes described in other related species are present in our assembled transcriptome (83.8 %). It should be noted that each of these genes are present too in *S. caripense.* In most cases, there are few differences in these sequences and only nine are identical among the two species. These results are summarized in Additional file 8: Table S1. Interestingly, the greatest differences between the cultigen and the wild species were found in genes related to characters like fruit stripes, anthocyanins and chlorogenic acid synthesis. These differences obviously relate to characters selected in artificial selection by ancient domesticators, and they also offer a clear idea about the variability available in the wild species that may be utilized in pepino breeding.

### Comparison with potato and tomato genomes

**Fig. 5:**
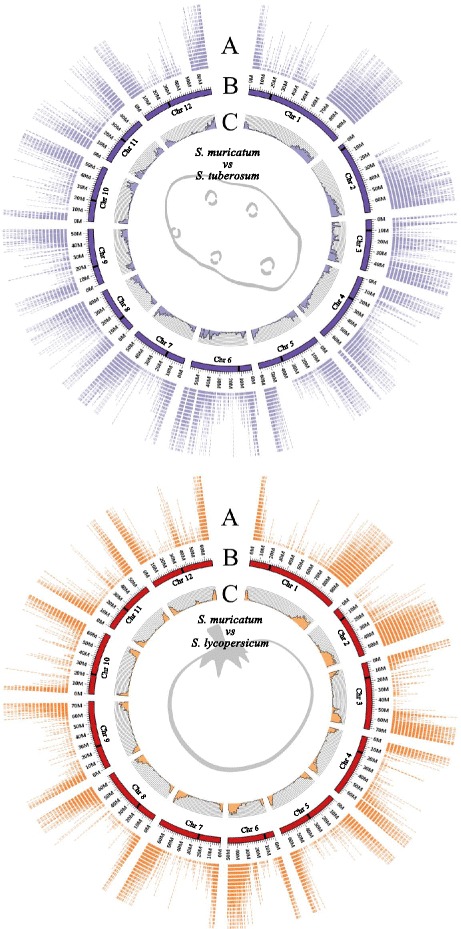
Graphical representation of the pepino unigenes positions on the chromosomes of potato (*top*) and tomato (*bottom*) and nucleotide-level variants distribution/density found on these chromosomes. **a** Distribution of the pepino unigenes on chromosomes, (**b**) ideograms of the 12 chromosomes, black bar indicates the approximated position of the centromere, (**c**) variants distribution/density, every column indicates the number of variants per Mb

All of the most expressed transcripts (75,832) were blasted to the potato genome, resulting in 40,113 (52.9 %) sequences mapped. Furthermore, and similarly, 37,813 (49.9 %) transcripts of pepino were mapped to the tomato genome. These comparisons allowed us to determine the quality of our assembly, to compare the number of genes with the model species (potato and tomato), and to substantially increase our understanding of these newly generated species genomes. In addition to this, the distribution of the pepino unigenes relative to the potato and tomato genomes was plotted using Circos software (Fig. 5). This graphical representation provides visual information of the location of the coding regions, but full information on chromosomal realignment and other changes awaits the development of the sequence of the whole pepino genome.

We have generated gene model predictions comparing our assembled transcriptome of *S. muricatum* with the tomato genome [36]. This alignment of the unigenes to the tomato genomic DNA was performed using the est2genome software, and the ORFs annotations were carried out using ESTScan software [50]. A large number (48,440) of the most expressed transcripts were predicted to have one ORF (63.9 %). On the other hand, we predicted the presence of introns in our unigenes. We found 130,528 in a total of 24,979 unigenes (32.9 %), which means 5.2 introns per unigene, with a maximum number of 19. Knowledge of the positions of these intronic regions is particularly important for discovery of SNPs and INDELs. The previously generated intron map allows us to discard those that are located in the vicinity of an intron, because that would make it difficult to design the primers to amplify these regions. The annotation results (ORFs, introns, descriptions, GO terms, orthologs, nucleotide-level variants and SSR) are deposited in Additional file 4 in GFF3 format.

### Molecular phylogeny among S*olanaceae* species

We used five genes for a phylogenetic study of the pepino and five other Solanaceae crops (potato, tomato, eggplant, capsicum pepper and tobacco). These genes were *waxy* or GBSSI [7, 51], SAMT [52], ADH, β-amylase and CesA [52]. After confirming that these genes are represented in our transcriptome, they were concatenated and aligned using ClustalW. The total length of the sequence analyzed was of 9407 bp including the five genes. Variations among the sequences were found for a total of 1809 positions, of which 507 were parsimony-informative, i.e., these sites contain at least two types of nucleotides, and at least two of them occur with a minimum frequency of two. Results of this alignment are presented in Additional file 9.

**Fig. 6:**
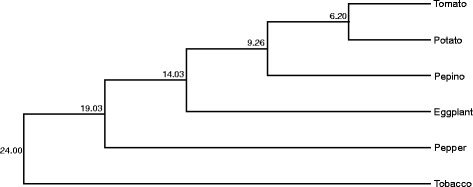
Phylogenetic relationship among Solanaceae species. The number in the nodes indicates the estimated time of divergence (in millions of years). The length of the branches is proportional to the divergence time. Bootstrap values are not shown as it is 100 % in all nodes

After alignment, a phylogenetic tree was constructed among the six species using the software MEGA6 [53]. The statistical method used was the maximum likelihood, but results were similar using other methods like, Neighbor Joining, UPGMA and Maximum Parsimony (data not shown). The estimated divergence time of tomato-potato (5.1 – 7.3 Mya [54]), was fixed at the intermediate value of 6.2 Mya and was used for time calibration. The tree constructed (Fig. 6) shows that the ancestors of pepino and of potato and tomato diverged at around 9.26 Mya. Other divergence estimates are between eggplant, an African member of the family Solanaceae and the rest of the American *Solanum*, occurred 14 Mya.

### SSR and nucleotide-level variants discovery

We performed a general screening on the *S. muricatum* transcriptome for the presence of microsatellites (SSRs), analyzing length, type and quality. We focused in the search for di-, tri- and tetra-nucleotide repeats, with a length limited to 20 repetitions. The total of potential SSRs was 1072 from 1049 unigenes; that is, approximately 1.4 % of the transcripts contain SSRs (Table 5). The maximum and minimum lengths of the SSR repeats were 48 and 17 respectively (the average length was 21 nucleotides). Tri-nucleotide repeats (707) were the most commonly found repetitions in our transcriptome accounting for almost 66 % of the SSRs identified. The most common motif was AAG (191) representing 17.8 % of the tri-nucleotide SSRs; other common repeats were AG (123) representing 11.5 %, and AAC representing the 9.6 % of the tri-nucleotide SSRs. Others motifs found in our analysis are summarized in Table 5. The completed list of SSRs and their characteristics are provided in the Additional file 10.

**Table 5:** Single sequence repeat (SSR) statistics according to the type of motif, the percentage of each motif and the amount of unigenes with SSRs. Complete information about these markers is shown in Additional file 10

Di-nucleotide motif	Number of Di-SSR	%	Unigenes
AG	123	48.62	
AT	101	39.92	
AC	29	11.46	
Total	253	100	253
Tri-nucleotide motif	Number of Tri-SSR	%	Unigenes
AAG	191	27.02	
AAC	103	14.57	
AAT	101	14.29	
ATC	93	13.15	
ACC	68	9.61	
AGG	56	7.92	
AGC	50	7.07	
ACT, ACG, CCG	45	6.36	
Total	707	100	684
Tetra-nucleotide motif	Number of Tetra-SSR	%	Unigenes
AAAT	28	25.00	
AAAC	17	15.18	
AAAG	16	14.29	
AGAT	10	8.93	
AATC	8	7.14	
Others tetra-nucleotides	33	29.46	
Total	112	100	112

High throughput sequencing of both transcriptomes has made possible to obtain a large collection of SNPs and INDELs. The variant calling was carried out using the default parameters recommended by the Freebayes software [55], which allows distinguishing and recognizing sequence variations from sequencing errors and mutations introduced during cDNA synthesis. The implementation of several filters described in the Methods has also allowed obtaining markers of potentially high quality, allowing their use in high throughput genotyping platforms [34]. Apart from this, the CAPS filter can be especially useful when other methods for SNPs detection are not available.

Applying these criteria, we identified a total of 11,735 SNPs and 766 INDELs in *S. muricatum* (SL), and 30,668 SNPs and 1494 INDELs in *S. caripense* (EC-40), as well as 84,972 SNPs and 4058 INDELs shared by both species (interspecific) (Table 6). Within the detected SNPs, as usual, transitions (62.9 %) were much more abundant than transversions (36.9 %), given that transitions are less likely to result in amino acid substitutions, and are therefore more likely to persist as silent substitutions [56]. Within transitions and transversions, both, A/T and C/T were equally abundant (Table 7). The complete list of these SNPs is provided in Additional file 11.

**Table 6:** Single nucleotide variants statistics for the *S. muricatum* and *S. caripense* transcriptomes

Species	SNPs	INDELs
*Solanum muricatum* (SL)	11,735	766
*Solanum caripense* (EC-40)	30,668	1494
*S. muricatum* (SL) vs. *S. caripense* (EC-40)	84,972	4058

**Table 7:** Single nucleotide polymorphism (SNPs) statistics. Type and number of transitions and transversions are shown for high quality SNPs identified in each species and between them

SNPs	Number (%)	SNPs	Number (%)	
Transitions		Transversions		Complex
A<->G	26,684 (31.4)	A<->T	8514 (10.0)	212
C<->T	26,806 (31.5)	G<->T	8311 (9.8)	
		C<->G	6250 (7.3)	
		A<->C	8288 (9.7)	
Total	53,490 (62.9 %)	Total	31,363 (36.9 %)	212 (0.3 %)

In order to determine their position, heterozygous intra and interspecific nucleotide-level variants were located in the tomato and potato genomes using the comparison files explained above. This analysis is summed up in Table 8, where we indicate the number of nucleotide-level variants predicted for every chromosome as well as their hypothetical position and density on chromosomes (using the Circos plot; diagram C in Fig. 5).

**Table 8:** Distribution of pepino nucleotide-level variants on chromosomes of tomato and potato

	Tomato	Potato
Chromosome 1	2931	3031
Chromosome 2	2529	2619
Chromosome 3	2441	2360
Chromosome 4	1984	2151
Chromosome 5	1548	1678
Chromosome 6	2086	2204
Chromosome 7	1867	1845
Chromosome 8	1628	1637
Chromosome 9	1610	1717
Chromosome 10	1636	1656
Chromosome 11	1530	1698
Chromosome 12	1608	1846
Total	23,501	24,442

## Discussion

We employed NGS technology for the first time for sequencing the transcriptome of pepino and its related wild species *S. caripense*. There is no reference genome for these species, consequently we used successfully the *de novo* assembly method [45, 57]. Using this methodology a large number of most expressed transcripts or unigenes (75,832) were obtained, with an average length of 704 bp. We were pleased that the number of unigenes obtained in this project is similar to or better than that obtained in previous studies using similar technologies, thus demonstrating the quality and potential utility of our work, both in sample preparation and assembling protocol [37, 38]. The high initial quality of the unigenes we have obtained is of fundamental importance for the remainder of the work.

The mRNA-Seq methodology gives the G/C content (ratio of guanine and cytosine) among unigenes. As a consequence of the nature of the chemical bond between G and C, this base pair is considered more stable than the A/T base pair. Thus, during evolution, variation in the G/C content would accumulate more slowly (although this assertion has been contested [39]). Accordingly, given the G/C content, markedly variable among different organisms [58], would be an indicator of closeness between species. The G/C contents obtained (41.7 % in *S. muricatum* and 42.5 % in *S. caripense*) were consistent with values found in other *Solanum* [31]. In particular, the tomato G/C content for cDNA reported in previous studies was 40.3 % [59], and for potato, the value is similar (43.1 % [60]). This suggest that the *S. muricatum* transcriptome represents a typical example of a Solanaceae transcriptome, thereby raising the probability of successfully employing our data for broader comparative studies in this genus.

The assembled unigenes were functionally annotated in order to better understanding the role of the genes represented. The knowledge of the functional role of the genes increases the probability of utilizing them for pepino breeding and demonstrates the possible ways in which the wild relatives of pepino could be of interest in developing new varieties. The annotation percentages in the different protein databases were similar to those obtained in other similar projects [43–45]. Most of the sequences were annotated in Swiss-Prot [40]. Since SwissProt is a highly-curated, highly-crossreferenced, and non-redundant database, that has a lower error rate than the automatically created databases, which gives high annotation reliability. The percentage of protein annotations in the other databases (ITAG2.4 [41] and UniRef90 [42]) was smaller, as expected. The GO annotation indicated that the majority of the unigenes were involved in molecular, cellular and biological processes. Within the molecular function category, the majority of the unigenes were assigned to different binding processes, hydrolase activity and catalytic activity. The majority of genes in the cellular component sequences functioned in cell and organelle structures (Fig. 3 and Additional file 6).

The KEGG pathway database is a resource for the systematic analysis of gene functions in terms of networks of genes and molecules in cells and their variants specific to particular organisms [61]. In our case, this analysis included 144 pathways involving 16,027 unigenes, importantly including pathways key to success in a dessert fruit like pepino. For example we had representation of important pathways like starch and sucrose metabolism, biosynthesis of carotenoids and anthocyanins and several vitamins (B1, B2, B6, H and A). Identifying changes in these genes and associating them with phenological differences will enable us to more efficiently manage future breeding programs involving these species [3]. Perhaps because we used transcripts representing only three tisues (i.e., not the whole genome), the comparison of the KEGG pathways we identified with those obtained from the potato and tomato genomes shows that some processes were not very well represented in the pepino or *S. caripense*. Despite this, our transcriptome data are an excellent representation of the metabolic processes that, with further analysis of these pathway-related genes, will improve our understanding of the pepino features, some of them unique and others shared with the rest of the Solanaceae [23, 62]. Obviously, the transcriptome data can contribute to expanding and enhancing the breeding resources for and with these species.

As a preliminary test of the application of our results to breeding programs, a set of candidate genes described from other Solanaceae were evaluated for their presence in the pepino transcriptome, and the associated nucleotide changes in *S. caripense*. These genes selected were either characters associated with domestication or those with potential for enhancement through breeding. The caveats we worked with in running these preliminary tests included recognition that there are large differences in nucleotide changes between *S. muricatum* and *S. caripense* in some characters of interest, like anthocyanins and chlorogenic acid synthesis [63–65]. There are significant morphological and fruit composition differences between the pepino and wild *S. caripense*; for example, differences related to plant habit, leaf complexity, trailing habit and seediness of the fruits [13, 66]. Correspondingly, our data demonstrate the existence of many differences at genomic level, including genes that are of great interest for breeding. Thus, we think this foundational work will provide the basis for broader studies, such as those where an in-depth and accurate phenotypic characterization can be related to changes at the nucleotide level. These kinds of studies may be helpful for understanding the genetics of these key characters, for providing a foundation for positive selection in the domestication process, and for establishing a breeding program [32, 67, 68]. For example, we have found concentrations of chlorogenic acid, a powerful bioactive molecule with application to human health as an antioxidant [53, 54], to be much higher in *S. caripense* than in the cultivated *S. muricatum* (unpublished data). Sequence differences found can be used as functional markers for marker-assisted breeding to transfer alleles from *S. caripense* to pepino. In this regard, differentially expressed genes (DEG) analysis [69], could be interesting for clearing up differences at the expression level in several developmental stages and tissues, which may provide relevant information about pepino domestication. Furthermore, the information obtained may be used for tomato or potato improvement in the near future using modern technologies for gene editing like CRISPR/Cas [70], or by transformation using cisgenic approaches [71].

Another issue to consider in a *de novo* assembly of a transcriptome is the genic structure. In this regard, we have generated gene model predictions comparing our assembled transcriptome of *S. muricatum* with the tomato genome [36]. Because these two species are very closely related [6, 7], this is a valid approach until, full genome sequencing is available.

Other genes present in our assembled transcriptome are used to study phylogenetic relationships within this large, and economically important, family (for example: e *waxy* or GBSSI [7, 51], SAMT [52], ADH, β-amylase and CesA [52]), and the sequence differences in pepino with other Solanaceae were used to elucidate its relationship. Further confidence in our results comes from the fact that they are consistent with previous studies such as Spooner *et al.* [6], Wang *et al.* [54] and Garzon-Martinez *et al.* [48], and the fact that the divergence times estimated are congruent with data deposited in TimeTree [72] (a public knowledge-base of divergence times among organisms). Our data indicate that the divergence among all the Solanaceae studied here (pepino, tomato, potato, eggplant, pepper and tobacco) occurred in the last 24 million years. The pepino and the tomato-potato clade shared a common ancestor from which they diverged 9.26 Mya. Other divergence estimates indicate that the eggplant, an African member of the family Solanaceae, and the rest of the American *Solanum*, occurred 14 Mya.

The total of potential SSRs obtained was 1072 in 1049 unigenes; that is, approximately 1.4 % of the transcripts contain SSRs (Table 5). The number of SSRs are slightly lower than expected, or at least lower than obtained in similar studies [73, 74]. This may be due to the application of strongest criteria on our study, the advantage being that we should have obtained markers of better quality. In any case, the number of markers is adequate to develop a high density genetic map, and for genetic diversity studies and marker assisted breeding studies.

As stated above, tri-nucleotide repeats were the most commonly found repetitions in our transcriptome accounting for almost 66 % of the SSRs identified. The tri-nucleotide repeats may be the most common because these SSRs do not change the frameshift and mutations have less dramatic effect [75]. There is considerable evidence that genic SSRs have important functions. For example, it has been postulated that SSRs may affect chromatin organization, and they also may be related to regulation of gene activity, recombination, and DNA replication [76]. Extra-genic SSR markers have several advantages beyond genomic SSRs because they are related to codifying sequences, and thus can be used as candidate genes to study association with phenotypic variation. Extra-genic SSR markers can be useful for genetic diversity studies, as demonstrated for pepino when using tomato EST-SSRs [13], for the development of genetic maps and for fingerprinting commercial cultivars, breeding lines or landraces [77].

By applying several bioinformatic approaches, we obtained a total of 11,735 SNPs and 766 INDELs in *Solanum muricatum*, and 30,668 SNPs and 1494 INDELs in *S. caripense*, as well as 84,972 SNPs and 4058 INDELs between the two species (interspecific) (Table 6). These nucleotide-level variant markers show that both clones present an important degree of heterozygosis, although the highest number of intraclone markers was obtained in *S. caripense*. This makes sense, given that *S. caripense* is an obligately allogamous wild species with a gametophytic self-incompatiblity system [18, 78, 79].

The large number of SNPs markers developed can be readily used in pepino research. These markers exhibit co-dominant inheritance and due to their abundance, they are widely used for different applications. Some of these applications include: diversity studies, development of saturated molecular genetic and physical maps, identification of QTLs or genes controlling traits of economic importance, marker-assisted selection, or association mapping with genome-wide association studies (GWAS) [80].

## Conclusions

This study constitutes the first genomic resource for pepino, a cultigen closely related to tomato and potato [6, 62]. These genomic studies are especially important because they promote the understanding of crop evolution in this group, and pepino enhancement. Furthermore, because the pepino is part of the tertiary genepool of tomatoes and potatoes [6], these genomic studies, provide a wide array of genomic information that may be useful for breeding in those groups as well. The high quality of the transcriptome presented here will enhance comparative studies within the genus *Solanum*, and will be useful for future annotations of the *S. muricatum* genome sequence. The detailed annotation provided in this work will facilitate the use of sequenced unigenes for gene discovery, in particular for traits of interest within pepino (such as soluble solids content, chlorogenic acid content and fruit size). In addition to the pepino, sequencing of the transcriptome of its sister wild relative *S. caripense* has allowed identification of a large number of molecular markers (SSRs and nucleotide-level variants), within each species, as well as between them. The filtering process applied in the search of these variants has facilitated the selection of the most suitable markers for high throughput genotyping platforms. Our results are an example of how high throughput sequencing technologies can contribute to knowledge of domesticates with no or limited genomic information, but where there are closely related species with the whole genome sequence available. Our assembled transcriptome, and the large collection of markers found, will enhance pepino breeding, facilitate molecular studies in this crop, and will be useful to develop the first genetic map of the pepino. Ultimately, the genomic information obtained will be of interest for tomato and potato breeding and for studying genomic changes during evolution and crop domestication in these important crops.

## Methods

### Plant material

**Fig. 7:**
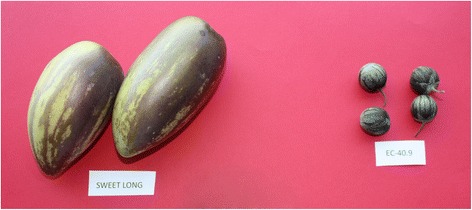
Fruits of *Solanum muricatum* var. Sweet Long (SL) (*left*) and of one of the wild relatives, *S. caripense* (EC-40) (*right*)

Plant material used consisted of the clonal pepino cultivar Sweet Long (SL) [81] and one clone obtained by vegetative propagation of a single plant of accession EC-40 of *S. caripense*, which was originally collected in Ecuador [66]. Plants for both have been maintained in *in vitro* culture at the Institute for Conservation and Improvement of Valencian Agrodiversity (COMAV). SL and EC-40 have contrasting phenotypes for many traits (Fig. 7), some of them interesting for pepino breeding fruit size (greater in SL), shape (elongated in SL and round in EC-40) and solid soluble content (SSC) (higher in EC-40) [13]. Five clonal replicates of each accession were acclimated and grown in a glasshouse in Valencia - Spain (GPS coordinates: lat. 39°29’01” N, long. 0°20’27” W) with quartz sand as substrate and under controlled conditions. From each accession, tissue was sampled from young leaves, flowers in the pre-anthesis stage, and from mature fruit. All samples taken were immediately frozen in liquid nitrogen and stored at −80 °C until used.

### RNA preparation, Illumina paired-end cDNA library construction and sequencing

Total RNA was extracted from each tissue using the TRI reagent (Sigma-Aldrich, St. Louis, USA). RNA integrity was confirmed by agarose electrophoresis, and RNA quantification was performed using a Nanodrop Spectrophotometer ND-1000 (Thermo Scientific, Wilmington, USA). For each of the two accessions, we combined equivalent amounts of RNA from each tissue into two pools. A total of 10 μg of total RNA for each pool was sent to Macrogen Korea (Seoul, South Korea) for Illumina RNA-seq performed in HiSeq 2000 sequencer (Illumina, San Diego, USA).

The cDNA library was constructed according to the manufacturer’s instructions (Illumina/Hiseq-2000 RNA-seq) by Macrogen Korea. Essentially, the mRNA molecules containing poly (A) were purified using Sera-mag Magnetic Oligo (dT) Beads from the RNA samples. A fragmentation buffer was added to break the mRNA into small fragments. Using these fragments as templates, the first strand of cDNA was synthesized. The second strand of cDNA was synthesized using the buffers containing dNTPs, RNase H, and DNA polymerase I. The synthesized cDNA was purified and connected with the sequencing adapters. Finally, a range of cDNA fragments (200 ± 25 bp) were excised from an agarose gel using a gel extraction kit. Then, the library was sequenced using the Illumina/Hiseq-2000 RNA-seq. These raw sequences are available at the NCBI Sequence Read Archive (SRA) as stated in the section titled, “Availability of Data and Material”.

### DNA sequence processing and *de novo* transcriptome assembly

**Fig. 8:**
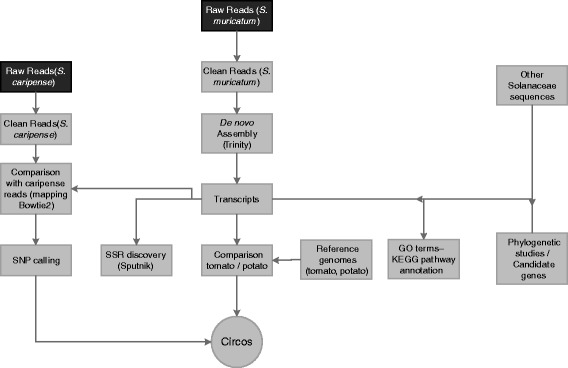
Schematic representxation of the overall sequencing and annotation workflow for the pepino transcriptome

The pipeline used for the bioinformatics analysis is shown in Fig. 8. After receiving the files with raw data, we used the software FastQC [82] to evaluate the quality of both samples.

In the case of *S. muricatum* Sweet Long, we found two sequences overexpressed after initial quality filtering. Blast against databases (NCBI-GenBank) showed that these sequences belong to the Pepino mosaic virus (PepMV), although plants were asymptomatic. These reads were eliminated using Bowtie2 [83]. We did not find other sequences overexpressed that would indicate the presence of more contaminants.

High quality reads are required for better assembling. We performed the following processes: trimming of adapter contamination, filtering of reads with “N” and trimming of low quality nucleotides Q ≥ 20 using NGS_CRUMBS (http://bioinf.comav.upv.es/ngs_crumbs).

We used Trinity software [26, 27] to build the primary assembly. This first assembly was post-processed with the following steps. First, we reduced the redundancy using CAP3 [76]. Then we removed low complex transcripts using DUST score. Next, we split some of the subcomponents into new subclusters, using blast and transitivity properties, as a way to enhance the results previously obtained. In this way if one transcript is similar to a second, and this second is similar to a third and so on, all of them are the same unigene, and they can be merged. For this purpose we used a handmade script. Finally, we removed low expression transcripts using RSEM (RNA-Seq by Expectation-Maximization) [84]. From the final assembly, we made a subset selecting only the most expressed transcript from each Trinity transcript cluster. A detailed explanation of the steps undertaken in the post-processed stage is shown in Additional file 12.

### Structural and functional annotation

Annotation of the assembled transcript sequences was performed using the bioinformatic application ngs_backbone [85], based in the BLASTX algorithm [39] against different databases. The order was established prioritizing handmade annotation databases. Accordingly, the databases used, in order, were Swiss-Prot [40], ITAG2.4 [41], and UniRef90 [42]. This analysis was released on February 2015. The first analysis compared all transcripts with the first database, the second compared transcripts not paired in the preceding and so on. A typical blast cut-off e-value of 1e^−20^ was used and other details of this analysis are showed in Additional file 12.

Additionally, we performed a functional classification of the transcripts following the Gene Ontology (GO) scheme using Blast2GO [86]. This analysis covers three steps as follows: (1) sequence alignment via Blastx with the NR (Non Redundant) database (cut-off e-value of 1e^−20^), (2) gene ontology mapping, and (3) functional annotation, including molecular functions, biological processes, and cellular components [86]. In this case, to sum up the functional information of our pepino transcriptome, we performed a plant specific GO slim. Additionally, when possible, Blast2GO gives an Enzyme Commission number (EC number). Meanwhile, KEGG pathways were retrieved from the Kyoto Encyclopaedia of Genes and Genomes (KEGG) database (version 73.0, January 1, 2015). This KEGG analysis includes a collection of manually drawn pathway maps representing experimental knowledge on metabolism and various other functions of the cell and the organism.

### Candidate genes

Taking the pepino transcriptome as a reference database, we evaluated the sequences of several genes associated with breeding characters of interest found in others related species. In total, we selected: 12 genes related to fruit shape [49], two related to inflorescence type [87], 11 with the anthocyanins synthesis route [88], 13 related to the synthesis of saponines [89], four with the chlorogenic acid synthesis pathway [90], one with sucrose accumulation [91] and one related to fruit stripes [92]. Some of these genes are part of genic families; consequently we evaluated the principal gene and the rest of its family. The total number of sequences evaluated was 115. Description of the genes and their features are shown in Table 4 and in Additional file 8: Table S1.

Using Blastn (cut-off of 1e^−60^), these genes were compared with pepino unigenes to determine its presence or absence in our assembled transcriptome. Once defined as part of our transcriptome, they were compared with the transcripts of *S. caripense* in order to recognize nucleotide variants between these two species.

### Comparison with tomato and potato genomes

The whole of the most expressed transcripts were compared to the *S. tuberosum* and *S. lycopersicum* genomes using Blastn (cut-off value of 1e^−20^) in order to obtain the physical position of our assembled sequences. Gene model prediction was performed using the Est2genome software [93, 94] which allows EST sequences to be aligned with genomic DNA sequences with high efficiency. The gene model prediction takes place by sequence homology with the tomato genome. Additionally we used the open reading frame detector ESTScan [50] for annotation of ORFs.

Circos, software that allows visualization of data and information in a circular layout [95], was used to represent our sequences over the tomato and potato reference genomes; this enabled visual estimation of the distribution of our codifying sequences.

### Molecular phylogeny between S*olanaceae* species

Using sequence data available in databases, we chose five nuclear protein-coding genes to investigate phylogenetic relationships with five of the most important Solanaceae crops (potato, tomato, eggplant, pepper and tobacco), in addition to the pepino. These genes were: (1) the widely used granule-bound starch synthase gene (*waxy* or GBSSI) [7, 51], (2) the salicylic acid methyltransferase gene (SAMT) [52], (3) the alcohol dehydrogenase gene (ADH), (4) the β-amylase gene, and (5) the cellulose synthase gene (CesA) [52]. Once isolated, the genes were concatenated one after the other and aligned using ClustalW2, a multiple sequence alignment program [96]. The alignment file generated was used to build a phylogenetic tree using the maximum likelihood distance with 500 bootstrap replications using MEGA6 [53]. Divergence times were estimated with the same program, and the tomato/potato split (5.1–7.3 million years ago) was used for time calibration [54].

### SSR and nucleotide-level variants discovery

Mapping reads of *S. caripense* to reference transcriptome of *S. muricatum.* Mining SSRs was carried out using the Sputnik software [97], specially designed for this function. Once the contigs with SSRs were isolated, they were filtered by quality, closeness to introns, number of repetitions and position in the genome of tomato.

Nucleotide-level variants calling (SNPs and INDELs) was performed comparing the assembled transcriptome of *S. muricatum* with the clean reads of both species. We mapped the reads with Bowtie2. For SNPs and INDELs calling we used Freebayes [55]. Several filters, shown in Additional file 11, were applied in order to maximize the successful validation and its future use in high throughput genotyping platforms. First, filters IV0, IV1 and IV2 were used to select the variants in and between the two species. The filter vks was used to select authentic SNPs on the one hand and INDELs on the other. Other filters were used for optimizing their future use in high throughput genotyping platforms (Additional file 8: Table S1). Circos [95] was also used for positioning the density (variants per Mb) and distribution of all these markers over both reference genomes.

### Availability of data and materials

The raw reads data are available at NCBI Sequence Read Archive (SRA) with accession number SRS1052501 (*S. muricatum*) and SRS1054035 (*S. caripense*), available at http://www.ncbi.nlm.nih.gov. The transcriptome assembly of *S. muricatum* is deposited into the NCBI Transcriptome Shotgun Assembly (TSA) repository within the bioproject number PRJNA294064.

## Additional files

Additional file 1: Figure S1. Boxplots indicating the quality scores across all bases in *S. muricatum *(left) and in *S. caripense* (right) (Word file). Horizontal axis represents the base position in base pair. Vertical axis represents quality score (Q). (DOCX 32 kb) Additional file 2:
*Solanum muricatum* assembled transcripts (Fasta format zip comprised). The fasta file provides the sequence of the 91,949 *S. muricatum* transcripts. (ZIP 20596 kb) Additional file 3:
*Solanum muricatum* most expressed transcripts or unigenes (Fasta format zip comprised). The fasta file provides the list of the 75,832 most expressed single-copy *S. muricatum* transcripts. (ZIP 15757 kb) Additional file 4: Annotation results in GFF3. All the annotation results (ORFs, introns, descriptions, GO terms, orthologs and markers) provided in the standard format GFF3. (ZIP 5290 kb) Additional file 5: Figure S2. Distribution of GO terms (pdf image). The unigenes distribution regarding the quantity of GO terms to which they are assigned. (PDF 17 kb) Additional file 6: GO annotation. A text file with the GO annotation for the whole *S. muricatum* unigene collection. (ZIP 1223 kb) Additional file 7: KEGG pathway annotation. A zip compressed file with a list of KEGGs pathways, graphics in png format, and a file with a comparison with KEGGs pathways of potato and tomato. (ZIP 4361 kb) Additional file 8: Table S1. Candidate genes list and features. A Word file with a description of the candidate genes used, the name of the origin sequences, the name of the pepino unigenes and the number of variants in these unigenes between pepino and *S. caripense*. (DOCX 26 kb) Additional file 9: ClustalW alignment. Text file with multiple alignment from the concatenation of the five genes studied in pepino, tomato, potato, eggplant, pepper and tobacco. (DOCX 34 kb) Additional file 10: List and features of SSRs. An Excel file that provides a list of the SSRs identified in the pepino transcripts, including information about the type and numbers of repetitions, and in which transcript they are present. (ZIP 73 kb) Additional file 11:
**1**: Nucleotide-level variants calling filters, list and features in VCF format– Part 1. A zip compressed file with the first part of a list of all the variants identified in the pepino transcripts, including their positions and the nucleotide changes. **2**: Nucleotide-level variants calling filters, list and features in VCF format– Part 2. A zip compressed file with the second part of a list of all the variants identified in the pepino transcripts, including their positions and the nucleotide changes. (ZIP 51450 kb) Additional file 12: Computational steps, software and their parameters used. A Word file with a description of the software used for the assembly process and annotation. (DOCX 16 kb)

## Abbreviations

AFLP: amplified fragment length polymorphism
COS: conserved ortholog set
CRISPR: clustered regularly interspaced short palindromic repeats EC, Enzyme commission
EST: expressed sequence tag
GO: gene ontology
KEGG: Kyoto Encyclopedia of Genes and Genomes
Mya: million years ago
ORF: open reading frame
QTL: quantitative trait locus
RAPD: random amplification of polymorphic DNA
SNP: single nucleotide polymorphism
SSC: soluble solid content
SSR: simple sequence repeat

## References

[1] Anderson GJ, Jansen RK, Kim Y (1996). The origin and relationships of the pepino, *Solanum muricatum* (Solanaceae): DNA restriction fragment evidence. Econ Bot.

[2] Anderson GJ, Martine CT, Prohens J, Nuez F (2006). *Solanum perlongistylum* and *S. catilliflorum*, new endemic Peruvian species of *Solanum*, Section *Basarthrum*, are close relatives of the domesticated pepino, *S. muricatum*. Novon.

[3] Rodríguez-Burruezo A, Prohens J, Fita AM (2011). Breeding strategies for improving the performance and fruit quality of the pepino (*Solanum muricatum*): A model for the enhancement of underutilized exotic fruits. Food Res Int.

[4] Yalçin H (2010). Effect of ripening period on composition of pepino (*Solanum muricatum*) fruit grown in Turkey. Afr J Biotechnol.

[5] Abouelnasr H, Li Y-Y, Zhang Z-Y, Liu J-Y, Li S-F, Li D-W, Yu J-L, McBeath JH, Han C-G (2014). First Report of Potato Virus H on *Solanum muricatum* in China. Plant Dis.

[6] Spooner DM, Anderson GJ, Jansen RK (1993). Chloroplast DNA evidence for the interrelationships of tomatoes, potatoes, and pepinos (Solanaceae). Am J Bot.

[7] Sarkinen T, Bohs L, Olmstead RG, Knapp S (2013). A phylogenetic framework for evolutionary study of the nightshades (Solanaceae): a dated 1000-tip tree. BMC Evol Biol.

[8] Nakitandwe J, Trognitz FCH, Trognitz BR (2006). Genetic mapping of *Solanum caripense*, a wild relative of pepino dulce, tomato and potato, and a genetic resource for resistance to potato late blight. VI International Solanaceae Conference: Genomics Meets Biodiversity 745.

[9] Sakomoto K, Taguchi T (1991). Regeneration of intergeneric somatic hybrid plants between *Lycopersicon esculentum* and *Solanum muricatum*. Theor Appl Genet.

[10] Bernardello LM, Anderson GJ (1990). Karyotypic studies in *Solanum* section *Basarthrum* (Solanaceae). Am J Bot.

[11] Arumuganathan K, Earle ED (2004). Nuclear DNA content of some important plant species. Plant Mol Biol Report.

[12] Spooner DM, Rodríguez F, Polgár Z, Ballard HE, Jansky SH (2008). Genomic origins of potato polyploids: GBSSI gene sequencing data. Crop Sci.

[13] Herraiz FJ, Vilanova S, Andújar I, Torrent D, Plazas M, Gramazio P, Prohens J (2015). Morphological and molecular characterization of local varieties, modern cultivars and wild relatives of an emerging vegetable crop, the pepino (*Solanum muricatum*), provides insight into its diversity, relationships and breeding history. Euphytica.

[14] Trognitz FC, Trognitz BR (2005). Survey of resistance gene analogs in *Solanum caripense*, a relative of potato and tomato, and update on R gene genealogy. Mol Genet Genomics.

[15] Hajjar R, Hodgkin T (2007). The use of wild relatives in crop improvement: a survey of developments over the last 20 years. Euphytica.

[16] Doebley JF, Gaut BS, Smith BD (2006). The molecular genetics of crop domestication. Cell.

[17] Blanca JM, Prohens J, Anderson GJ, Zuriaga E, Canizares J, Nuez F (2007). AFLP and DNA sequence variation in an Andean domesticate, pepino (*Solanum muricatum*, Solanaceae): implications for evolution and domestication. Am J Bot.

[18] Rodríguez-Burruezo A, Prohens J, Nuez F (2003). Wild relatives can contribute to the improvement of fruit quality in pepino (*Solanum muricatum*). Euphytica.

[19] Herraiz FJ, Villaño D, Plazas M, Vilanova S, Ferreres F, Prohens J, Moreno DA (2016). Phenolic profile and biological activities of the pepino (Solanum muricatum) fruit and its wild relative S. caripense. Int J Mol Sci.

[20] Leiva-Brondo M, Prohens J, Nuez F (2006). Characterization of pepino accessions and hybrids resistant to Tomato mosaic virus (ToMV). J Food Agric Env.

[21] Nakitandwe J, Trognitz F, Trognitz B (2007). Reliable allele detection using SNP-based PCR primers containing Locked Nucleic Acid: application in genetic mapping. Plant Methods.

[22] Andrivon D (1996). The origin of Phytophthora infestans populations present in Europe in the 1840s: a critical review of historical and scientific evidence. Plant Pathol.

[23] Prohens J, Ruiz JJ, Nuez F (1996). The pepino (*Solanum muricatum*, Solanaceae): A “new” crop with a history. Econ Bot.

[24] Heiser CB (1964). Origin and Variability of the Pepino (*Solanum Muricatum*). Preliminary Report.

[25] Ahmad H, Khan A, Muhammad K, Nadeem MS, Ahmad W, Iqbal S, Nosheen A, Akbar N, Ahmad I, Que Y (2014). Morphogenetic study of pepino and other members of solanaceae family. Am J Plant Sci.

[26] Haas BJ, Papanicolaou A, Yassour M, Grabherr M, Blood PD, Bowden J, Couger MB, Eccles D, Li B, Lieber M (2013). De novo transcript sequence reconstruction from RNA-seq using the Trinity platform for reference generation and analysis. Nat Protoc.

[27] Grabherr MG, Haas BJ, Yassour M, Levin JZ, Thompson DA, Amit I, Adiconis X, Fan L, Raychowdhury R, Zeng Q (2011). Full-length transcriptome assembly from RNA-Seq data without a reference genome. Nat Biotechnol.

[28] Wang Z, Gerstein M, Snyder M (2009). RNA-Seq: a revolutionary tool for transcriptomics. Nat Rev Genet.

[29] McKain MR, Wickett N, Zhang Y, Ayyampalayam S, McCombie WR, Chase MW, Pires JC, de Pamphilis CW, Leebens-Mack J (2012). Phylogenomic analysis of transcriptome data elucidates co-occurrence of a paleopolyploid event and the origin of bimodal karyotypes in Agavoideae (Asparagaceae). Am J Bot.

[30] Barker MS, Vogel H, Schranz ME (2009). Paleopolyploidy in the Brassicales: analyses of the Cleome transcriptome elucidate the history of genome duplications in *Arabidopsis* and other Brassicales. Genome Biol Evol.

[31] Rensink W, Lee Y, Liu J, Iobst S, Ouyang S, Buell CR (2005). Comparative analyses of six solanaceous transcriptomes reveal a high degree of sequence conservation and species-specific transcripts. BMC Genomics.

[32] Koenig D, Jimenez-Gomez JM, Kimura S, Fulop D, Chitwood DH, Headland LR, Kumar R, Covington MF, Devisetty UK, Tat AV, Tohge T, Bolger A, Schneeberger K, Ossowski S, Lanz C, Xiong G, Taylor-Teeples M, Brady SM, Pauly M, Weigel D, Usadel B, Fernie AR, Peng J, Sinha NR, Maloof JN (2013). Comparative transcriptomics reveals patterns of selection in domesticated and wild tomato. Proc Natl Acad Sci U S A.

[33] Blanca JM, Cañizares J, Ziarsolo P, Esteras C, Mir G, Nuez F, Garcia-Mas J, Picó MB (2011). Melon transcriptome characterization: Simple sequence repeats and single nucleotide polymorphisms discovery for high throughput genotyping across the species. Plant Genome.

[34] Blanca J, Canizares J, Roig C, Ziarsolo P, Nuez F, Pico B (2011). Transcriptome characterization and high throughput SSRs and SNPs discovery in *Cucurbita pepo* (Cucurbitaceae). BMC Genomics.

[35] Howe GT, Yu J, Knaus B, Cronn R, Kolpak S, Dolan P, Lorenz WW, Dean JF (2013). A SNP resource for Douglas-fir: de novo transcriptome assembly and SNP detection and validation. BMC Genomics.

[36] Consortium TG (2012). The tomato genome sequence provides insights into fleshy fruit evolution. Nature.

[37] Potato Genome Sequencing Consortium (2011). Genome sequence and analysis of the tuber crop potato. Nature.

[38] Anderson GJ, Jansen RK (1994). Biosystematic and molecular systematic studies of *Solanum* section *Basarthrum* and the origin and relationships of the pepino (*S. muricatum*). Proceedings of the VI Congreso Latinoamericano de botanica: Mar del Plata, Argentina.

[39] Altschul SF, Madden TL, Schaffer AA, Zhang J, Zhang Z, Miller W, Lipman DJ (1997). Gapped BLAST and PSI-BLAST: a new generation of protein database search programs. Nucleic Acids Res.

[40] Swiss Prot [http://web.expasy.org/docs/swiss-prot_guideline.html]. Accessed 29 Apr 2016.

[41] SGN release versionITAG2.4 [ftp://ftp.sgn.cornell.edu/tomato_genome/annotation/]. Accessed 29 Apr 2016.

[42] Uniref [http://www.ebi.ac.uk/uniprot/database/download.html]. Accessed 29 Apr 2016.

[43] Wei D-D, Chen E-H, Ding T-B, Chen S-C, Dou W, Wang J-J (2013). De novo assembly, gene annotation, and marker discovery in stored-product pest *Liposcelis entomophila* (Enderlein) using transcriptome sequences. PLoS One.

[44] Li D, Deng Z, Qin B, Liu X, Men Z (2012). De novo assembly and characterization of bark transcriptome using Illumina sequencing and development of EST-SSR markers in rubber tree (*Hevea brasiliensis* Muell. Arg.). BMC Genomics.

[45] Lulin H, Xiao Y, Pei S, Wen T, Shangqin H (2012). The first Illumina-based de novo transcriptome sequencing and analysis of safflower flowers. PLoS One.

[46] Mitraki A, Barge A, Chroboczek J, Andrieu JP, Gagnon J, Ruigrok RWH (1999). Nomenclature committee of the international union of biochemistry and molecular biology (NC-IUBMB). Eur J Biochem.

[47] Sierro N, Battey JN, Ouadi S, Bovet L, Goepfert S, Bakaher N, Peitsch MC, Ivanov NV (2013). Reference genomes and transcriptomes of *Nicotiana sylvestris* and *Nicotiana tomentosiformis*. Genome Biol.

[48] Garzon-Martinez GA, Zhu ZI, Landsman D, Barrero LS, Marino-Ramirez L (2012). The *Physalis peruviana* leaf transcriptome: assembly, annotation and gene model prediction. BMC Genomics.

[49] Wang L, Li J, Zhao J, He C (2015). Evolutionary developmental genetics of fruit morphological variation within the Solanaceae. Front Plant Sci.

[50] Iseli C, Jongeneel CV, Bucher P (1999). ESTScan: a program for detecting, evaluating, and reconstructing potential coding regions in EST sequences. Proc Int Conf Intell Syst Mol Biol.

[51] Peralta IE, Spooner DM (2001). Granule-bound starch synthase (GBSSI) gene phylogeny of wild tomatoes (*Solanum* L. section *Lycopersicon* [Mill.] Wettst. subsection *Lycopersicon*). Am J Bot.

[52] Martins TR, Barkman TJ, Smith JF (2005). Reconstruction of Solanaceae phylogeny using the nuclear gene SAMT. Syst Bot.

[53] Tamura K, Stecher G, Peterson D, Filipski A, Kumar S (2013). MEGA6: Molecular Evolutionary Genetics Analysis version 6.0.. Mol Biol Evol.

[54] Wang Y, Diehl A, Wu F, Vrebalov J, Giovannoni J, Siepel A, Tanksley SD (2008). Sequencing and comparative analysis of a conserved syntenic segment in the Solanaceae. Genetics.

[55] Garrison E (2010). FreeBayes. Marth Lab.

[56] Collins DW, Jukes TH (1994). Rates of transition and transversion in coding sequences since the human-rodent divergence. Genomics.

[57] Xie F, Burklew CE, Yang Y, Liu M, Xiao P, Zhang B, Qiu D (2012). De novo sequencing and a comprehensive analysis of purple sweet potato (*Ipomoea batatas* L.) transcriptome. Planta.

[58] Mooers AØ, Holmes EC (2000). The evolution of base composition and phylogenetic inference. Trends Ecol Evol.

[59] Aoki K, Yano K, Suzuki A, Kawamura S, Sakurai N, Suda K, Kurabayashi A, Suzuki T, Tsugane T, Watanabe M, Ooga K, Torii M, Narita T, Shin-I T, Kohara Y, Yamamoto N, Takahashi H, Watanabe Y, Egusa M, Kodama M, Ichinose Y, Kikuchi M, Fukushima S, Okabe A, Arie T, Sato Y, Yazawa K, Satoh S, Omura T, Ezura H (2010). Large-scale analysis of full-length cDNAs from the tomato (*Solanum lycopersicum*) cultivar Micro-Tom, a reference system for the Solanaceae genomics. BMC Genomics.

[60] Crookshanks M, Emmersen J, Welinder KG, Nielsen KL (2001). The potato tuber transcriptome: analysis of 6077 expressed sequence tags. FEBS Lett.

[61] Kanehisa M, Goto S (2000). KEGG: kyoto encyclopedia of genes and genomes. Nucleic Acids Res.

[62] Lester RN, Hawkes JG, Lester RN, Nee M, Estrad N (1991). Evolutionary relationships of tomato, potato, pepino, and wild species of *Lycopersicon* and *Solanum*. Solanaceae III Taxonomy, Chem Evol Kew Linn Soc London.

[63] Butelli E, Titta L, Giorgio M, Mock H-P, Matros A, Peterek S, Schijlen EGWM, Hall RD, Bovy AG, Luo J, Martin C (2008). Enrichment of tomato fruit with health-promoting anthocyanins by expression of select transcription factors. Nat Biotech.

[64] Clé C, Hill LM, Niggeweg R, Martin CR, Guisez Y, Prinsen E, Jansen MAK (2008). Modulation of chlorogenic acid biosynthesis in *Solanum lycopersicum*; consequences for phenolic accumulation and UV-tolerance. Phytochemistry.

[65] Niggeweg R, Michael AJ, Martin C (2004). Engineering plants with increased levels of the antioxidant chlorogenic acid. Nat Biotechnol.

[66] Prohens J, Sánchez MC, Rodríguez-Burruezo A, Cámara M, Torija E, Nuez F (2005). Morphological and physico-chemical characteristics of fruits of pepino (*Solanum muricatum*), wild relatives (*S. caripense* and *S. tabanoense*) and interspecific hybrids. Implications in pepino breeding. Eur J Hortic Sci.

[67] Blanca J, Montero-Pau J, Sauvage C, Bauchet G, Illa E, D’iez MJ, Francis D, Causse M, van der Knaap E, Cañizares J (2015). Genomic variation in tomato, from wild ancestors to contemporary breeding accessions. BMC Genomics.

[68] Rong J, Lammers Y, Strasburg JL, Schidlo NS, Ariyurek Y, de Jong TJ, Klinkhamer PGL, Smulders MJM, Vrieling K (2014). New insights into domestication of carrot from root transcriptome analyses. BMC Genomics.

[69] Swanson-Wagner R, Briskine R, Schaefer R, Hufford MB, Ross-Ibarra J, Myers CL, Tiffin P, Springer NM (2012). Reshaping of the maize transcriptome by domestication. Proc Natl Acad Sci.

[70] Feng Z, Zhang B, Ding W, Liu X, Yang D-L, Wei P, Cao F, Zhu S, Zhang F, Mao Y (2013). Efficient genome editing in plants using a CRISPR/Cas system. Cell Res.

[71] Park T, Vleeshouwers V, Jacobsen E, Van Der Vossen E, Visser RGF (2009). Molecular breeding for resistance to *Phytophthora infestans* (Mont.) de Bary in potato (*Solanum tuberosum* L.): a perspective of cisgenesis. Plant Breed.

[72] Hedges SB, Dudley J, Kumar S (2006). TimeTree: a public knowledge-base of divergence times among organisms. Bioinformatics.

[73] Zhai L, Xu L, Wang Y, Cheng H, Chen Y, Gong Y, Liu L (2014). Novel and useful genic-SSR markers from de novo transcriptome sequencing of radish (*Raphanus sativus* L.). Mol Breed.

[74] Ahn Y-K, Tripathi S, Kim J-H, Cho Y-I, Lee H-E, Kim D-S, Woo J-G, Yoon M-K (2014). Microsatellite marker information from high-throughput next-generation sequence data of *Capsicum annuum* varieties Mandarin and Blackcluster. Sci Hortic.

[75] Metzgar D, Bytof J, Wills C (2000). Selection against frameshift mutations limits microsatellite expansion in coding DNA. Genome Res.

[76] Li Y, Korol AB, Fahima T, Beiles A, Nevo E (2002). Microsatellites: genomic distribution, putative functions and mutational mechanisms: a review. Mol Ecol.

[77] Varshney RK, Graner A, Sorrells ME (2005). Genic microsatellite markers in plants: features and applications. Trends Biotechnol.

[78] Anderson GJ (1975). The variation and evolution of selected species of *Solanum* section *Basarthrum*. Brittonia.

[79] Murray BG, Hammett KRW, Grigg FDW (1992). Seed set and breeding system in the pepino *Solanum muricatum* Ait., Solanaceae. Sci Hortic (Amsterdam).

[80] Perez-de-Castro AM, Vilanova S, Canizares J, Pascual L, Blanca JM, Diez MJ, Prohens J, Pico B (2012). Application of genomic tools in plant breeding. Curr Genomics.

[81] Ruiz JJ, Prohens J, Nuez F (1997). “Sweet Round” and “Sweet Long”: Two pepino cultivars for Mediterranean, climates. HortSci.

[82] FASTAQC [http://www.bioinformatics.babraham.ac.uk/projects/fastqc/]. Accessed 29 Apr 2016.

[83] Langmead B, Salzberg SL (2012). Fast gapped-read alignment with Bowtie 2. Nat Methods.

[84] Li B, Dewey CN (2011). RSEM: accurate transcript quantification from RNA-Seq data with or without a reference genome. BMC Bioinformatics.

[85] Blanca JM, Pascual L, Ziarsolo P, Nuez F, Cañizares J (2011). ngs_backbone: a pipeline for read cleaning, mapping and SNP calling using Next Generation Sequence. BMC Genomics.

[86] Conesa A, Gotz S (2008). Blast2GO: A comprehensive suite for functional analysis in plant genomics. Int J Plant Genomics.

[87] Lippman ZB, Cohen O, Alvarez JP, Abu-Abied M, Pekker I, Paran I, Eshed Y, Zamir D (2008). The making of a compound inflorescence in tomato and related nightshades. PLoS Biol.

[88] Zhang Y, Hu Z, Chu G, Huang C, Tian S, Zhao Z, Chen G (2014). Anthocyanin accumulation and molecular analysis of anthocyanin biosynthesis-associated genes in eggplant (*Solanum melongena* L.). J Agric Food Chem.

[89] Kohara A, Nakajima C, Hashimoto K, Ikenaga T, Tanaka H, Shoyama Y, Yoshida S, Muranaka T (2005). A novel glucosyltransferase involved in steroid saponin biosynthesis in *Solanum aculeatissimum*. Plant Mol Biol.

[90] Gramazio P, Prohens J, Plazas M, Andujar I, Herraiz FJ, Castillo E, Knapp S, Meyer RS, Vilanova S (2014). Location of chlorogenic acid biosynthesis pathway and polyphenol oxidase genes in a new interspecific anchored linkage map of eggplant. BMC Plant Biol.

[91] Klann E, Yelle S, Bennett AB (1992). Tomato fruit Acid invertase complementary DNA: nucleotide and deduced amino Acid sequences. Plant Physiol.

[92] Lam Cheng KL (2013). Golden2--like (GLK2) Transcription Factor: Developmental Control of Tomato Fruit Photosynthesis and Its Contribution to Ripe Fruit Characteristics.

[93] Mott R (1997). EST_GENOME: A program to align spliced DNA sequences to unspliced genomic DNA. Comput Appl Biosci.

[94] EMBOSS [http://www.bioinformatics.nl/emboss-explorer/]. Accessed 29 Apr 2016.

[95] Krzywinski M, Schein J, Birol I, Connors J, Gascoyne R, Horsman D, Jones SJ, Marra MA (2009). Circos: an information aesthetic for comparative genomics. Genome Res.

[96] Larkin MA, Blackshields G, Brown NP, Chenna R, McGettigan PA, McWilliam H, Valentin F, Wallace IM, Wilm A, Lopez R, Thompson JD, Gibson TJ, Higgins DG (2007). Clustal W and Clustal X version 2.0.. Bioinformatics.

[97] Abajian C. Sputnik. University of Washington Department of Molecular Biotechnology. 1994.[http://wheat.pw.usda.gov/ITMI/EST-SSR/LaRota]. Accessed 29 Apr 2016.

