# Supplementary figures and images for: The first de novo transcriptome of pepino (Solanum muricatum): assembly, comprehensive analysis and comparison with the closely related species S. caripense, potato and tomato (part 1 of 2)
Source: BMC Genomics. 2016 May 4;17:321. doi: 10.1186/s12864-016-2656-8 (PMC4855764; doi:10.1186/s12864-016-2656-8)

| 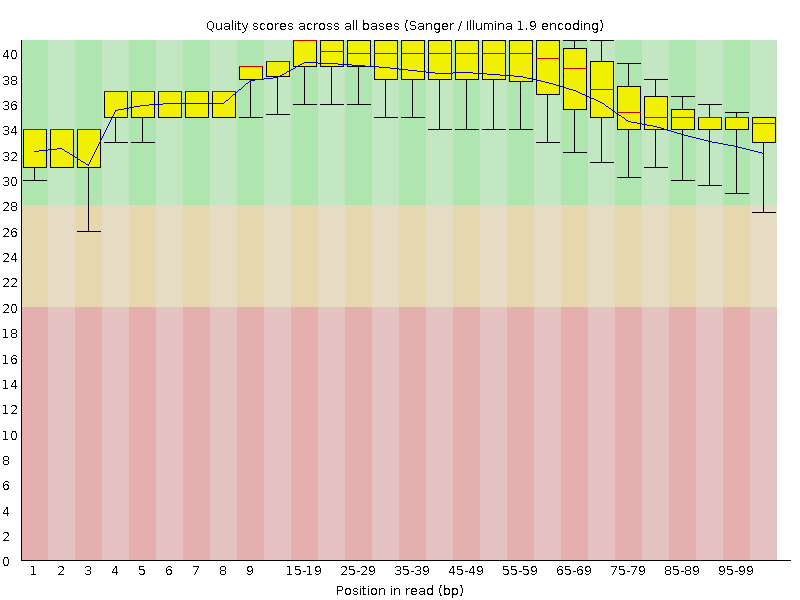 | 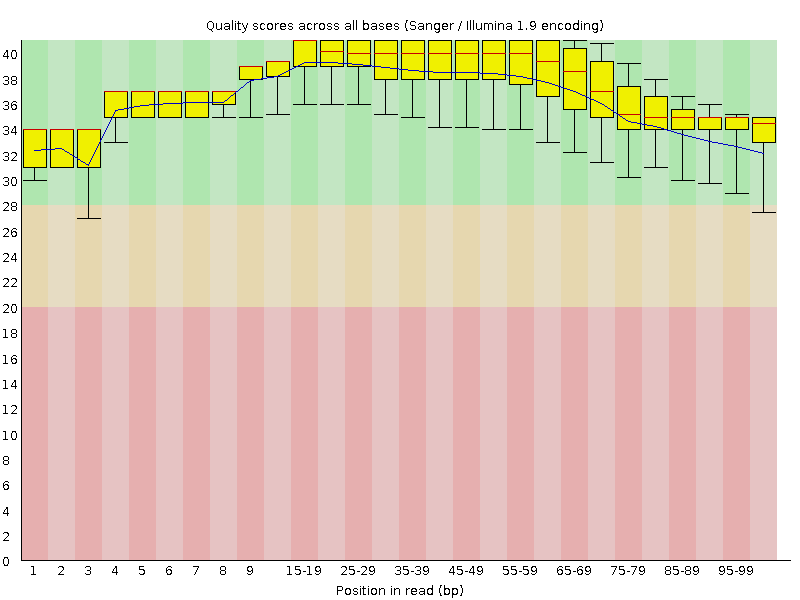 |
| --- | --- |

Supplement: Additional file 1: Figure S1. — Boxplots indicating the quality scores across all bases in S. muricatum (left) and in S. caripense (right) (Word file). Horizontal axis represents the base position in base pair. Vertical axis represents quality score (Q). (DOCX 32 kb) [file 12864_2016_2656_MOESM1_ESM.docx]

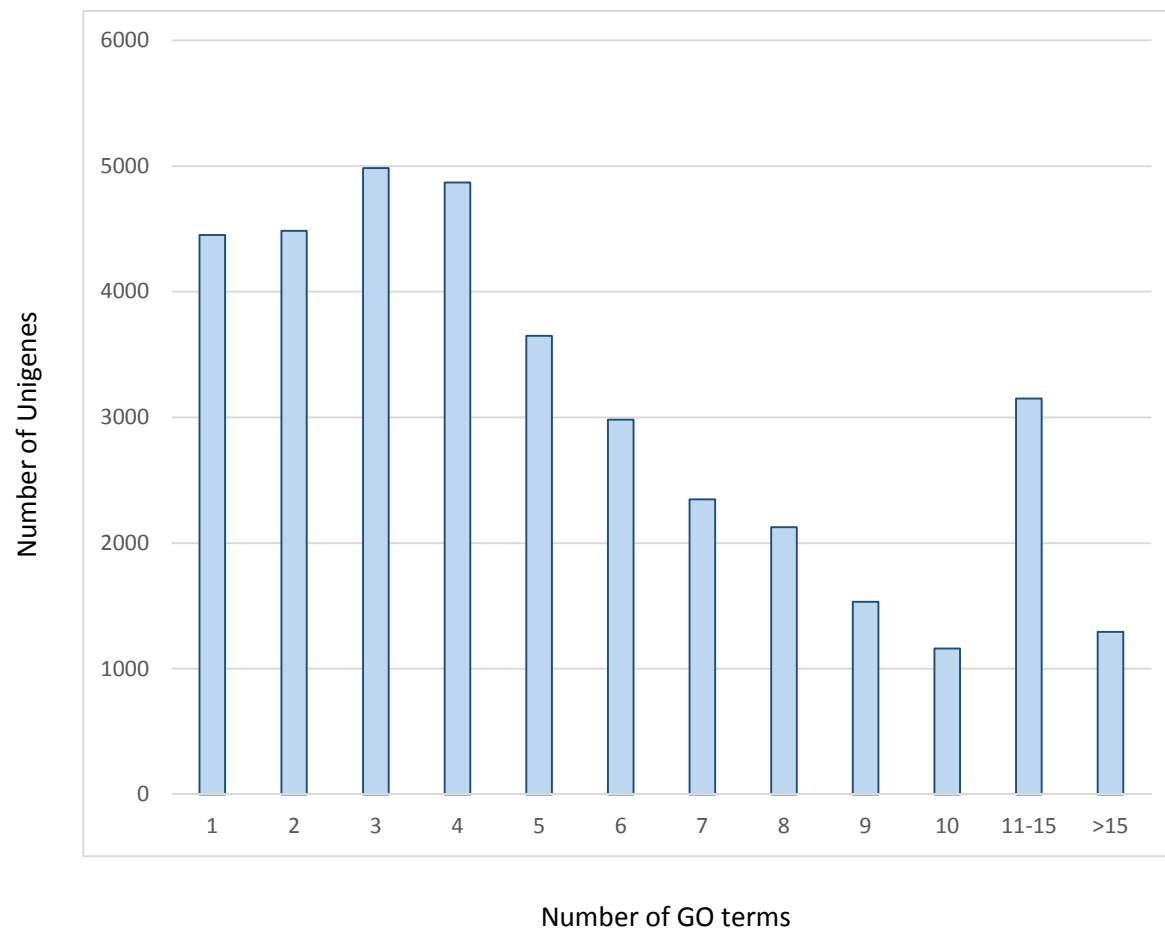

Supplement: Additional file 5: Figure S2. — Distribution of GO terms (pdf image). The unigenes distribution regarding the quantity of GO terms to which they are assigned. (PDF 17 kb) [file 12864_2016_2656_MOESM5_ESM.pdf]

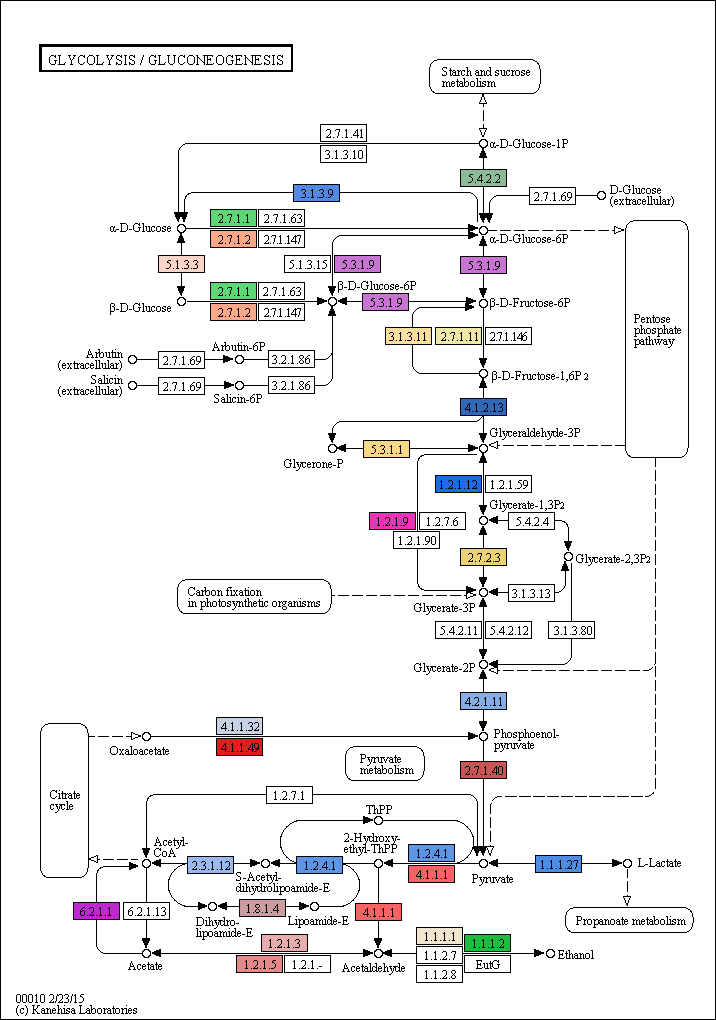

Supplement: Additional file 7: — KEGG pathway annotation. A zip compressed file with a list of KEGGs pathways, graphics in png format, and a file with a comparison with KEGGs pathways of potato and tomato. (ZIP 4361 kb) [file 12864_2016_2656_MOESM7_ESM.zip › Pathway representations/map00010_20150305161352.png]

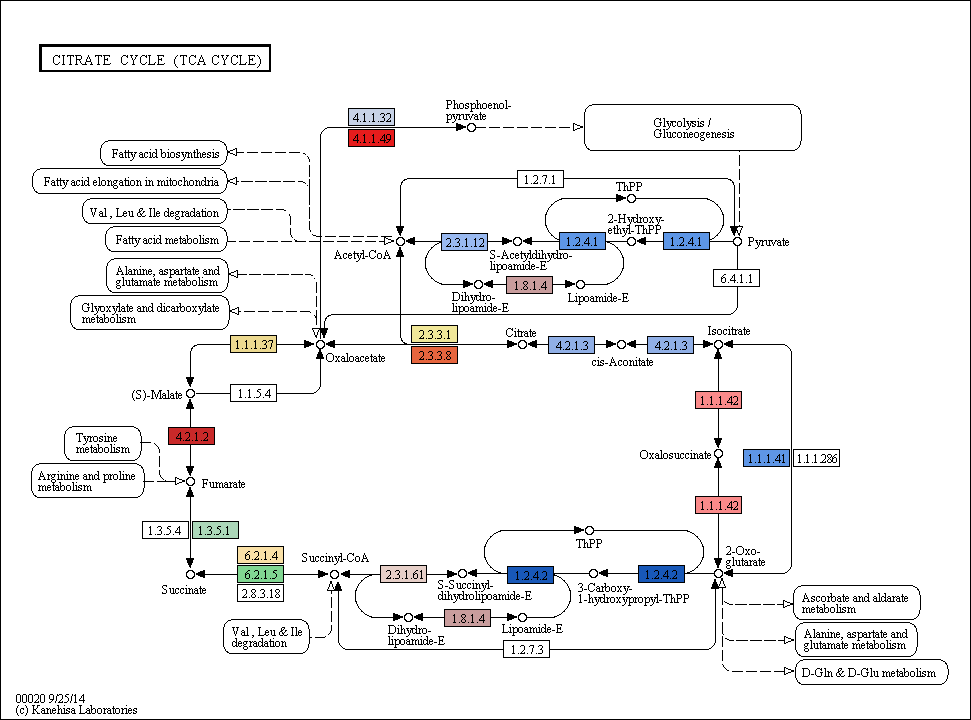

Supplement: Additional file 7: — KEGG pathway annotation. A zip compressed file with a list of KEGGs pathways, graphics in png format, and a file with a comparison with KEGGs pathways of potato and tomato. (ZIP 4361 kb) [file 12864_2016_2656_MOESM7_ESM.zip › Pathway representations/map00020_20150305161317.png]

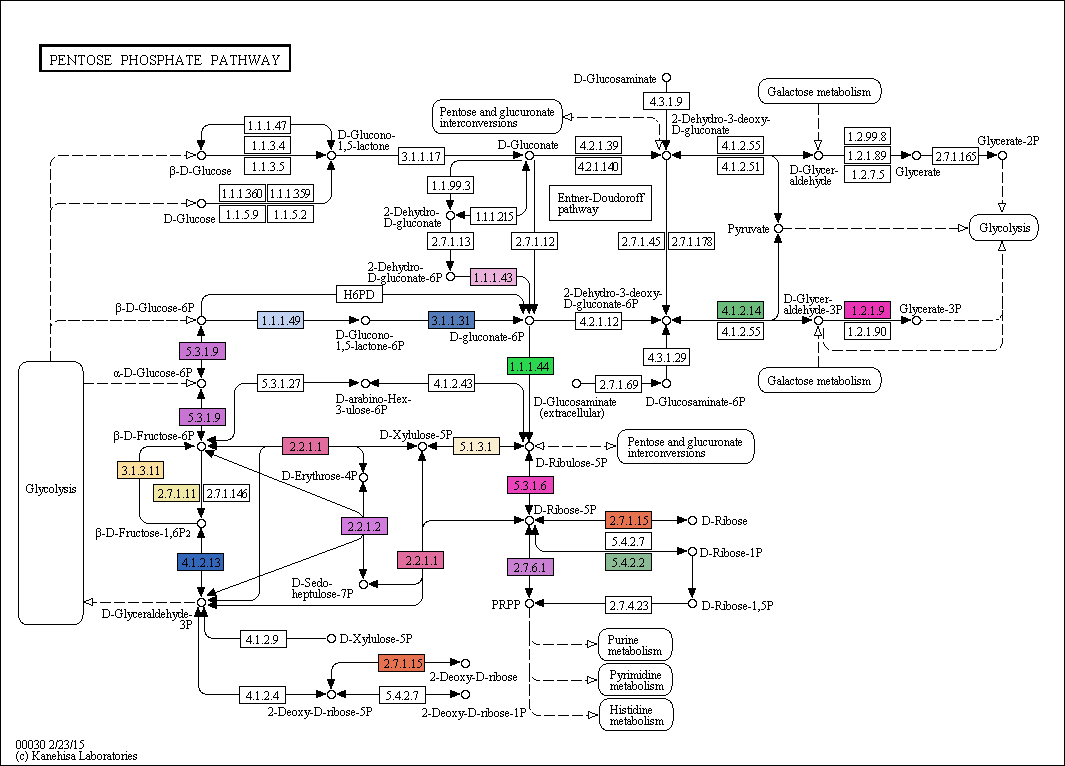

Supplement: Additional file 7: — KEGG pathway annotation. A zip compressed file with a list of KEGGs pathways, graphics in png format, and a file with a comparison with KEGGs pathways of potato and tomato. (ZIP 4361 kb) [file 12864_2016_2656_MOESM7_ESM.zip › Pathway representations/map00030_20150305161152.png]

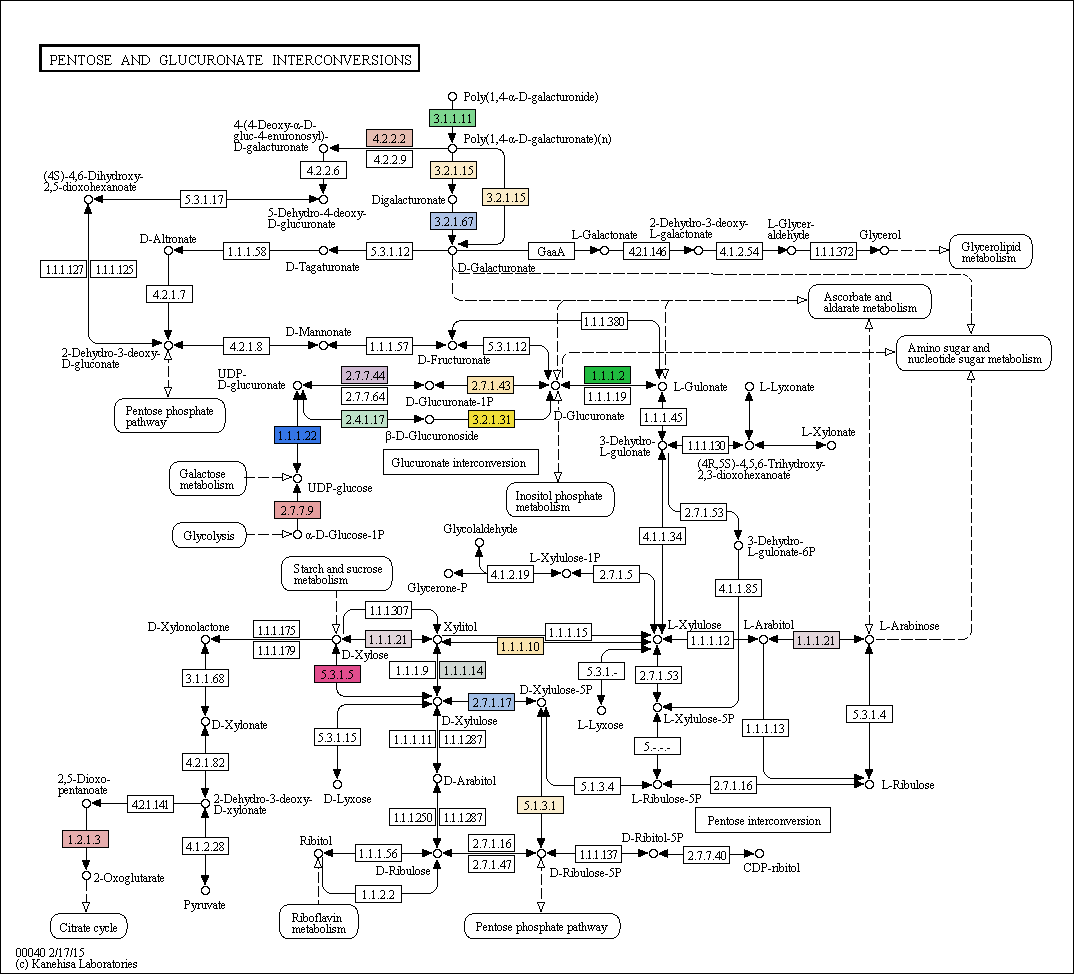

Supplement: Additional file 7: — KEGG pathway annotation. A zip compressed file with a list of KEGGs pathways, graphics in png format, and a file with a comparison with KEGGs pathways of potato and tomato. (ZIP 4361 kb) [file 12864_2016_2656_MOESM7_ESM.zip › Pathway representations/map00040_20150305161044.png]

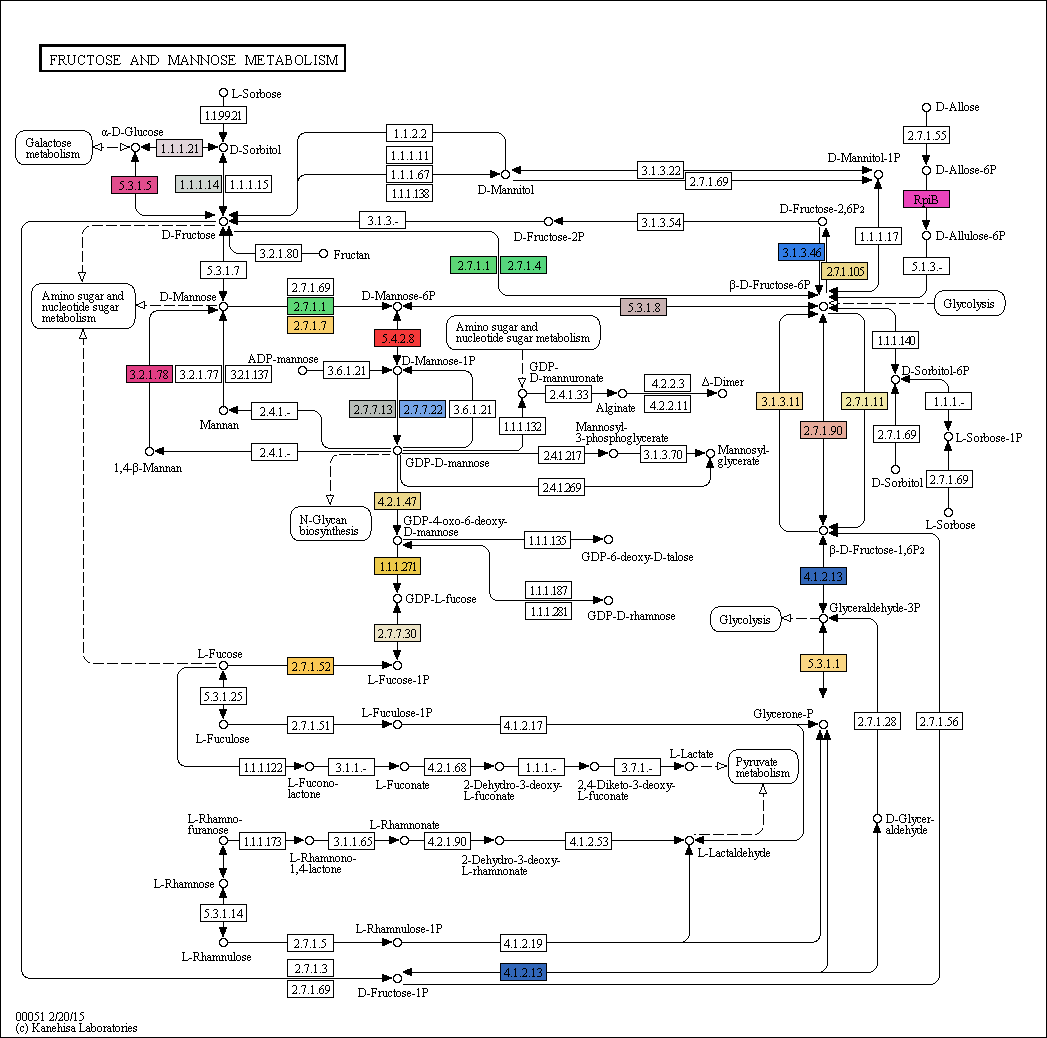

Supplement: Additional file 7: — KEGG pathway annotation. A zip compressed file with a list of KEGGs pathways, graphics in png format, and a file with a comparison with KEGGs pathways of potato and tomato. (ZIP 4361 kb) [file 12864_2016_2656_MOESM7_ESM.zip › Pathway representations/map00051_20150305161009.png]

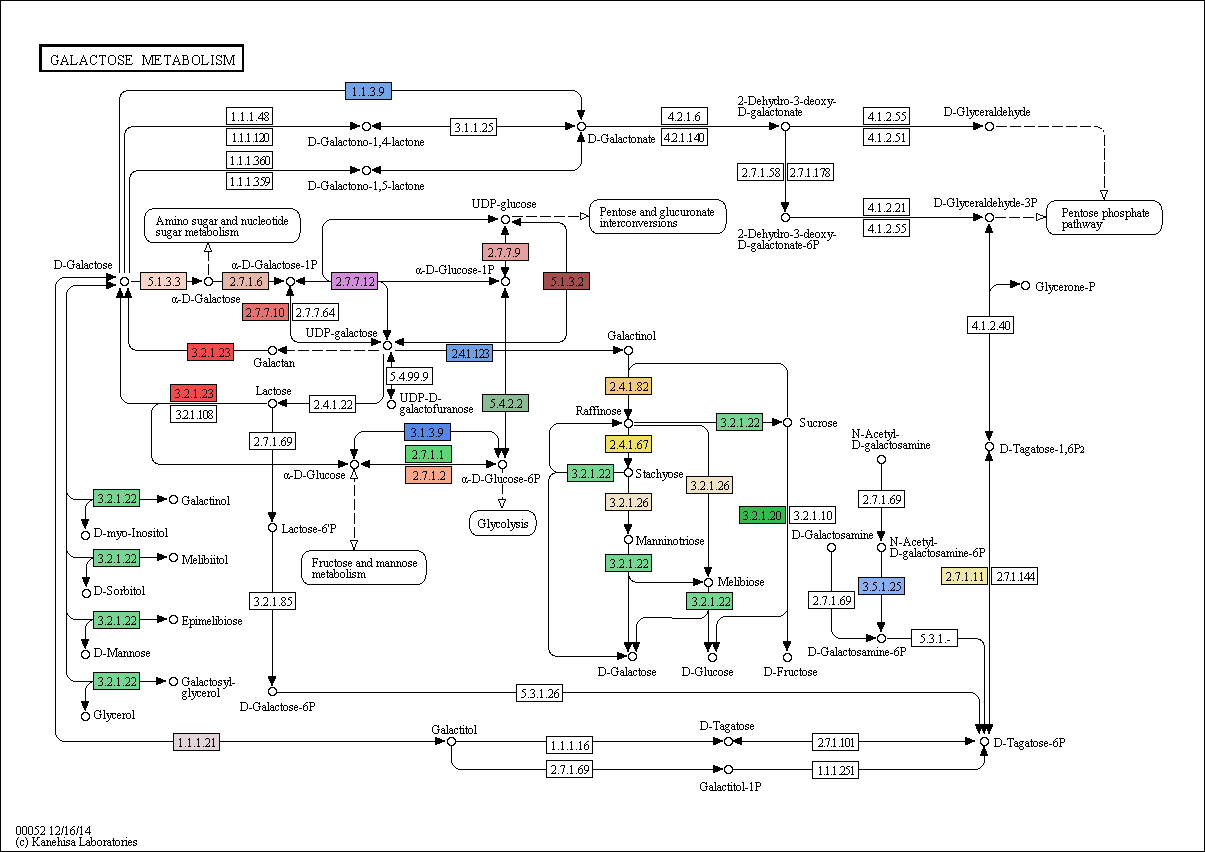

Supplement: Additional file 7: — KEGG pathway annotation. A zip compressed file with a list of KEGGs pathways, graphics in png format, and a file with a comparison with KEGGs pathways of potato and tomato. (ZIP 4361 kb) [file 12864_2016_2656_MOESM7_ESM.zip › Pathway representations/map00052_20150305161002.png]

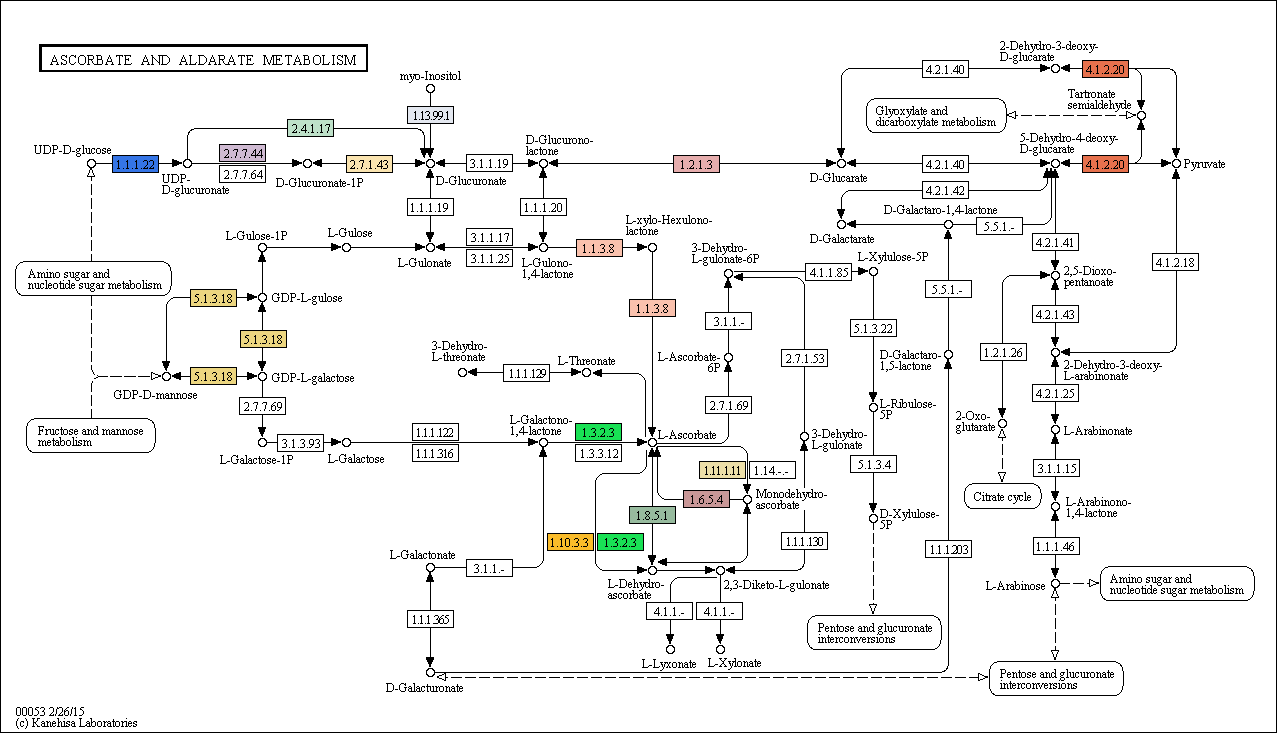

Supplement: Additional file 7: — KEGG pathway annotation. A zip compressed file with a list of KEGGs pathways, graphics in png format, and a file with a comparison with KEGGs pathways of potato and tomato. (ZIP 4361 kb) [file 12864_2016_2656_MOESM7_ESM.zip › Pathway representations/map00053_20150305160955.png]

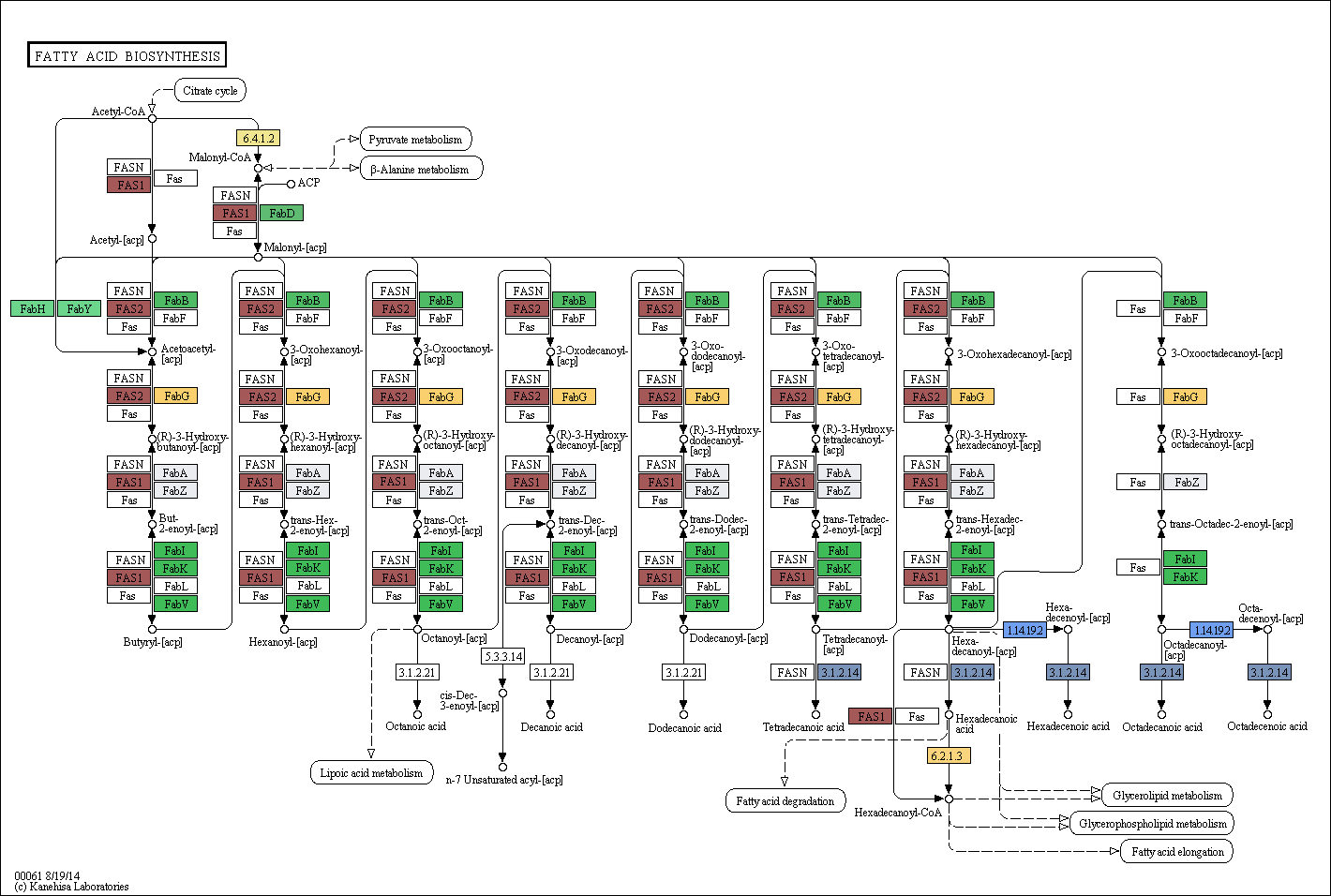

Supplement: Additional file 7: — KEGG pathway annotation. A zip compressed file with a list of KEGGs pathways, graphics in png format, and a file with a comparison with KEGGs pathways of potato and tomato. (ZIP 4361 kb) [file 12864_2016_2656_MOESM7_ESM.zip › Pathway representations/map00061_20150305160925.png]

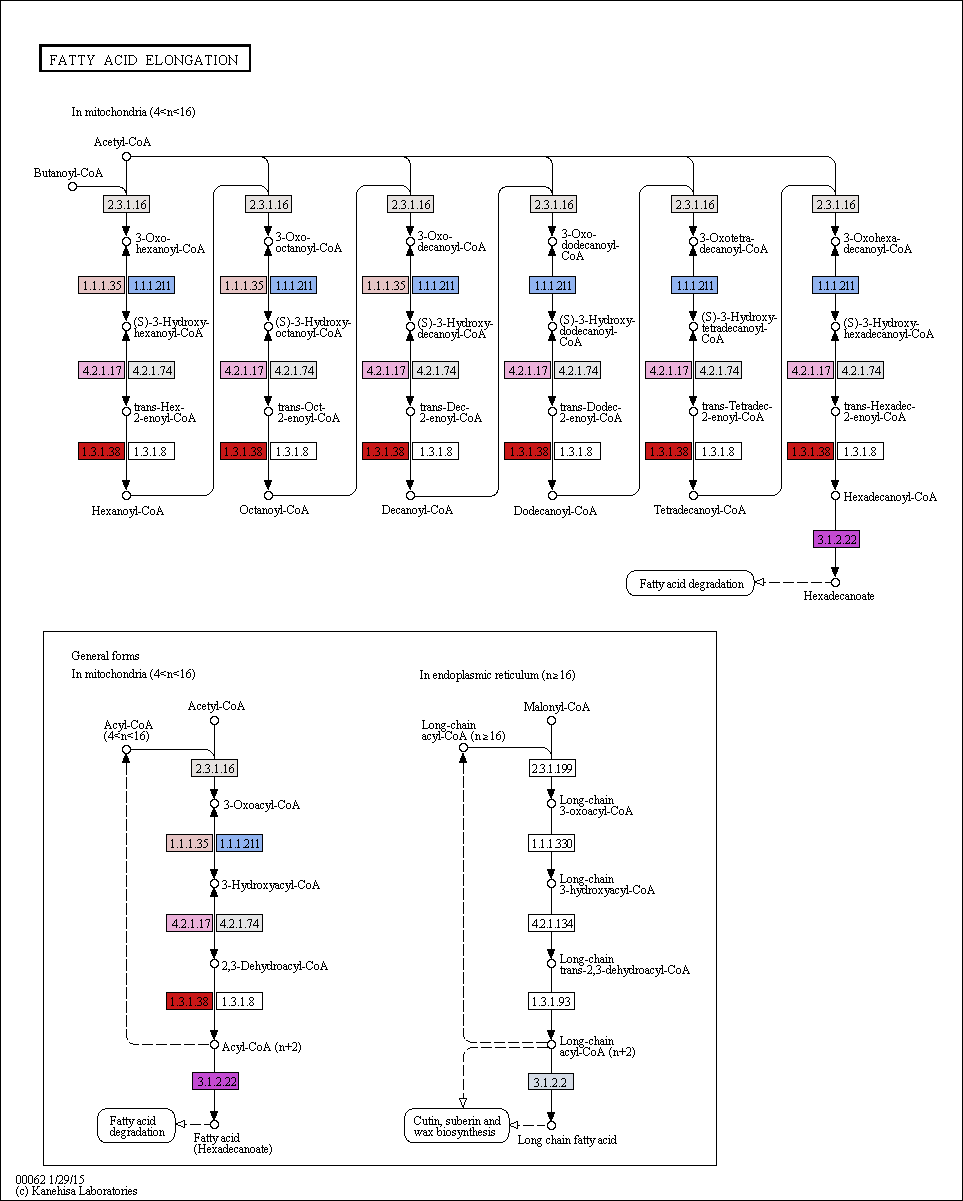

Supplement: Additional file 7: — KEGG pathway annotation. A zip compressed file with a list of KEGGs pathways, graphics in png format, and a file with a comparison with KEGGs pathways of potato and tomato. (ZIP 4361 kb) [file 12864_2016_2656_MOESM7_ESM.zip › Pathway representations/map00062_20150305160919.png]

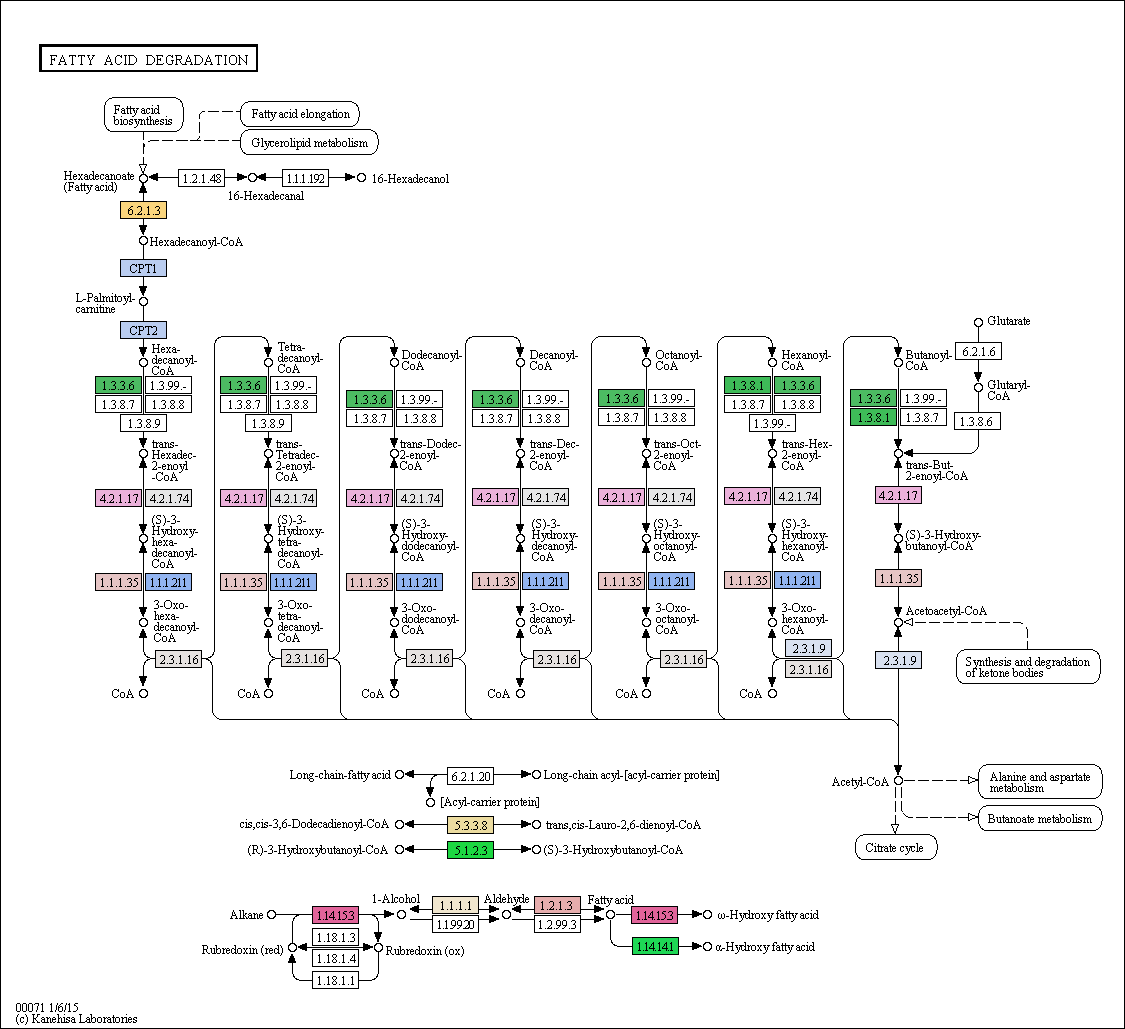

Supplement: Additional file 7: — KEGG pathway annotation. A zip compressed file with a list of KEGGs pathways, graphics in png format, and a file with a comparison with KEGGs pathways of potato and tomato. (ZIP 4361 kb) [file 12864_2016_2656_MOESM7_ESM.zip › Pathway representations/map00071_20150305160823.png]

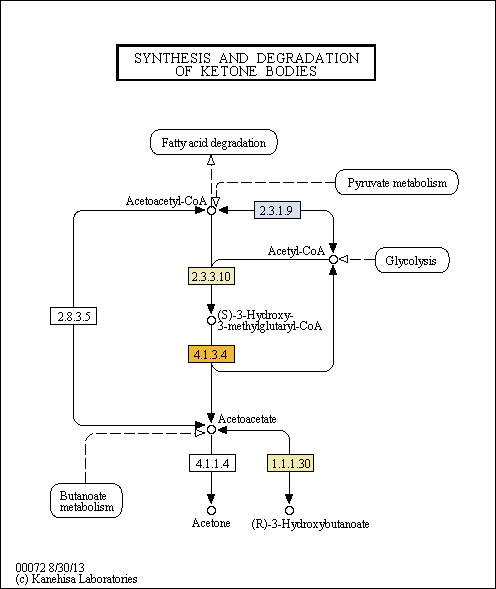

Supplement: Additional file 7: — KEGG pathway annotation. A zip compressed file with a list of KEGGs pathways, graphics in png format, and a file with a comparison with KEGGs pathways of potato and tomato. (ZIP 4361 kb) [file 12864_2016_2656_MOESM7_ESM.zip › Pathway representations/map00072_20150305160817.png]

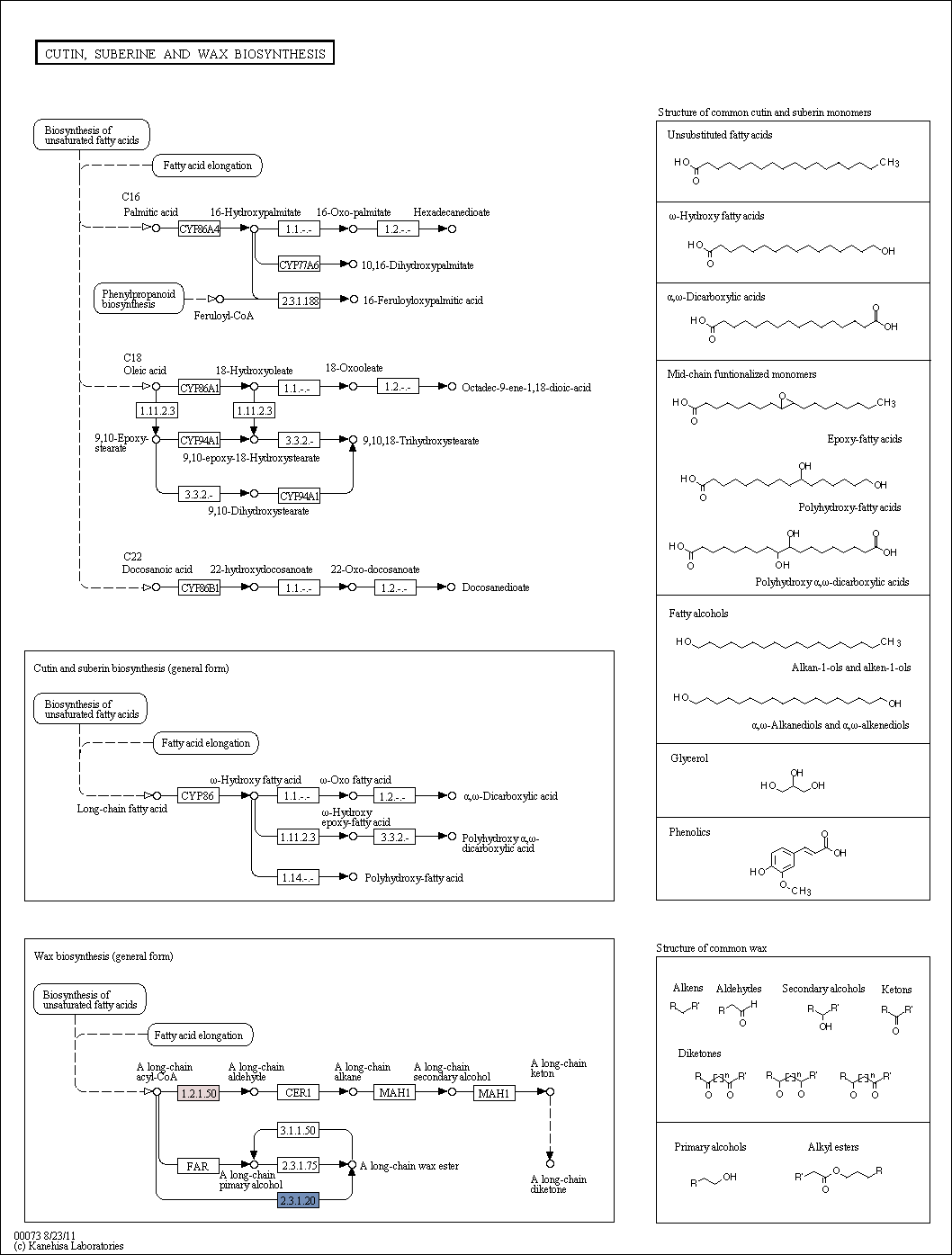

Supplement: Additional file 7: — KEGG pathway annotation. A zip compressed file with a list of KEGGs pathways, graphics in png format, and a file with a comparison with KEGGs pathways of potato and tomato. (ZIP 4361 kb) [file 12864_2016_2656_MOESM7_ESM.zip › Pathway representations/map00073_20150305160813.png]

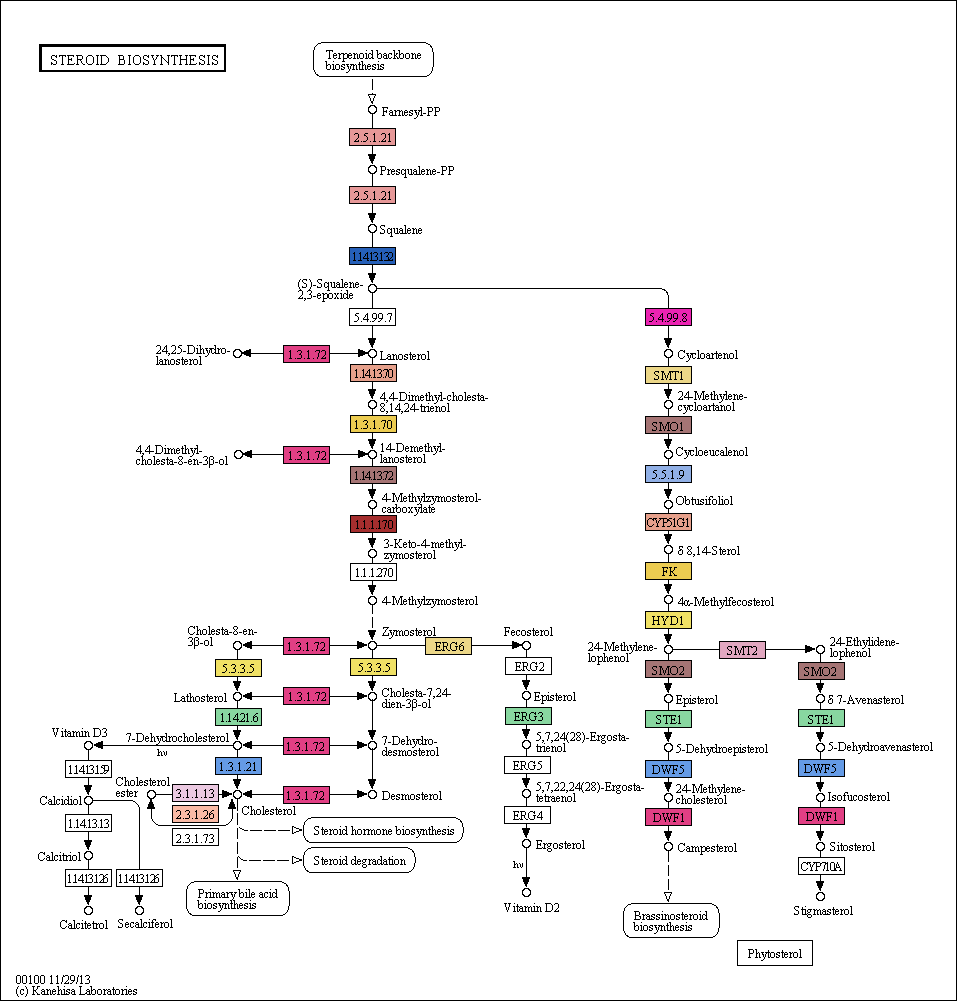

Supplement: Additional file 7: — KEGG pathway annotation. A zip compressed file with a list of KEGGs pathways, graphics in png format, and a file with a comparison with KEGGs pathways of potato and tomato. (ZIP 4361 kb) [file 12864_2016_2656_MOESM7_ESM.zip › Pathway representations/map00100_20150305160905.png]

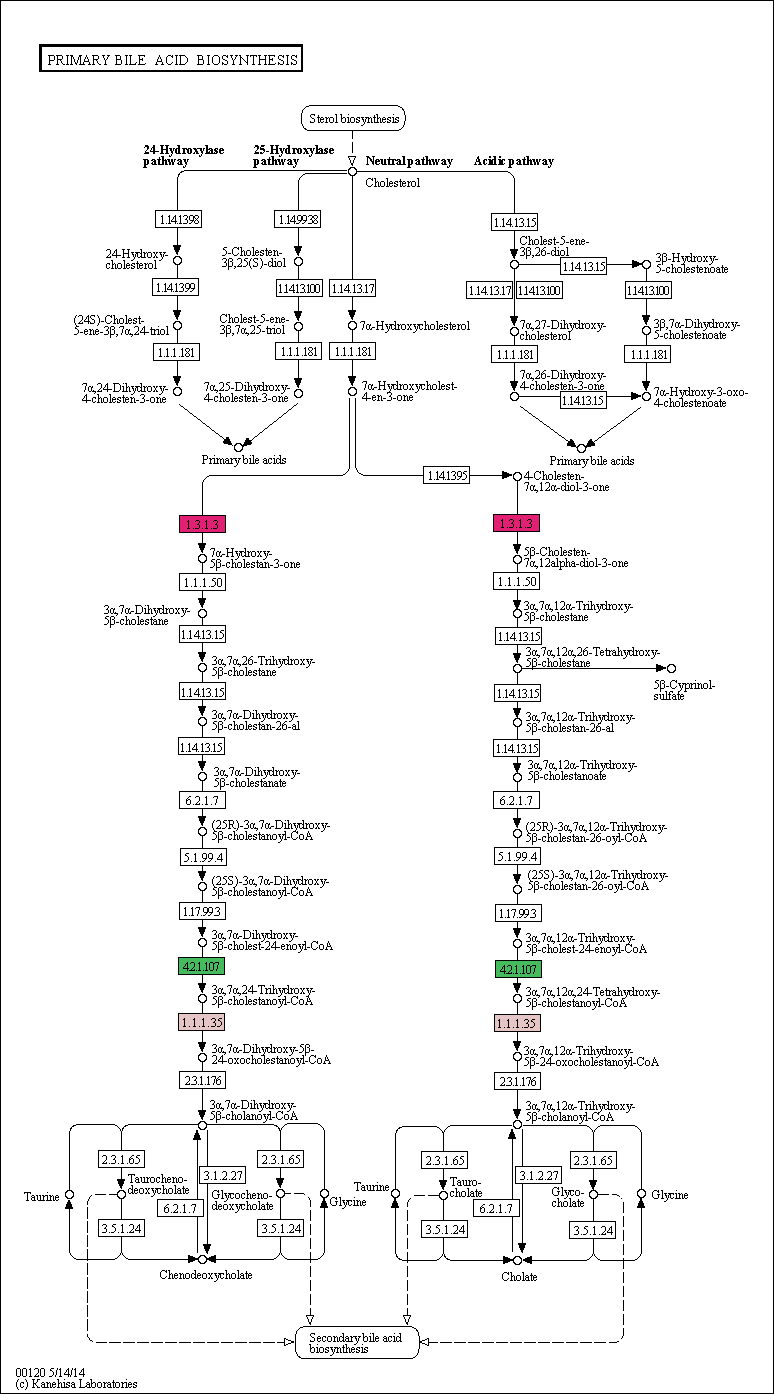

Supplement: Additional file 7: — KEGG pathway annotation. A zip compressed file with a list of KEGGs pathways, graphics in png format, and a file with a comparison with KEGGs pathways of potato and tomato. (ZIP 4361 kb) [file 12864_2016_2656_MOESM7_ESM.zip › Pathway representations/map00120_20150305160614.png]

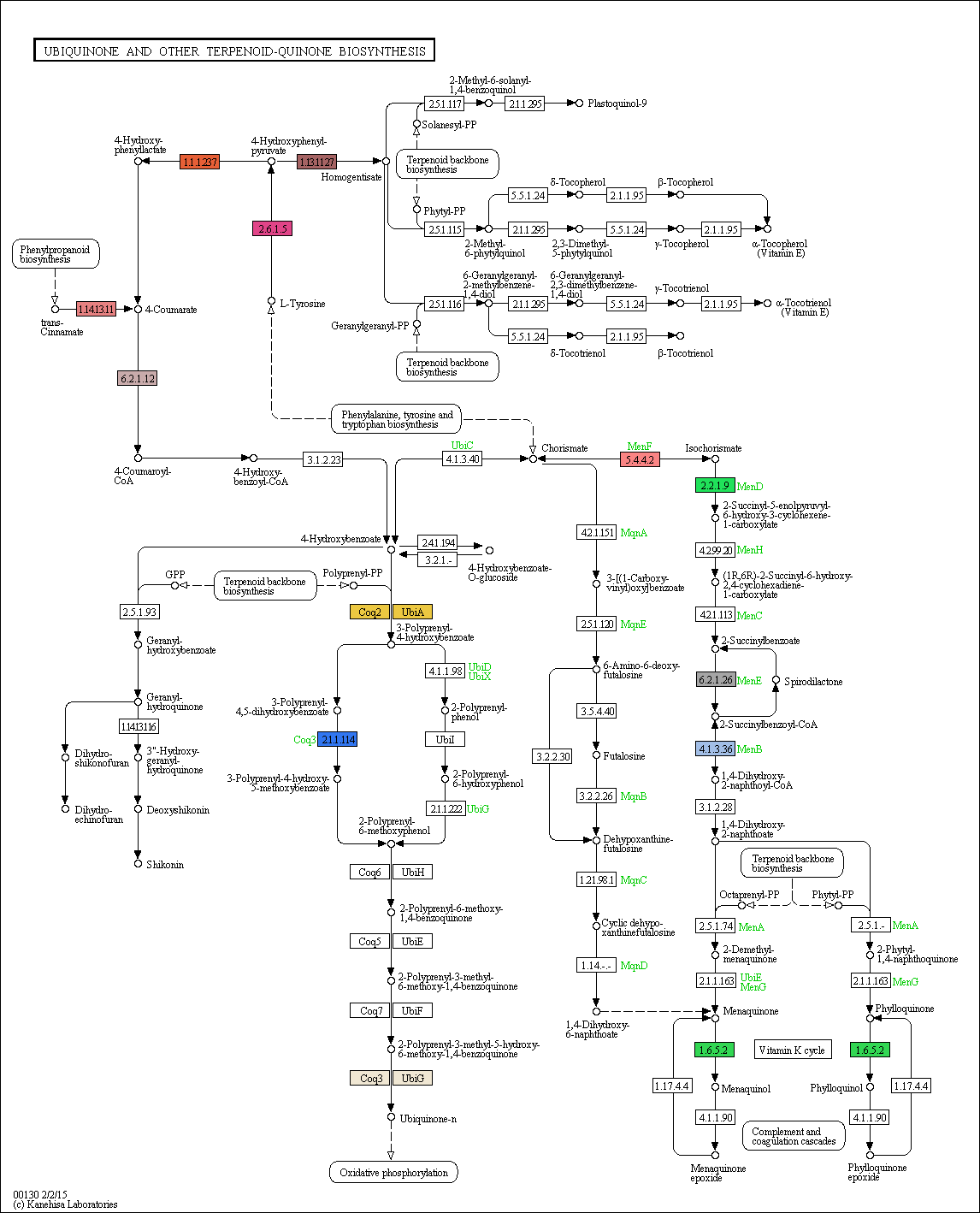

Supplement: Additional file 7: — KEGG pathway annotation. A zip compressed file with a list of KEGGs pathways, graphics in png format, and a file with a comparison with KEGGs pathways of potato and tomato. (ZIP 4361 kb) [file 12864_2016_2656_MOESM7_ESM.zip › Pathway representations/map00130_20150305160500.png]

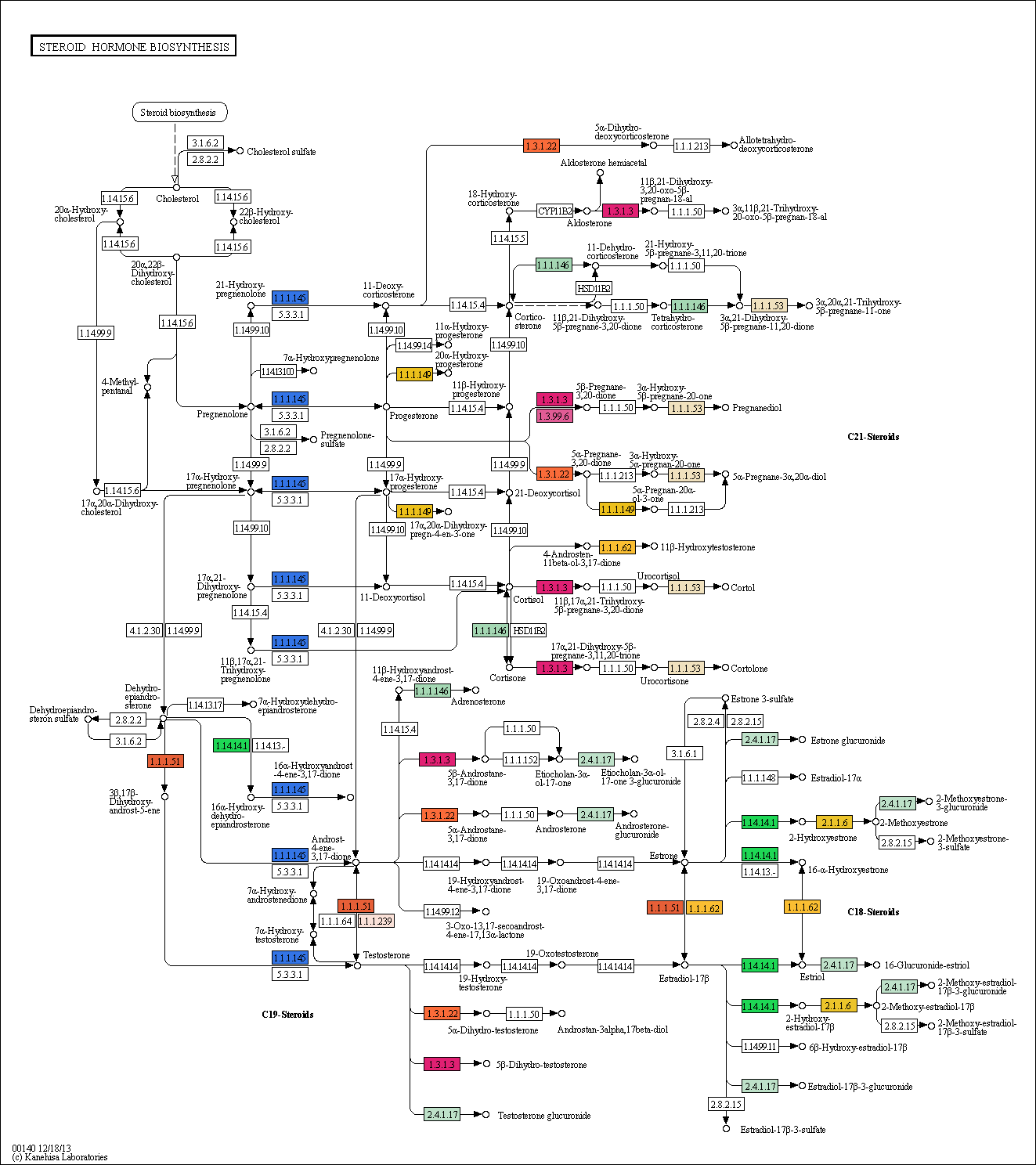

Supplement: Additional file 7: — KEGG pathway annotation. A zip compressed file with a list of KEGGs pathways, graphics in png format, and a file with a comparison with KEGGs pathways of potato and tomato. (ZIP 4361 kb) [file 12864_2016_2656_MOESM7_ESM.zip › Pathway representations/map00140_20150305160416.png]

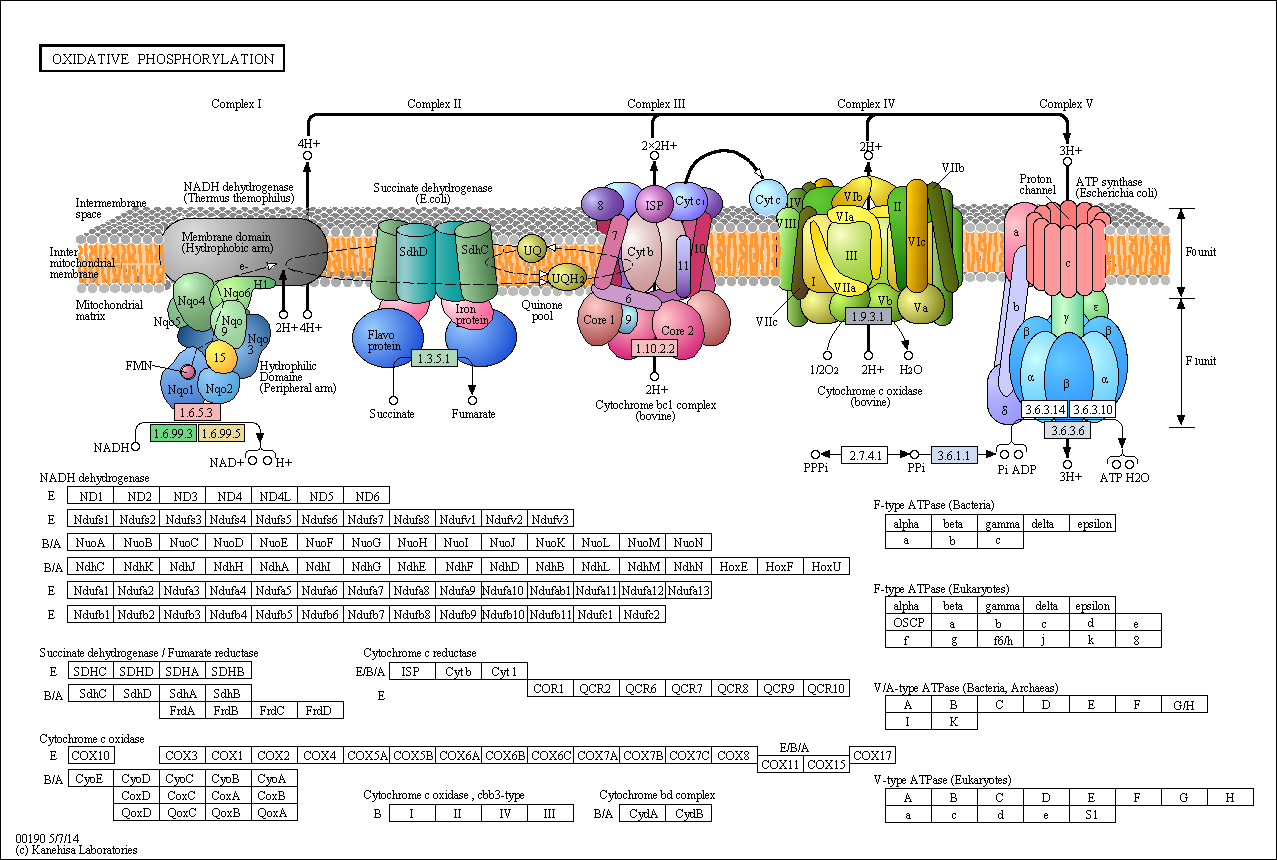

Supplement: Additional file 7: — KEGG pathway annotation. A zip compressed file with a list of KEGGs pathways, graphics in png format, and a file with a comparison with KEGGs pathways of potato and tomato. (ZIP 4361 kb) [file 12864_2016_2656_MOESM7_ESM.zip › Pathway representations/map00190_20150305161200.png]

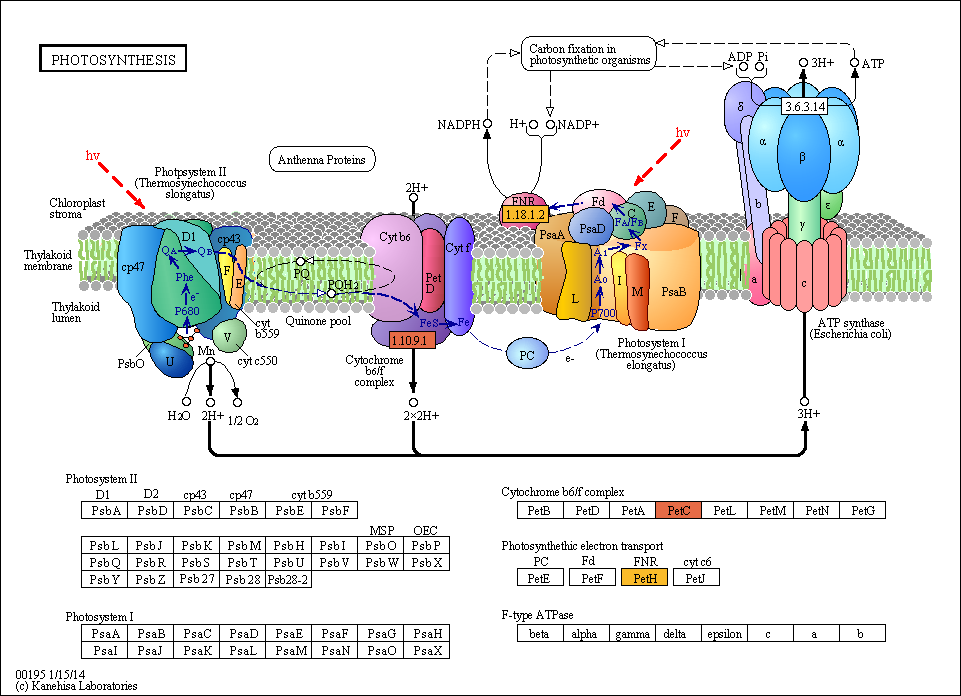

Supplement: Additional file 7: — KEGG pathway annotation. A zip compressed file with a list of KEGGs pathways, graphics in png format, and a file with a comparison with KEGGs pathways of potato and tomato. (ZIP 4361 kb) [file 12864_2016_2656_MOESM7_ESM.zip › Pathway representations/map00195_20150305161145.png]

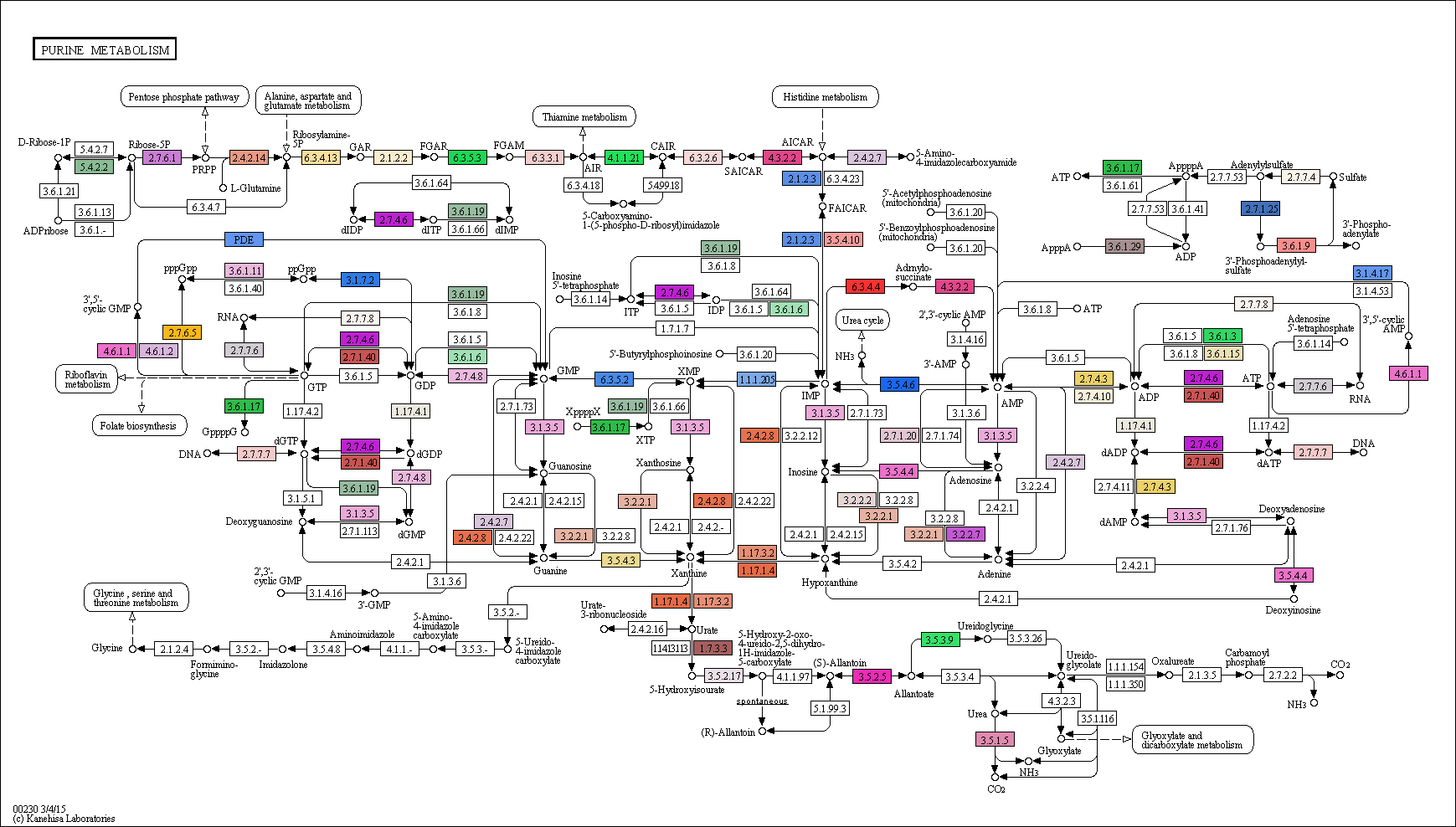

Supplement: Additional file 7: — KEGG pathway annotation. A zip compressed file with a list of KEGGs pathways, graphics in png format, and a file with a comparison with KEGGs pathways of potato and tomato. (ZIP 4361 kb) [file 12864_2016_2656_MOESM7_ESM.zip › Pathway representations/map00230_20150305161143.png]

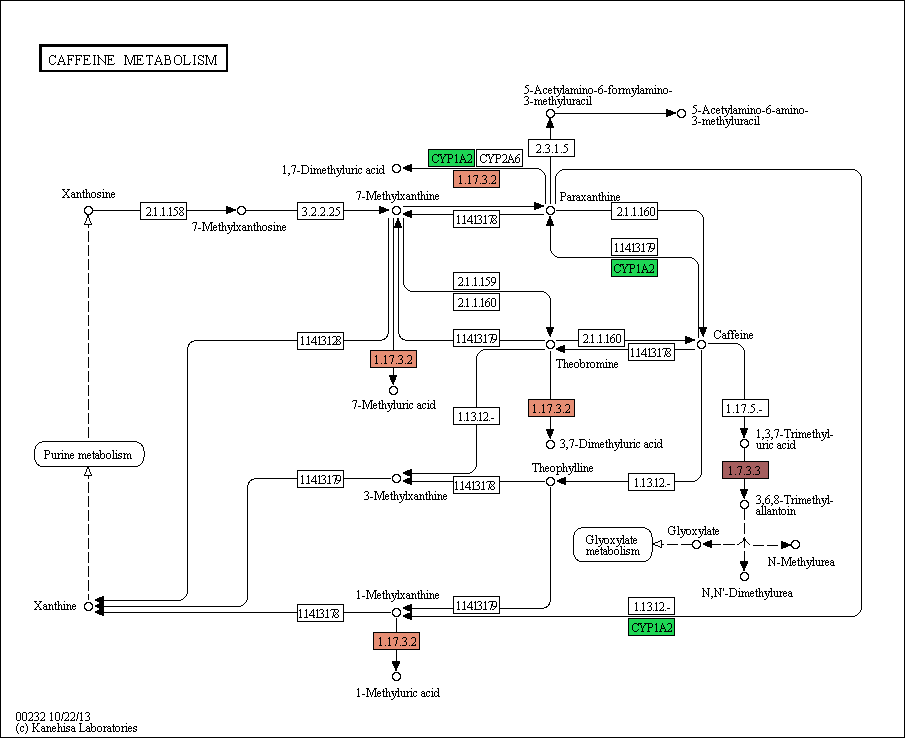

Supplement: Additional file 7: — KEGG pathway annotation. A zip compressed file with a list of KEGGs pathways, graphics in png format, and a file with a comparison with KEGGs pathways of potato and tomato. (ZIP 4361 kb) [file 12864_2016_2656_MOESM7_ESM.zip › Pathway representations/map00232_20150305161127.png]

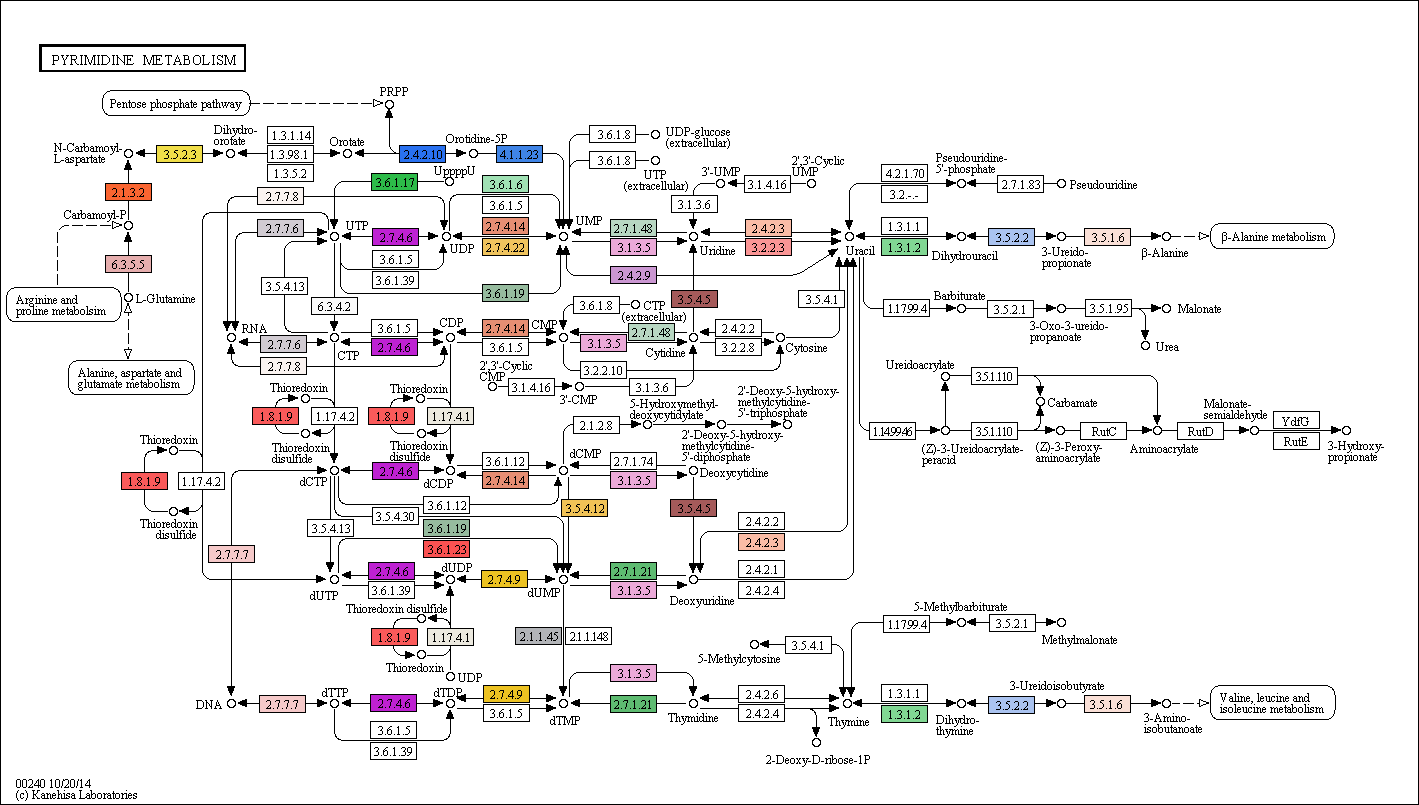

Supplement: Additional file 7: — KEGG pathway annotation. A zip compressed file with a list of KEGGs pathways, graphics in png format, and a file with a comparison with KEGGs pathways of potato and tomato. (ZIP 4361 kb) [file 12864_2016_2656_MOESM7_ESM.zip › Pathway representations/map00240_20150305161033.png]

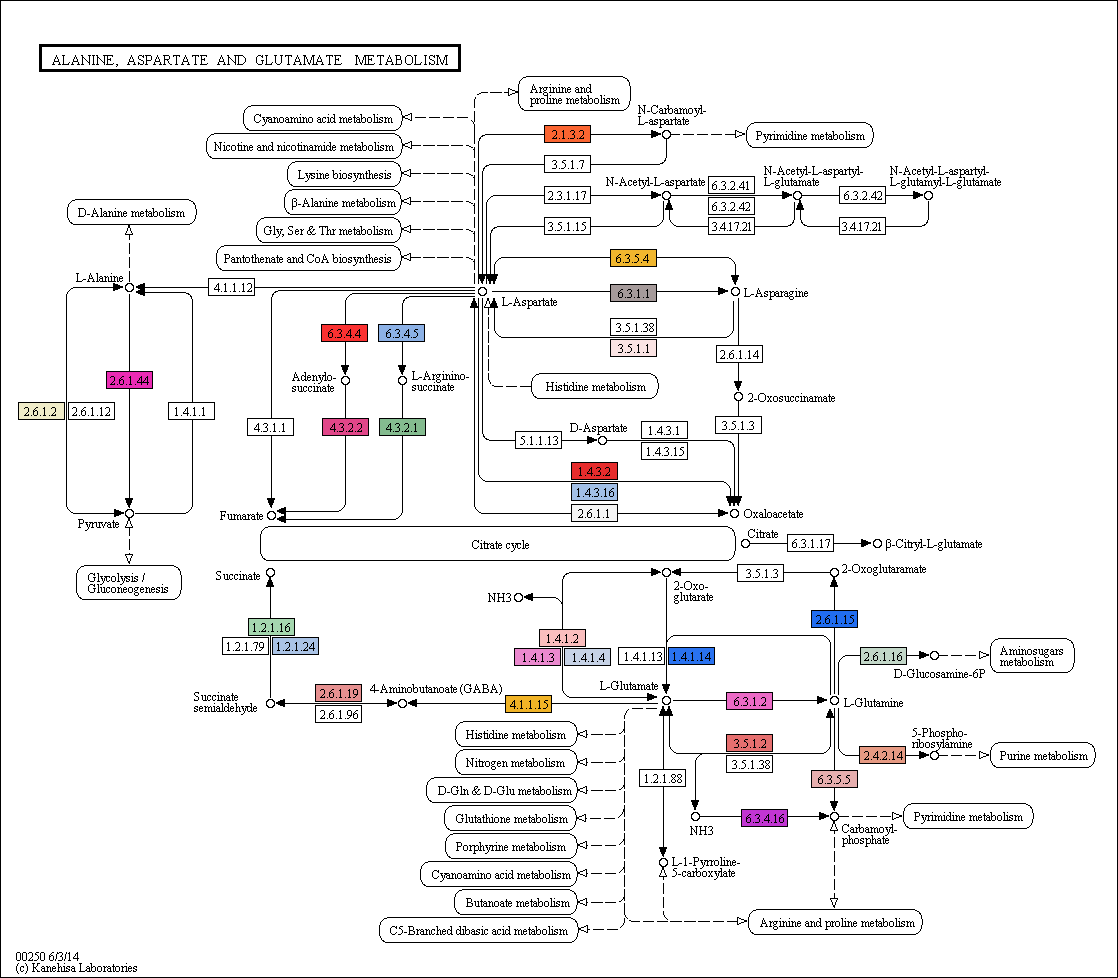

Supplement: Additional file 7: — KEGG pathway annotation. A zip compressed file with a list of KEGGs pathways, graphics in png format, and a file with a comparison with KEGGs pathways of potato and tomato. (ZIP 4361 kb) [file 12864_2016_2656_MOESM7_ESM.zip › Pathway representations/map00250_20150305160949.png]

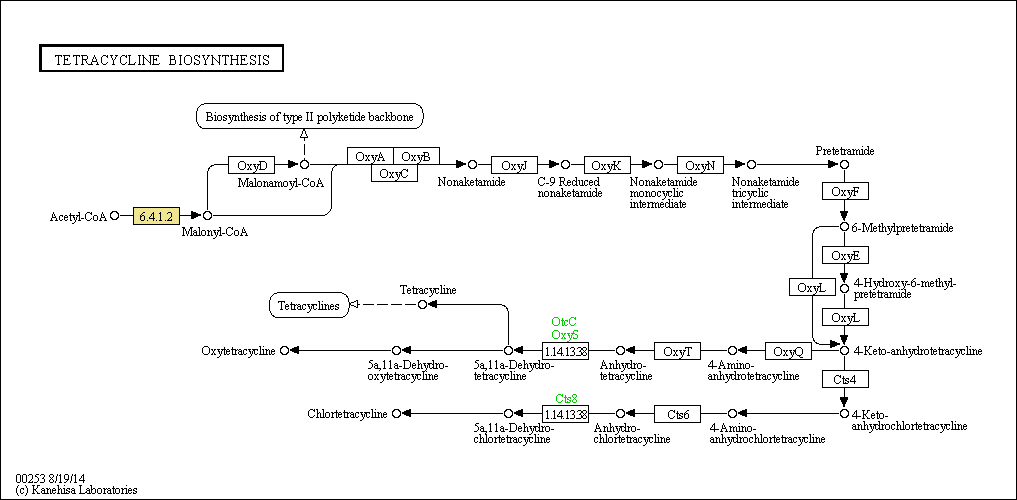

Supplement: Additional file 7: — KEGG pathway annotation. A zip compressed file with a list of KEGGs pathways, graphics in png format, and a file with a comparison with KEGGs pathways of potato and tomato. (ZIP 4361 kb) [file 12864_2016_2656_MOESM7_ESM.zip › Pathway representations/map00253_20150305160941.png]

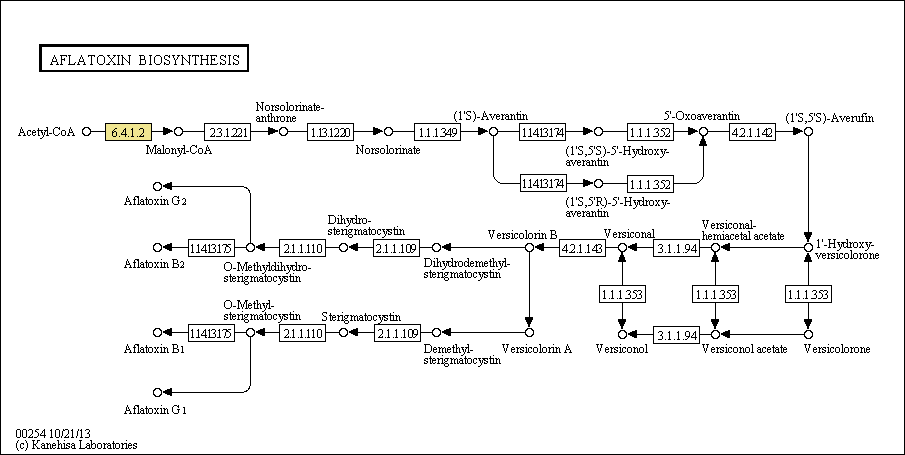

Supplement: Additional file 7: — KEGG pathway annotation. A zip compressed file with a list of KEGGs pathways, graphics in png format, and a file with a comparison with KEGGs pathways of potato and tomato. (ZIP 4361 kb) [file 12864_2016_2656_MOESM7_ESM.zip › Pathway representations/map00254_20150305160939.png]

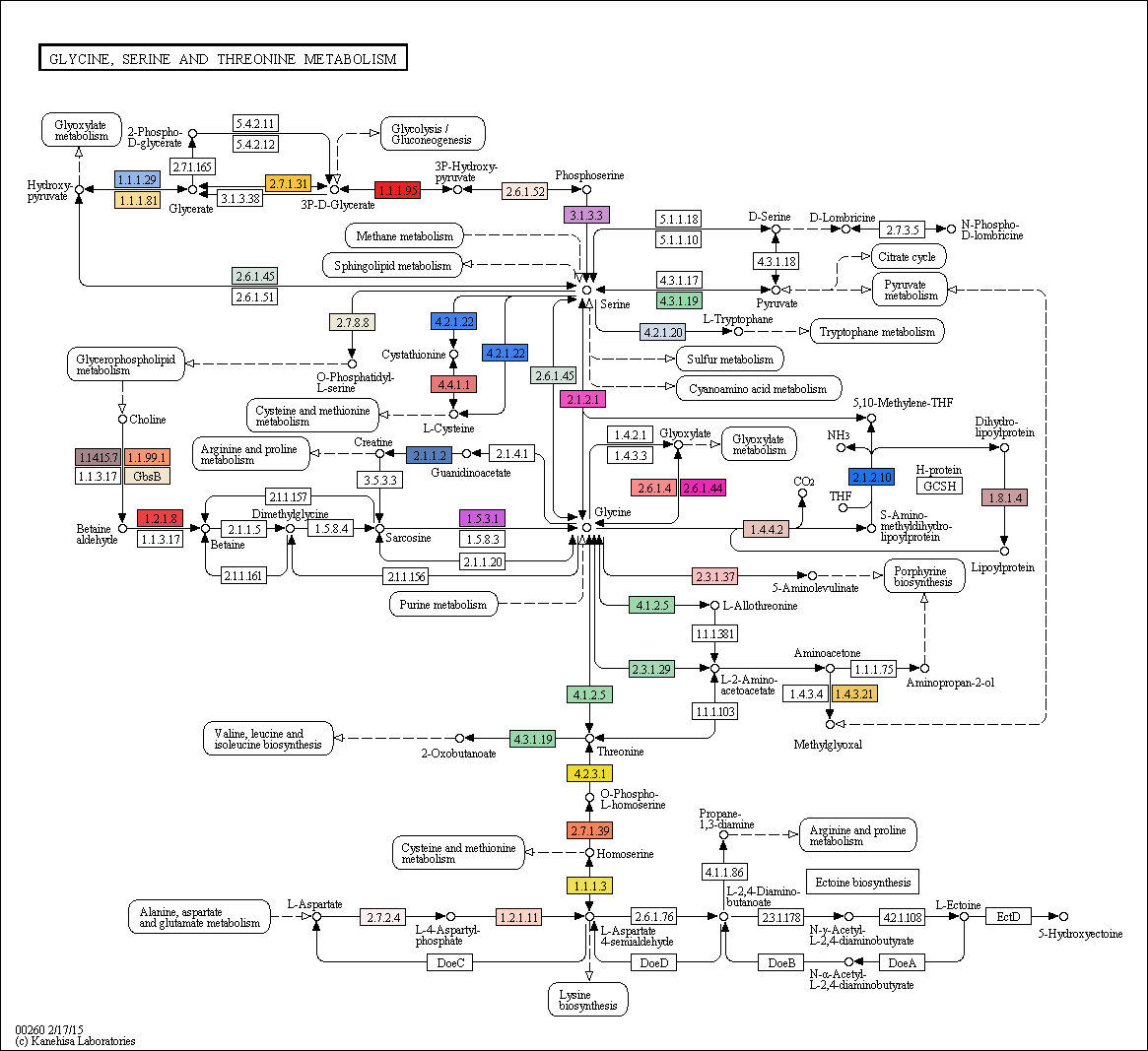

Supplement: Additional file 7: — KEGG pathway annotation. A zip compressed file with a list of KEGGs pathways, graphics in png format, and a file with a comparison with KEGGs pathways of potato and tomato. (ZIP 4361 kb) [file 12864_2016_2656_MOESM7_ESM.zip › Pathway representations/map00260_20150305160915.png]

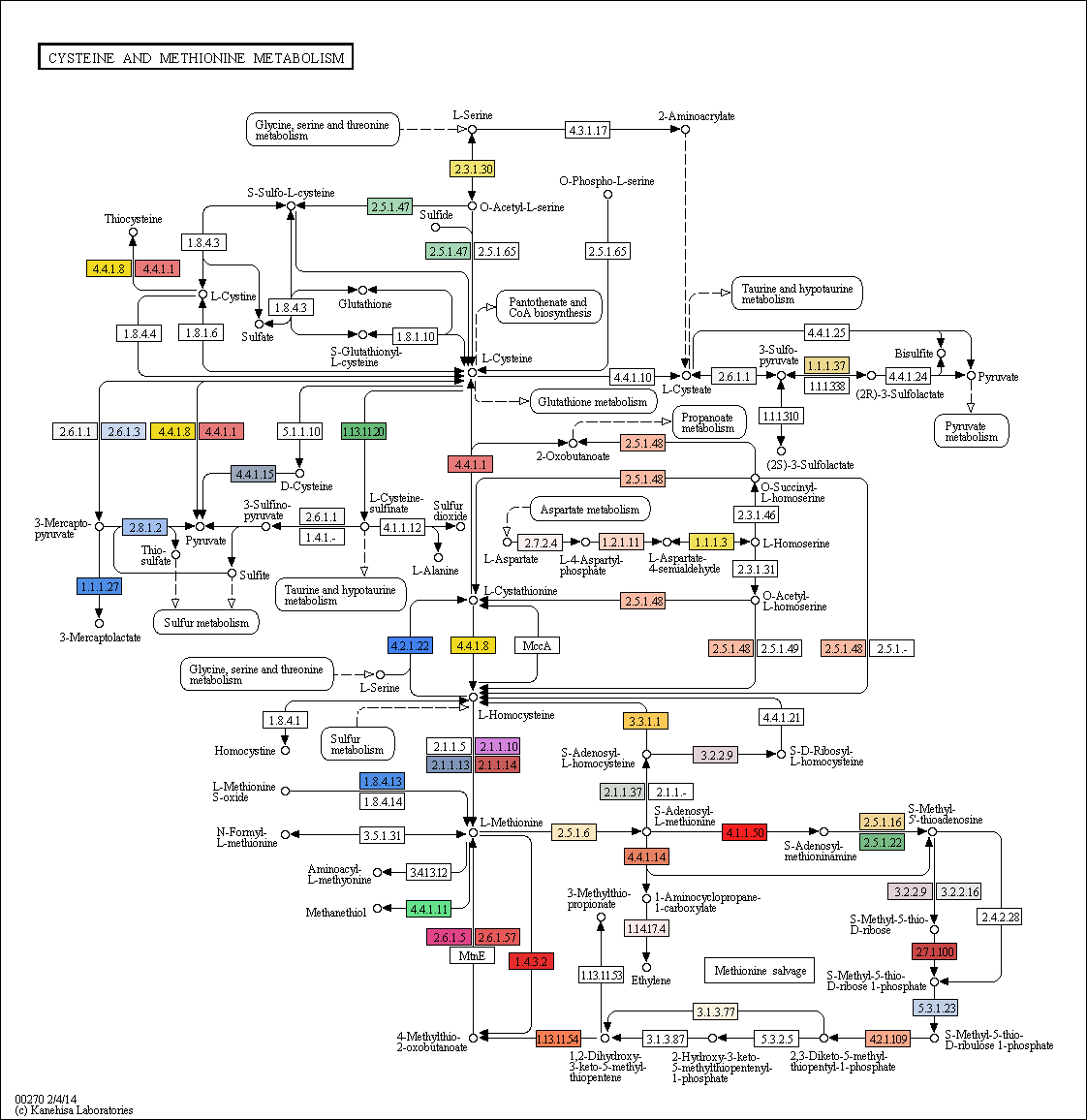

Supplement: Additional file 7: — KEGG pathway annotation. A zip compressed file with a list of KEGGs pathways, graphics in png format, and a file with a comparison with KEGGs pathways of potato and tomato. (ZIP 4361 kb) [file 12864_2016_2656_MOESM7_ESM.zip › Pathway representations/map00270_20150305160759.png]

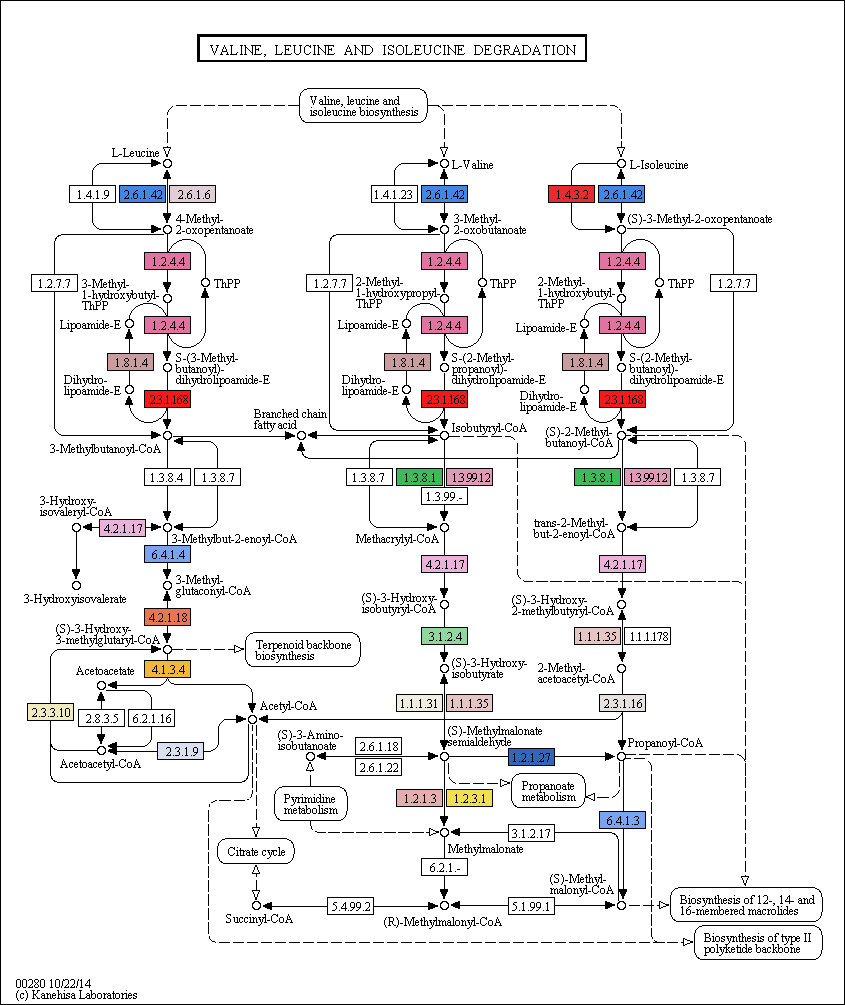

Supplement: Additional file 7: — KEGG pathway annotation. A zip compressed file with a list of KEGGs pathways, graphics in png format, and a file with a comparison with KEGGs pathways of potato and tomato. (ZIP 4361 kb) [file 12864_2016_2656_MOESM7_ESM.zip › Pathway representations/map00280_20150305160625.png]

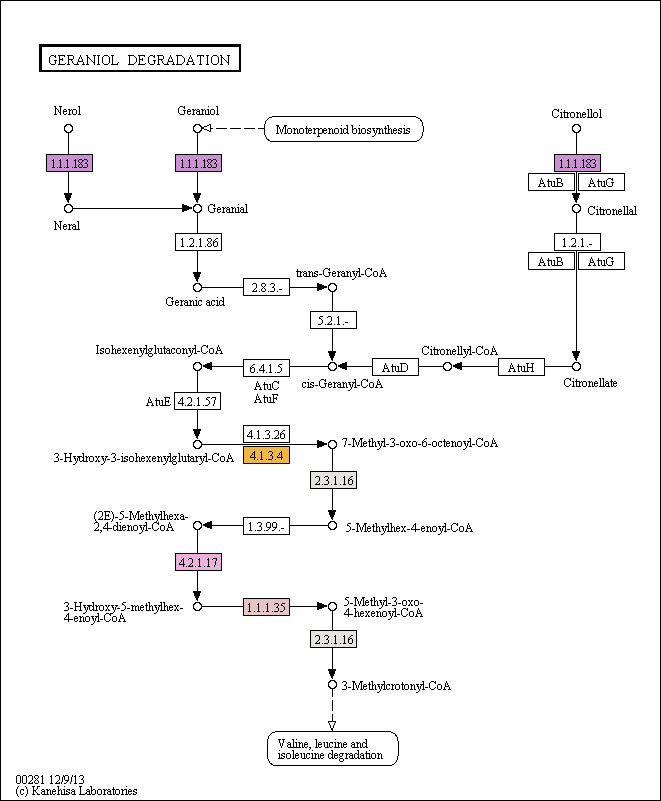

Supplement: Additional file 7: — KEGG pathway annotation. A zip compressed file with a list of KEGGs pathways, graphics in png format, and a file with a comparison with KEGGs pathways of potato and tomato. (ZIP 4361 kb) [file 12864_2016_2656_MOESM7_ESM.zip › Pathway representations/map00281_20150305160618.png]

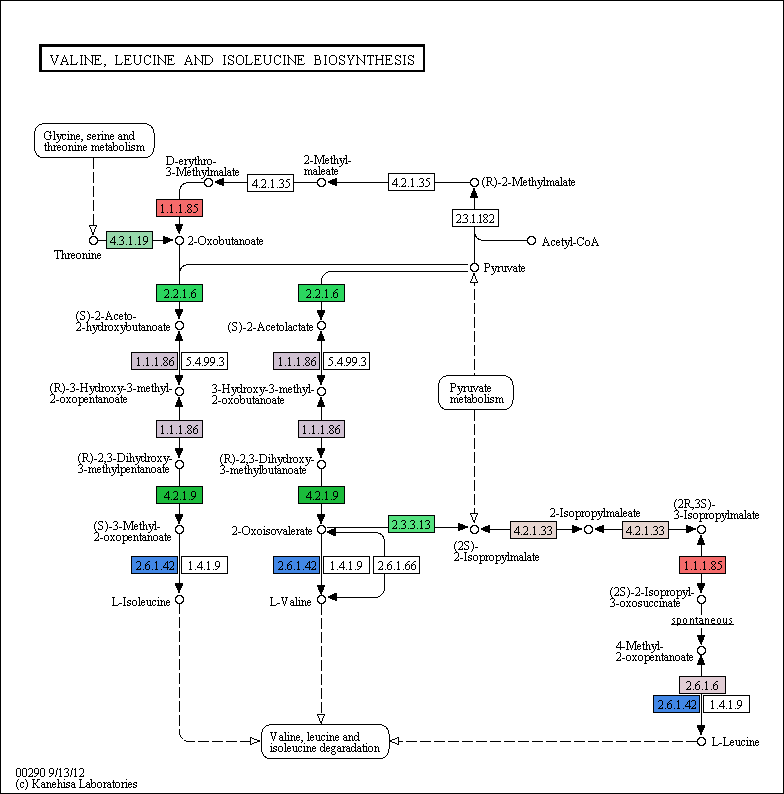

Supplement: Additional file 7: — KEGG pathway annotation. A zip compressed file with a list of KEGGs pathways, graphics in png format, and a file with a comparison with KEGGs pathways of potato and tomato. (ZIP 4361 kb) [file 12864_2016_2656_MOESM7_ESM.zip › Pathway representations/map00290_20150305160505.png]

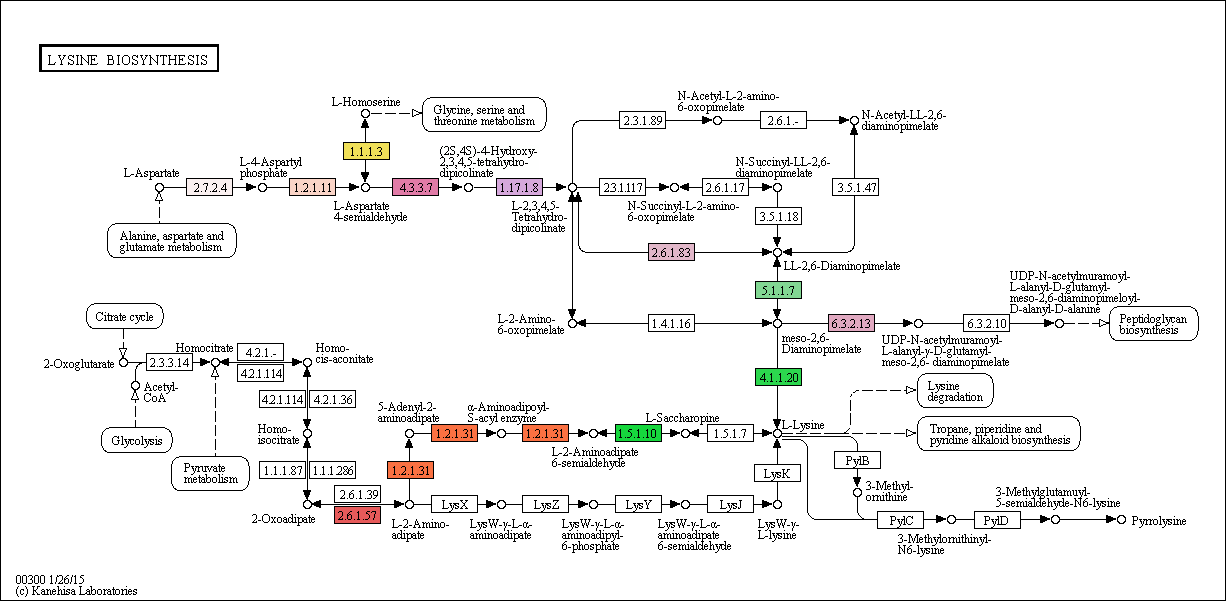

Supplement: Additional file 7: — KEGG pathway annotation. A zip compressed file with a list of KEGGs pathways, graphics in png format, and a file with a comparison with KEGGs pathways of potato and tomato. (ZIP 4361 kb) [file 12864_2016_2656_MOESM7_ESM.zip › Pathway representations/map00300_20150305160854.png]

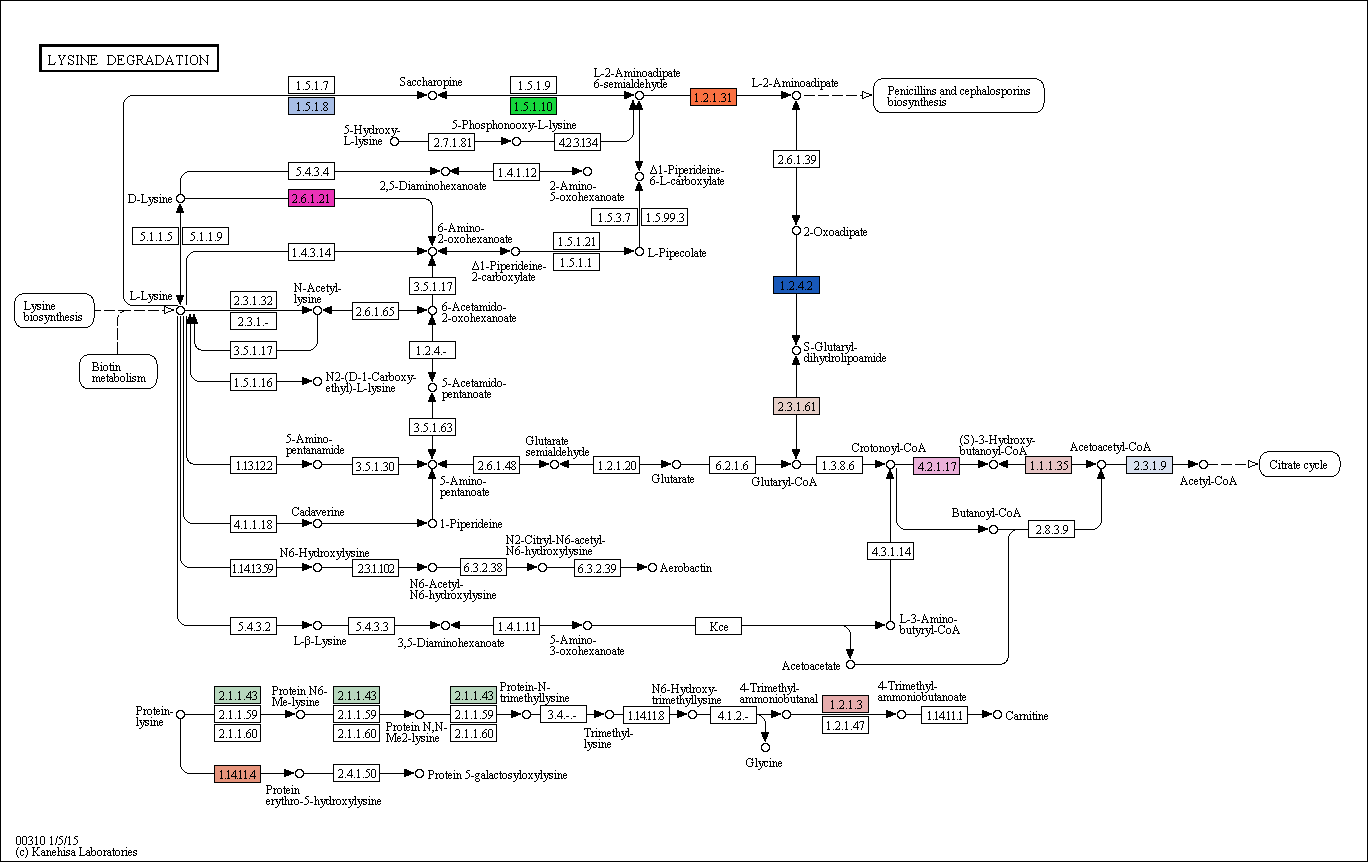

Supplement: Additional file 7: — KEGG pathway annotation. A zip compressed file with a list of KEGGs pathways, graphics in png format, and a file with a comparison with KEGGs pathways of potato and tomato. (ZIP 4361 kb) [file 12864_2016_2656_MOESM7_ESM.zip › Pathway representations/map00310_20150305160714.png]

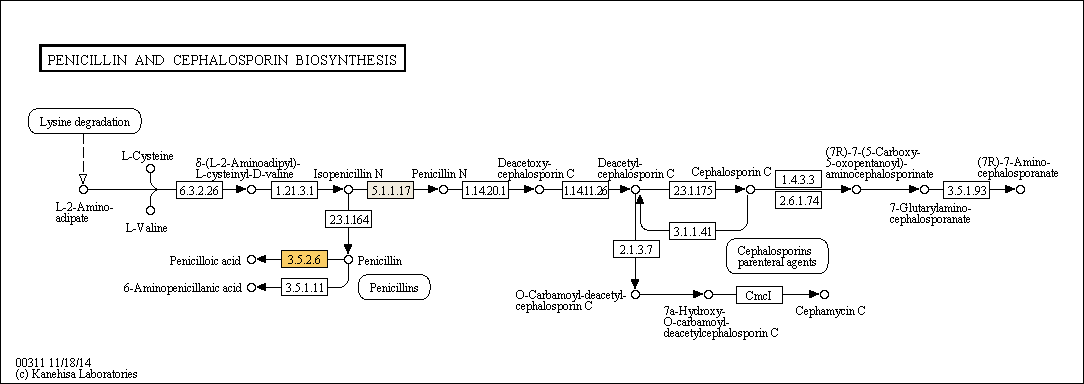

Supplement: Additional file 7: — KEGG pathway annotation. A zip compressed file with a list of KEGGs pathways, graphics in png format, and a file with a comparison with KEGGs pathways of potato and tomato. (ZIP 4361 kb) [file 12864_2016_2656_MOESM7_ESM.zip › Pathway representations/map00311_20150305160704.png]

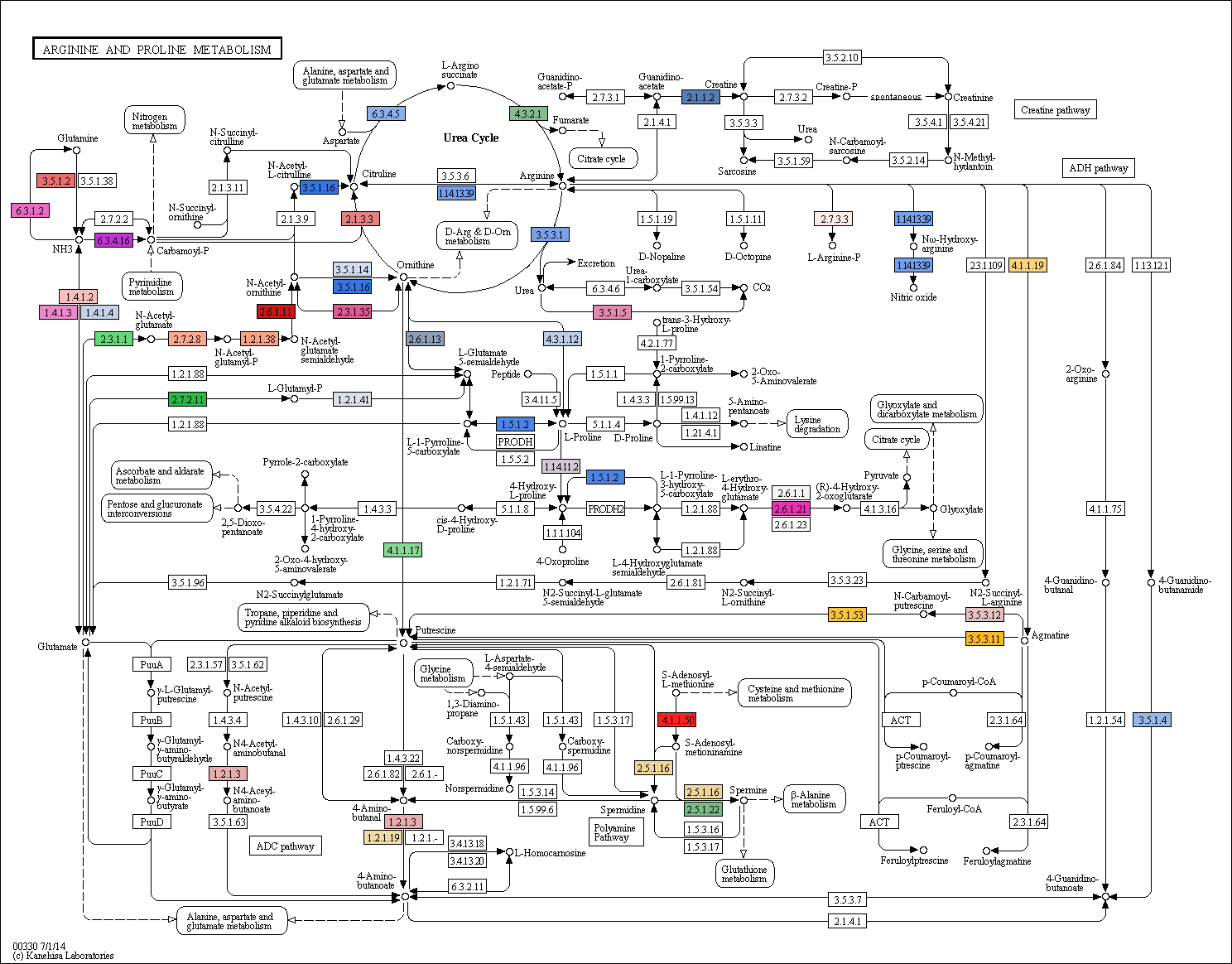

Supplement: Additional file 7: — KEGG pathway annotation. A zip compressed file with a list of KEGGs pathways, graphics in png format, and a file with a comparison with KEGGs pathways of potato and tomato. (ZIP 4361 kb) [file 12864_2016_2656_MOESM7_ESM.zip › Pathway representations/map00330_20150305160453.png]

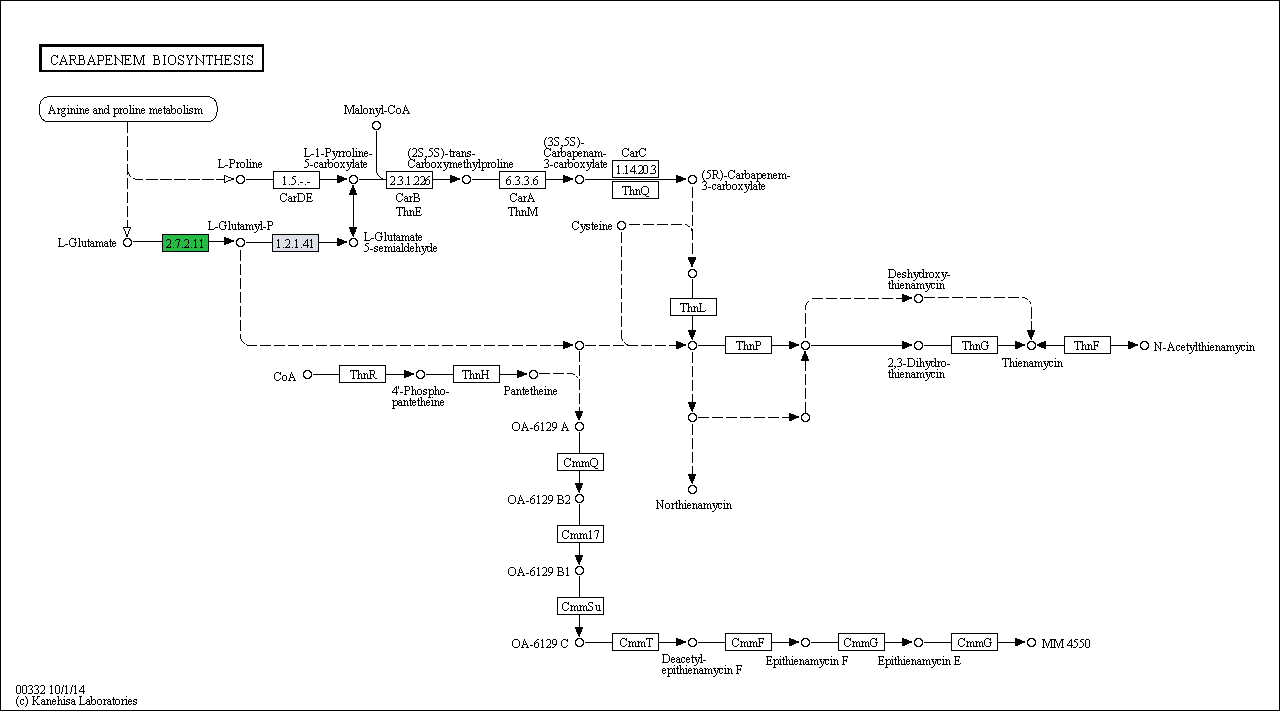

Supplement: Additional file 7: — KEGG pathway annotation. A zip compressed file with a list of KEGGs pathways, graphics in png format, and a file with a comparison with KEGGs pathways of potato and tomato. (ZIP 4361 kb) [file 12864_2016_2656_MOESM7_ESM.zip › Pathway representations/map00332_20150305160441.png]

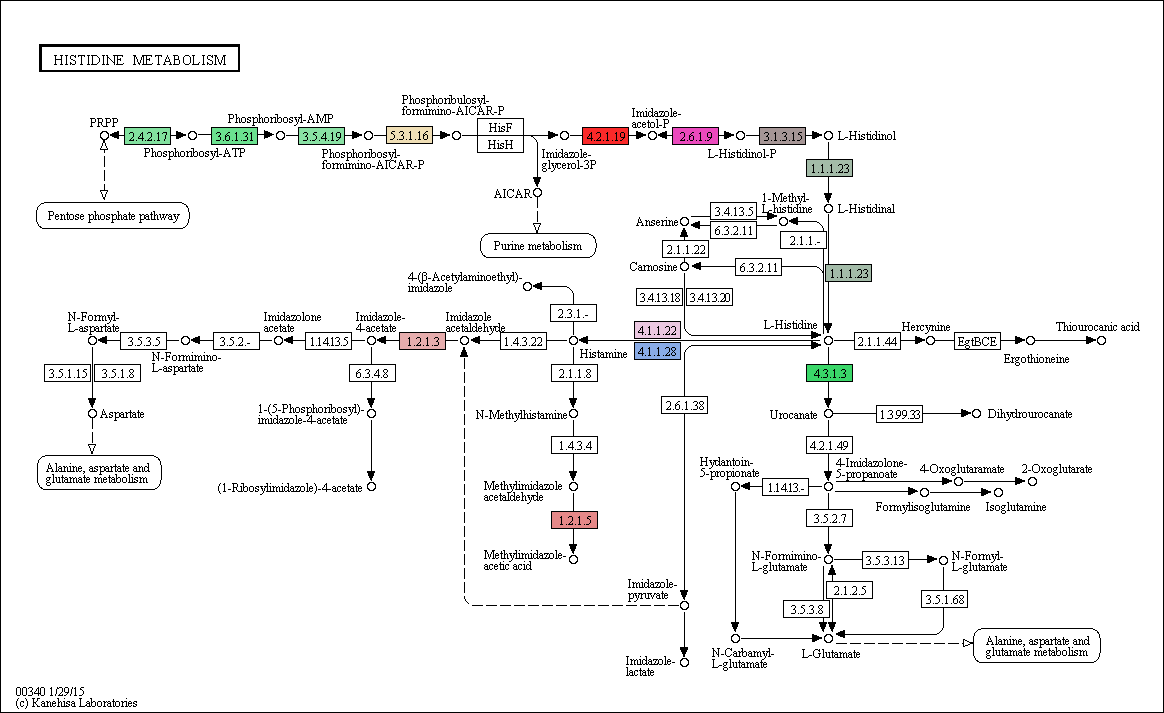

Supplement: Additional file 7: — KEGG pathway annotation. A zip compressed file with a list of KEGGs pathways, graphics in png format, and a file with a comparison with KEGGs pathways of potato and tomato. (ZIP 4361 kb) [file 12864_2016_2656_MOESM7_ESM.zip › Pathway representations/map00340_20150305161632.png]

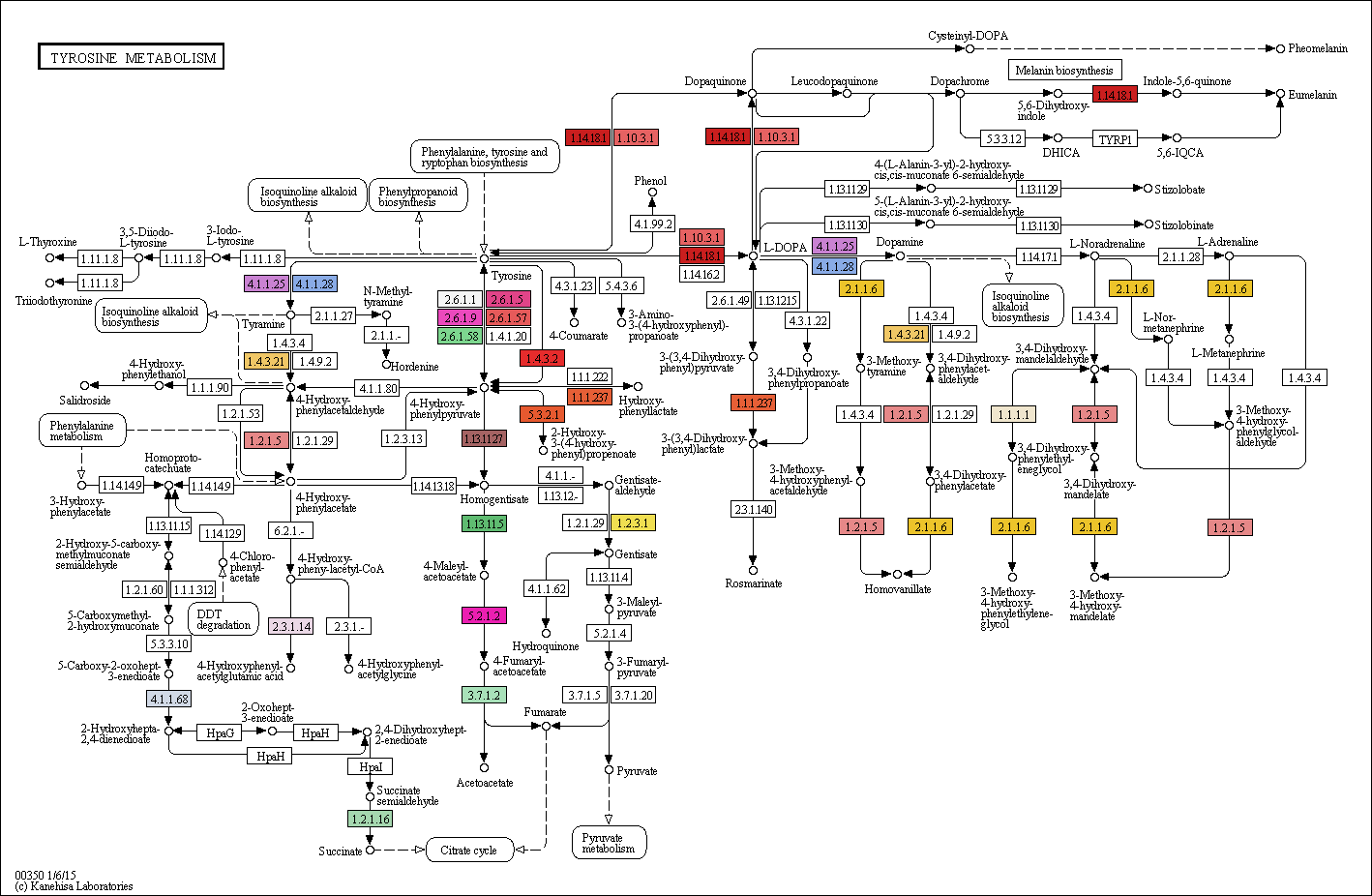

Supplement: Additional file 7: — KEGG pathway annotation. A zip compressed file with a list of KEGGs pathways, graphics in png format, and a file with a comparison with KEGGs pathways of potato and tomato. (ZIP 4361 kb) [file 12864_2016_2656_MOESM7_ESM.zip › Pathway representations/map00350_20150305161551.png]

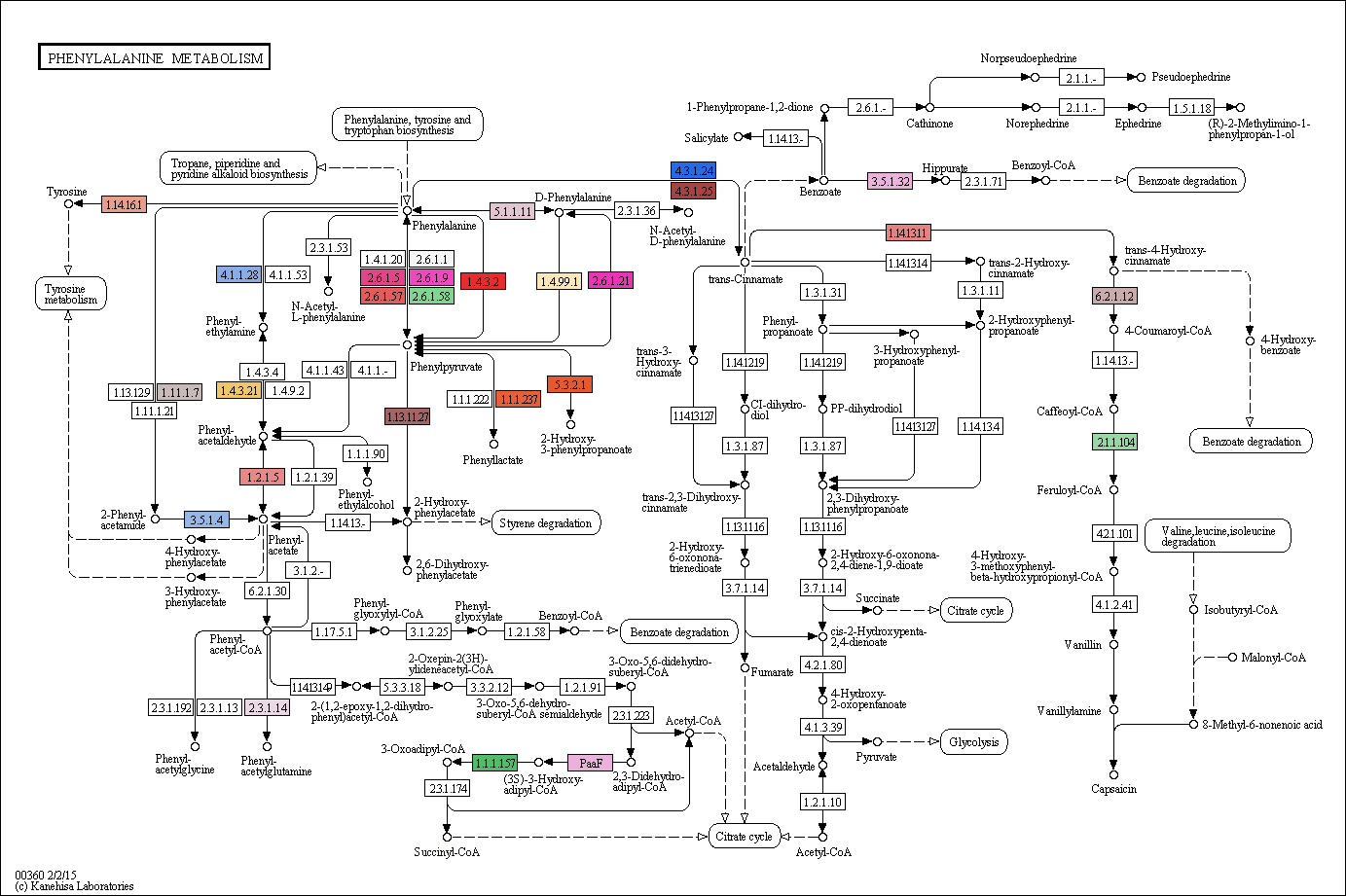

Supplement: Additional file 7: — KEGG pathway annotation. A zip compressed file with a list of KEGGs pathways, graphics in png format, and a file with a comparison with KEGGs pathways of potato and tomato. (ZIP 4361 kb) [file 12864_2016_2656_MOESM7_ESM.zip › Pathway representations/map00360_20150305161527.png]

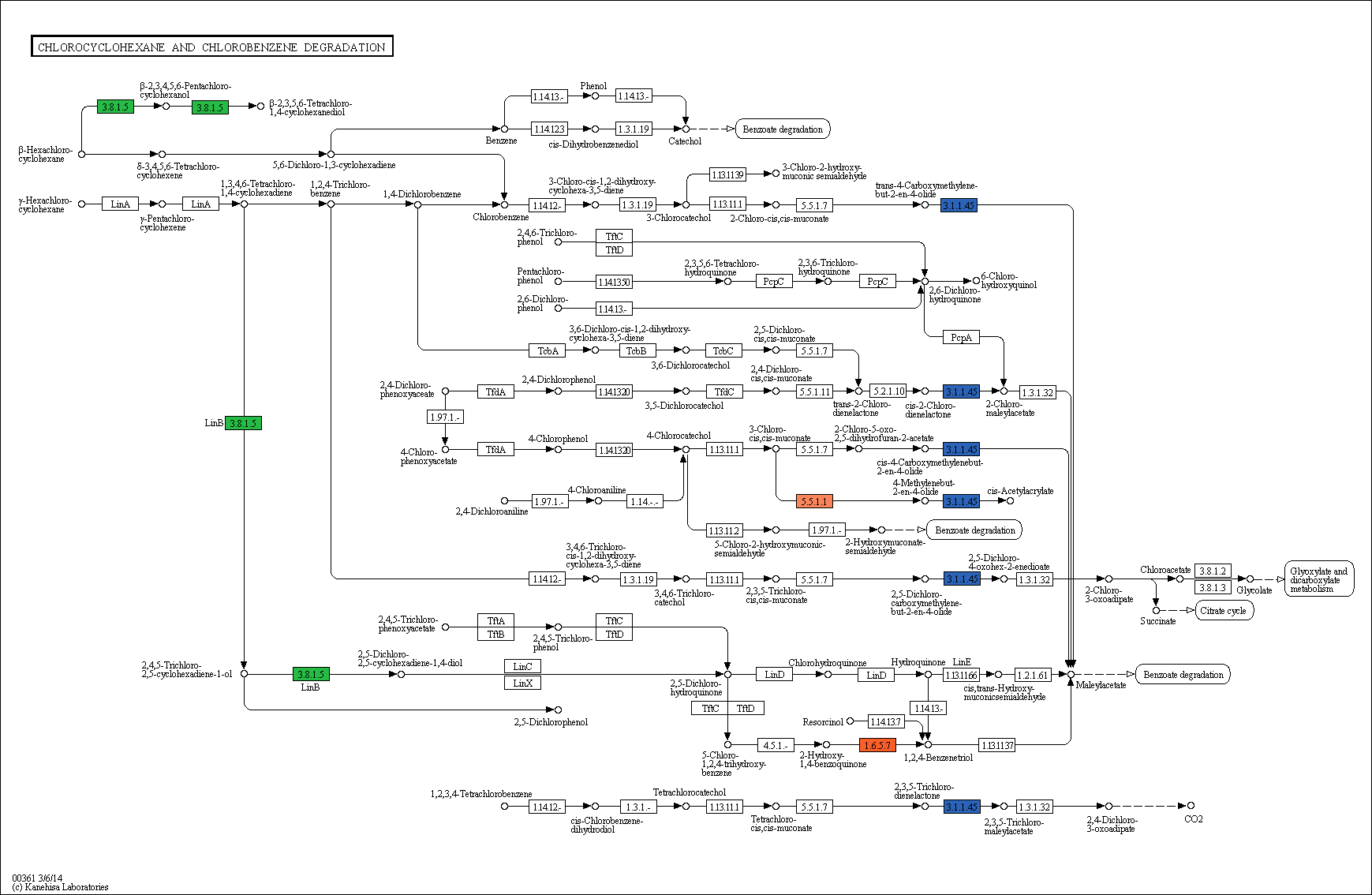

Supplement: Additional file 7: — KEGG pathway annotation. A zip compressed file with a list of KEGGs pathways, graphics in png format, and a file with a comparison with KEGGs pathways of potato and tomato. (ZIP 4361 kb) [file 12864_2016_2656_MOESM7_ESM.zip › Pathway representations/map00361_20150305161518.png]

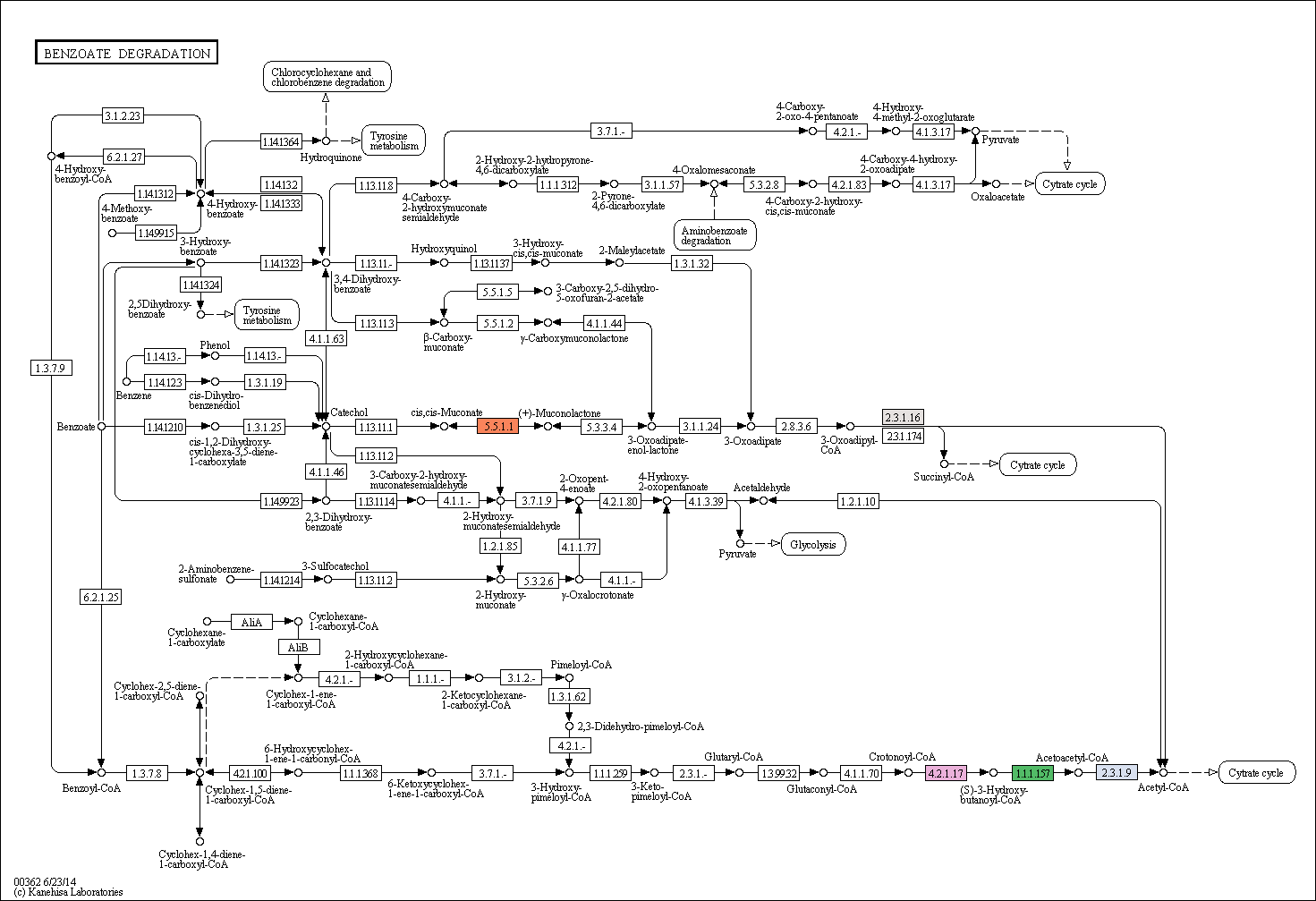

Supplement: Additional file 7: — KEGG pathway annotation. A zip compressed file with a list of KEGGs pathways, graphics in png format, and a file with a comparison with KEGGs pathways of potato and tomato. (ZIP 4361 kb) [file 12864_2016_2656_MOESM7_ESM.zip › Pathway representations/map00362_20150305161513.png]

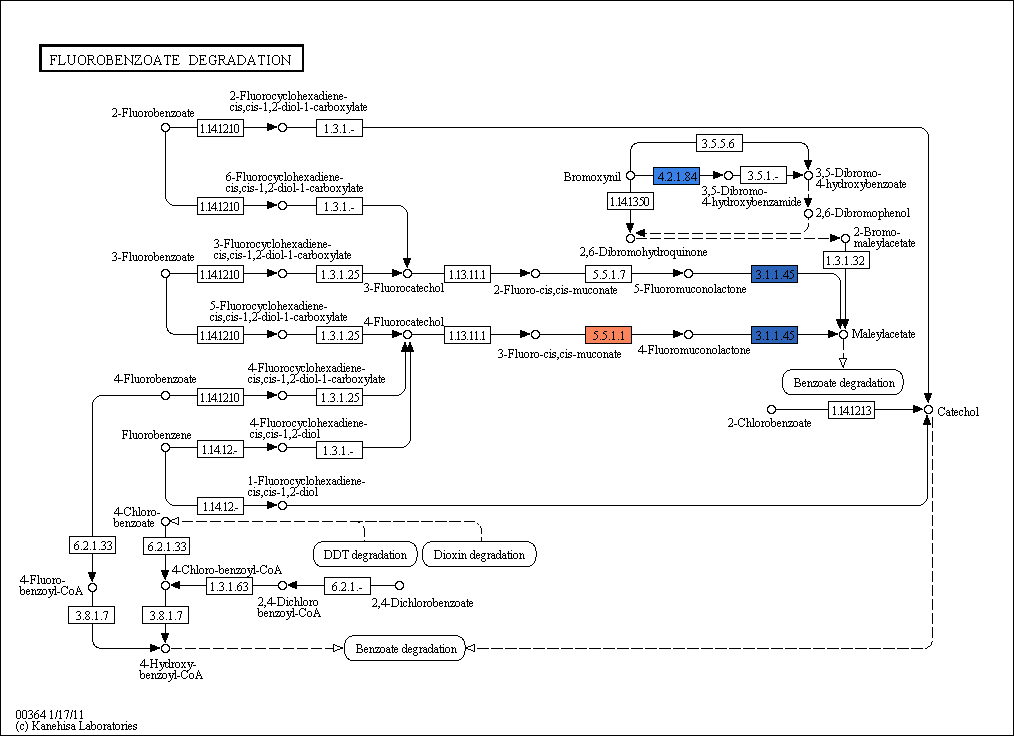

Supplement: Additional file 7: — KEGG pathway annotation. A zip compressed file with a list of KEGGs pathways, graphics in png format, and a file with a comparison with KEGGs pathways of potato and tomato. (ZIP 4361 kb) [file 12864_2016_2656_MOESM7_ESM.zip › Pathway representations/map00364_20150305161507.png]

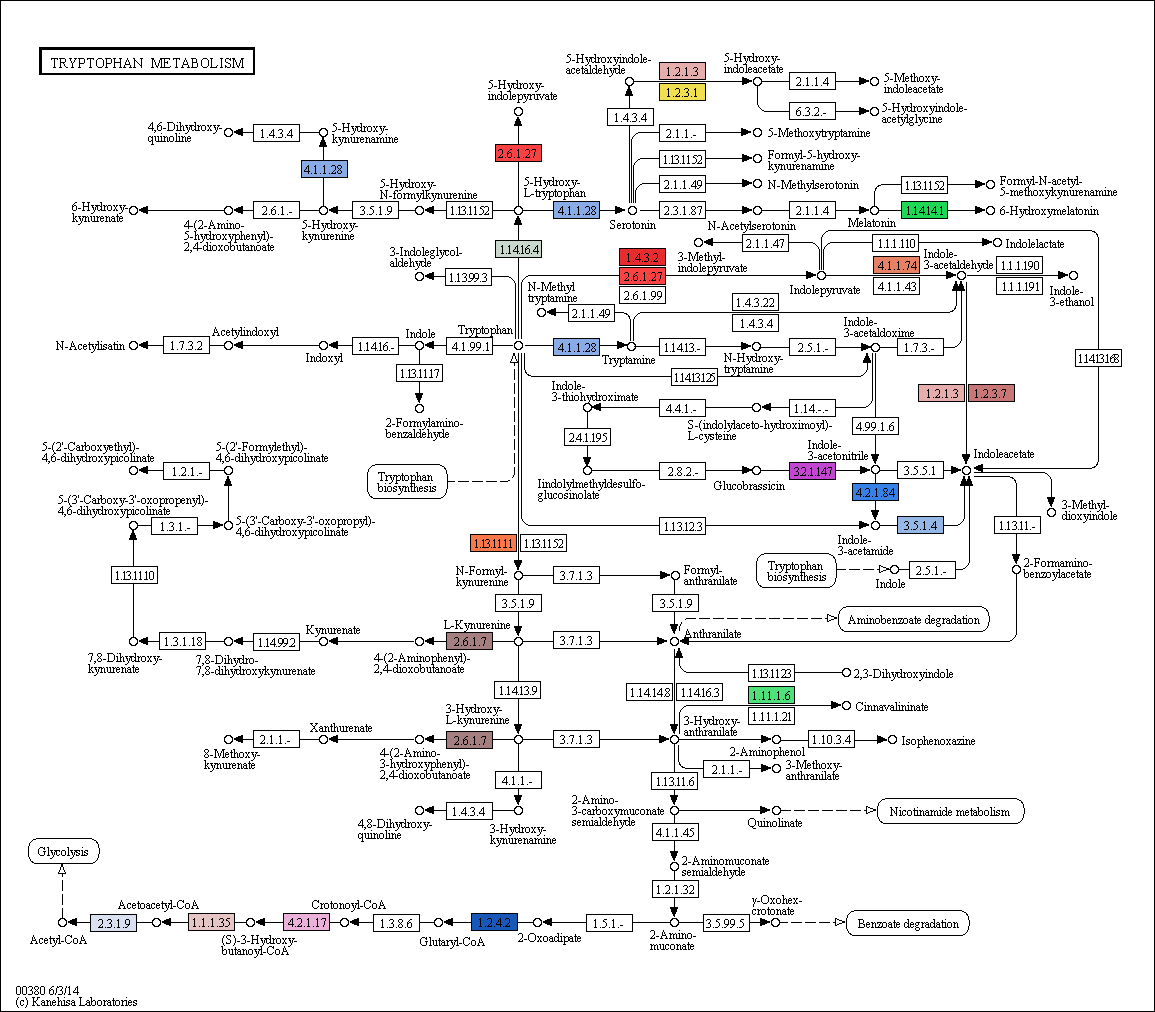

Supplement: Additional file 7: — KEGG pathway annotation. A zip compressed file with a list of KEGGs pathways, graphics in png format, and a file with a comparison with KEGGs pathways of potato and tomato. (ZIP 4361 kb) [file 12864_2016_2656_MOESM7_ESM.zip › Pathway representations/map00380_20150305161311.png]

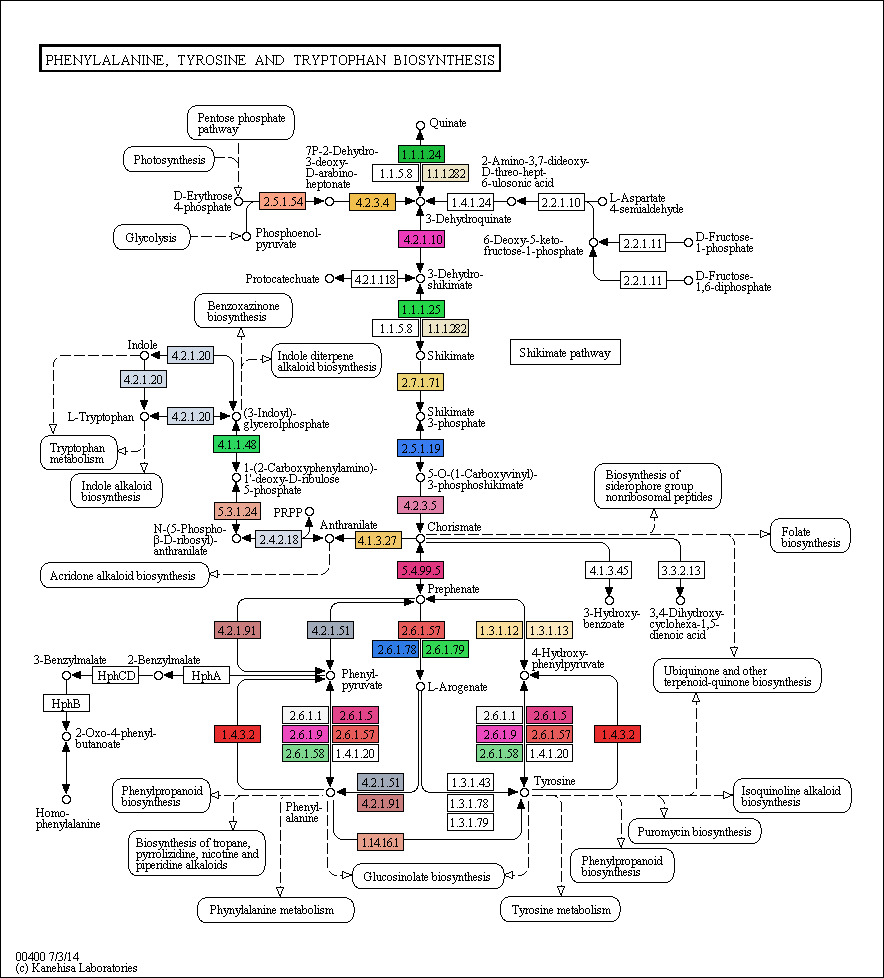

Supplement: Additional file 7: — KEGG pathway annotation. A zip compressed file with a list of KEGGs pathways, graphics in png format, and a file with a comparison with KEGGs pathways of potato and tomato. (ZIP 4361 kb) [file 12864_2016_2656_MOESM7_ESM.zip › Pathway representations/map00400_20150305161451.png]

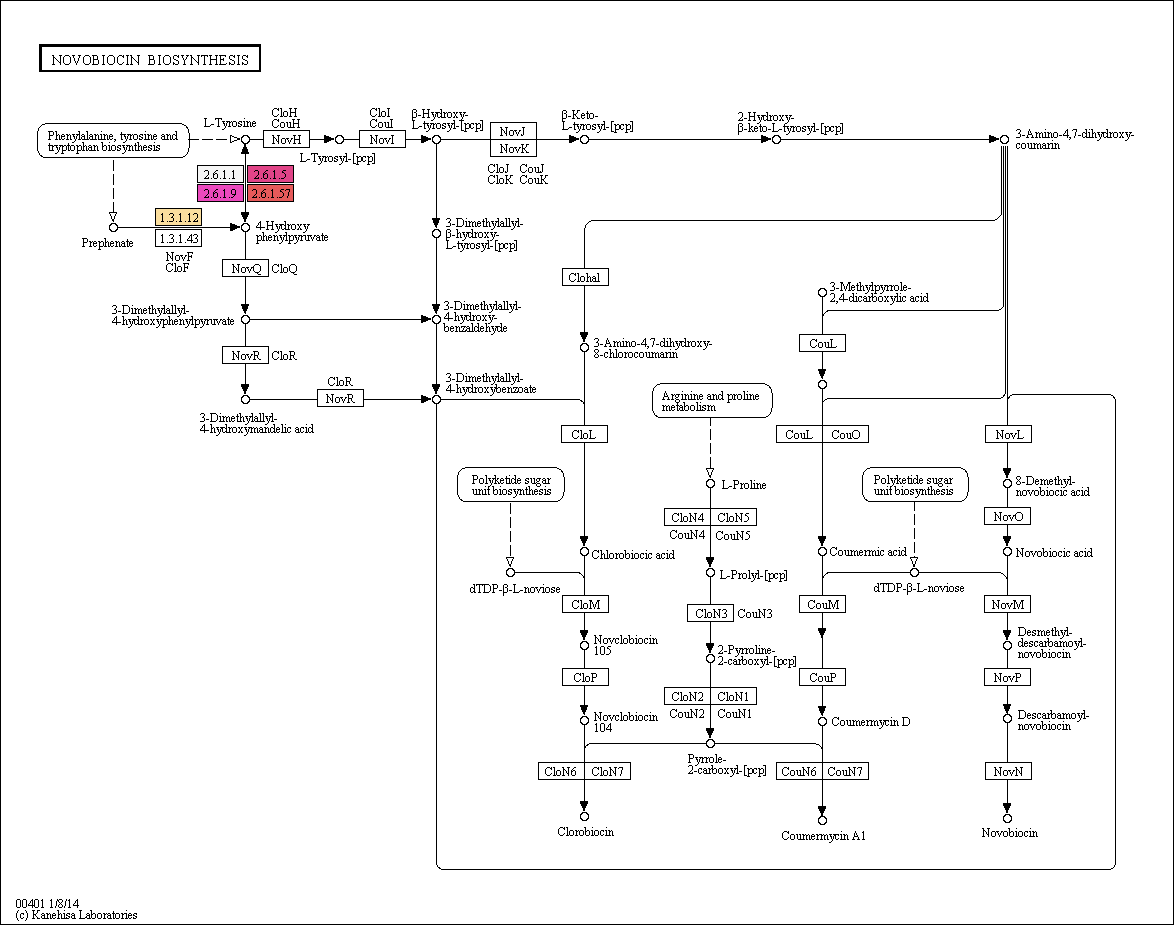

Supplement: Additional file 7: — KEGG pathway annotation. A zip compressed file with a list of KEGGs pathways, graphics in png format, and a file with a comparison with KEGGs pathways of potato and tomato. (ZIP 4361 kb) [file 12864_2016_2656_MOESM7_ESM.zip › Pathway representations/map00401_20150305161441.png]

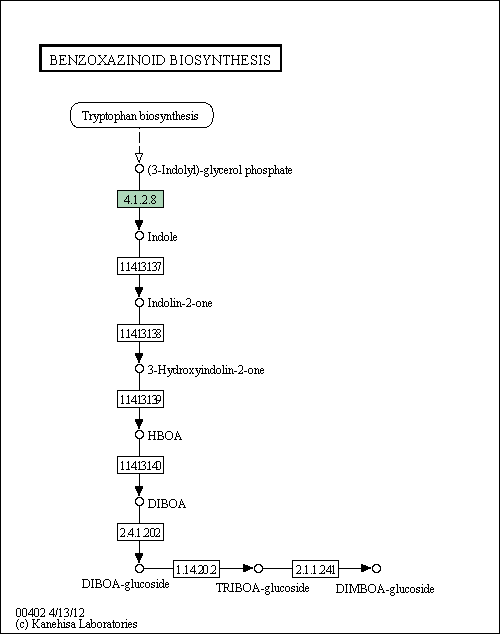

Supplement: Additional file 7: — KEGG pathway annotation. A zip compressed file with a list of KEGGs pathways, graphics in png format, and a file with a comparison with KEGGs pathways of potato and tomato. (ZIP 4361 kb) [file 12864_2016_2656_MOESM7_ESM.zip › Pathway representations/map00402_20150305161429.png]

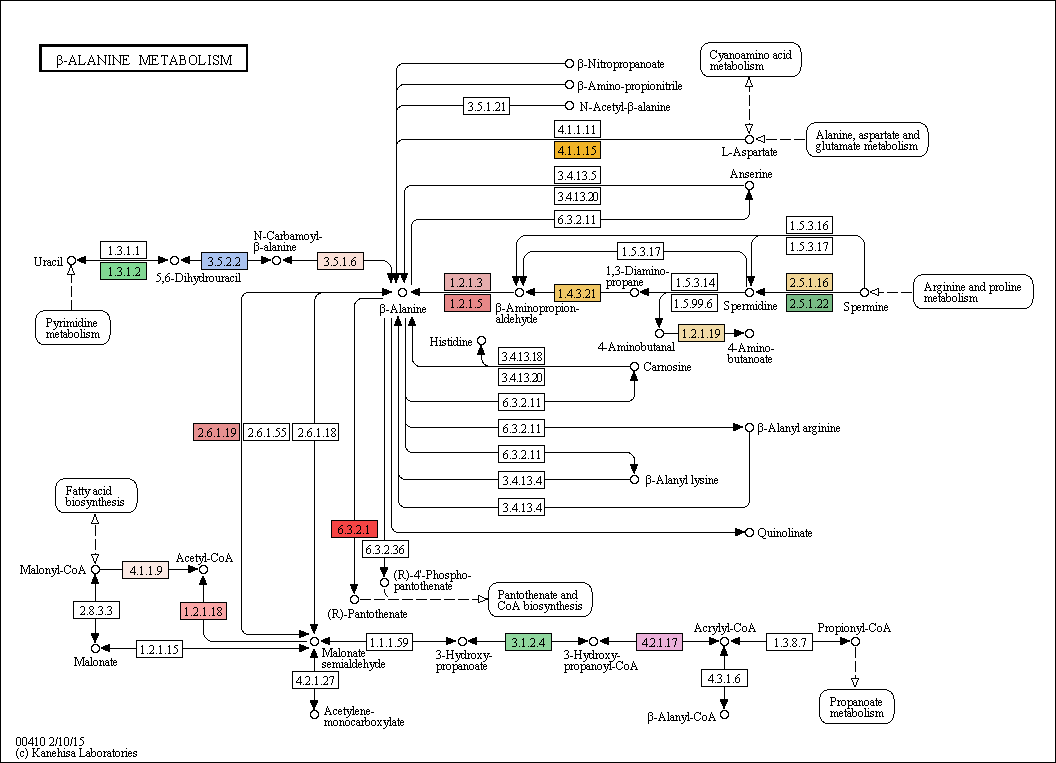

Supplement: Additional file 7: — KEGG pathway annotation. A zip compressed file with a list of KEGGs pathways, graphics in png format, and a file with a comparison with KEGGs pathways of potato and tomato. (ZIP 4361 kb) [file 12864_2016_2656_MOESM7_ESM.zip › Pathway representations/map00410_20150305161336.png]

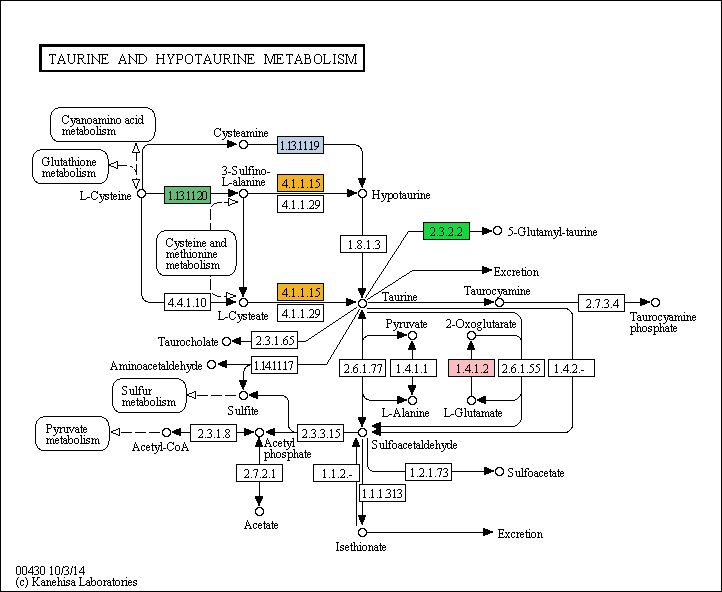

Supplement: Additional file 7: — KEGG pathway annotation. A zip compressed file with a list of KEGGs pathways, graphics in png format, and a file with a comparison with KEGGs pathways of potato and tomato. (ZIP 4361 kb) [file 12864_2016_2656_MOESM7_ESM.zip › Pathway representations/map00430_20150305161108.png]

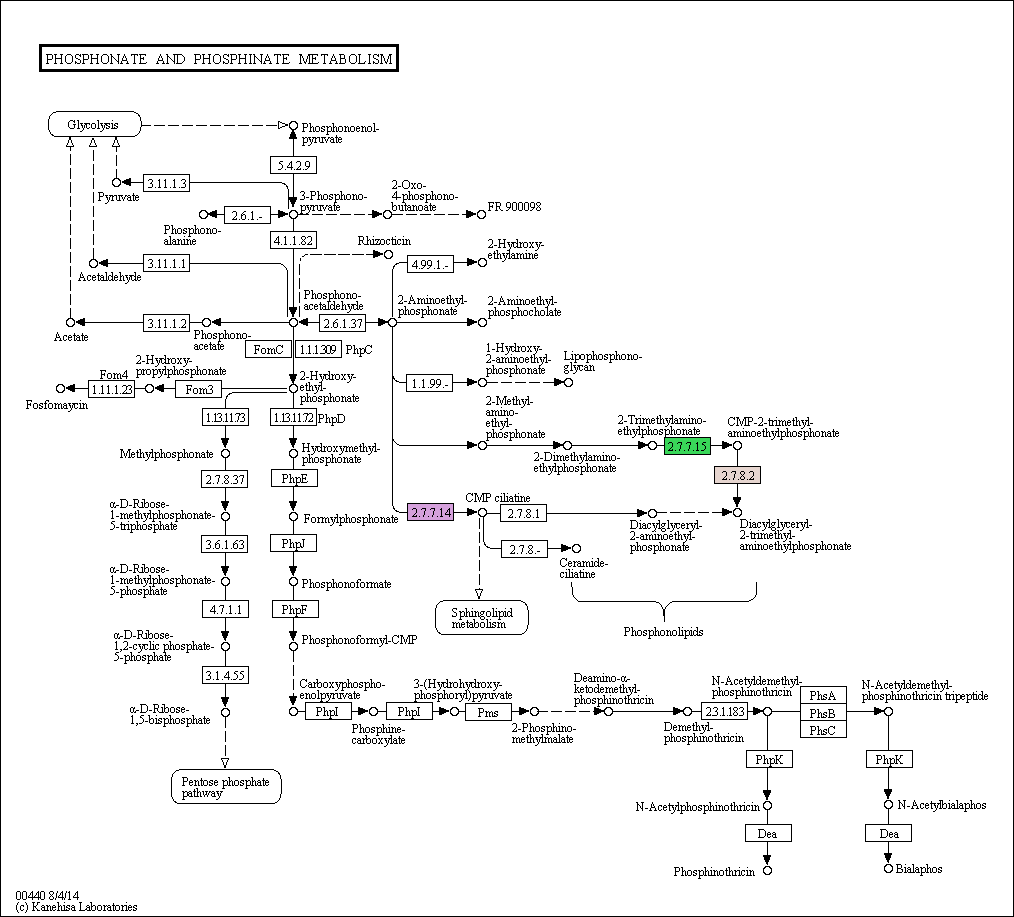

Supplement: Additional file 7: — KEGG pathway annotation. A zip compressed file with a list of KEGGs pathways, graphics in png format, and a file with a comparison with KEGGs pathways of potato and tomato. (ZIP 4361 kb) [file 12864_2016_2656_MOESM7_ESM.zip › Pathway representations/map00440_20150305161024.png]

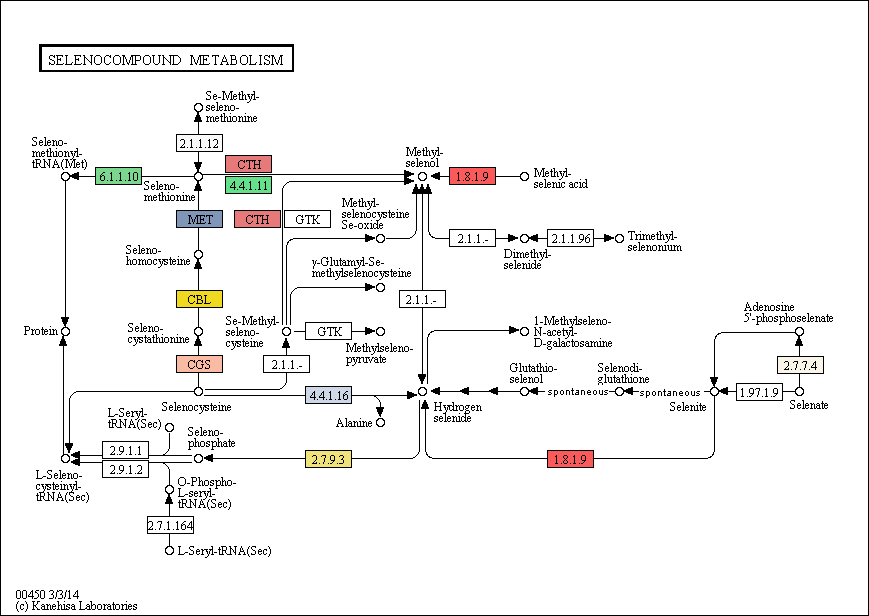

Supplement: Additional file 7: — KEGG pathway annotation. A zip compressed file with a list of KEGGs pathways, graphics in png format, and a file with a comparison with KEGGs pathways of potato and tomato. (ZIP 4361 kb) [file 12864_2016_2656_MOESM7_ESM.zip › Pathway representations/map00450_20150305160937.png]

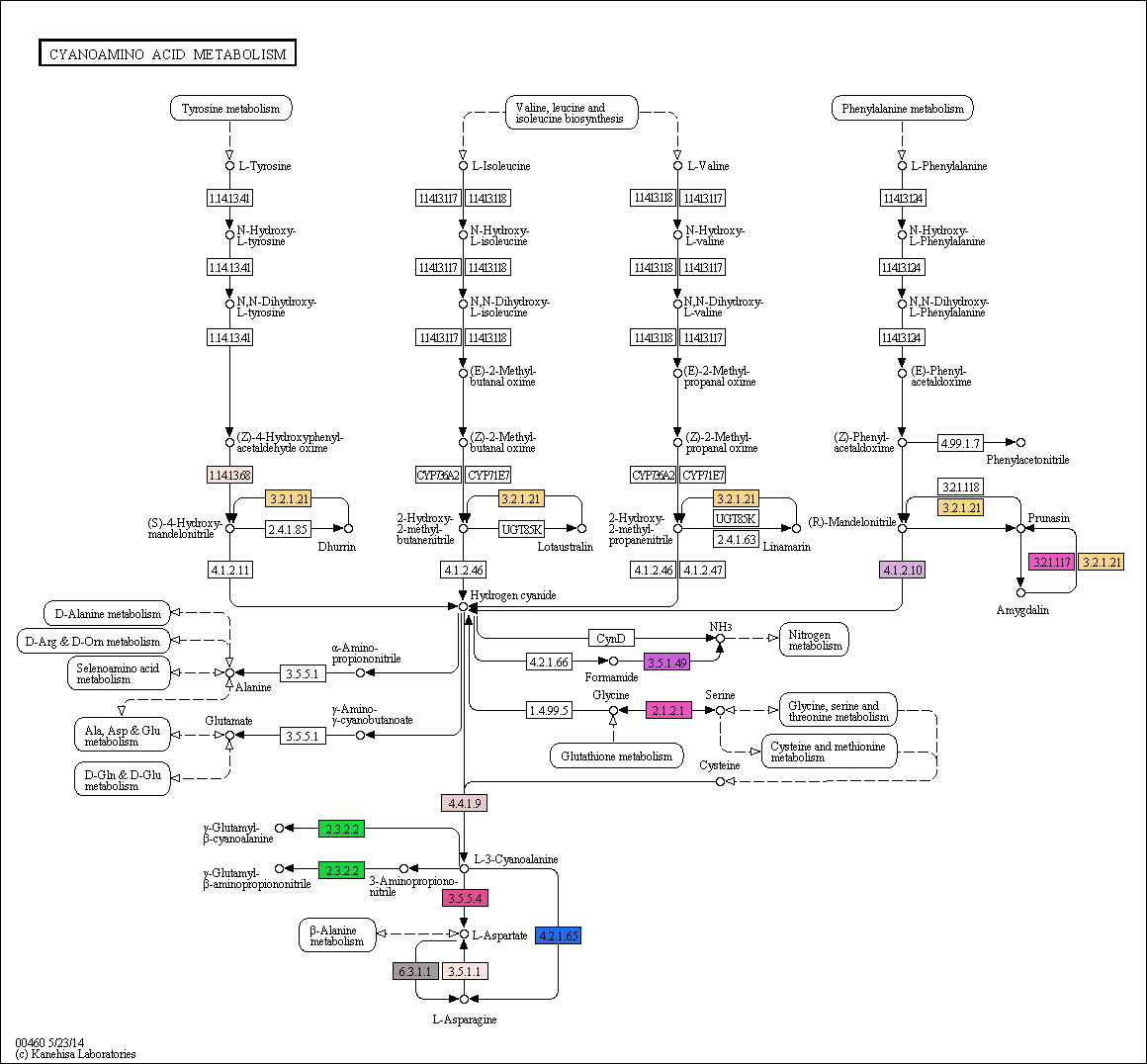

Supplement: Additional file 7: — KEGG pathway annotation. A zip compressed file with a list of KEGGs pathways, graphics in png format, and a file with a comparison with KEGGs pathways of potato and tomato. (ZIP 4361 kb) [file 12864_2016_2656_MOESM7_ESM.zip › Pathway representations/map00460_20150305160900.png]

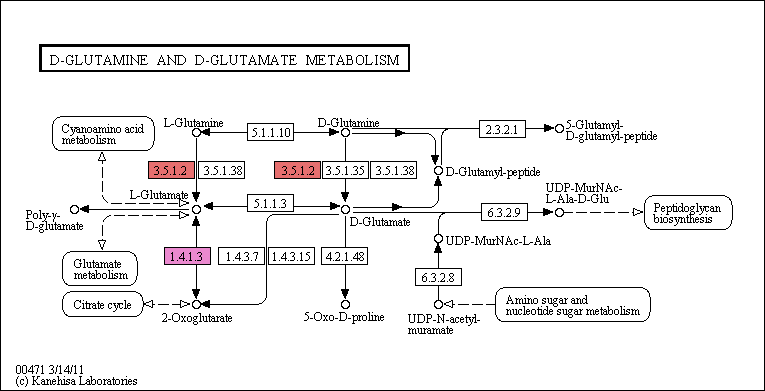

Supplement: Additional file 7: — KEGG pathway annotation. A zip compressed file with a list of KEGGs pathways, graphics in png format, and a file with a comparison with KEGGs pathways of potato and tomato. (ZIP 4361 kb) [file 12864_2016_2656_MOESM7_ESM.zip › Pathway representations/map00471_20150305160720.png]

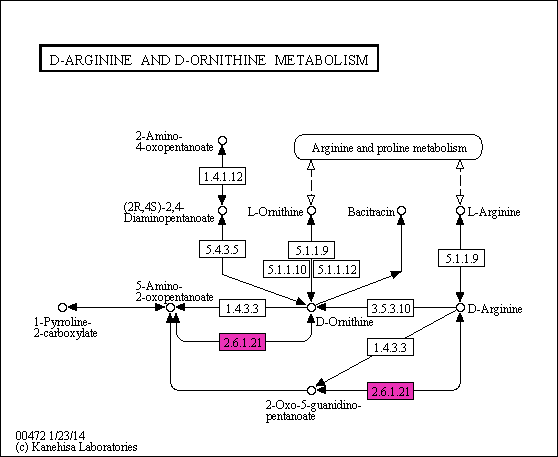

Supplement: Additional file 7: — KEGG pathway annotation. A zip compressed file with a list of KEGGs pathways, graphics in png format, and a file with a comparison with KEGGs pathways of potato and tomato. (ZIP 4361 kb) [file 12864_2016_2656_MOESM7_ESM.zip › Pathway representations/map00472_20150305160716.png]

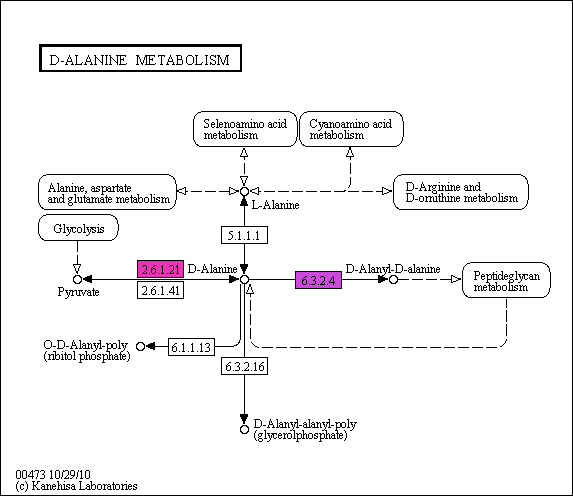

Supplement: Additional file 7: — KEGG pathway annotation. A zip compressed file with a list of KEGGs pathways, graphics in png format, and a file with a comparison with KEGGs pathways of potato and tomato. (ZIP 4361 kb) [file 12864_2016_2656_MOESM7_ESM.zip › Pathway representations/map00473_20150305160708.png]

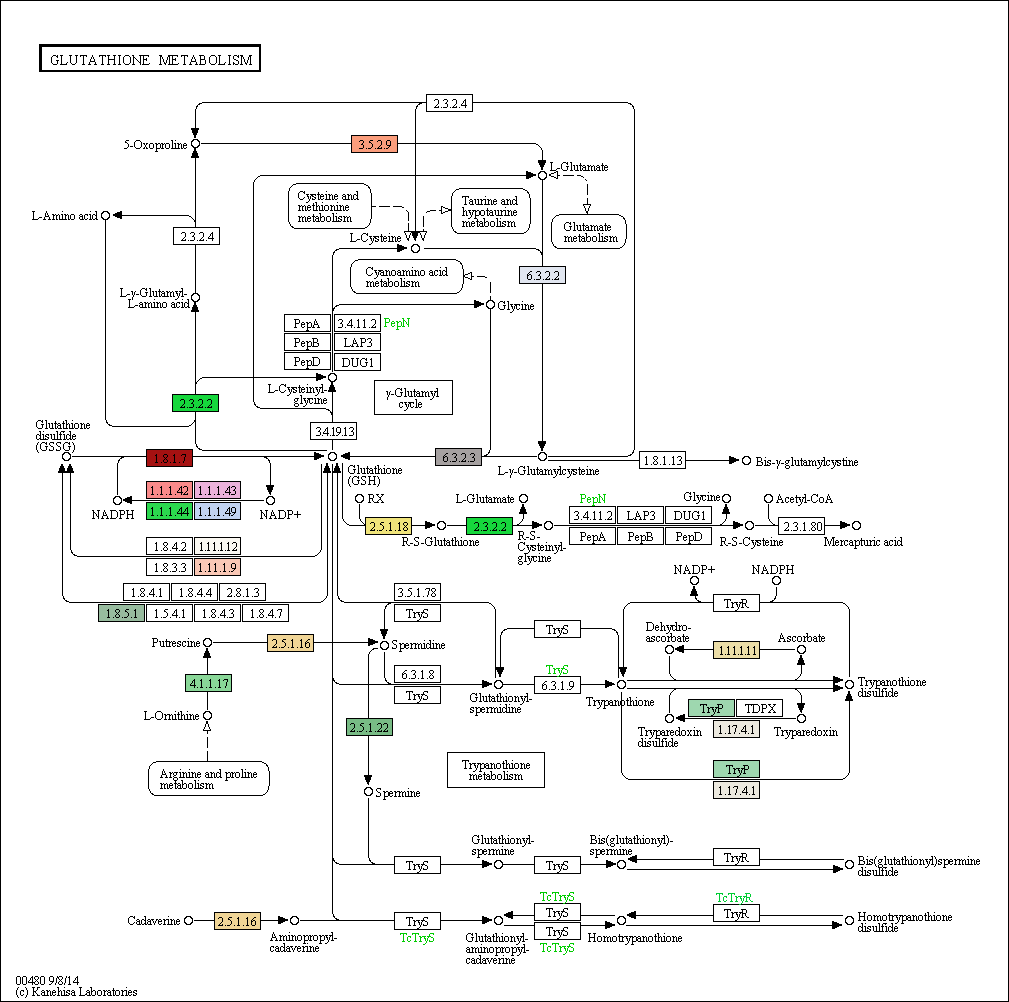

Supplement: Additional file 7: — KEGG pathway annotation. A zip compressed file with a list of KEGGs pathways, graphics in png format, and a file with a comparison with KEGGs pathways of potato and tomato. (ZIP 4361 kb) [file 12864_2016_2656_MOESM7_ESM.zip › Pathway representations/map00480_20150305160608.png]

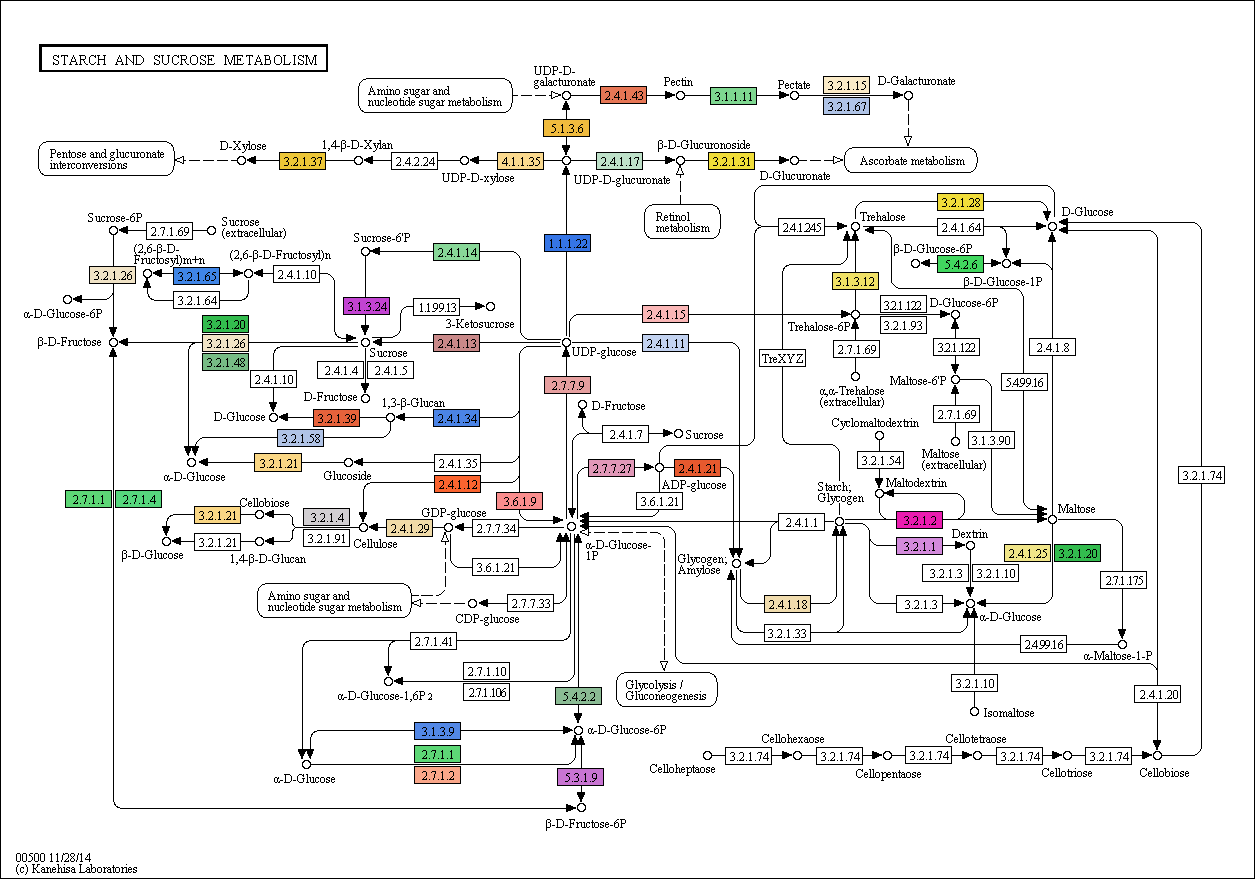

Supplement: Additional file 7: — KEGG pathway annotation. A zip compressed file with a list of KEGGs pathways, graphics in png format, and a file with a comparison with KEGGs pathways of potato and tomato. (ZIP 4361 kb) [file 12864_2016_2656_MOESM7_ESM.zip › Pathway representations/map00500_20150305160845.png]

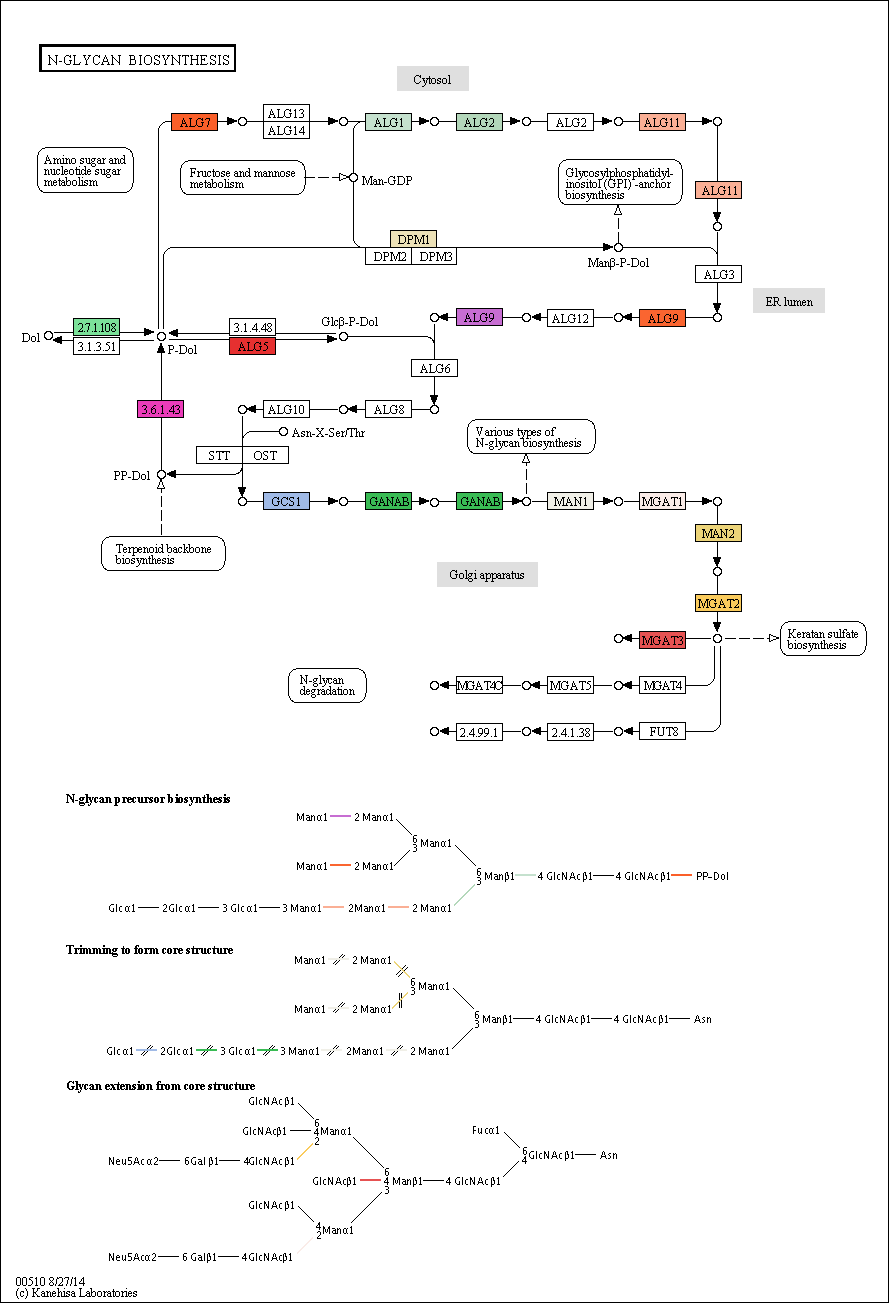

Supplement: Additional file 7: — KEGG pathway annotation. A zip compressed file with a list of KEGGs pathways, graphics in png format, and a file with a comparison with KEGGs pathways of potato and tomato. (ZIP 4361 kb) [file 12864_2016_2656_MOESM7_ESM.zip › Pathway representations/map00510_20150305160656.png]

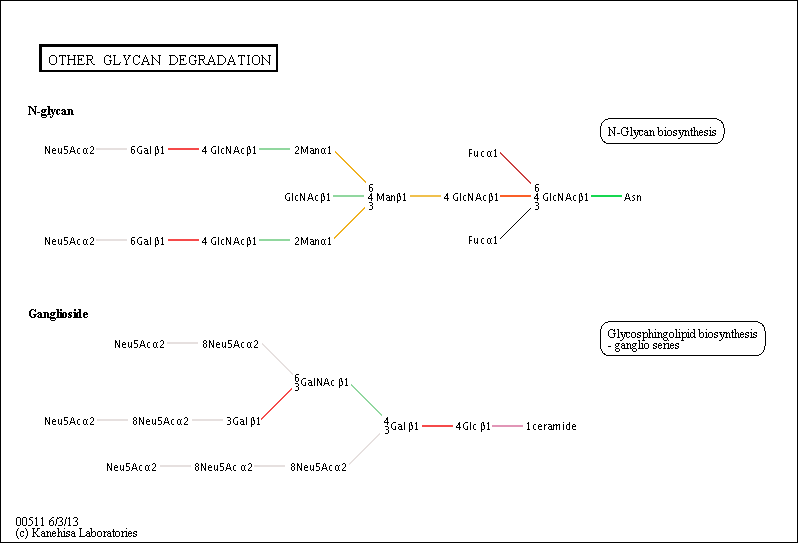

Supplement: Additional file 7: — KEGG pathway annotation. A zip compressed file with a list of KEGGs pathways, graphics in png format, and a file with a comparison with KEGGs pathways of potato and tomato. (ZIP 4361 kb) [file 12864_2016_2656_MOESM7_ESM.zip › Pathway representations/map00511_20150305160651.png]

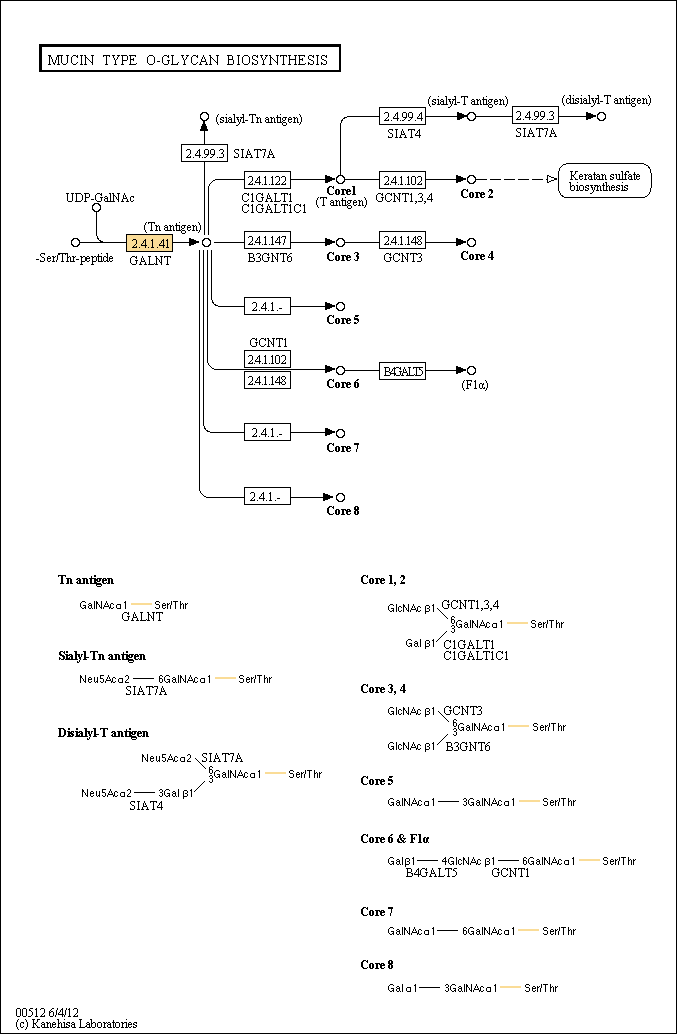

Supplement: Additional file 7: — KEGG pathway annotation. A zip compressed file with a list of KEGGs pathways, graphics in png format, and a file with a comparison with KEGGs pathways of potato and tomato. (ZIP 4361 kb) [file 12864_2016_2656_MOESM7_ESM.zip › Pathway representations/map00512_20150305160646.png]

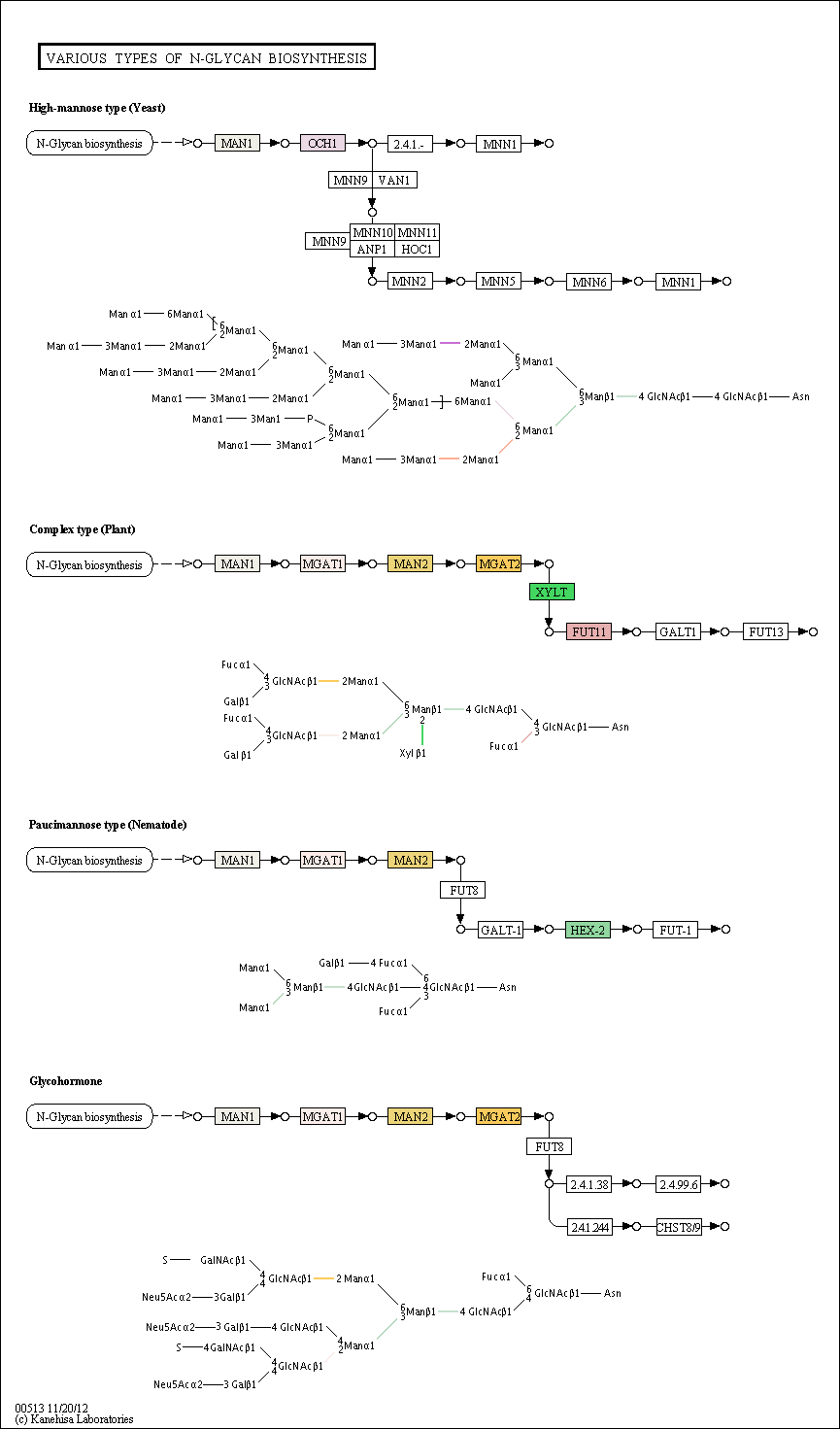

Supplement: Additional file 7: — KEGG pathway annotation. A zip compressed file with a list of KEGGs pathways, graphics in png format, and a file with a comparison with KEGGs pathways of potato and tomato. (ZIP 4361 kb) [file 12864_2016_2656_MOESM7_ESM.zip › Pathway representations/map00513_20150305160644.png]

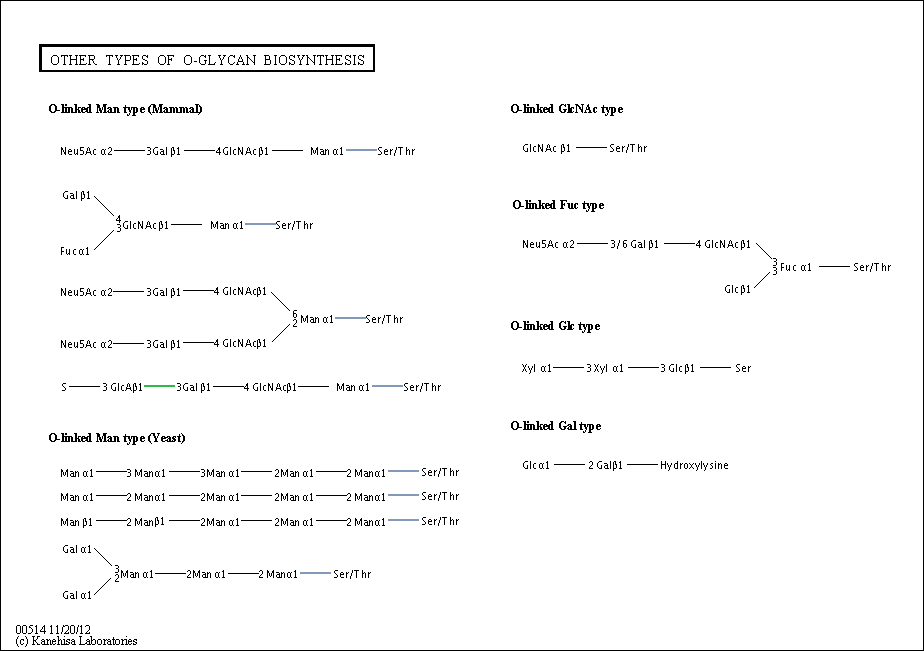

Supplement: Additional file 7: — KEGG pathway annotation. A zip compressed file with a list of KEGGs pathways, graphics in png format, and a file with a comparison with KEGGs pathways of potato and tomato. (ZIP 4361 kb) [file 12864_2016_2656_MOESM7_ESM.zip › Pathway representations/map00514_20150305160639.png]

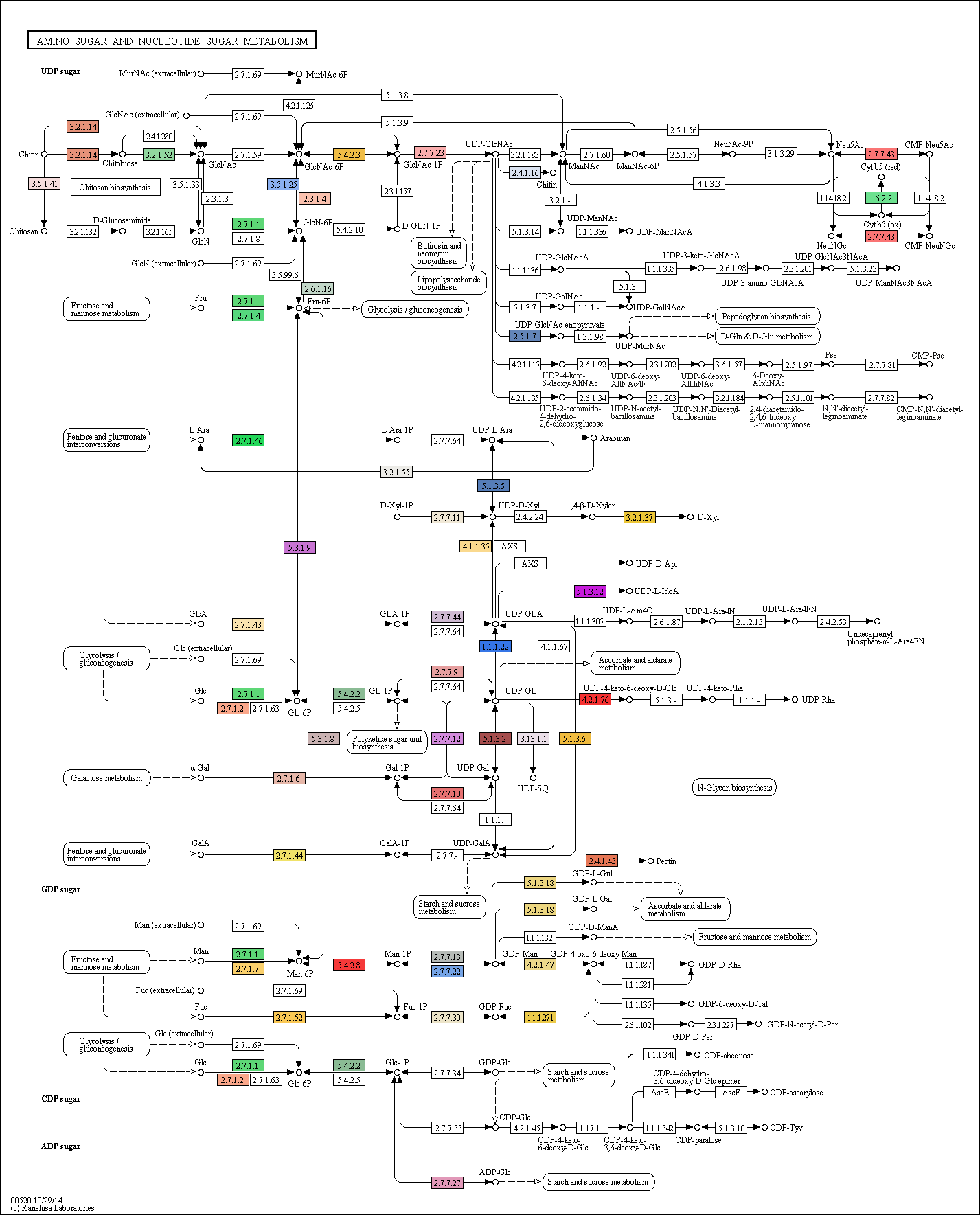

Supplement: Additional file 7: — KEGG pathway annotation. A zip compressed file with a list of KEGGs pathways, graphics in png format, and a file with a comparison with KEGGs pathways of potato and tomato. (ZIP 4361 kb) [file 12864_2016_2656_MOESM7_ESM.zip › Pathway representations/map00520_20150305160547.png]

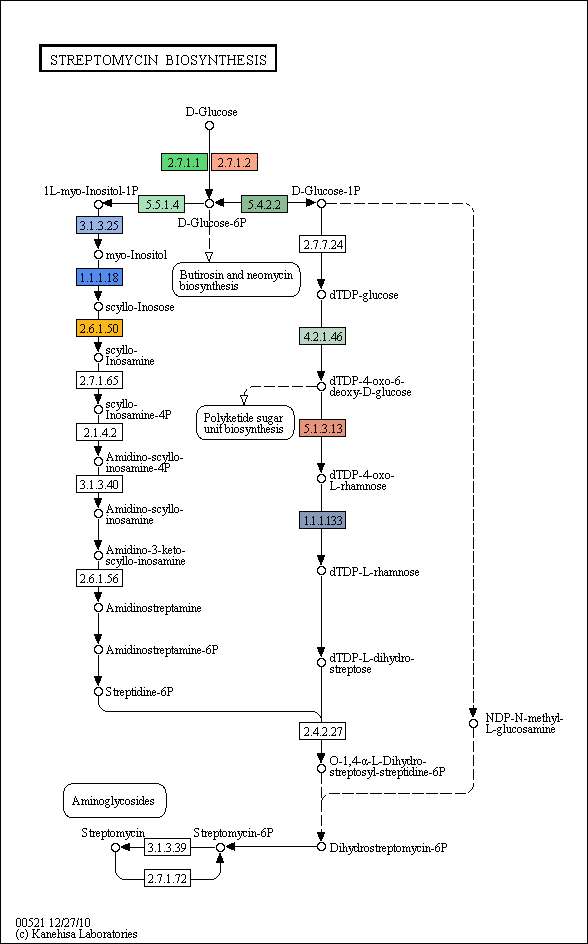

Supplement: Additional file 7: — KEGG pathway annotation. A zip compressed file with a list of KEGGs pathways, graphics in png format, and a file with a comparison with KEGGs pathways of potato and tomato. (ZIP 4361 kb) [file 12864_2016_2656_MOESM7_ESM.zip › Pathway representations/map00521_20150305160530.png]

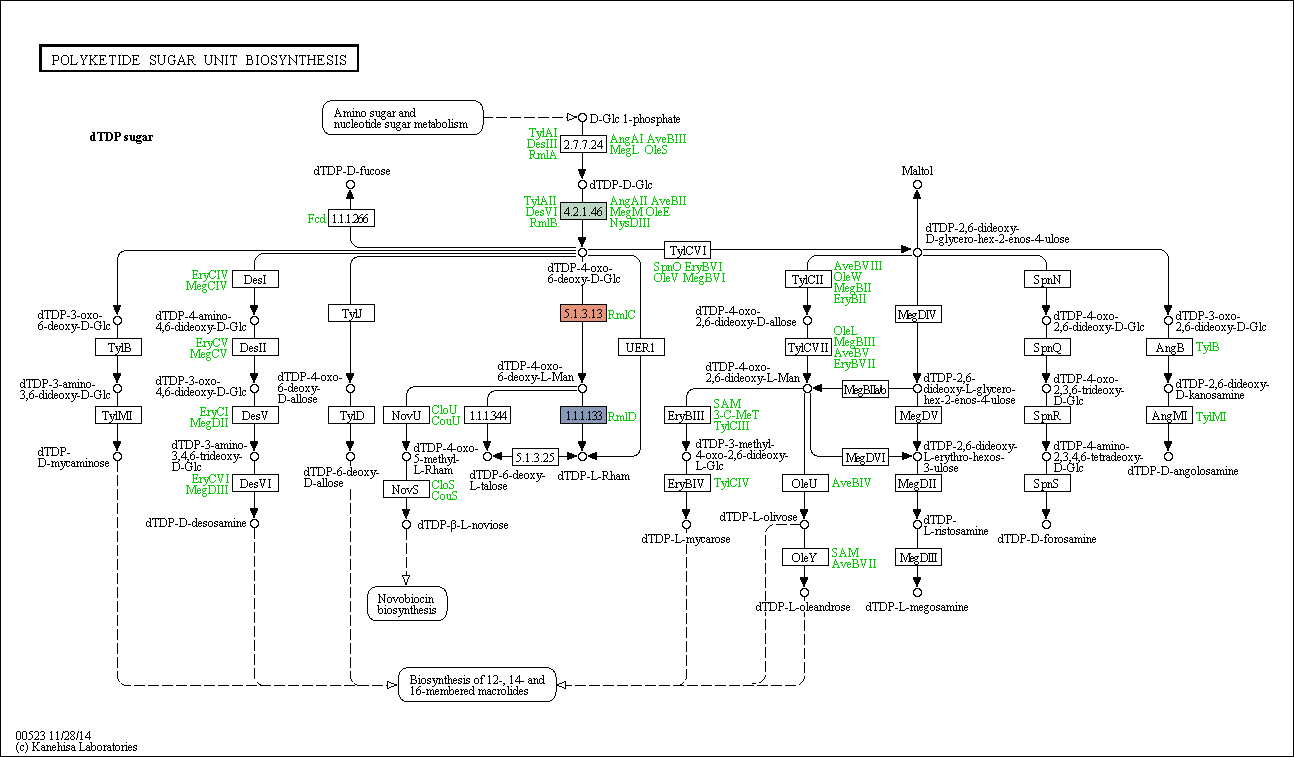

Supplement: Additional file 7: — KEGG pathway annotation. A zip compressed file with a list of KEGGs pathways, graphics in png format, and a file with a comparison with KEGGs pathways of potato and tomato. (ZIP 4361 kb) [file 12864_2016_2656_MOESM7_ESM.zip › Pathway representations/map00523_20150305160526.png]

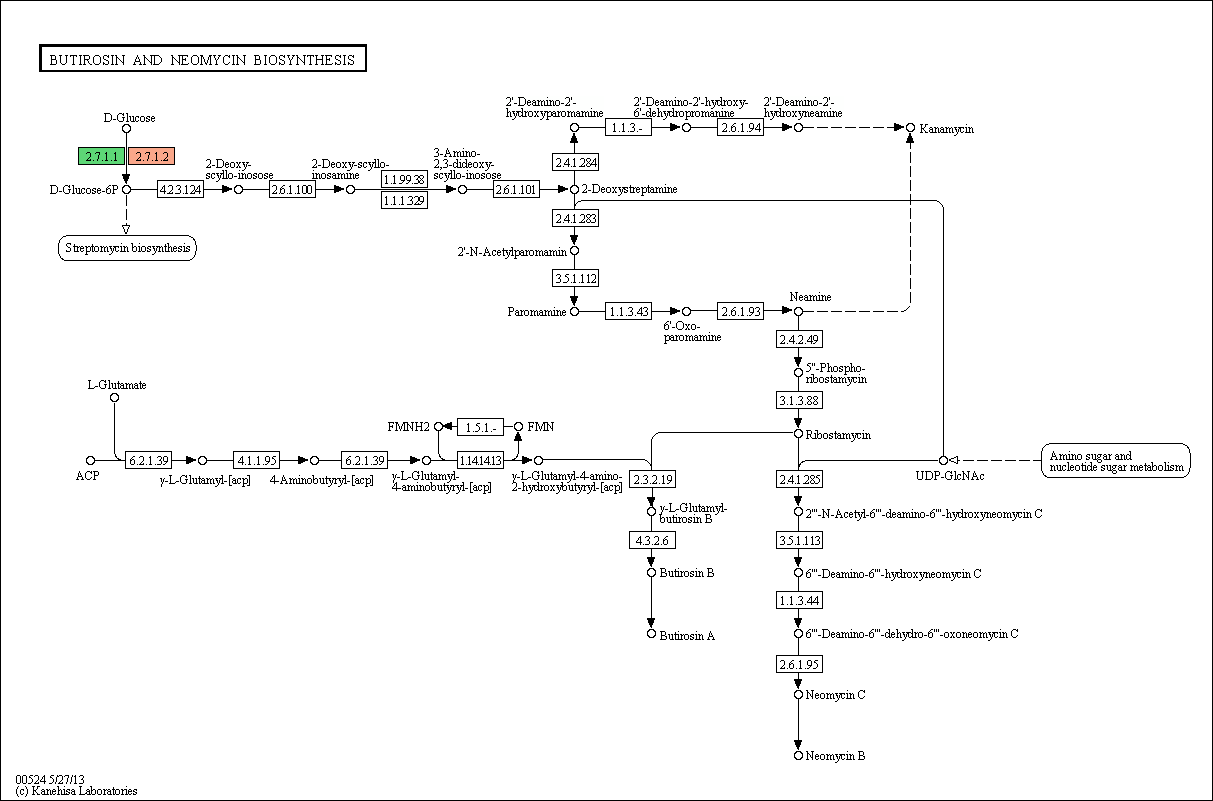

Supplement: Additional file 7: — KEGG pathway annotation. A zip compressed file with a list of KEGGs pathways, graphics in png format, and a file with a comparison with KEGGs pathways of potato and tomato. (ZIP 4361 kb) [file 12864_2016_2656_MOESM7_ESM.zip › Pathway representations/map00524_20150305160521.png]

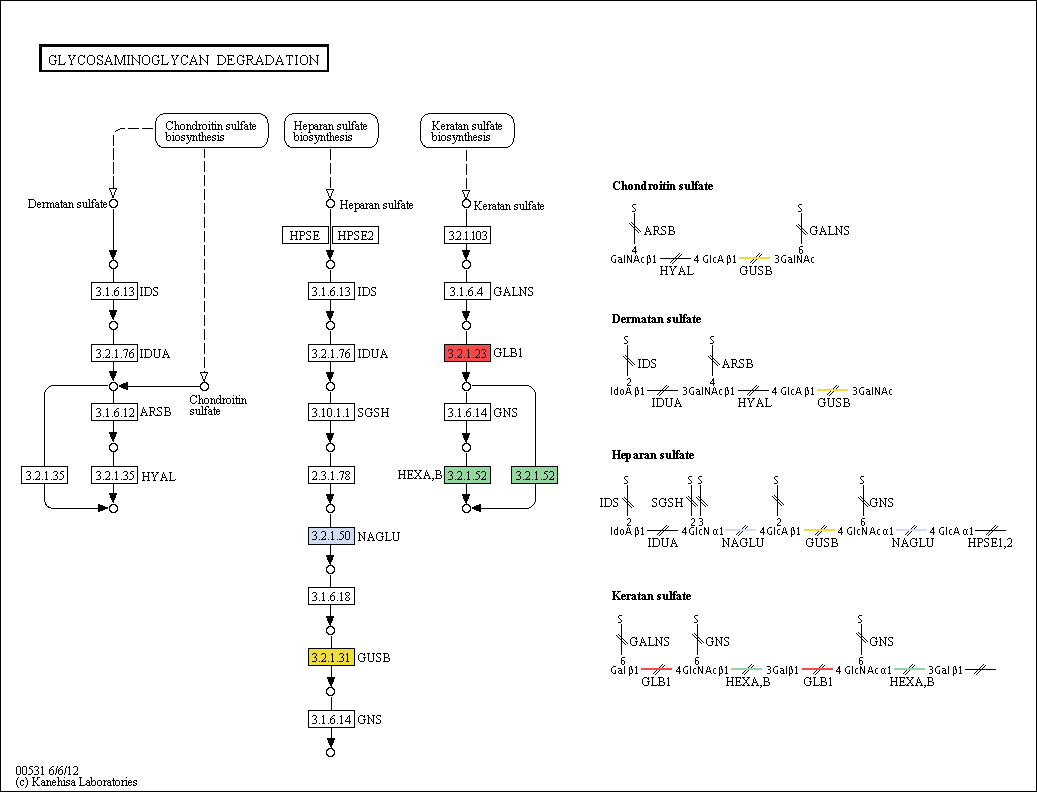

Supplement: Additional file 7: — KEGG pathway annotation. A zip compressed file with a list of KEGGs pathways, graphics in png format, and a file with a comparison with KEGGs pathways of potato and tomato. (ZIP 4361 kb) [file 12864_2016_2656_MOESM7_ESM.zip › Pathway representations/map00531_20150305160436.png]

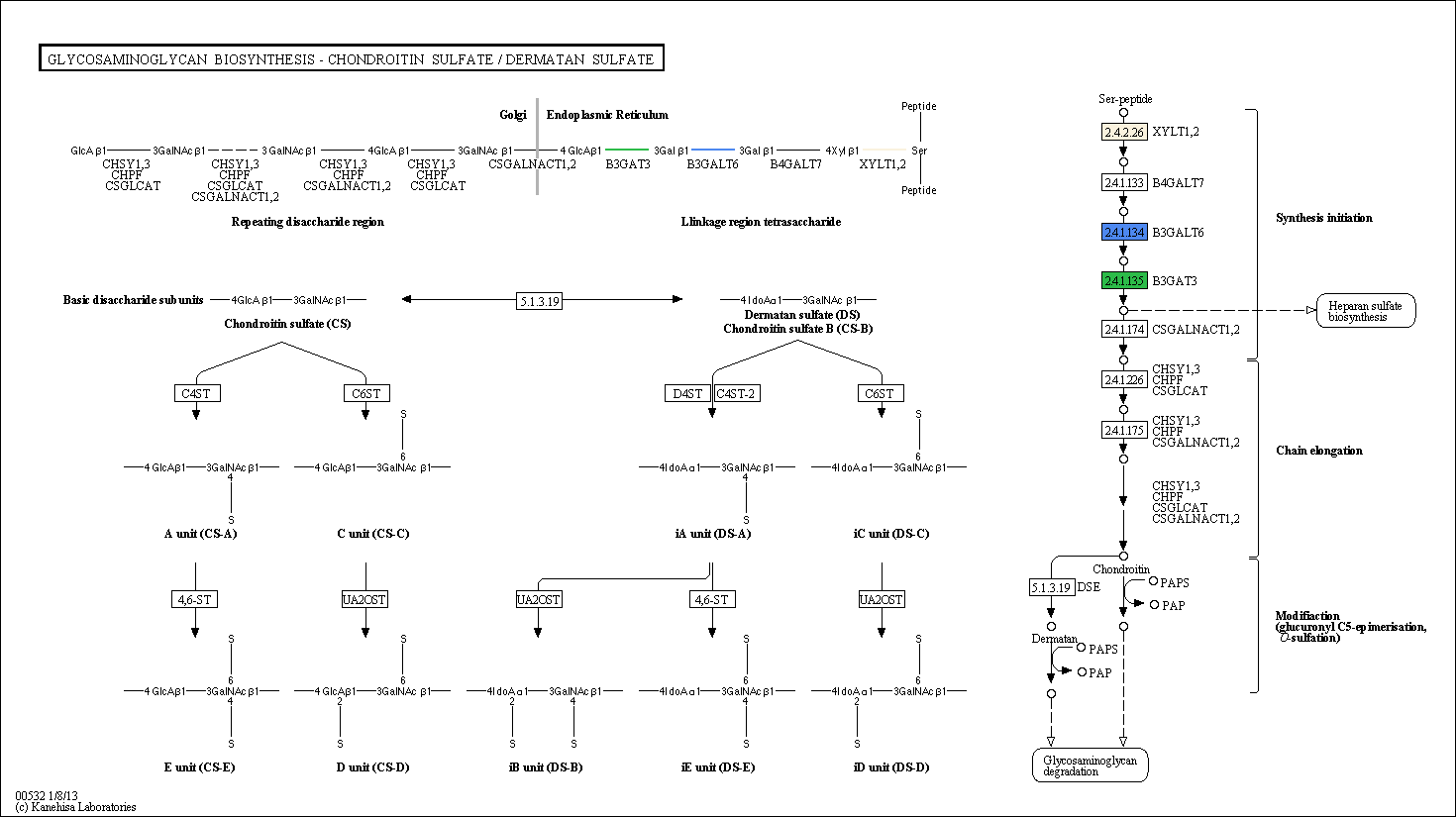

Supplement: Additional file 7: — KEGG pathway annotation. A zip compressed file with a list of KEGGs pathways, graphics in png format, and a file with a comparison with KEGGs pathways of potato and tomato. (ZIP 4361 kb) [file 12864_2016_2656_MOESM7_ESM.zip › Pathway representations/map00532_20150305160433.png]

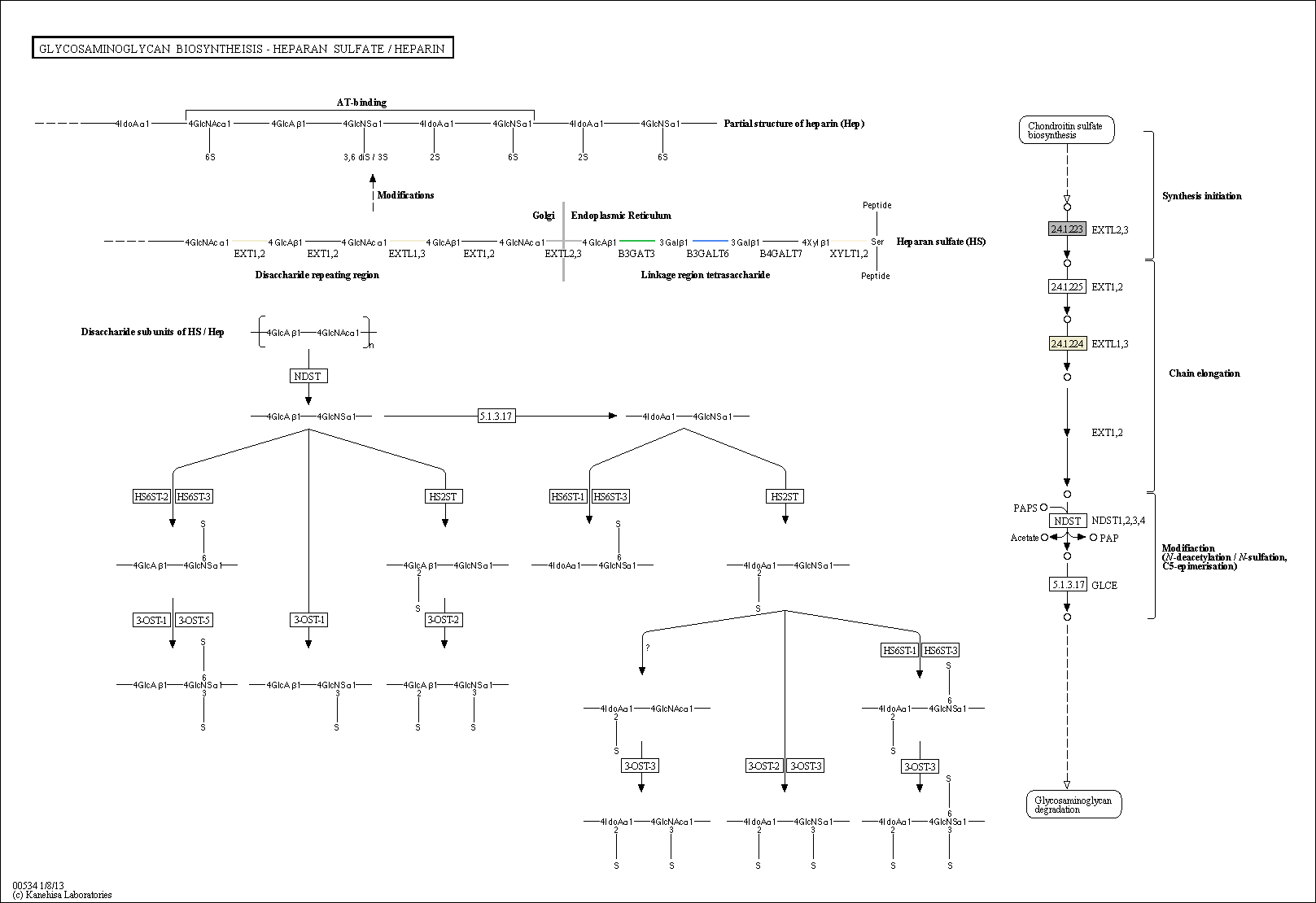

Supplement: Additional file 7: — KEGG pathway annotation. A zip compressed file with a list of KEGGs pathways, graphics in png format, and a file with a comparison with KEGGs pathways of potato and tomato. (ZIP 4361 kb) [file 12864_2016_2656_MOESM7_ESM.zip › Pathway representations/map00534_20150305160430.png]

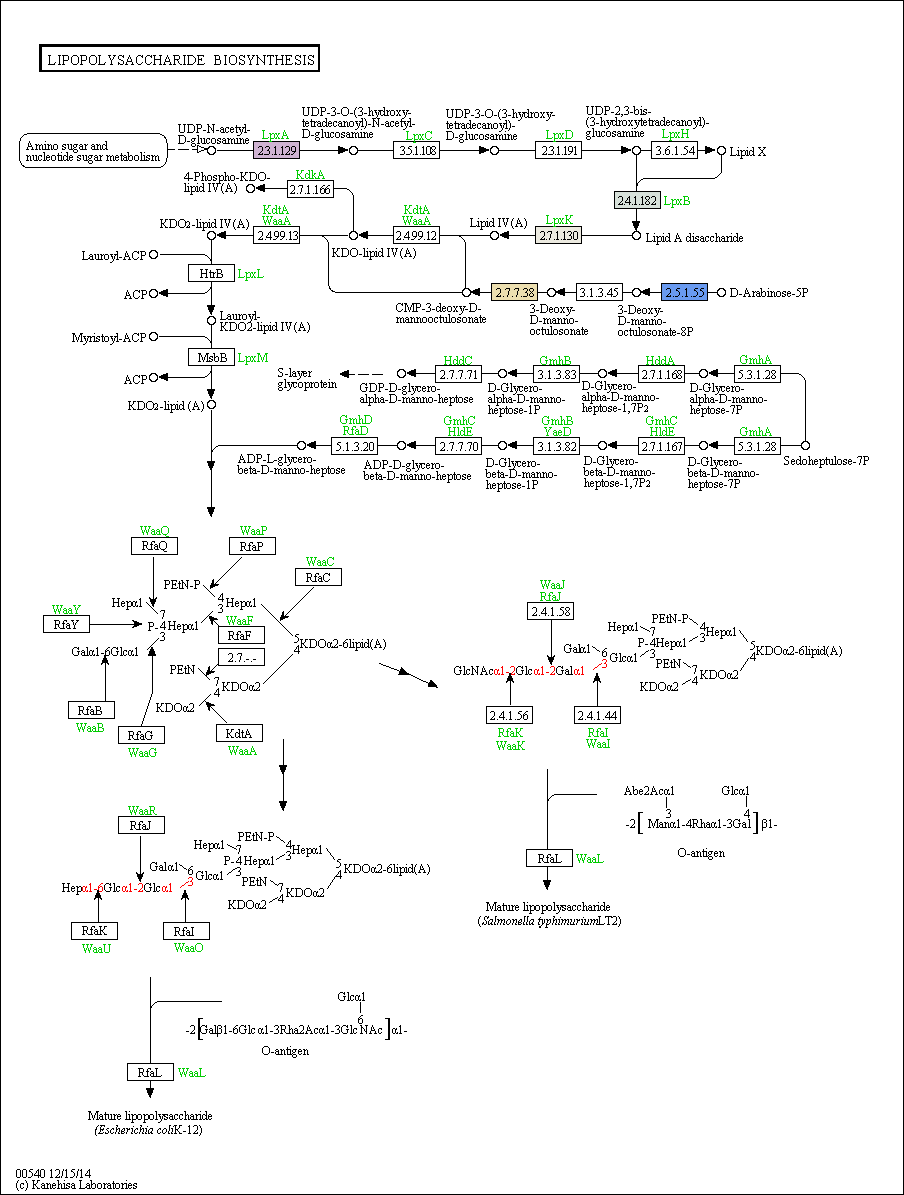

Supplement: Additional file 7: — KEGG pathway annotation. A zip compressed file with a list of KEGGs pathways, graphics in png format, and a file with a comparison with KEGGs pathways of potato and tomato. (ZIP 4361 kb) [file 12864_2016_2656_MOESM7_ESM.zip › Pathway representations/map00540_20150305161626.png]

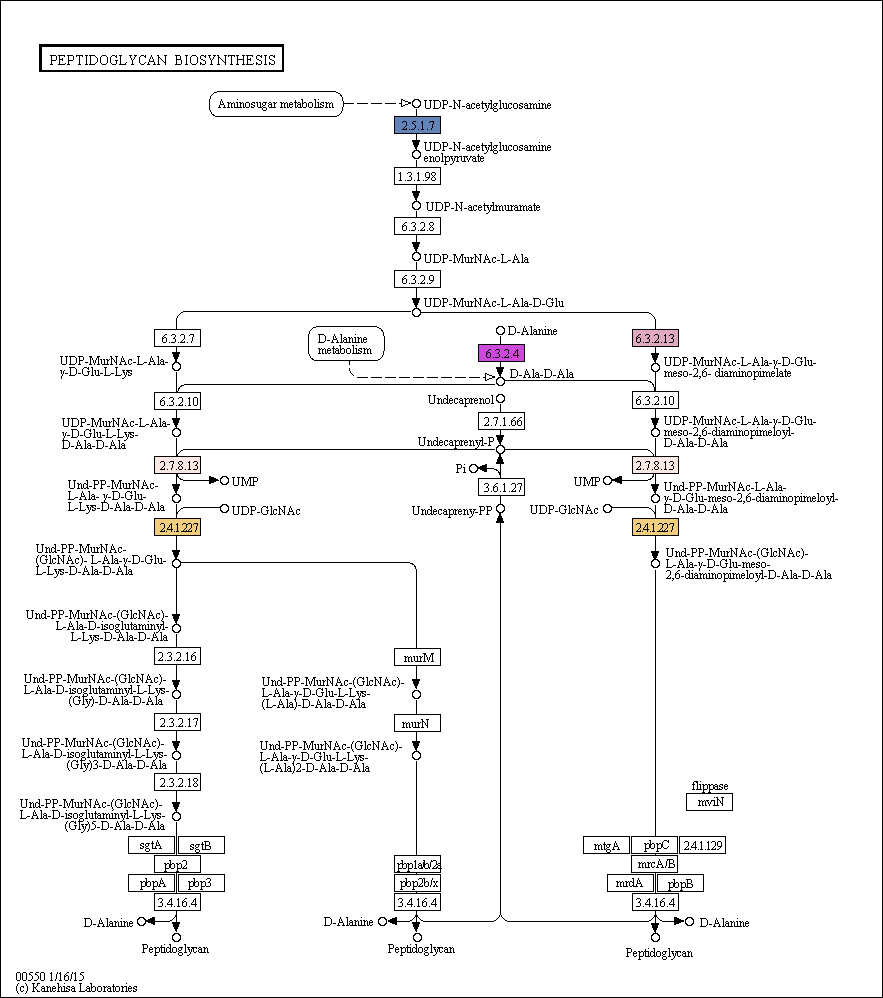

Supplement: Additional file 7: — KEGG pathway annotation. A zip compressed file with a list of KEGGs pathways, graphics in png format, and a file with a comparison with KEGGs pathways of potato and tomato. (ZIP 4361 kb) [file 12864_2016_2656_MOESM7_ESM.zip › Pathway representations/map00550_20150305161543.png]

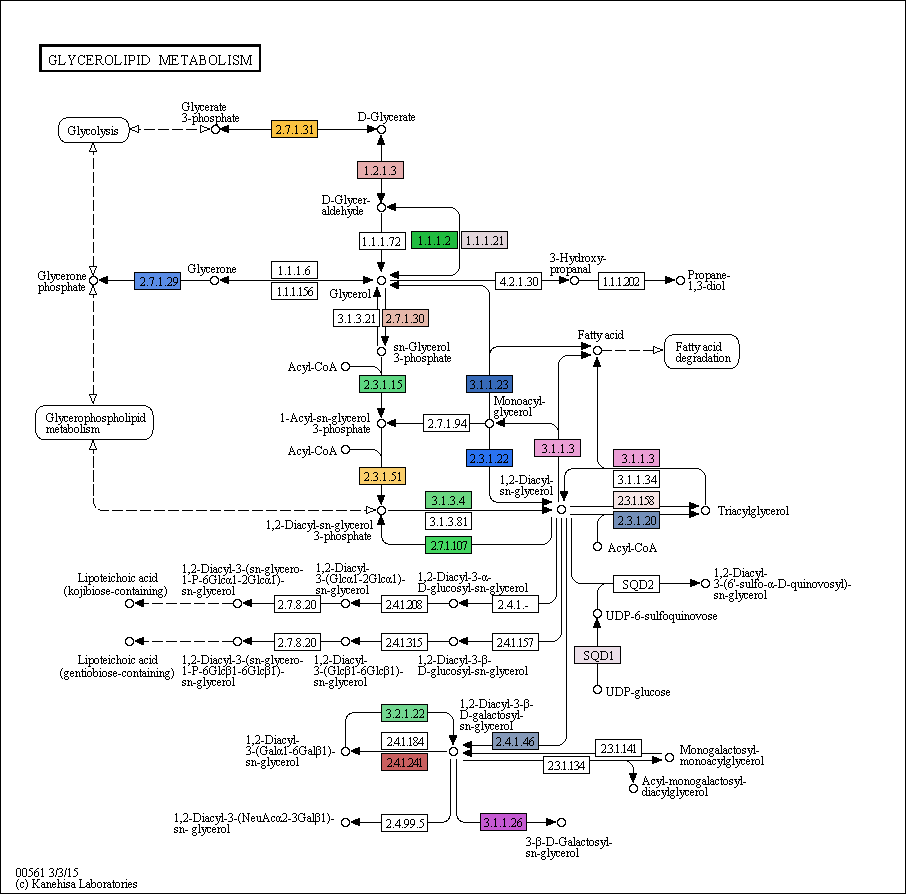

Supplement: Additional file 7: — KEGG pathway annotation. A zip compressed file with a list of KEGGs pathways, graphics in png format, and a file with a comparison with KEGGs pathways of potato and tomato. (ZIP 4361 kb) [file 12864_2016_2656_MOESM7_ESM.zip › Pathway representations/map00561_20150305161504.png]

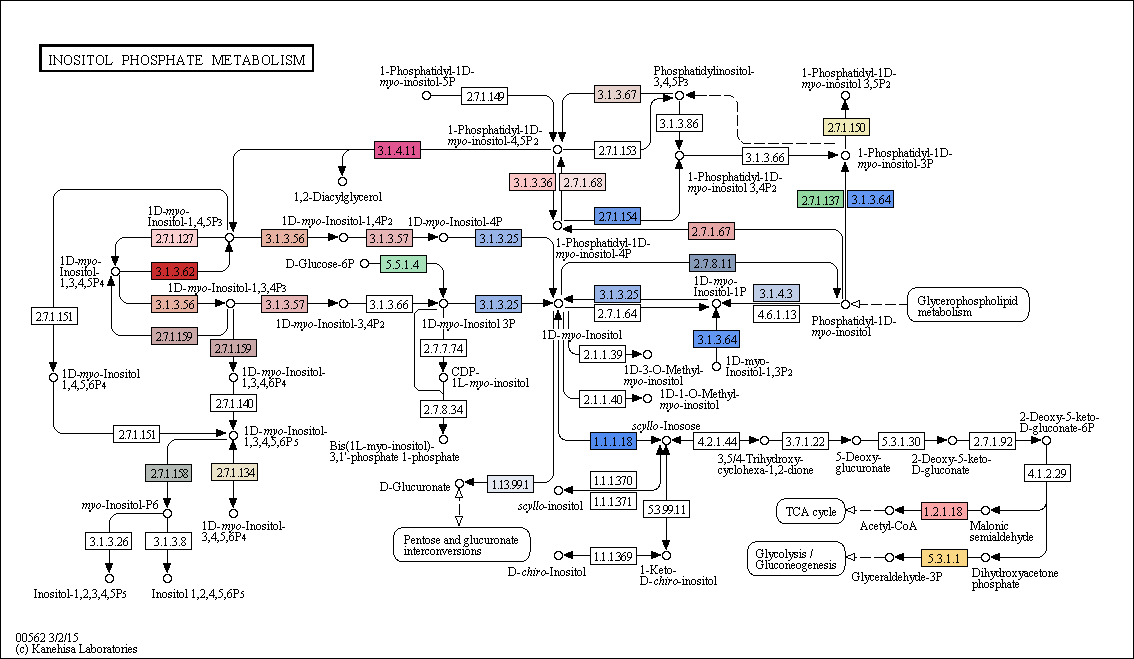

Supplement: Additional file 7: — KEGG pathway annotation. A zip compressed file with a list of KEGGs pathways, graphics in png format, and a file with a comparison with KEGGs pathways of potato and tomato. (ZIP 4361 kb) [file 12864_2016_2656_MOESM7_ESM.zip › Pathway representations/map00562_20150305161458.png]

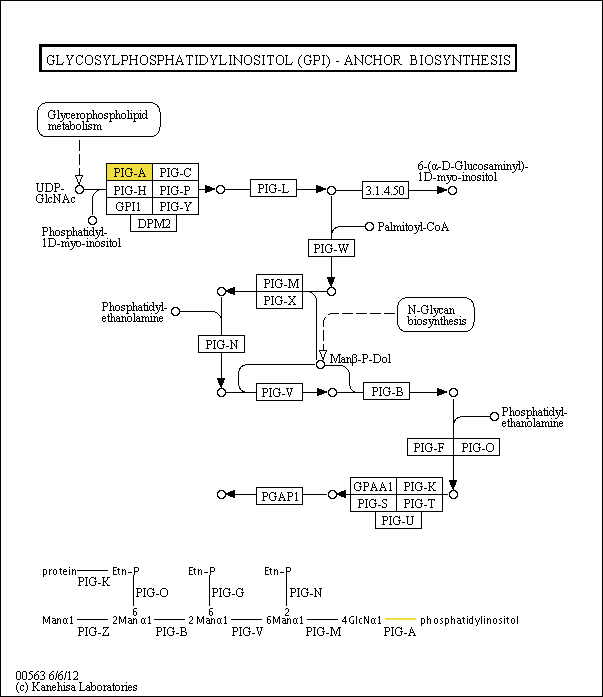

Supplement: Additional file 7: — KEGG pathway annotation. A zip compressed file with a list of KEGGs pathways, graphics in png format, and a file with a comparison with KEGGs pathways of potato and tomato. (ZIP 4361 kb) [file 12864_2016_2656_MOESM7_ESM.zip › Pathway representations/map00563_20150305161444.png]

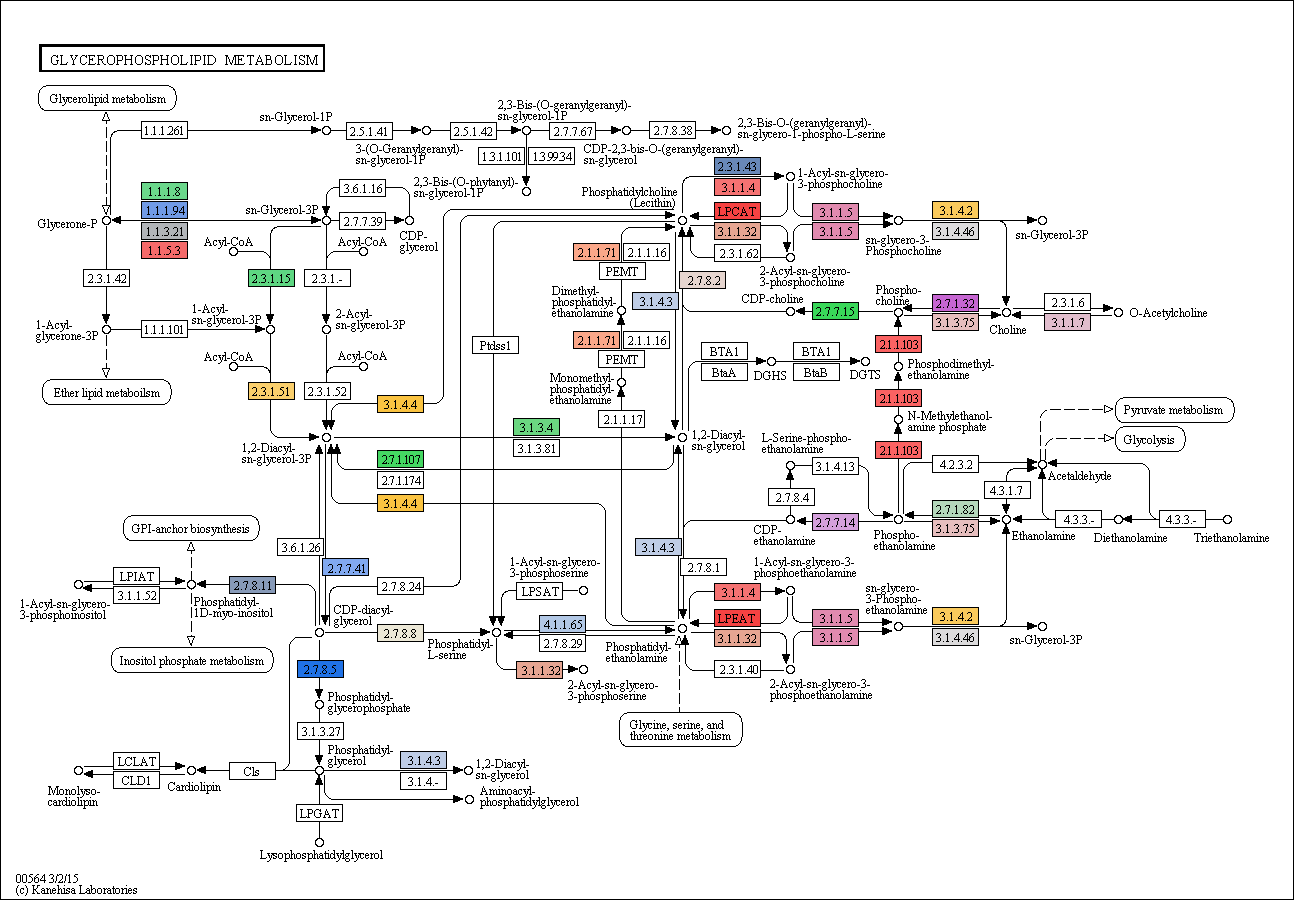

Supplement: Additional file 7: — KEGG pathway annotation. A zip compressed file with a list of KEGGs pathways, graphics in png format, and a file with a comparison with KEGGs pathways of potato and tomato. (ZIP 4361 kb) [file 12864_2016_2656_MOESM7_ESM.zip › Pathway representations/map00564_20150305161437.png]

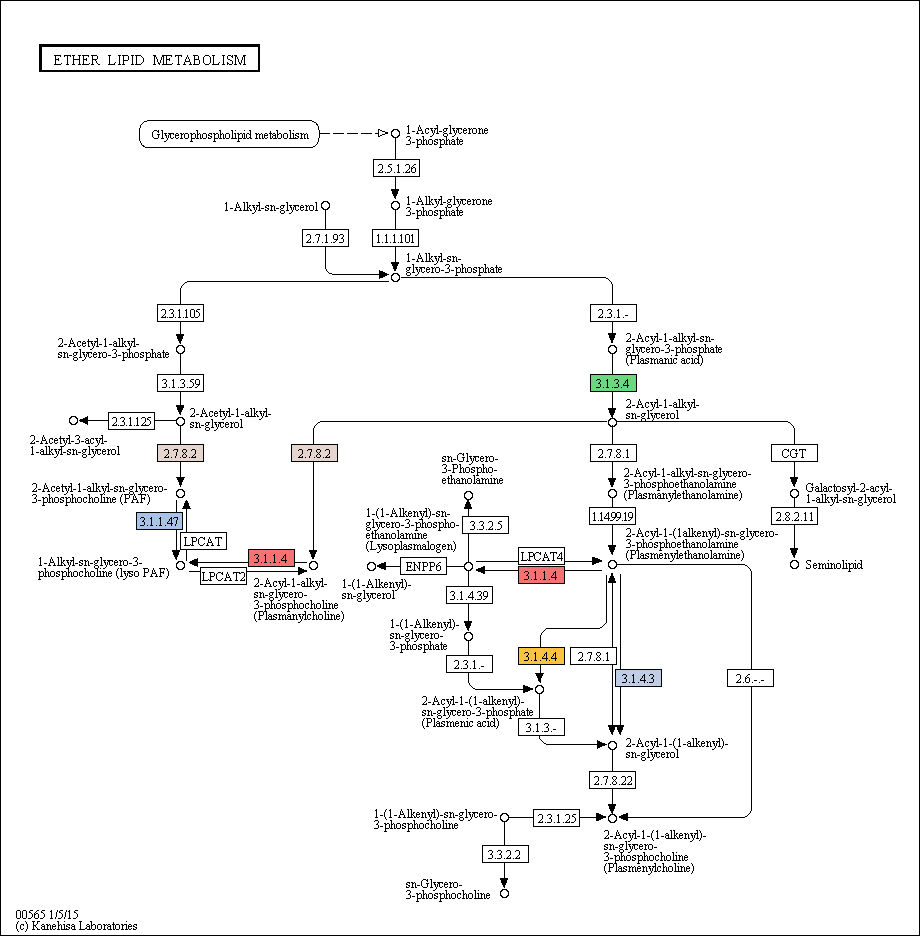

Supplement: Additional file 7: — KEGG pathway annotation. A zip compressed file with a list of KEGGs pathways, graphics in png format, and a file with a comparison with KEGGs pathways of potato and tomato. (ZIP 4361 kb) [file 12864_2016_2656_MOESM7_ESM.zip › Pathway representations/map00565_20150305161426.png]

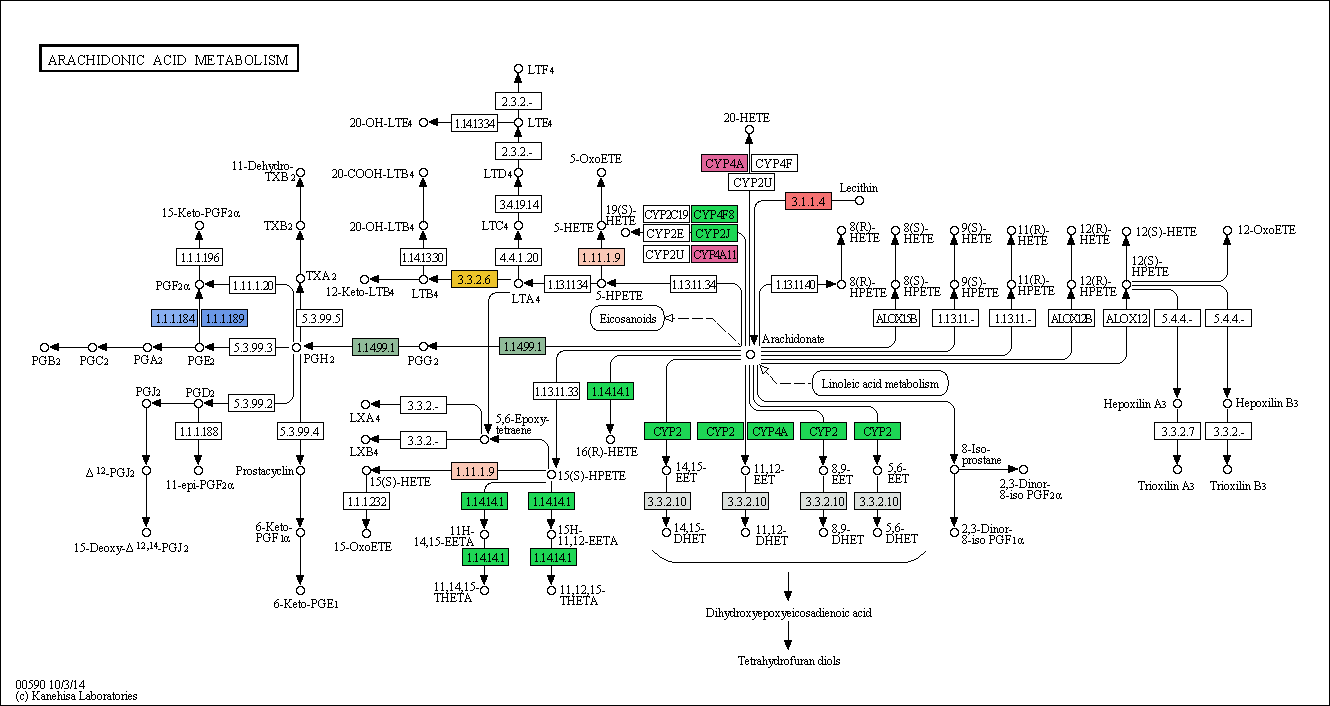

Supplement: Additional file 7: — KEGG pathway annotation. A zip compressed file with a list of KEGGs pathways, graphics in png format, and a file with a comparison with KEGGs pathways of potato and tomato. (ZIP 4361 kb) [file 12864_2016_2656_MOESM7_ESM.zip › Pathway representations/map00590_20150305161125.png]

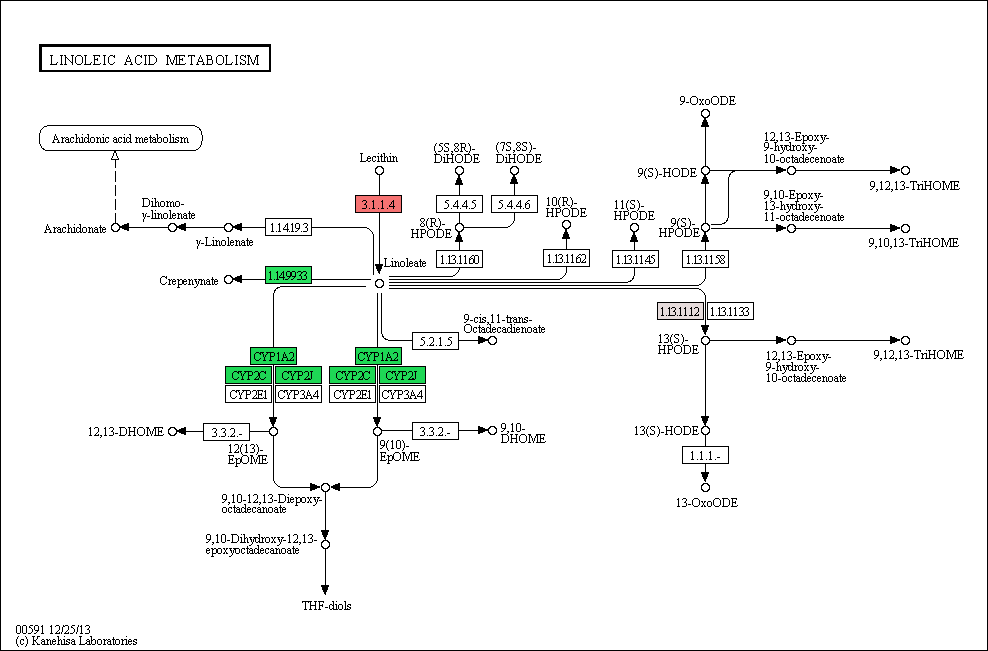

Supplement: Additional file 7: — KEGG pathway annotation. A zip compressed file with a list of KEGGs pathways, graphics in png format, and a file with a comparison with KEGGs pathways of potato and tomato. (ZIP 4361 kb) [file 12864_2016_2656_MOESM7_ESM.zip › Pathway representations/map00591_20150305161115.png]

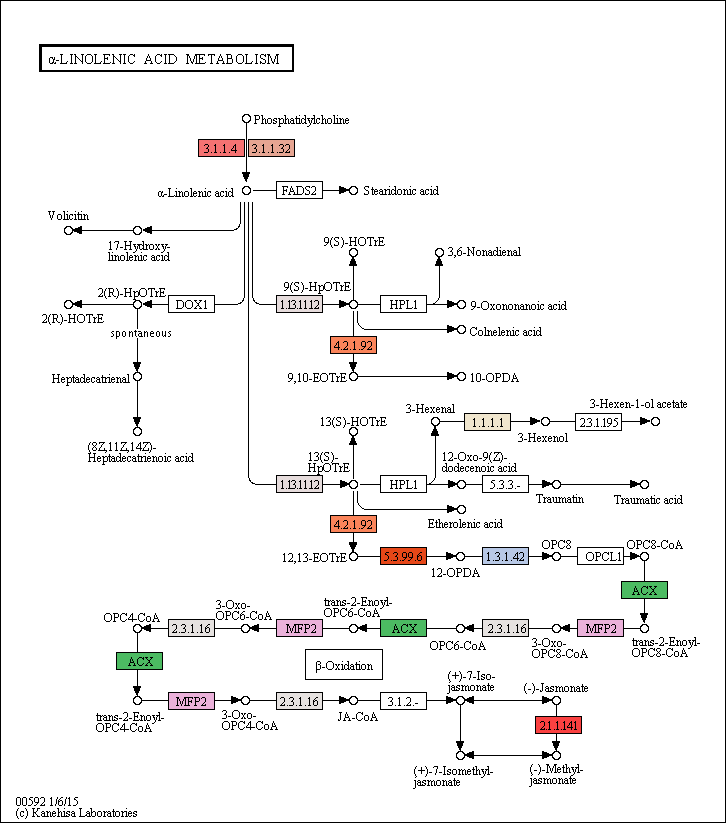

Supplement: Additional file 7: — KEGG pathway annotation. A zip compressed file with a list of KEGGs pathways, graphics in png format, and a file with a comparison with KEGGs pathways of potato and tomato. (ZIP 4361 kb) [file 12864_2016_2656_MOESM7_ESM.zip › Pathway representations/map00592_20150305161112.png]

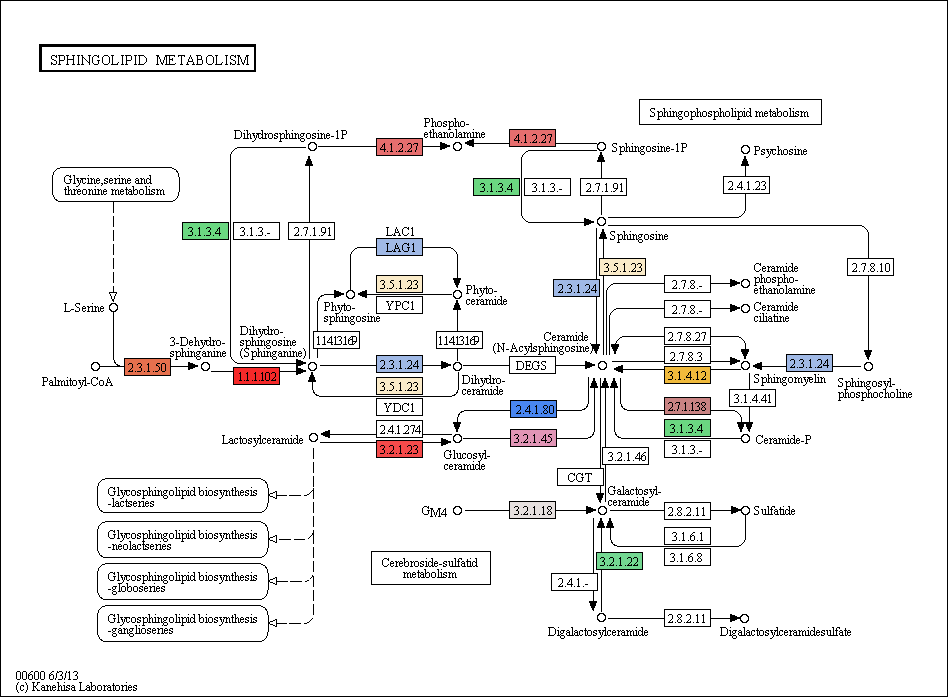

Supplement: Additional file 7: — KEGG pathway annotation. A zip compressed file with a list of KEGGs pathways, graphics in png format, and a file with a comparison with KEGGs pathways of potato and tomato. (ZIP 4361 kb) [file 12864_2016_2656_MOESM7_ESM.zip › Pathway representations/map00600_20150305161414.png]

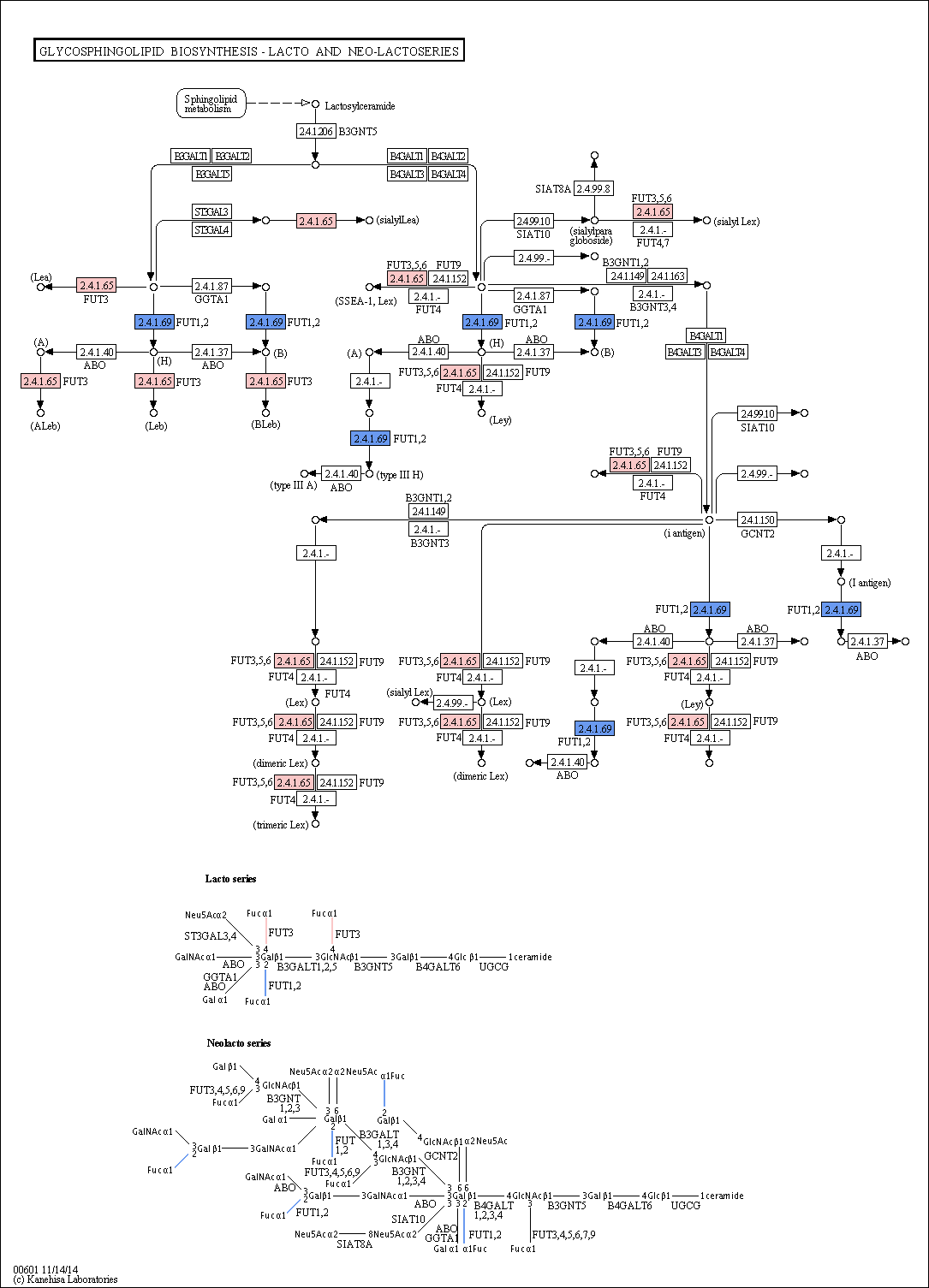

Supplement: Additional file 7: — KEGG pathway annotation. A zip compressed file with a list of KEGGs pathways, graphics in png format, and a file with a comparison with KEGGs pathways of potato and tomato. (ZIP 4361 kb) [file 12864_2016_2656_MOESM7_ESM.zip › Pathway representations/map00601_20150305161409.png]

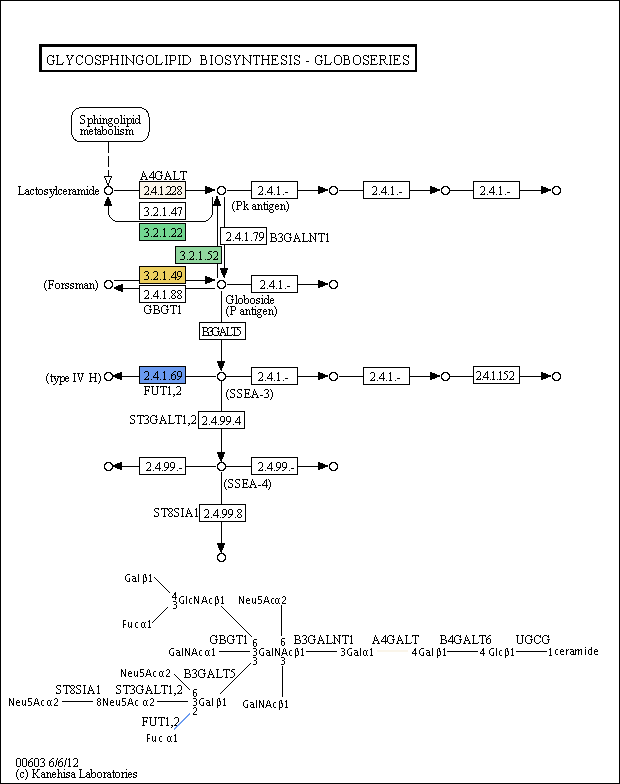

Supplement: Additional file 7: — KEGG pathway annotation. A zip compressed file with a list of KEGGs pathways, graphics in png format, and a file with a comparison with KEGGs pathways of potato and tomato. (ZIP 4361 kb) [file 12864_2016_2656_MOESM7_ESM.zip › Pathway representations/map00603_20150305161405.png]

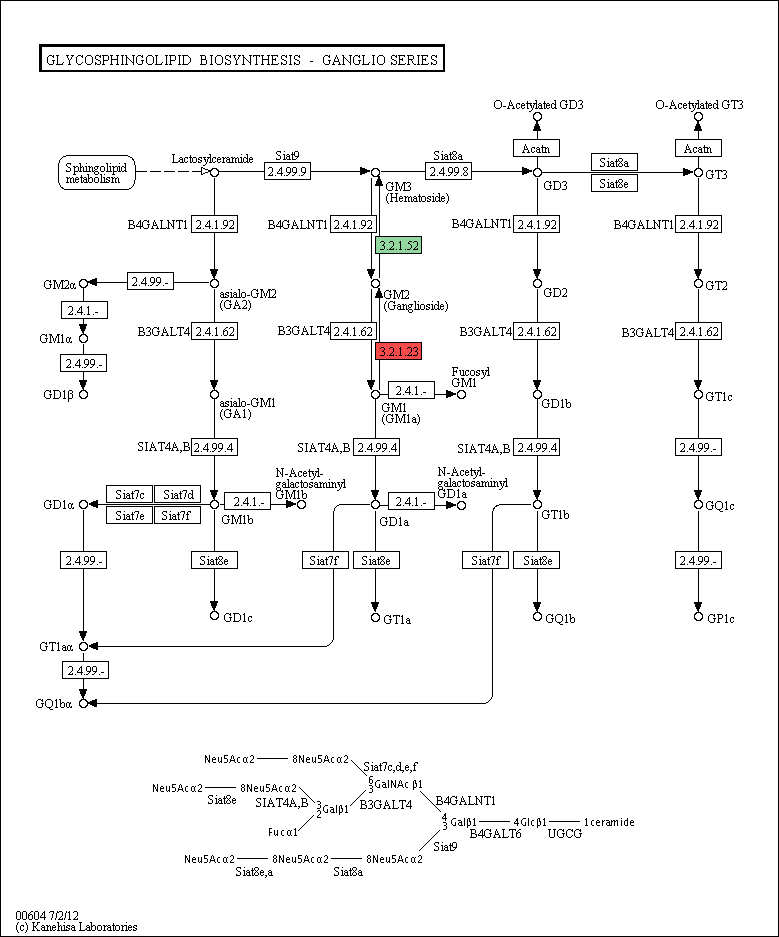

Supplement: Additional file 7: — KEGG pathway annotation. A zip compressed file with a list of KEGGs pathways, graphics in png format, and a file with a comparison with KEGGs pathways of potato and tomato. (ZIP 4361 kb) [file 12864_2016_2656_MOESM7_ESM.zip › Pathway representations/map00604_20150305161402.png]

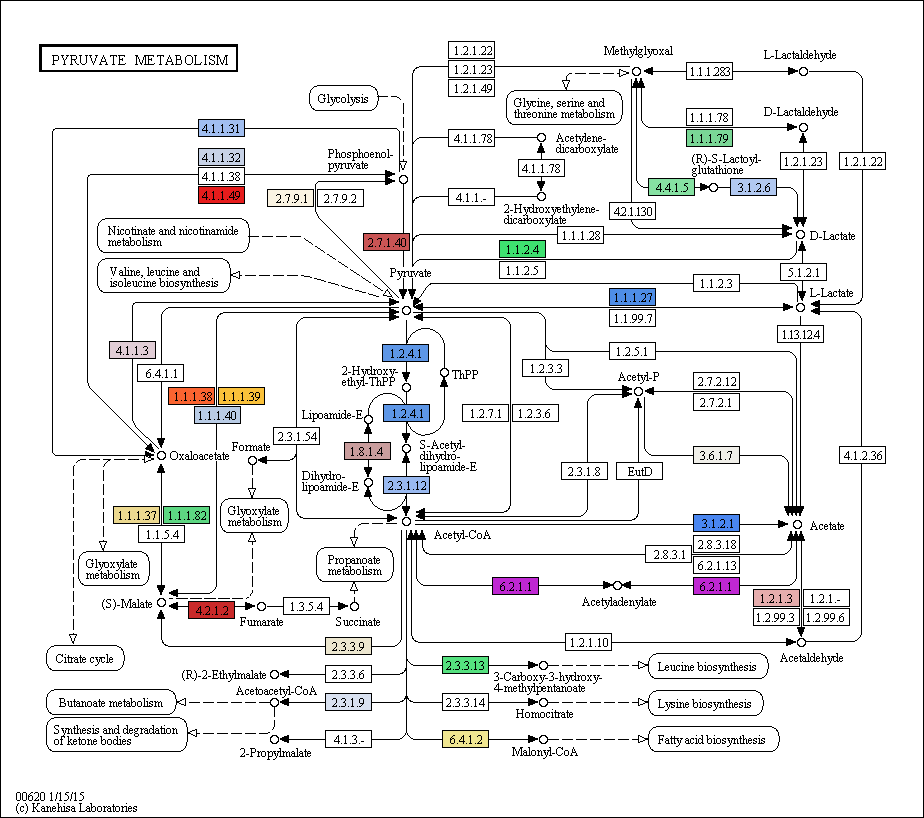

Supplement: Additional file 7: — KEGG pathway annotation. A zip compressed file with a list of KEGGs pathways, graphics in png format, and a file with a comparison with KEGGs pathways of potato and tomato. (ZIP 4361 kb) [file 12864_2016_2656_MOESM7_ESM.zip › Pathway representations/map00620_20150305161250.png]

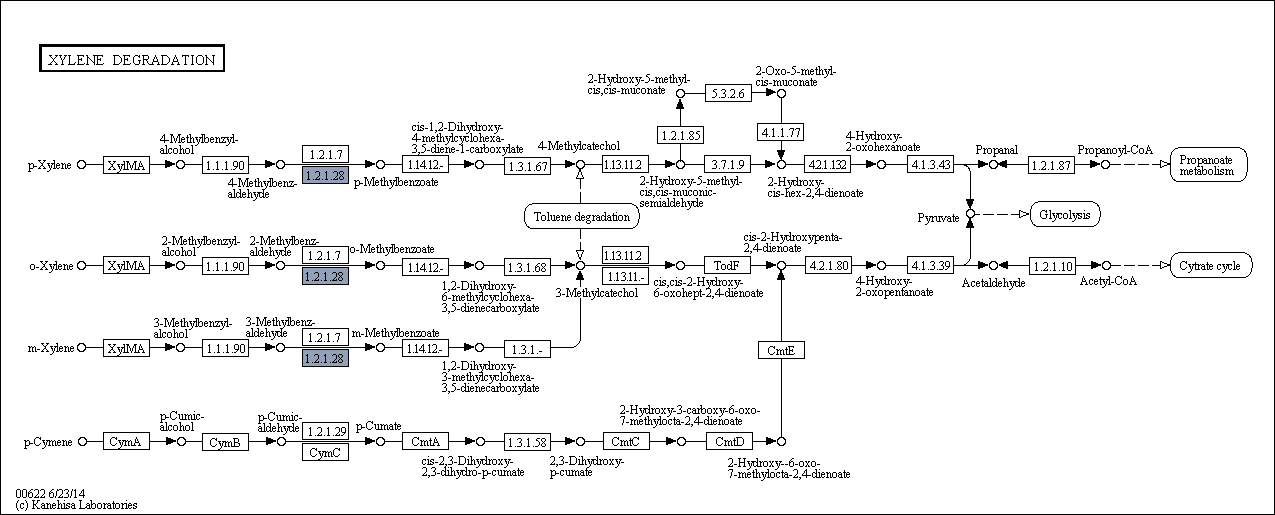

Supplement: Additional file 7: — KEGG pathway annotation. A zip compressed file with a list of KEGGs pathways, graphics in png format, and a file with a comparison with KEGGs pathways of potato and tomato. (ZIP 4361 kb) [file 12864_2016_2656_MOESM7_ESM.zip › Pathway representations/map00622_20150305161243.png]

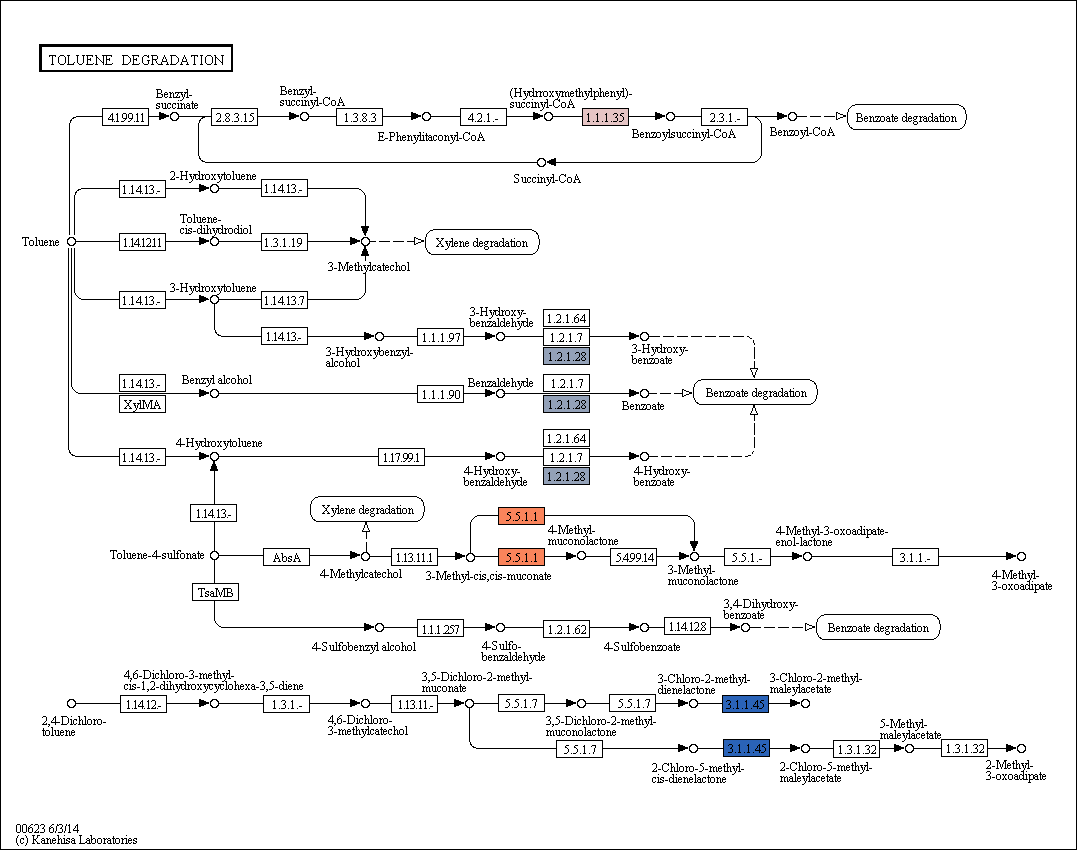

Supplement: Additional file 7: — KEGG pathway annotation. A zip compressed file with a list of KEGGs pathways, graphics in png format, and a file with a comparison with KEGGs pathways of potato and tomato. (ZIP 4361 kb) [file 12864_2016_2656_MOESM7_ESM.zip › Pathway representations/map00623_20150305161237.png]

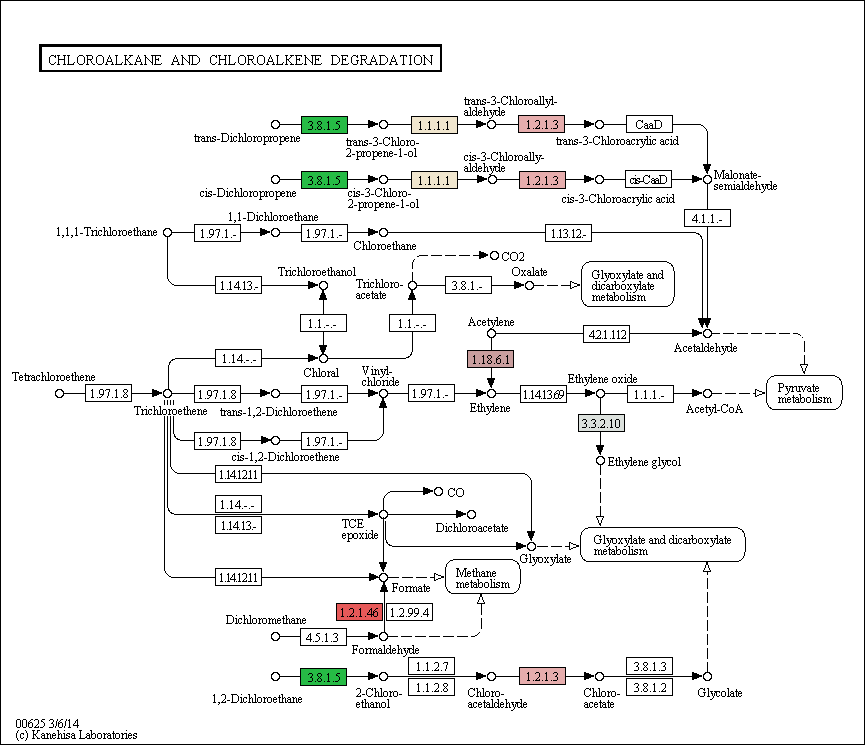

Supplement: Additional file 7: — KEGG pathway annotation. A zip compressed file with a list of KEGGs pathways, graphics in png format, and a file with a comparison with KEGGs pathways of potato and tomato. (ZIP 4361 kb) [file 12864_2016_2656_MOESM7_ESM.zip › Pathway representations/map00625_20150305161227.png]

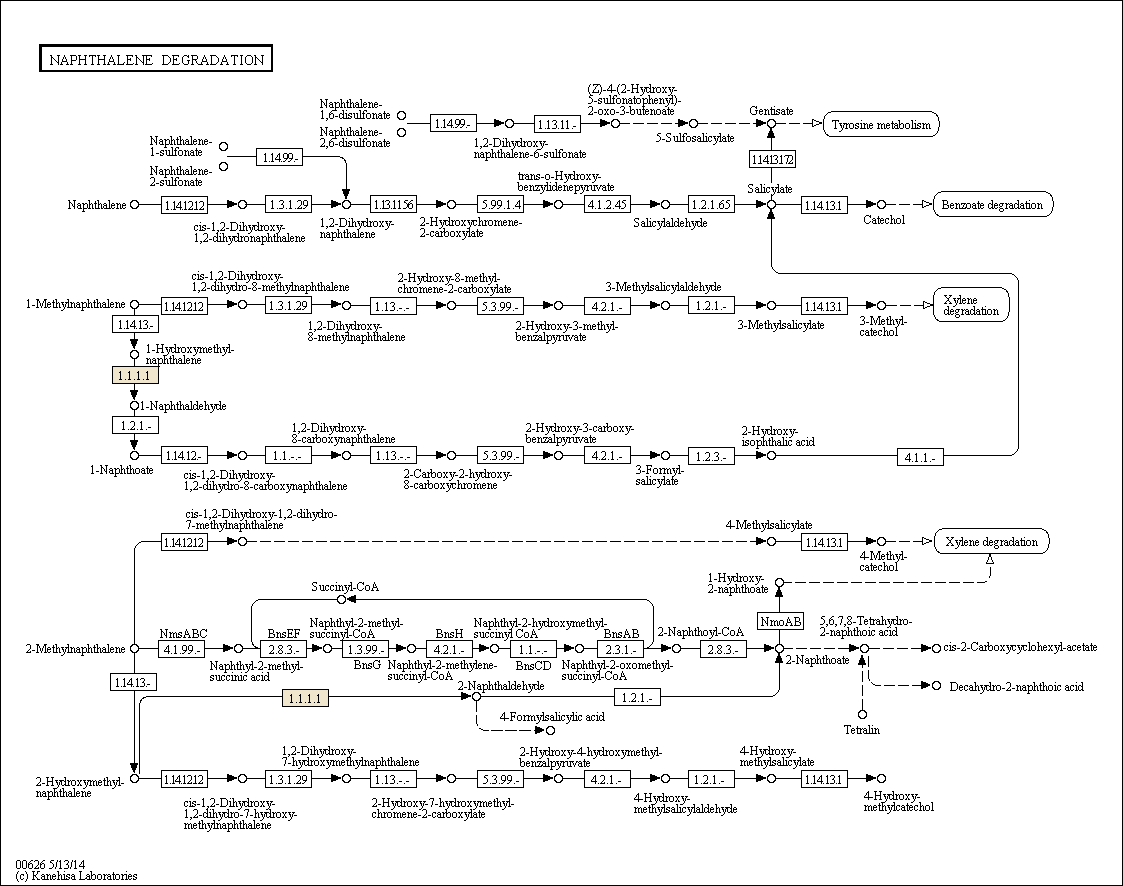

Supplement: Additional file 7: — KEGG pathway annotation. A zip compressed file with a list of KEGGs pathways, graphics in png format, and a file with a comparison with KEGGs pathways of potato and tomato. (ZIP 4361 kb) [file 12864_2016_2656_MOESM7_ESM.zip › Pathway representations/map00626_20150305161220.png]

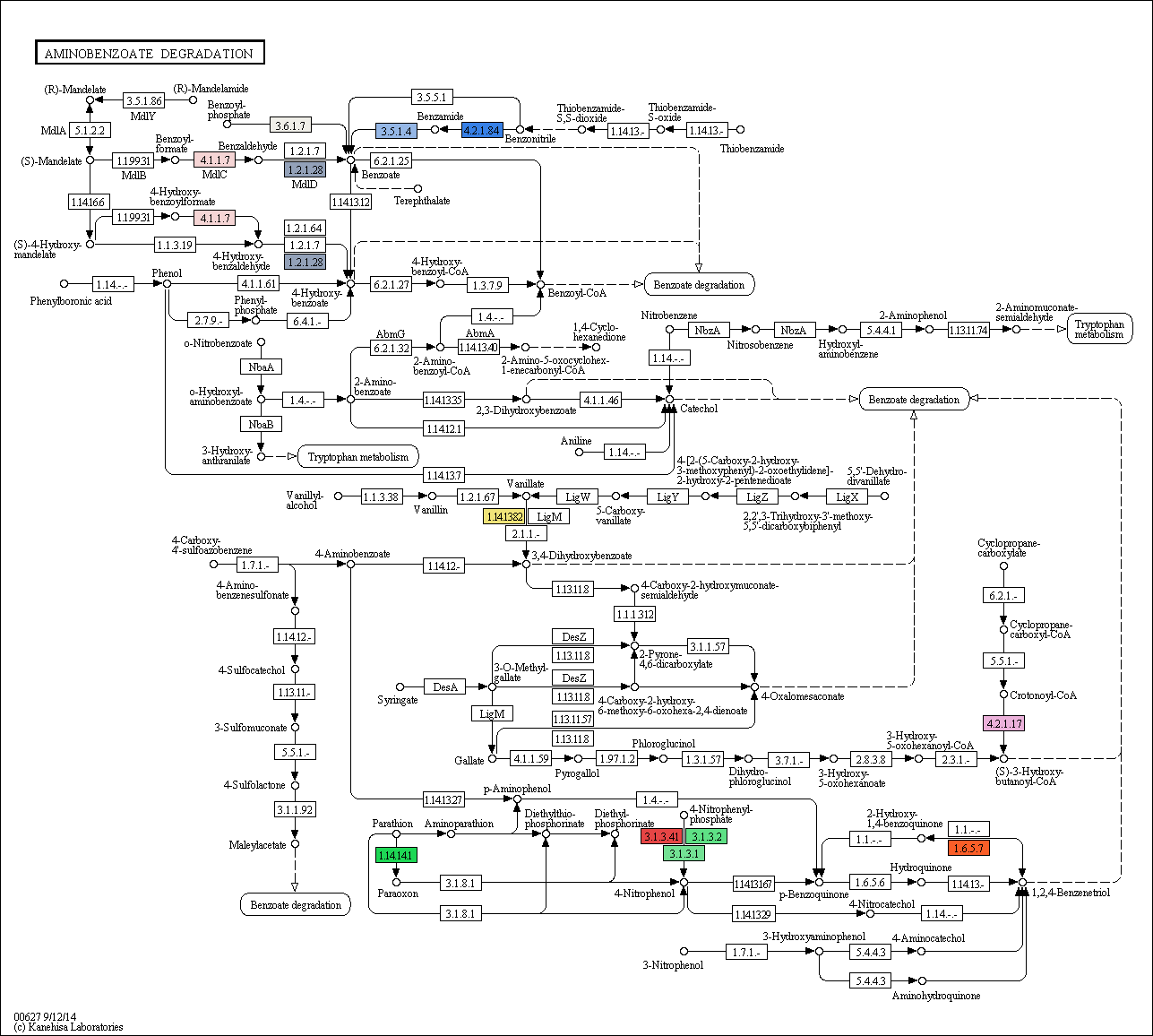

Supplement: Additional file 7: — KEGG pathway annotation. A zip compressed file with a list of KEGGs pathways, graphics in png format, and a file with a comparison with KEGGs pathways of potato and tomato. (ZIP 4361 kb) [file 12864_2016_2656_MOESM7_ESM.zip › Pathway representations/map00627_20150305161211.png]

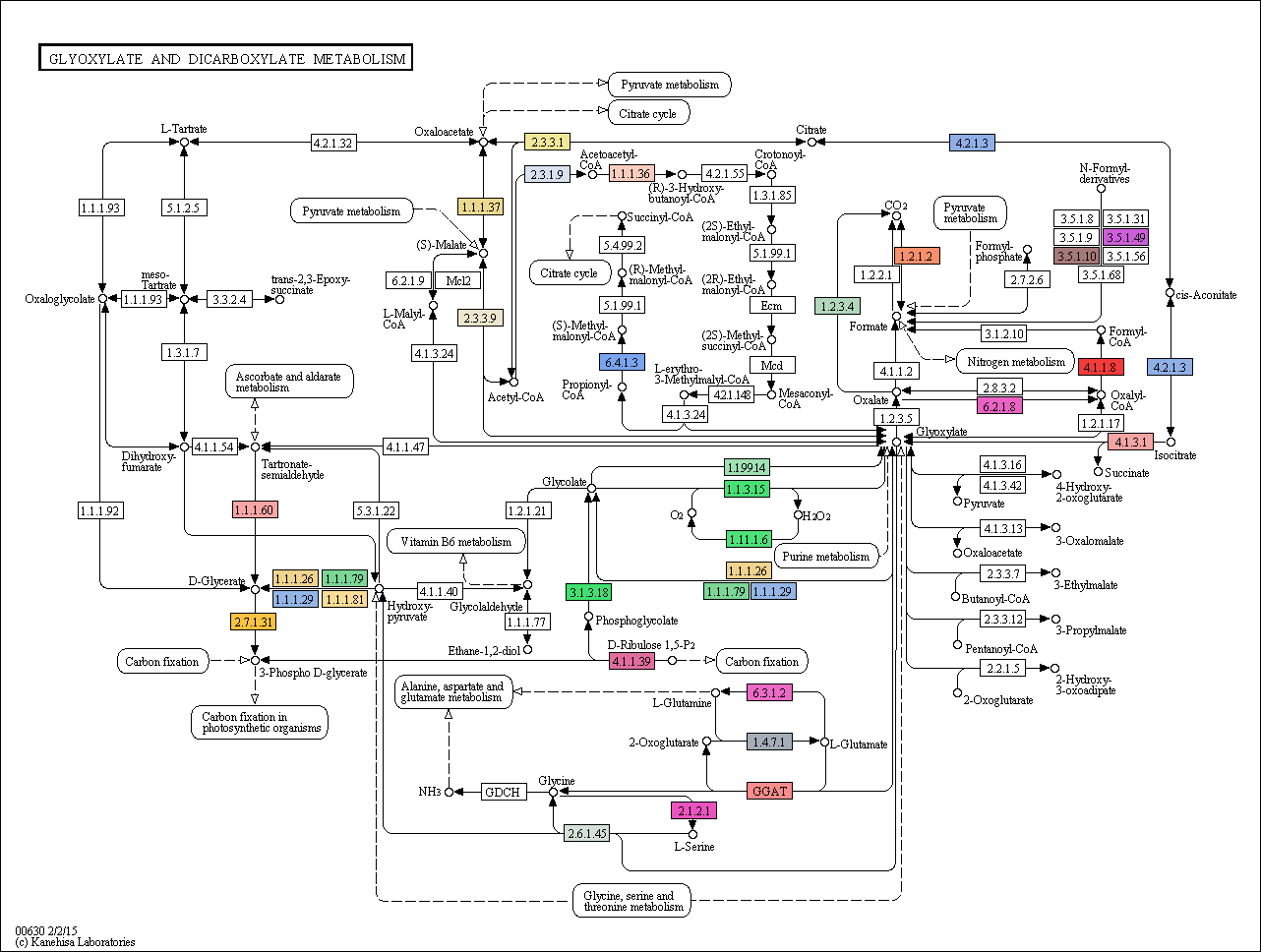

Supplement: Additional file 7: — KEGG pathway annotation. A zip compressed file with a list of KEGGs pathways, graphics in png format, and a file with a comparison with KEGGs pathways of potato and tomato. (ZIP 4361 kb) [file 12864_2016_2656_MOESM7_ESM.zip › Pathway representations/map00630_20150305161056.png]

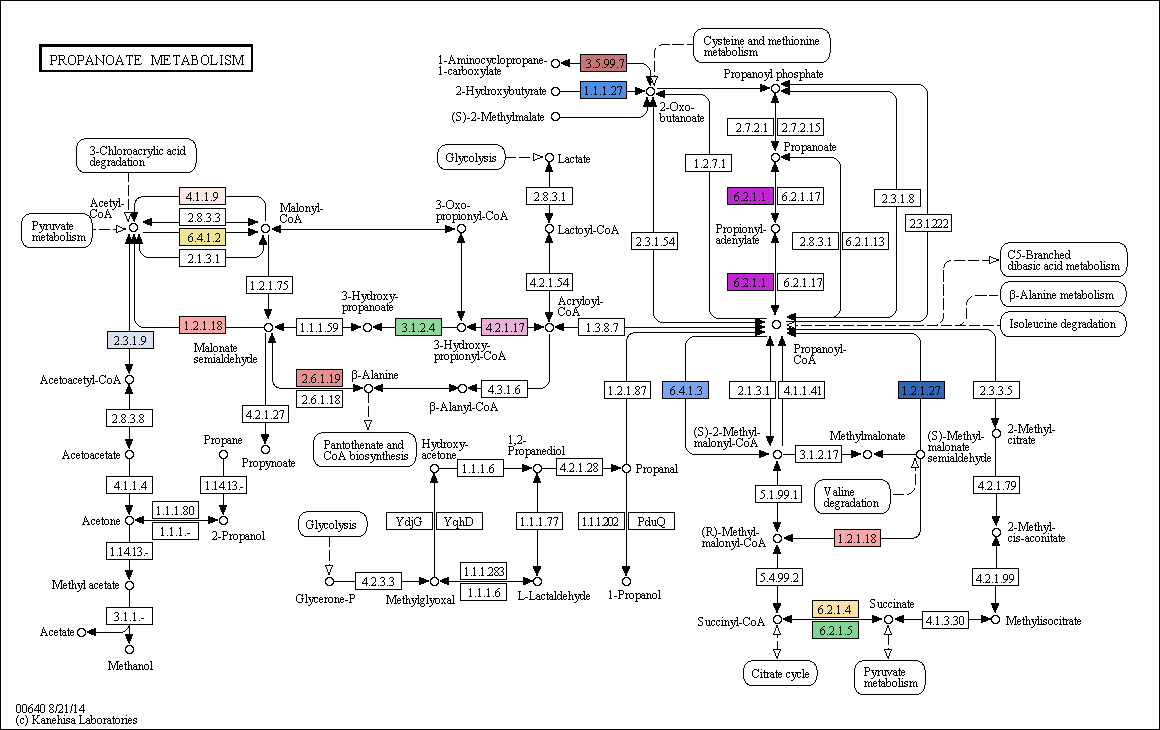

Supplement: Additional file 7: — KEGG pathway annotation. A zip compressed file with a list of KEGGs pathways, graphics in png format, and a file with a comparison with KEGGs pathways of potato and tomato. (ZIP 4361 kb) [file 12864_2016_2656_MOESM7_ESM.zip › Pathway representations/map00640_20150305161021.png]

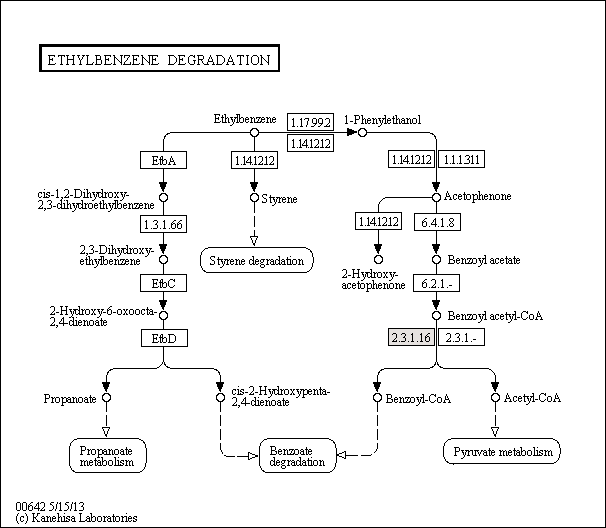

Supplement: Additional file 7: — KEGG pathway annotation. A zip compressed file with a list of KEGGs pathways, graphics in png format, and a file with a comparison with KEGGs pathways of potato and tomato. (ZIP 4361 kb) [file 12864_2016_2656_MOESM7_ESM.zip › Pathway representations/map00642_20150305161015.png]

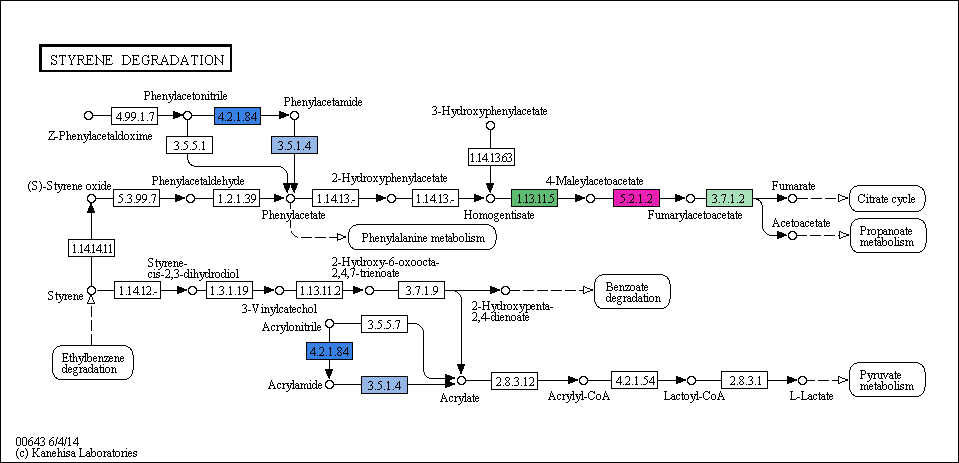

Supplement: Additional file 7: — KEGG pathway annotation. A zip compressed file with a list of KEGGs pathways, graphics in png format, and a file with a comparison with KEGGs pathways of potato and tomato. (ZIP 4361 kb) [file 12864_2016_2656_MOESM7_ESM.zip › Pathway representations/map00643_20150305161013.png]

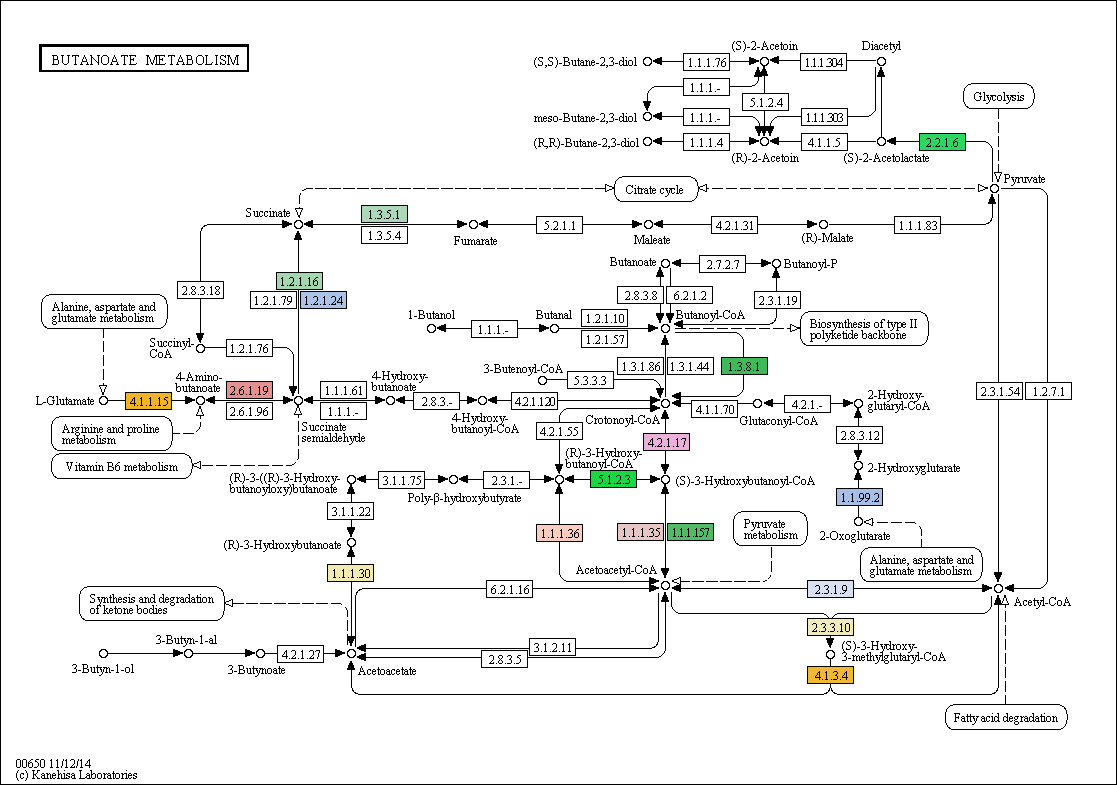

Supplement: Additional file 7: — KEGG pathway annotation. A zip compressed file with a list of KEGGs pathways, graphics in png format, and a file with a comparison with KEGGs pathways of potato and tomato. (ZIP 4361 kb) [file 12864_2016_2656_MOESM7_ESM.zip › Pathway representations/map00650_20150305160931.png]

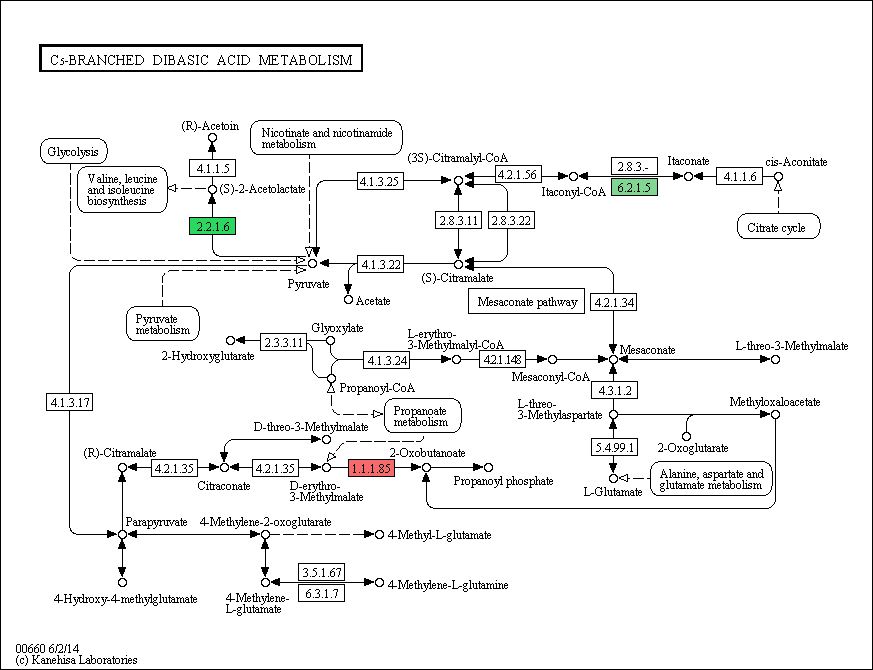

Supplement: Additional file 7: — KEGG pathway annotation. A zip compressed file with a list of KEGGs pathways, graphics in png format, and a file with a comparison with KEGGs pathways of potato and tomato. (ZIP 4361 kb) [file 12864_2016_2656_MOESM7_ESM.zip › Pathway representations/map00660_20150305160848.png]

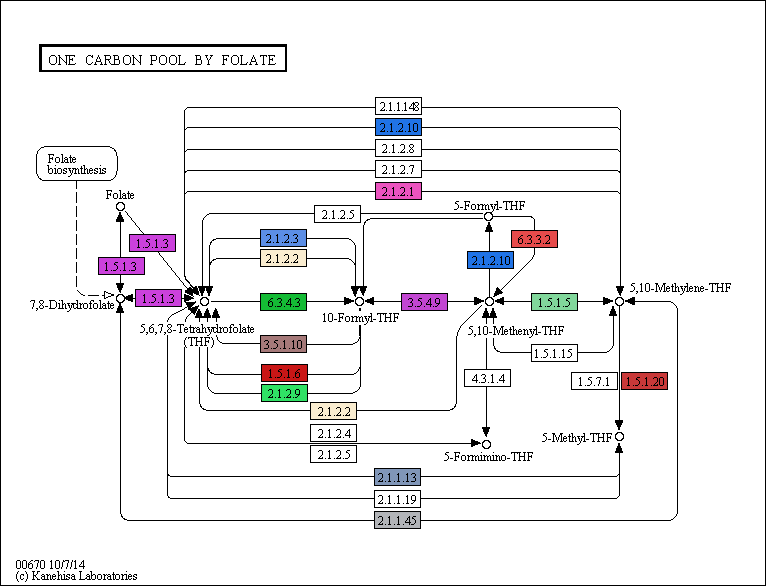

Supplement: Additional file 7: — KEGG pathway annotation. A zip compressed file with a list of KEGGs pathways, graphics in png format, and a file with a comparison with KEGGs pathways of potato and tomato. (ZIP 4361 kb) [file 12864_2016_2656_MOESM7_ESM.zip › Pathway representations/map00670_20150305160702.png]

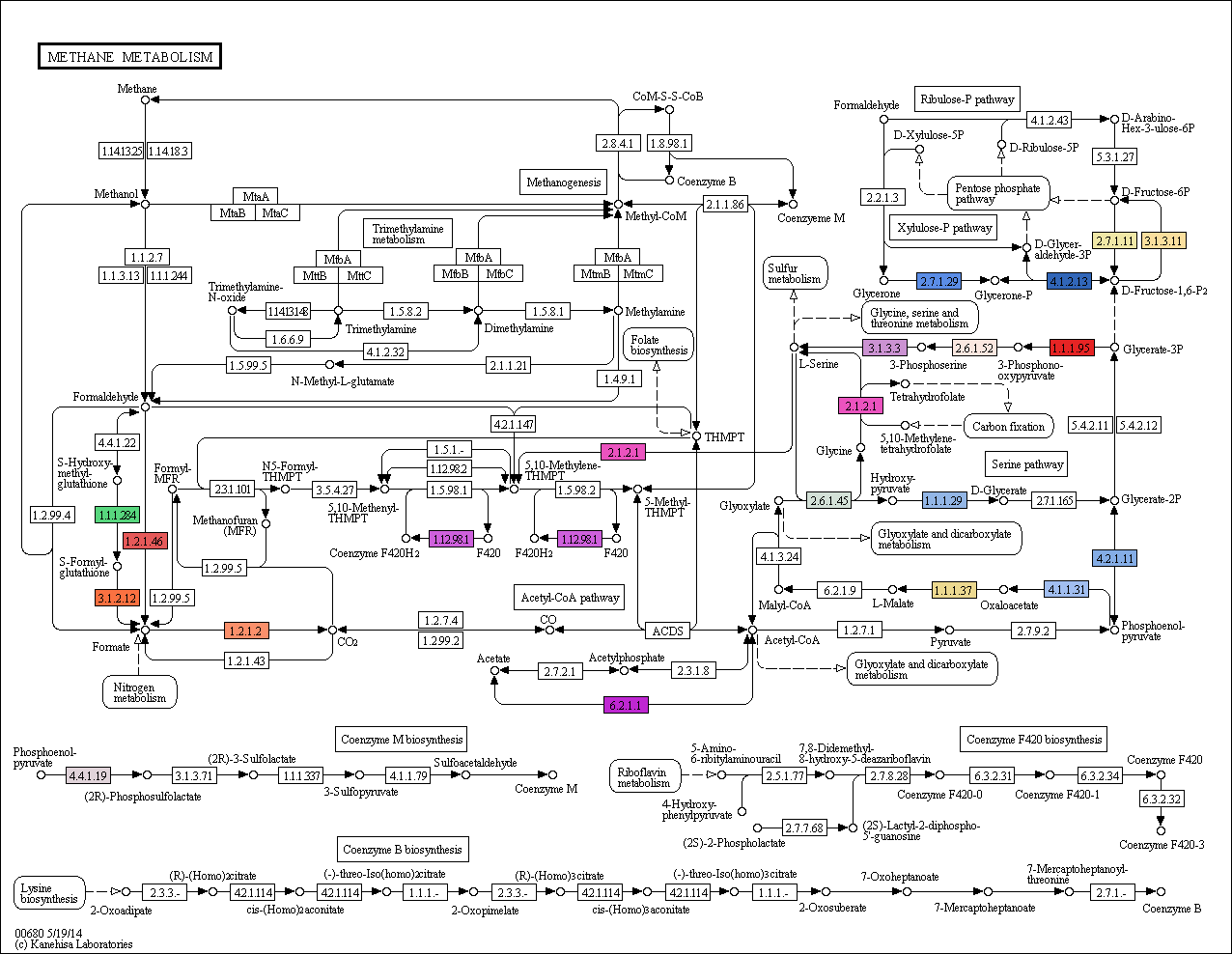

Supplement: Additional file 7: — KEGG pathway annotation. A zip compressed file with a list of KEGGs pathways, graphics in png format, and a file with a comparison with KEGGs pathways of potato and tomato. (ZIP 4361 kb) [file 12864_2016_2656_MOESM7_ESM.zip › Pathway representations/map00680_20150305160555.png]

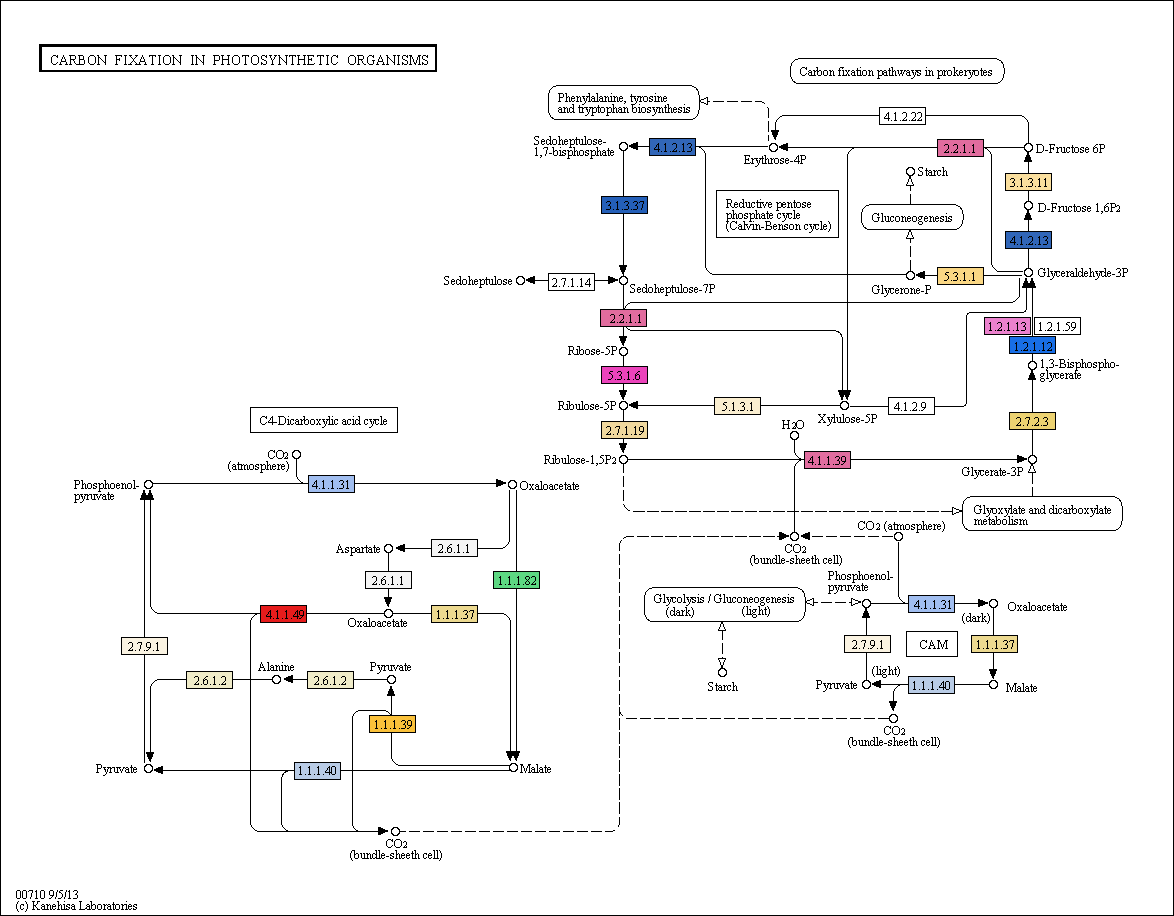

Supplement: Additional file 7: — KEGG pathway annotation. A zip compressed file with a list of KEGGs pathways, graphics in png format, and a file with a comparison with KEGGs pathways of potato and tomato. (ZIP 4361 kb) [file 12864_2016_2656_MOESM7_ESM.zip › Pathway representations/map00710_20150305160636.png]

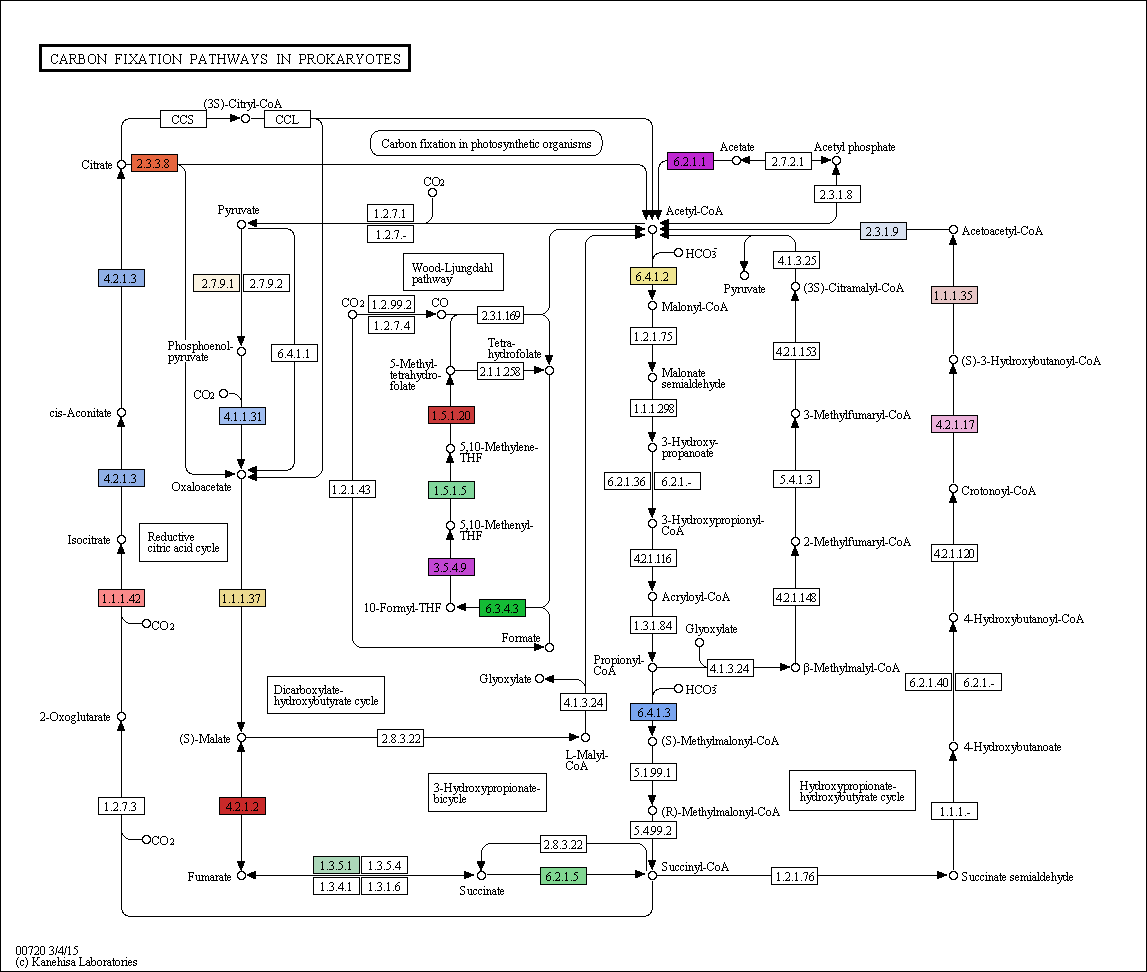

Supplement: Additional file 7: — KEGG pathway annotation. A zip compressed file with a list of KEGGs pathways, graphics in png format, and a file with a comparison with KEGGs pathways of potato and tomato. (ZIP 4361 kb) [file 12864_2016_2656_MOESM7_ESM.zip › Pathway representations/map00720_20150305160518.png]

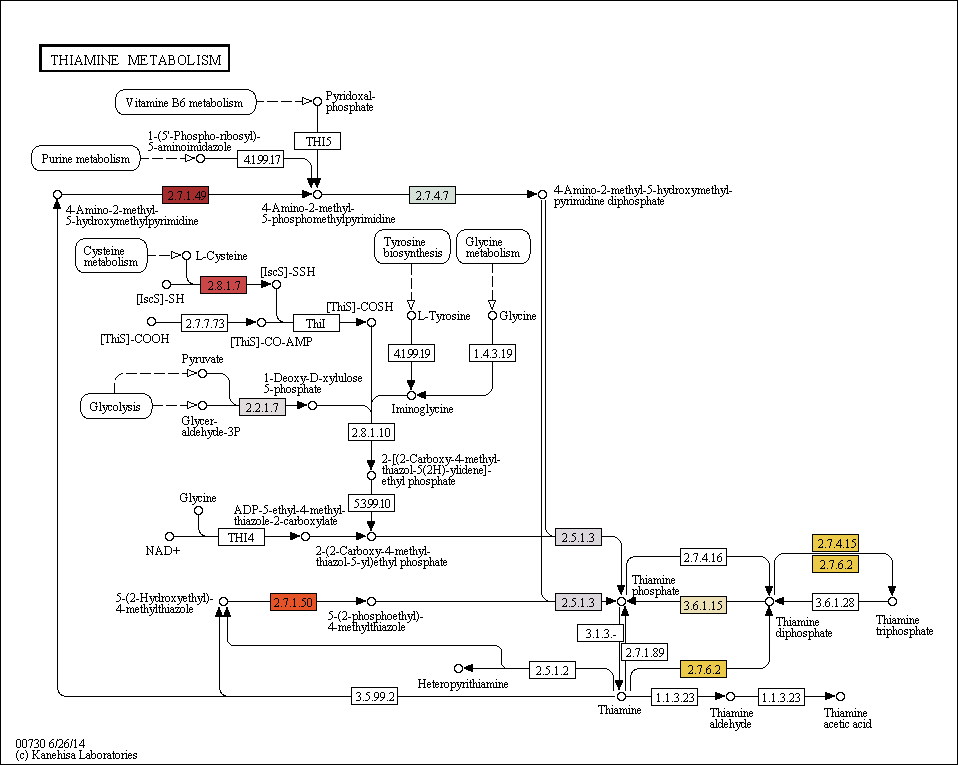

Supplement: Additional file 7: — KEGG pathway annotation. A zip compressed file with a list of KEGGs pathways, graphics in png format, and a file with a comparison with KEGGs pathways of potato and tomato. (ZIP 4361 kb) [file 12864_2016_2656_MOESM7_ESM.zip › Pathway representations/map00730_20150305160426.png]

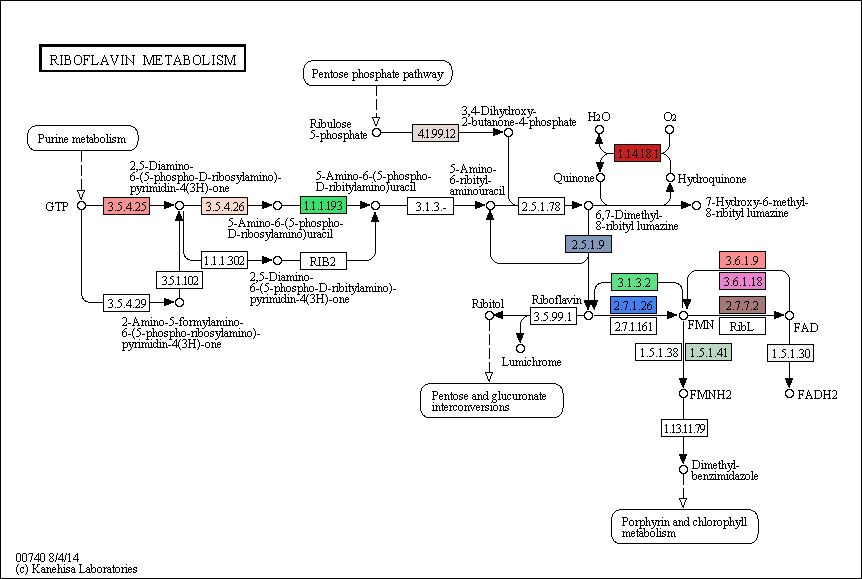

Supplement: Additional file 7: — KEGG pathway annotation. A zip compressed file with a list of KEGGs pathways, graphics in png format, and a file with a comparison with KEGGs pathways of potato and tomato. (ZIP 4361 kb) [file 12864_2016_2656_MOESM7_ESM.zip › Pathway representations/map00740_20150305161622.png]
