# Supplementary material for: The first de novo transcriptome of pepino (Solanum muricatum): assembly, comprehensive analysis and comparison with the closely related species S. caripense, potato and tomato
Source: BMC Genomics. 2016 May 4;17:321. doi: 10.1186/s12864-016-2656-8 (PMC4855764; doi:10.1186/s12864-016-2656-8)
Supplement: Additional file 7: — KEGG pathway annotation. A zip compressed file with a list of KEGGs pathways, graphics in png format, and a file with a comparison with KEGGs pathways of potato and tomato. (ZIP 4361 kb) [file 12864_2016_2656_MOESM7_ESM.zip › KEGG pathway enrichment in Pepino.docx]

| No | Pathway description | Gene members (%) | Pathway ID | Pathway Image |
| --- | --- | --- | --- | --- |
| 1 | Purine metabolism | 1012 (6,27) | map00230 | map00230_20150305161143.png |
| 2 | Starch and sucrose metabolism | 727 (4,50) | map00500 | map00500_20150305160845.png |
| 3 | Phenylalanine metabolism | 464 (2,87) | map00360 | map00360_20150305161527.png |
| 4 | Amino sugar and nucleotide sugar metabolism | 332 (2,06) | map00520 | map00520_20150305160547.png |
| 5 | Pyrimidine metabolism | 326 (2,02) | map00240 | map00240_20150305161033.png |
| 6 | Thiamine metabolism | 325 (2,01) | map00730 | map00730_20150305160426.png |
| 7 | Pyruvate metabolism | 324 (2,01) | map00620 | map00620_20150305161250.png |
| 8 | Glycolysis / Gluconeogenesis | 321 (1,99) | map00010 | map00010_20150305161352.png |
| 9 | Glycerolipid metabolism | 318 (1,97) | map00561 | map00561_20150305161504.png |
| 10 | Cysteine and methionine metabolism | 313 (1,94) | map00270 | map00270_20150305160759.png |
| 11 | Phenylpropanoid biosynthesis | 280 (1,73) | map00940 | map00940_20150305161617.png |
| 12 | Carbon fixation pathways in prokaryotes | 272 (1,68) | map00720 | map00720_20150305160518.png |
| 13 | Pentose and glucuronate interconversions | 268 (1,66) | map00040 | map00040_20150305161044.png |
| 14 | Tyrosine metabolism | 258 (1,60) | map00350 | map00350_20150305161551.png |
| 15 | Phenylalanine, tyrosine and tryptophan biosynthesis | 255 (1,58) | map00400 | map00400_20150305161451.png |
| 16 | Glycerophospholipid metabolism | 252 (1,56) | map00564 | map00564_20150305161437.png |
| 17 | Arginine and proline metabolism | 236 (1,46) | map00330 | map00330_20150305160453.png |
| 18 | Galactose metabolism | 230 (1,42) | map00052 | map00052_20150305161002.png |
| 19 | Fatty acid degradation | 223 (1,38) | map00071 | map00071_20150305160823.png |
| 20 | Valine, leucine and isoleucine degradation | 216 (1,34) | map00280 | map00280_20150305160625.png |
| 21 | T cell receptor signaling pathway | 212 (1,31) | map04660 | map04660_20150305160610.png |
| 22 | Aminobenzoate degradation | 212 (1,31) | map00627 | map00627_20150305161211.png |
| 23 | Carbon fixation in photosynthetic organisms | 208 (1,29) | map00710 | map00710_20150305160636.png |
| 24 | Glycine, serine and threonine metabolism | 204 (1,26) | map00260 | map00260_20150305160915.png |
| 25 | Citrate cycle (TCA cycle) | 204 (1,26) | map00020 | map00020_20150305161317.png |
| 26 | Methane metabolism | 195 (1,21) | map00680 | map00680_20150305160555.png |
| 27 | Phosphatidylinositol signaling system | 191 (1,18) | map04070 | map04070_20150305160601.png |
| 28 | Glutathione metabolism | 187 (1,16) | map00480 | map00480_20150305160608.png |
| 29 | Tryptophan metabolism | 186 (1,15) | map00380 | map00380_20150305161311.png |
| 30 | Oxidative phosphorylation | 185 (1,15) | map00190 | map00190_20150305161200.png |
| 31 | Tropane, piperidine and pyridine alkaloid biosynthesis | 179 (1,11) | map00960 | map00960_20150305161359.png |
| 32 | Fructose and mannose metabolism | 178 (1,10) | map00051 | map00051_20150305161009.png |
| 33 | Propanoate metabolism | 175 (1,08) | map00640 | map00640_20150305161021.png |
| 34 | Pentose phosphate pathway | 172 (1,07) | map00030 | map00030_20150305161152.png |
| 35 | Isoquinoline alkaloid biosynthesis | 170 (1,05) | map00950 | map00950_20150305161533.png |
| 36 | alpha-Linolenic acid metabolism | 168 (1,04) | map00592 | map00592_20150305161112.png |
| 37 | Drug metabolism - cytochrome P450 | 168 (1,04) | map00982 | map00982_20150305161216.png |
| 38 | Glyoxylate and dicarboxylate metabolism | 167 (1,03) | map00630 | map00630_20150305161056.png |
| 39 | Alanine, aspartate and glutamate metabolism | 163 (1,01) | map00250 | map00250_20150305160949.png |
| 40 | Inositol phosphate metabolism | 157 (0,97) | map00562 | map00562_20150305161458.png |
| 41 | Lysine degradation | 154 (0,95) | map00310 | map00310_20150305160714.png |
| 42 | Butanoate metabolism | 154 (0,95) | map00650 | map00650_20150305160931.png |
| 43 | beta-Alanine metabolism | 151 (0,94) | map00410 | map00410_20150305161336.png |
| 44 | Fatty acid biosynthesis | 146 (0,90) | map00061 | map00061_20150305160925.png |
| 45 | Metabolism of xenobiotics by cytochrome P450 | 140 (0,87) | map00980 | map00980_20150305161233.png |
| 46 | Porphyrin and chlorophyll metabolism | 138 (0,85) | map00860 | map00860_20150305160835.png |
| 47 | Sphingolipid metabolism | 137 (0,85) | map00600 | map00600_20150305161414.png |
| 48 | Novobiocin biosynthesis | 137 (0,85) | map00401 | map00401_20150305161441.png |
| 49 | Sulfur metabolism | 131 (0,81) | map00920 | map00920_20150305160512.png |
| 50 | Aminoacyl-tRNA biosynthesis | 124 (0,77) | map00970 | map00970_20150305161325.png |
| 51 | Ascorbate and aldarate metabolism | 123 (0,76) | map00053 | map00053_20150305160955.png |
| 52 | Terpenoid backbone biosynthesis | 118 (0,73) | map00900 | map00900_20150305160810.png |
| 53 | Flavonoid biosynthesis | 118 (0,73) | map00941 | map00941_20150305161611.png |
| 54 | Riboflavin metabolism | 117 (0,72) | map00740 | map00740_20150305161622.png |
| 55 | Biosynthesis of unsaturated fatty acids | 115 (0,71) | map01040 | map01040_20150305161342.png |
| 56 | Ubiquinone and other terpenoid-quinone biosynthesis | 105 (0,65) | map00130 | map00130_20150305160500.png |
| 57 | Steroid hormone biosynthesis | 99 (0,61) | map00140 | map00140_20150305160416.png |
| 58 | Cyanoamino acid metabolism | 98 (0,61) | map00460 | map00460_20150305160900.png |
| 59 | Biotin metabolism | 98 (0,61) | map00780 | map00780_20150305161255.png |
| 60 | One carbon pool by folate | 97 (0,60) | map00670 | map00670_20150305160702.png |
| 61 | Nicotinate and nicotinamide metabolism | 97 (0,60) | map00760 | map00760_20150305161420.png |
| 62 | Drug metabolism - other enzymes | 96 (0,59) | map00983 | map00983_20150305161205.png |
| 63 | Linoleic acid metabolism | 92 (0,57) | map00591 | map00591_20150305161115.png |
| 64 | Other glycan degradation | 91 (0,56) | map00511 | map00511_20150305160651.png |
| 65 | Arachidonic acid metabolism | 90 (0,56) | map00590 | map00590_20150305161125.png |
| 66 | Retinol metabolism | 89 (0,55) | map00830 | map00830_20150305161047.png |
| 67 | Fatty acid elongation | 86 (0,53) | map00062 | map00062_20150305160919.png |
| 68 | Histidine metabolism | 80 (0,50) | map00340 | map00340_20150305161632.png |
| 69 | Lysine biosynthesis | 77 (0,48) | map00300 | map00300_20150305160854.png |
| 70 | Geraniol degradation | 75 (0,46) | map00281 | map00281_20150305160618.png |
| 71 | Selenocompound metabolism | 74 (0,46) | map00450 | map00450_20150305160937.png |
| 72 | Caprolactam degradation | 71 (0,44) | map00930 | map00930_20150305160421.png |
| 73 | Nitrogen metabolism | 70 (0,43) | map00910 | map00910_20150305160631.png |
| 74 | Glycosaminoglycan degradation | 68 (0,42) | map00531 | map00531_20150305160436.png |
| 75 | Pantothenate and CoA biosynthesis | 68 (0,42) | map00770 | map00770_20150305161330.png |
| 76 | Zeatin biosynthesis | 64 (0,40) | map00908 | map00908_20150305160728.png |
| 77 | Glycosaminoglycan biosynthesis - heparan sulfate / heparin | 63 (0,39) | map00534 | map00534_20150305160430.png |
| 78 | Folate biosynthesis | 59 (0,37) | map00790 | map00790_20150305161104.png |
| 79 | mTOR signaling pathway | 59 (0,37) | map04150 | map04150_20150305161422.png |
| 80 | Valine, leucine and isoleucine biosynthesis | 58 (0,36) | map00291 | map00290_20150305160505.png |
| 81 | Glycosphingolipid biosynthesis - ganglio series | 57 (0,35) | map00604 | map00604_20150305161402.png |
| 82 | Ether lipid metabolism | 56 (0,35) | map00565 | map00565_20150305161426.png |
| 83 | N-Glycan biosynthesis | 54 (0,33) | map00510 | map00510_20150305160656.png |
| 84 | Limonene and pinene degradation | 54 (0,33) | map00903 | map00903_20150305160744.png |
| 85 | Streptomycin biosynthesis | 52 (0,32) | map00521 | map00521_20150305160530.png |
| 86 | Glycosaminoglycan biosynthesis - chondroitin sulfate / dermatan sulfate | 50 (0,31) | map00532 | map00532_20150305160433.png |
| 87 | Steroid biosynthesis | 50 (0,31) | map00100 | map00100_20150305160905.png |
| 88 | Chloroalkane and chloroalkene degradation | 50 (0,31) | map00625 | map00625_20150305161227.png |
| 89 | Benzoate degradation | 49 (0,30) | map00362 | map00362_20150305161513.png |
| 90 | Monoterpenoid biosynthesis | 43 (0,27) | map00902 | map00902_20150305160748.png |
| 91 | Cutin, suberine and wax biosynthesis | 42 (0,26) | map00073 | map00073_20150305160813.png |
| 92 | Various types of N-glycan biosynthesis | 40 (0,25) | map00513 | map00513_20150305160644.png |
| 93 | Indole alkaloid biosynthesis | 40 (0,25) | map00901 | map00901_20150305160802.png |
| 94 | Caffeine metabolism | 39 (0,24) | map00232 | map00232_20150305161127.png |
| 95 | Toluene degradation | 37 (0,23) | map00623 | map00623_20150305161237.png |
| 96 | Steroid degradation | 36 (0,22) | map00984 | map00984_20150305161154.png |
| 97 | Synthesis and degradation of ketone bodies | 35 (0,22) | map00072 | map00072_20150305160817.png |
| 98 | Biosynthesis of ansamycins | 35 (0,22) | map01051 | map01051_20150305161304.png |
| 99 | Taurine and hypotaurine metabolism | 34 (0,21) | map00430 | map00430_20150305161108.png |
| 100 | Carotenoid biosynthesis | 33 (0,20) | map00906 | map00906_20150305160733.png |
| 101 | C5-Branched dibasic acid metabolism | 33 (0,20) | map00660 | map00660_20150305160848.png |
| 102 | Styrene degradation | 33 (0,20) | map00643 | map00643_20150305161013.png |
| 103 | Primary bile acid biosynthesis | 32 (0,20) | map00120 | map00120_20150305160614.png |
| 104 | Flavone and flavonol biosynthesis | 31 (0,19) | map00944 | map00944_20150305161558.png |
| 105 | Anthocyanin biosynthesis | 31 (0,19) | map00942 | map00942_20150305161605.png |
| 106 | Vitamin B6 metabolism | 28 (0,17) | map00750 | map00750_20150305161538.png |
| 107 | Diterpenoid biosynthesis | 27 (0,17) | map00904 | map00904_20150305160740.png |
| 108 | Lipopolysaccharide biosynthesis | 24 (0,15) | map00540 | map00540_20150305161626.png |
| 109 | Stilbenoid, diarylheptanoid and gingerol biosynthesis | 22 (0,14) | map00945 | map00945_20150305161554.png |
| 110 | Naphthalene degradation | 20 (0,12) | map00626 | map00626_20150305161220.png |
| 111 | Glucosinolate biosynthesis | 20 (0,12) | map00966 | map00966_20150305161344.png |
| 112 | Sesquiterpenoid and triterpenoid biosynthesis | 19 (0,12) | map00909 | map00909_20150305160724.png |
| 113 | Betalain biosynthesis | 19 (0,12) | map00965 | map00965_20150305161354.png |
| 114 | Glycosphingolipid biosynthesis - globo series | 18 (0,11) | map00603 | map00603_20150305161405.png |
| 115 | Butirosin and neomycin biosynthesis | 17 (0,11) | map00524 | map00524_20150305160521.png |
| 116 | Aflatoxin biosynthesis | 16 (0,10) | map00254 | map00254_20150305160939.png |
| 117 | Tetracycline biosynthesis | 16 (0,10) | map00253 | map00253_20150305160941.png |
| 118 | Chlorocyclohexane and chlorobenzene degradation | 14 (0,09) | map00361 | map00361_20150305161518.png |
| 119 | Peptidoglycan biosynthesis | 14 (0,09) | map00550 | map00550_20150305161543.png |
| 120 | D-Glutamine and D-glutamate metabolism | 13 (0,08) | map00471 | map00471_20150305160720.png |
| 121 | Biosynthesis of siderophore group nonribosomal peptides | 13 (0,08) | map01053 | map01053_20150305161301.png |
| 122 | Other types of O-glycan biosynthesis | 12 (0,07) | map00514 | map00514_20150305160639.png |
| 123 | Polyketide sugar unit biosynthesis | 11 (0,07) | map00523 | map00523_20150305160526.png |
| 124 | Carbapenem biosynthesis | 10 (0,06) | map00332 | map00332_20150305160441.png |
| 125 | Atrazine degradation | 10 (0,06) | map00791 | map00791_20150305161058.png |
| 126 | Glycosylphosphatidylinositol(GPI)-anchor biosynthesis | 10 (0,06) | map00563 | map00563_20150305161444.png |
| 127 | D-Alanine metabolism | 9 (0,06) | map00473 | map00473_20150305160708.png |
| 128 | Fluorobenzoate degradation | 9 (0,06) | map00364 | map00364_20150305161507.png |
| 129 | Isoflavonoid biosynthesis | 9 (0,06) | map00943 | map00943_20150305161602.png |
| 130 | Phosphonate and phosphinate metabolism | 7 (0,04) | map00440 | map00440_20150305161024.png |
| 131 | Photosynthesis | 7 (0,04) | map00195 | map00195_20150305161145.png |
| 132 | D-Arginine and D-ornithine metabolism | 6 (0,04) | map00472 | map00472_20150305160716.png |
| 133 | Ethylbenzene degradation | 6 (0,04) | map00642 | map00642_20150305161015.png |
| 134 | Biosynthesis of terpenoids and steroids | 5 (0,03) | map01062 | map01062_20150305161119.png |
| 135 | Insect hormone biosynthesis | 5 (0,03) | map00981 | map00981_20150305161223.png |
| 136 | Lipoic acid metabolism | 5 (0,03) | map00785 | map00785_20150305161241.png |
| 137 | Penicillin and cephalosporin biosynthesis | 4 (0,02) | map00311 | map00311_20150305160704.png |
| 138 | Biosynthesis of vancomycin group antibiotics | 4 (0,02) | map01055 | map01055_20150305161257.png |
| 139 | Mucin type O-Glycan biosynthesis | 2 (0,01) | map00512 | map00512_20150305160646.png |
| 140 | Brassinosteroid biosynthesis | 2 (0,01) | map00905 | map00905_20150305160736.png |
| 141 | Xylene degradation | 2 (0,01) | map00622 | map00622_20150305161243.png |
| 142 | Glycosphingolipid biosynthesis - lacto and neolacto series | 2 (0,01) | map00601 | map00601_20150305161409.png |
| 143 | beta-Lactam resistance | 1 (0,01) | map01501 | map01501_20150305161037.png |
| 144 | Benzoxazinoid biosynthesis | 1 (0,01) | map00402 | map00402_20150305161429.png |
