# Supplementary figures and images for: The first de novo transcriptome of pepino (Solanum muricatum): assembly, comprehensive analysis and comparison with the closely related species S. caripense, potato and tomato (part 2 of 2)
Source: BMC Genomics. 2016 May 4;17:321. doi: 10.1186/s12864-016-2656-8 (PMC4855764; doi:10.1186/s12864-016-2656-8)

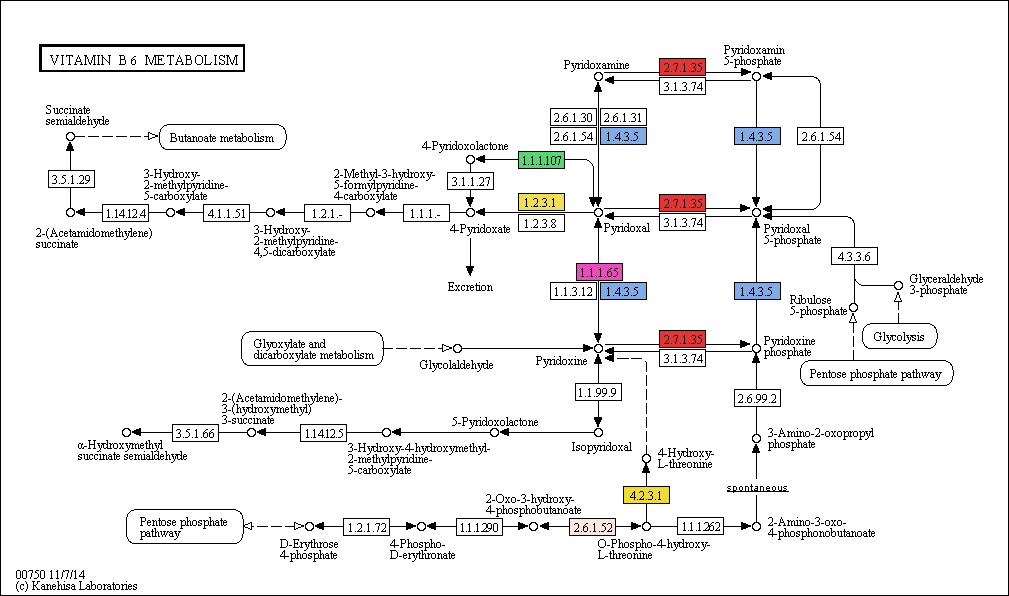

Supplement: Additional file 7: — KEGG pathway annotation. A zip compressed file with a list of KEGGs pathways, graphics in png format, and a file with a comparison with KEGGs pathways of potato and tomato. (ZIP 4361 kb) [file 12864_2016_2656_MOESM7_ESM.zip › Pathway representations/map00750_20150305161538.png]

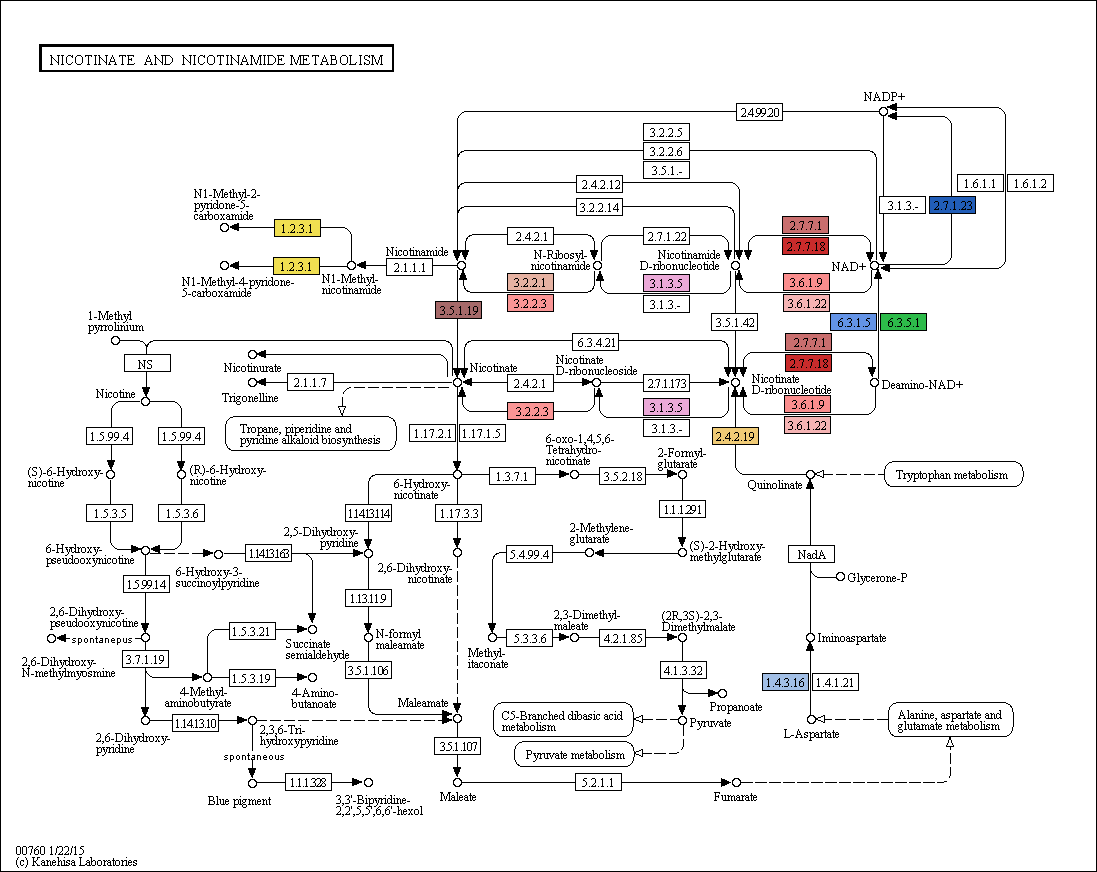

Supplement: Additional file 7: — KEGG pathway annotation. A zip compressed file with a list of KEGGs pathways, graphics in png format, and a file with a comparison with KEGGs pathways of potato and tomato. (ZIP 4361 kb) [file 12864_2016_2656_MOESM7_ESM.zip › Pathway representations/map00760_20150305161420.png]

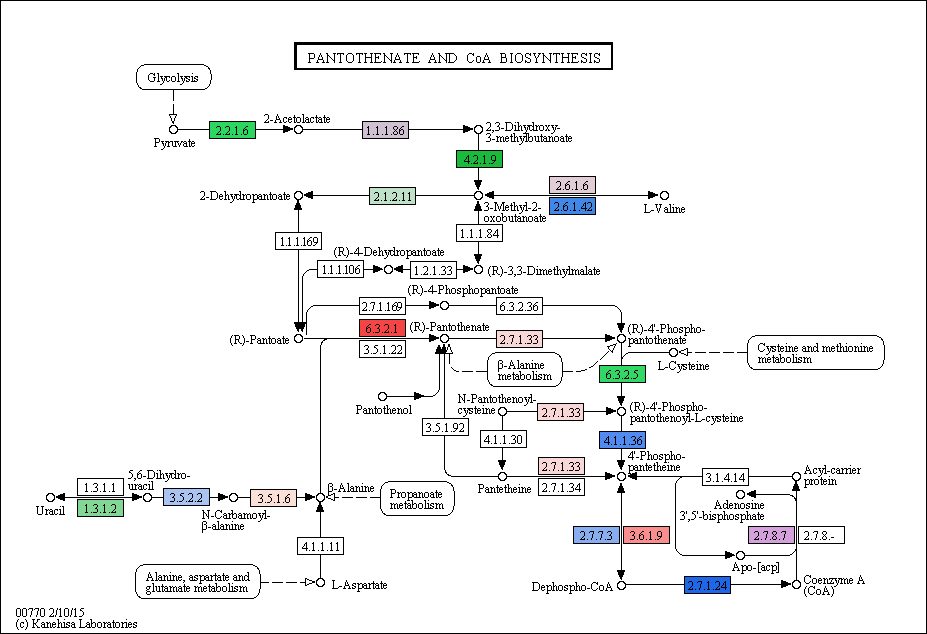

Supplement: Additional file 7: — KEGG pathway annotation. A zip compressed file with a list of KEGGs pathways, graphics in png format, and a file with a comparison with KEGGs pathways of potato and tomato. (ZIP 4361 kb) [file 12864_2016_2656_MOESM7_ESM.zip › Pathway representations/map00770_20150305161330.png]

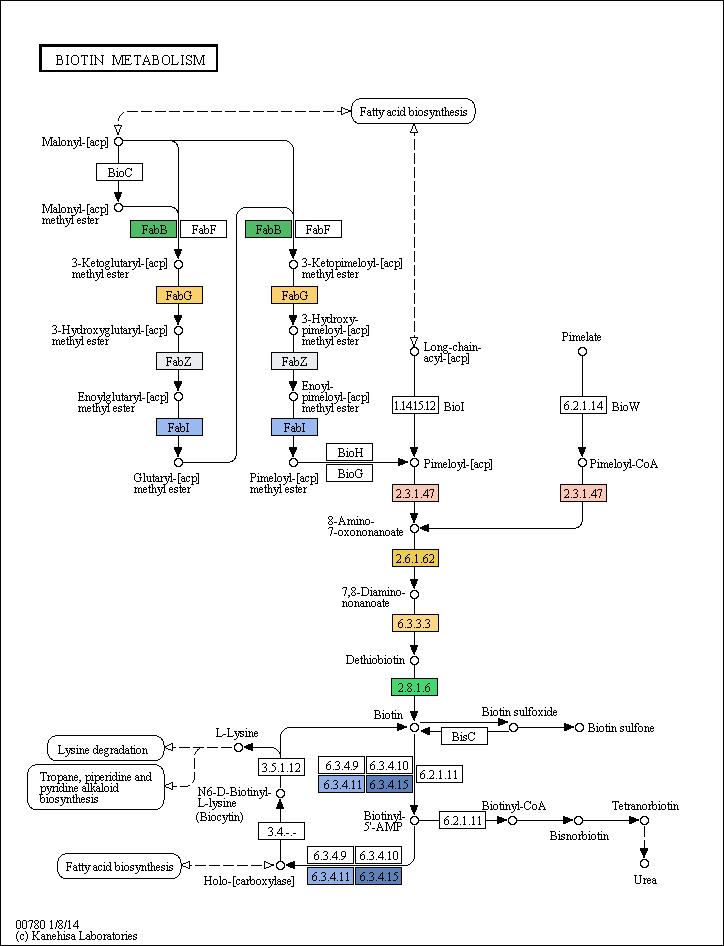

Supplement: Additional file 7: — KEGG pathway annotation. A zip compressed file with a list of KEGGs pathways, graphics in png format, and a file with a comparison with KEGGs pathways of potato and tomato. (ZIP 4361 kb) [file 12864_2016_2656_MOESM7_ESM.zip › Pathway representations/map00780_20150305161255.png]

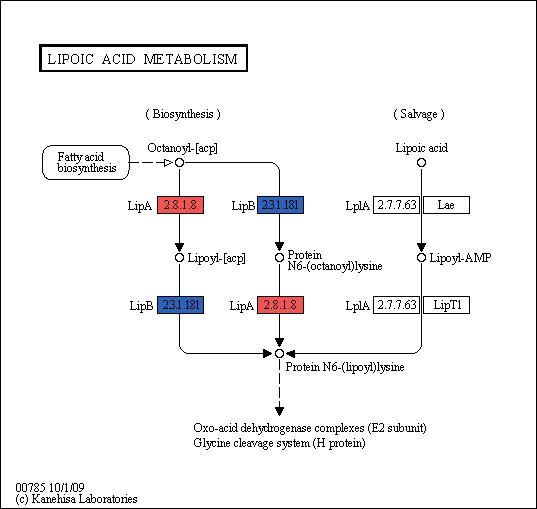

Supplement: Additional file 7: — KEGG pathway annotation. A zip compressed file with a list of KEGGs pathways, graphics in png format, and a file with a comparison with KEGGs pathways of potato and tomato. (ZIP 4361 kb) [file 12864_2016_2656_MOESM7_ESM.zip › Pathway representations/map00785_20150305161241.png]

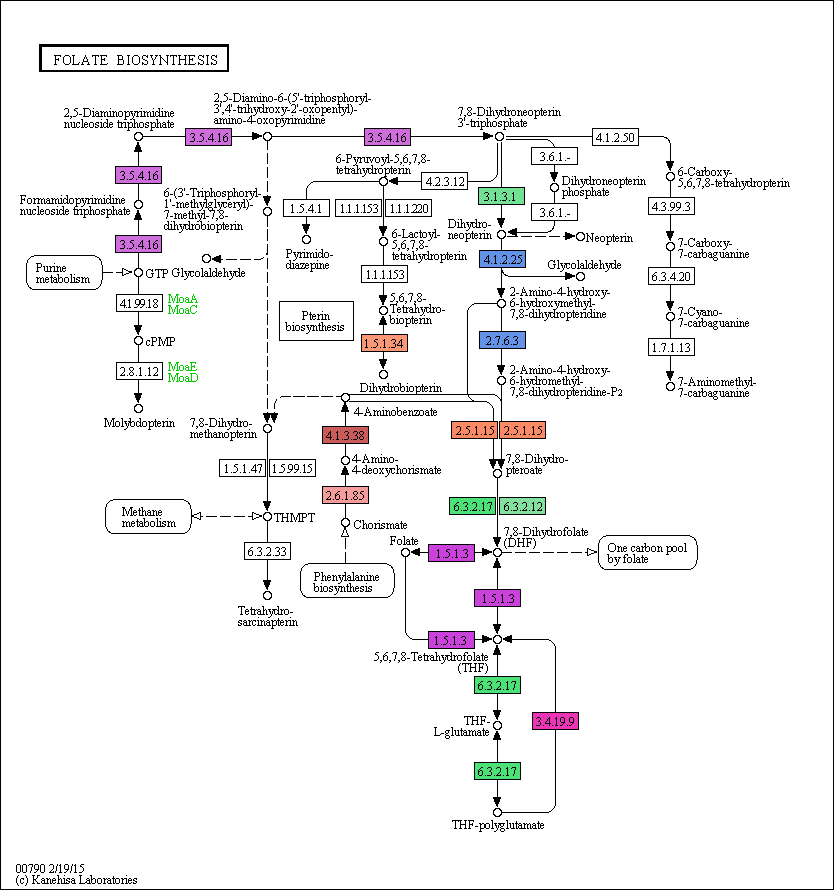

Supplement: Additional file 7: — KEGG pathway annotation. A zip compressed file with a list of KEGGs pathways, graphics in png format, and a file with a comparison with KEGGs pathways of potato and tomato. (ZIP 4361 kb) [file 12864_2016_2656_MOESM7_ESM.zip › Pathway representations/map00790_20150305161104.png]

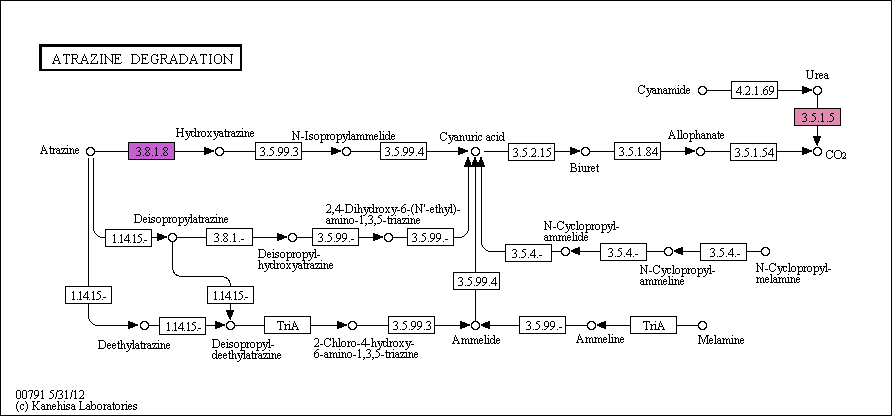

Supplement: Additional file 7: — KEGG pathway annotation. A zip compressed file with a list of KEGGs pathways, graphics in png format, and a file with a comparison with KEGGs pathways of potato and tomato. (ZIP 4361 kb) [file 12864_2016_2656_MOESM7_ESM.zip › Pathway representations/map00791_20150305161058.png]

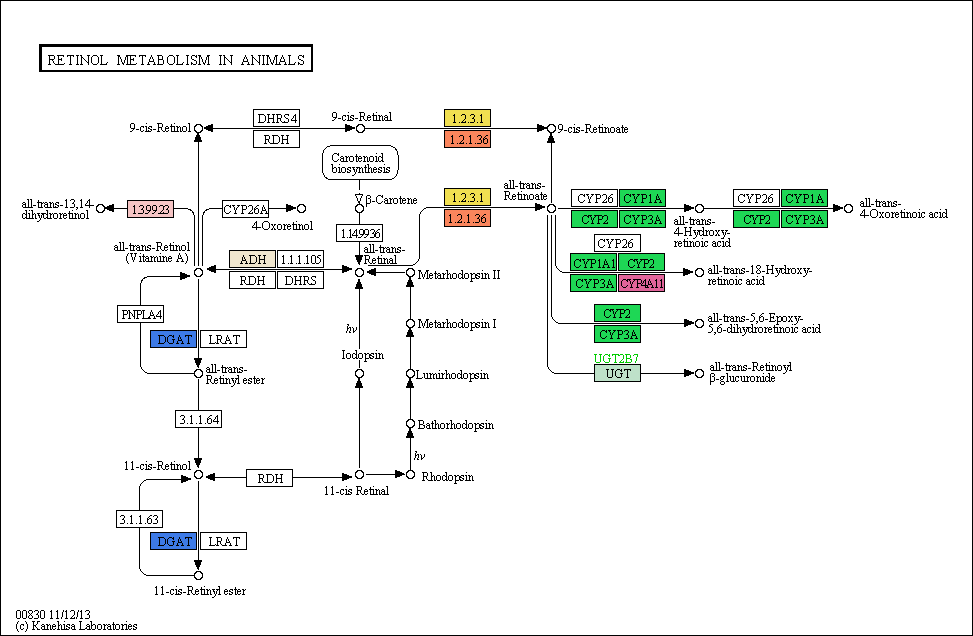

Supplement: Additional file 7: — KEGG pathway annotation. A zip compressed file with a list of KEGGs pathways, graphics in png format, and a file with a comparison with KEGGs pathways of potato and tomato. (ZIP 4361 kb) [file 12864_2016_2656_MOESM7_ESM.zip › Pathway representations/map00830_20150305161047.png]

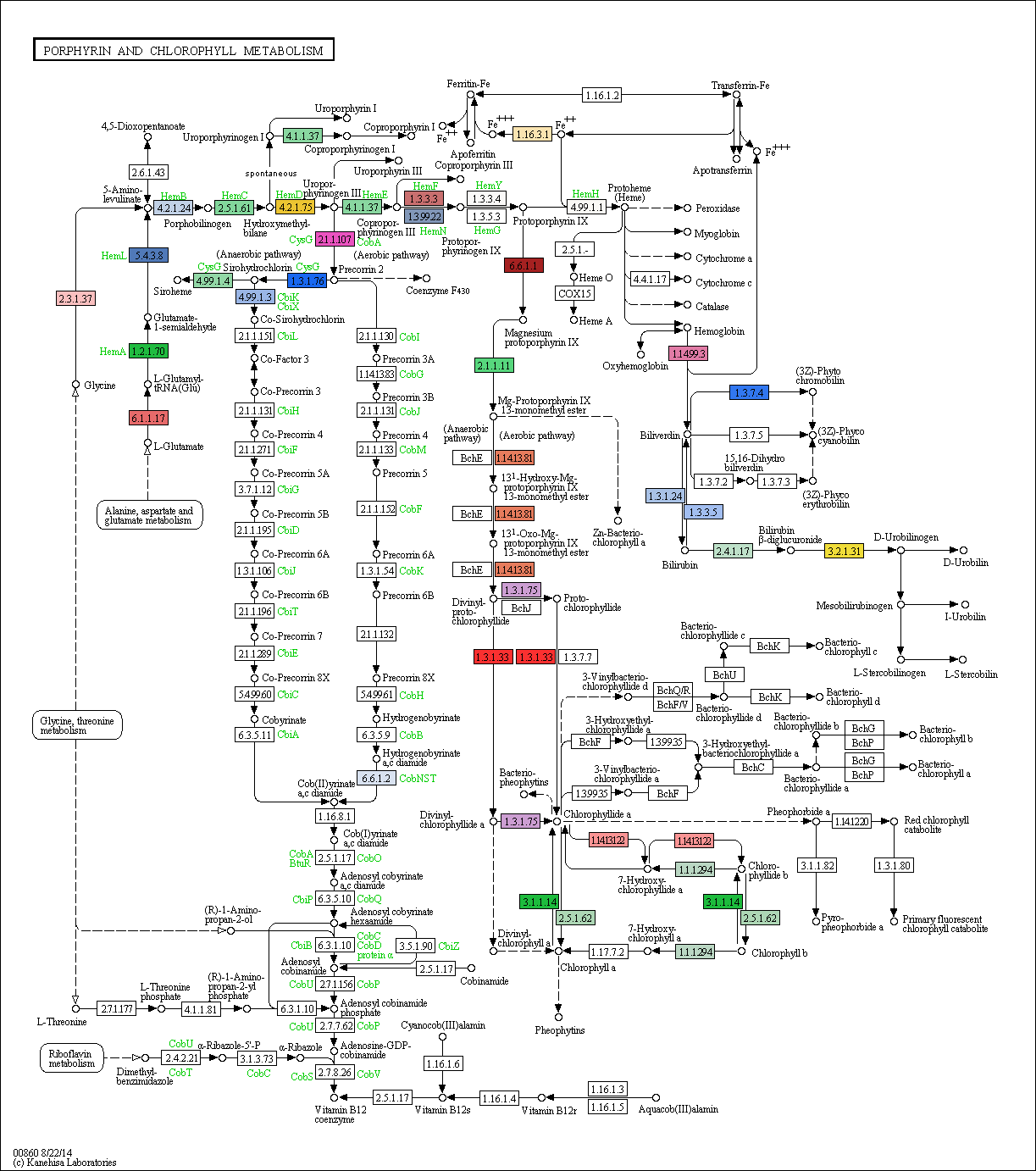

Supplement: Additional file 7: — KEGG pathway annotation. A zip compressed file with a list of KEGGs pathways, graphics in png format, and a file with a comparison with KEGGs pathways of potato and tomato. (ZIP 4361 kb) [file 12864_2016_2656_MOESM7_ESM.zip › Pathway representations/map00860_20150305160835.png]

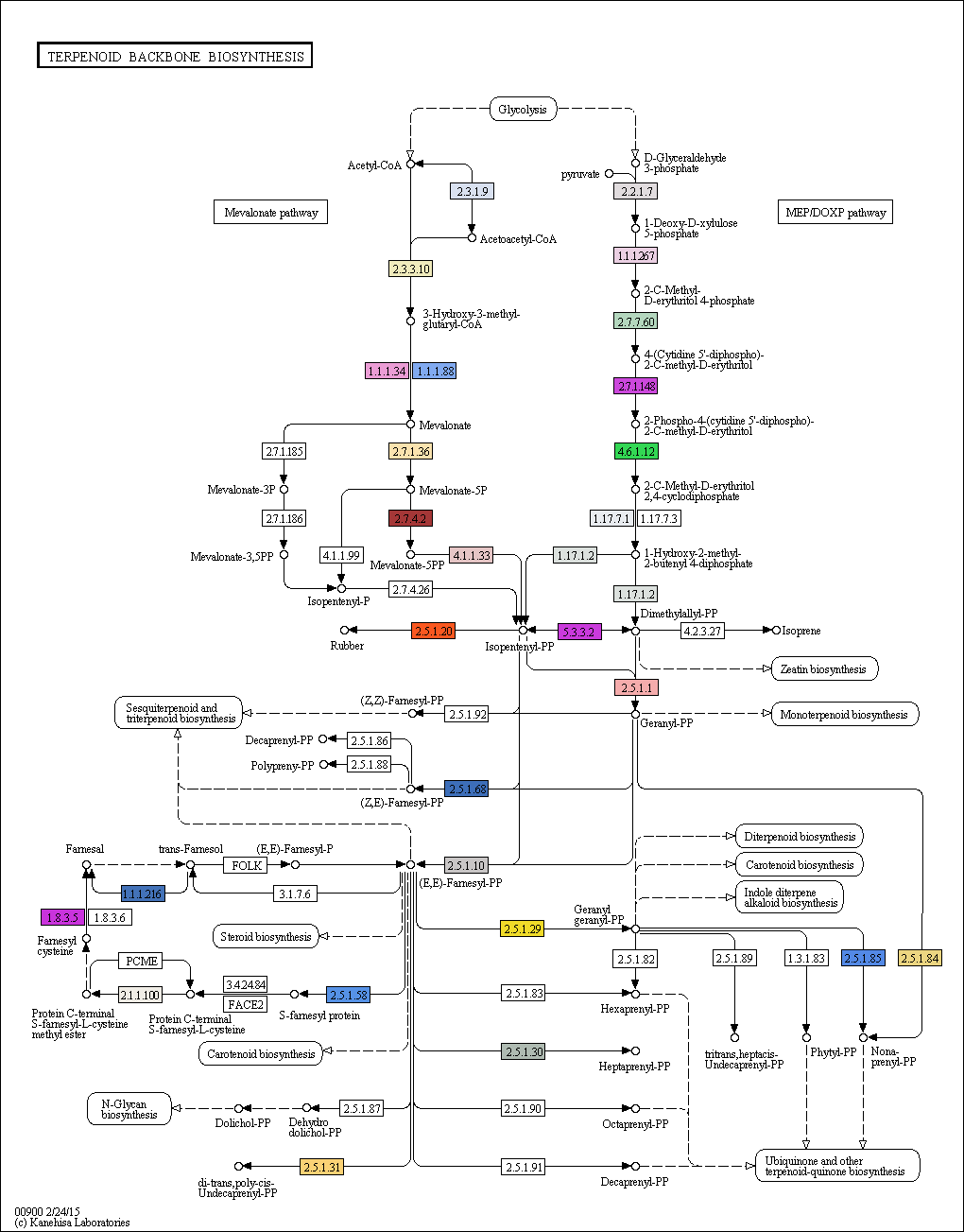

Supplement: Additional file 7: — KEGG pathway annotation. A zip compressed file with a list of KEGGs pathways, graphics in png format, and a file with a comparison with KEGGs pathways of potato and tomato. (ZIP 4361 kb) [file 12864_2016_2656_MOESM7_ESM.zip › Pathway representations/map00900_20150305160810.png]

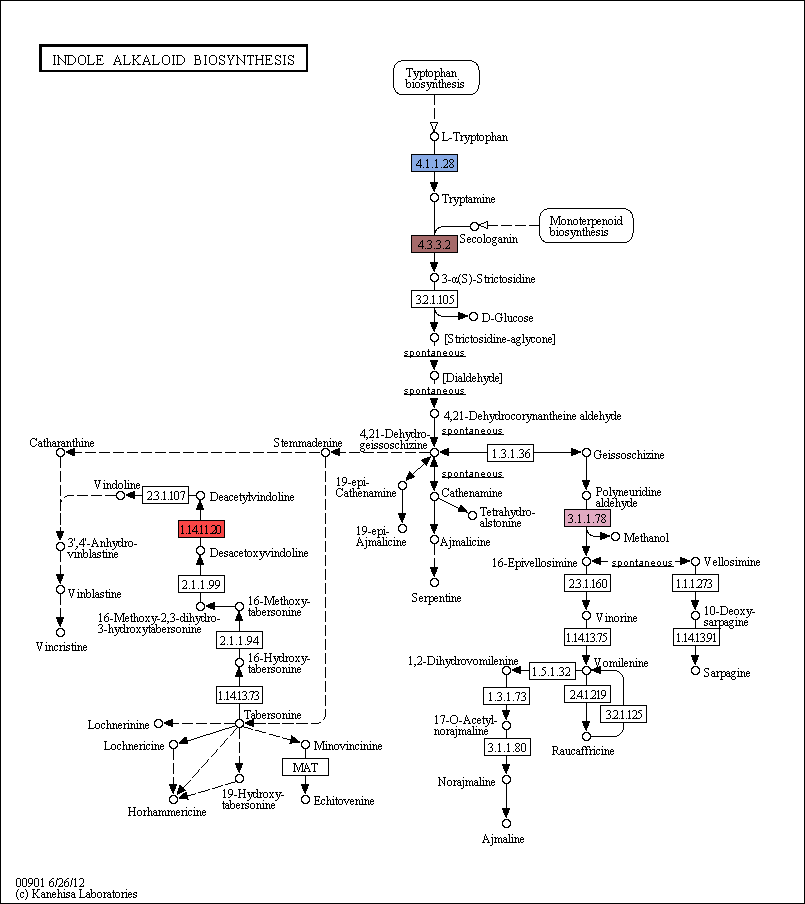

Supplement: Additional file 7: — KEGG pathway annotation. A zip compressed file with a list of KEGGs pathways, graphics in png format, and a file with a comparison with KEGGs pathways of potato and tomato. (ZIP 4361 kb) [file 12864_2016_2656_MOESM7_ESM.zip › Pathway representations/map00901_20150305160802.png]

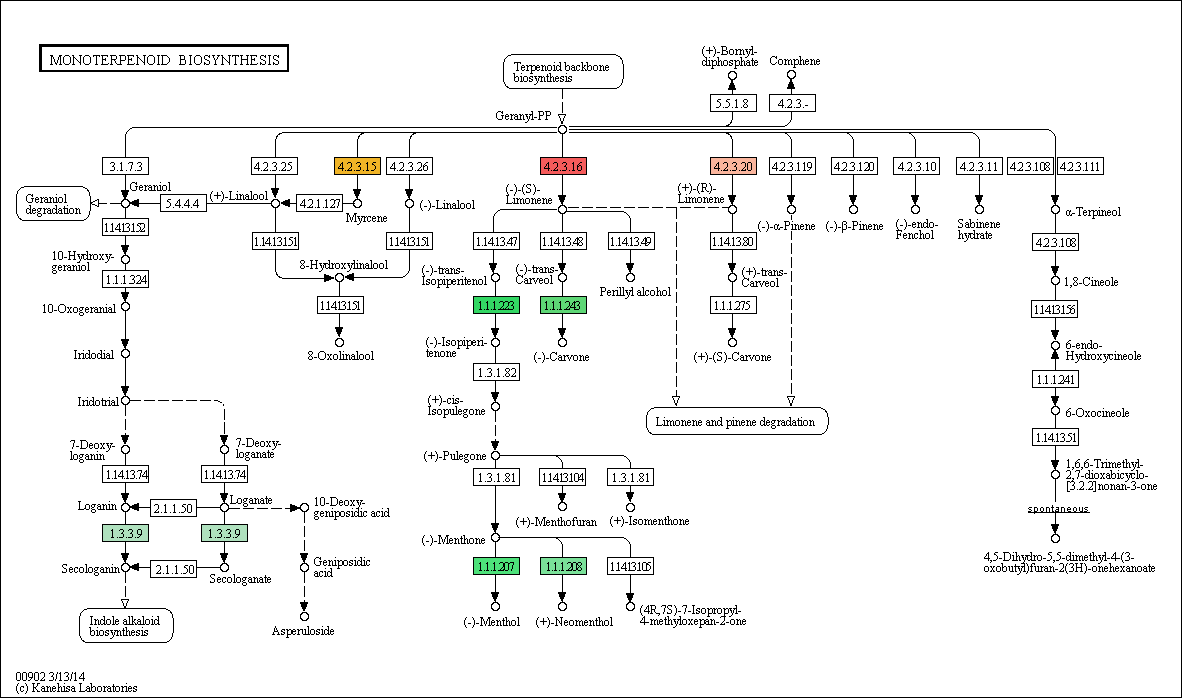

Supplement: Additional file 7: — KEGG pathway annotation. A zip compressed file with a list of KEGGs pathways, graphics in png format, and a file with a comparison with KEGGs pathways of potato and tomato. (ZIP 4361 kb) [file 12864_2016_2656_MOESM7_ESM.zip › Pathway representations/map00902_20150305160748.png]

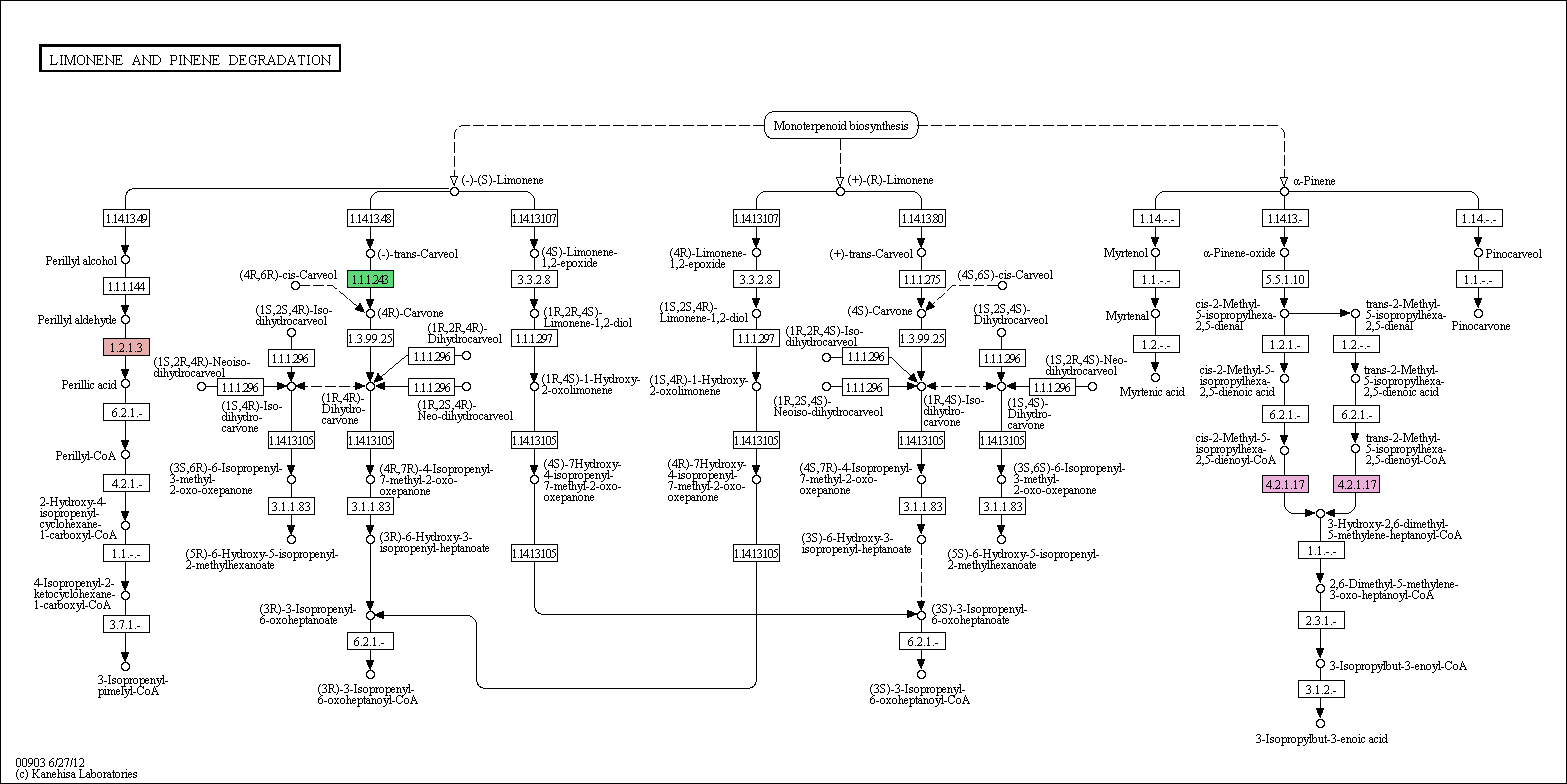

Supplement: Additional file 7: — KEGG pathway annotation. A zip compressed file with a list of KEGGs pathways, graphics in png format, and a file with a comparison with KEGGs pathways of potato and tomato. (ZIP 4361 kb) [file 12864_2016_2656_MOESM7_ESM.zip › Pathway representations/map00903_20150305160744.png]

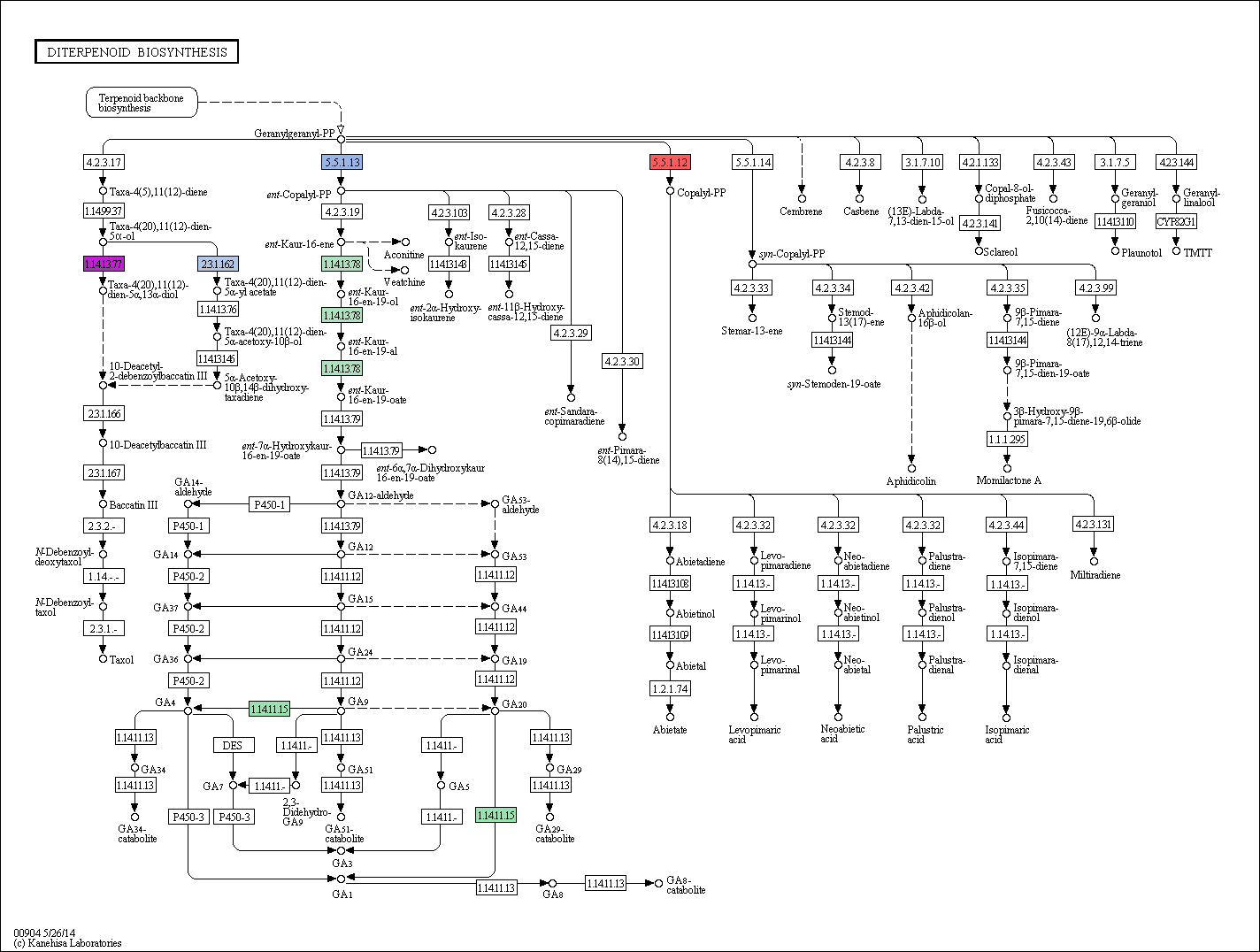

Supplement: Additional file 7: — KEGG pathway annotation. A zip compressed file with a list of KEGGs pathways, graphics in png format, and a file with a comparison with KEGGs pathways of potato and tomato. (ZIP 4361 kb) [file 12864_2016_2656_MOESM7_ESM.zip › Pathway representations/map00904_20150305160740.png]

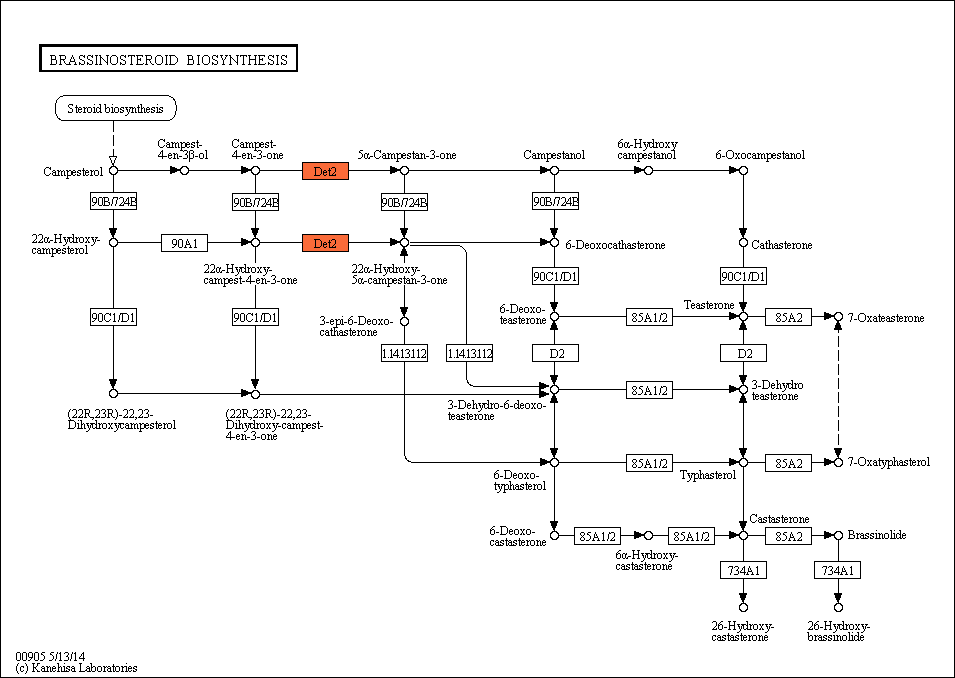

Supplement: Additional file 7: — KEGG pathway annotation. A zip compressed file with a list of KEGGs pathways, graphics in png format, and a file with a comparison with KEGGs pathways of potato and tomato. (ZIP 4361 kb) [file 12864_2016_2656_MOESM7_ESM.zip › Pathway representations/map00905_20150305160736.png]

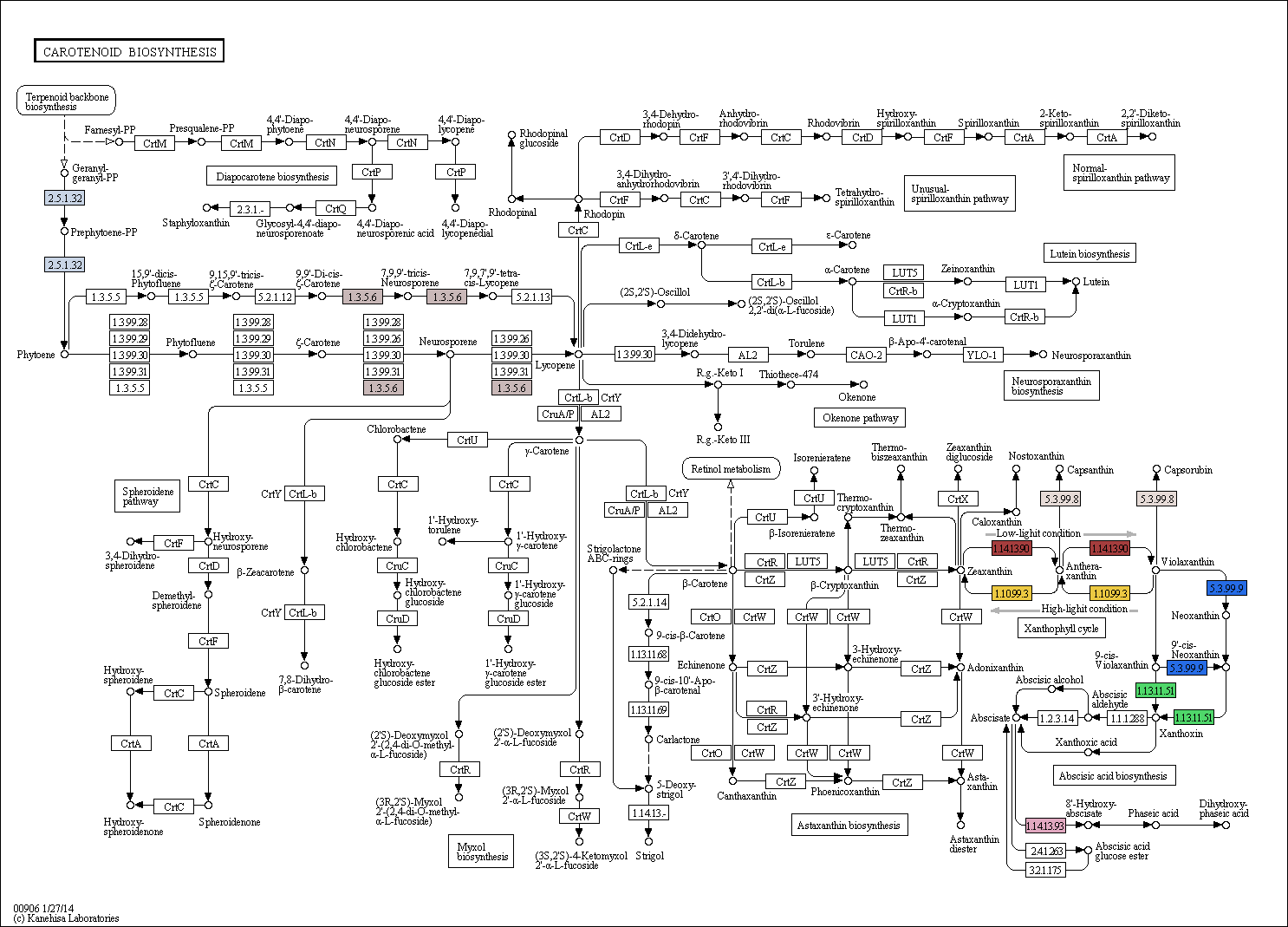

Supplement: Additional file 7: — KEGG pathway annotation. A zip compressed file with a list of KEGGs pathways, graphics in png format, and a file with a comparison with KEGGs pathways of potato and tomato. (ZIP 4361 kb) [file 12864_2016_2656_MOESM7_ESM.zip › Pathway representations/map00906_20150305160733.png]

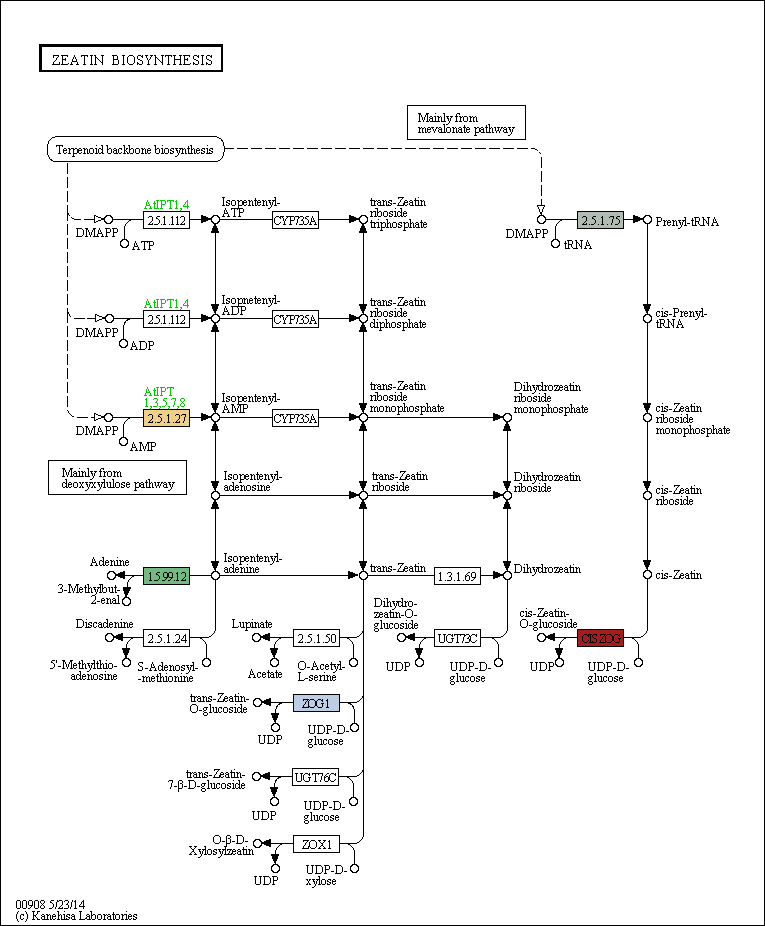

Supplement: Additional file 7: — KEGG pathway annotation. A zip compressed file with a list of KEGGs pathways, graphics in png format, and a file with a comparison with KEGGs pathways of potato and tomato. (ZIP 4361 kb) [file 12864_2016_2656_MOESM7_ESM.zip › Pathway representations/map00908_20150305160728.png]

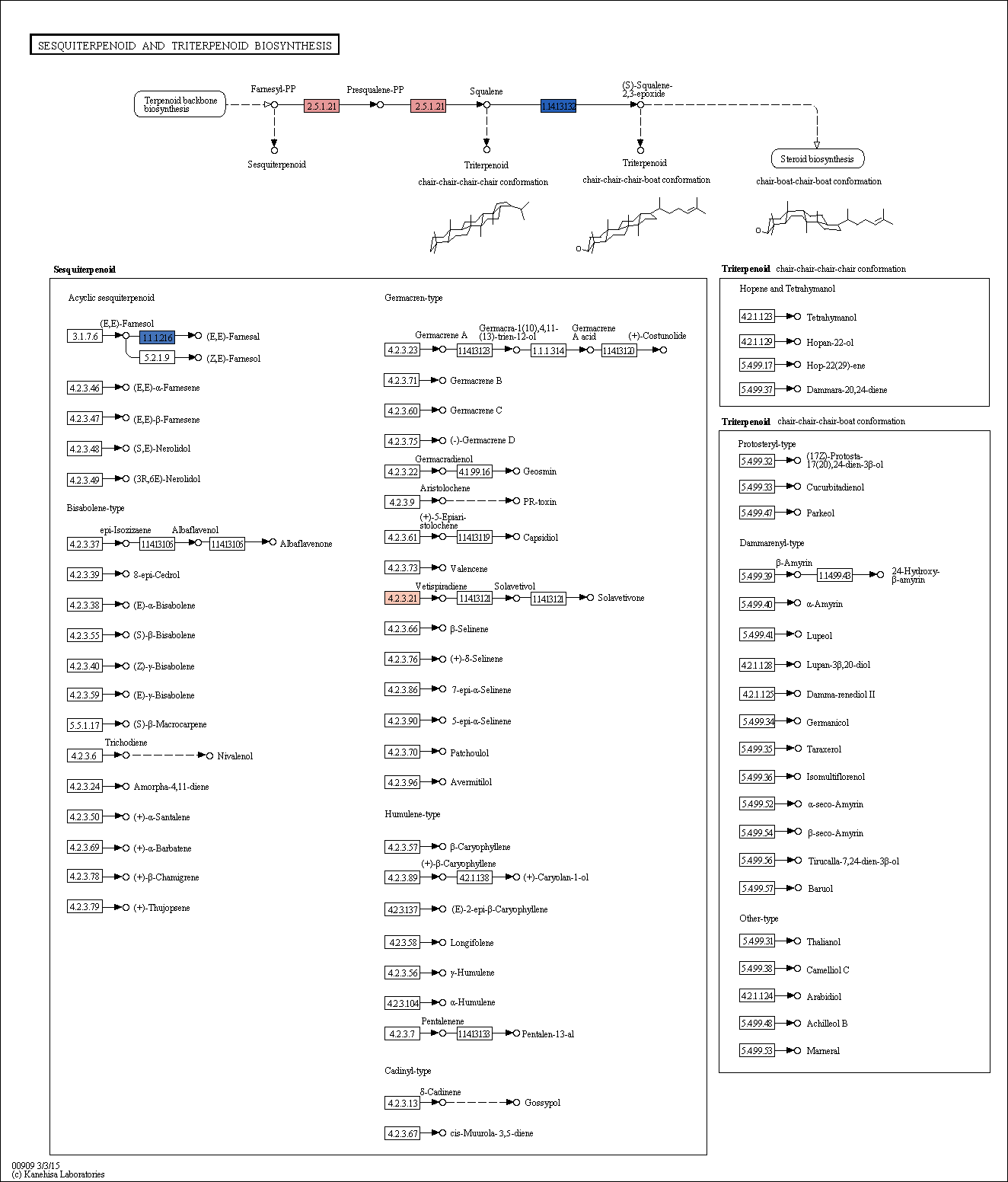

Supplement: Additional file 7: — KEGG pathway annotation. A zip compressed file with a list of KEGGs pathways, graphics in png format, and a file with a comparison with KEGGs pathways of potato and tomato. (ZIP 4361 kb) [file 12864_2016_2656_MOESM7_ESM.zip › Pathway representations/map00909_20150305160724.png]

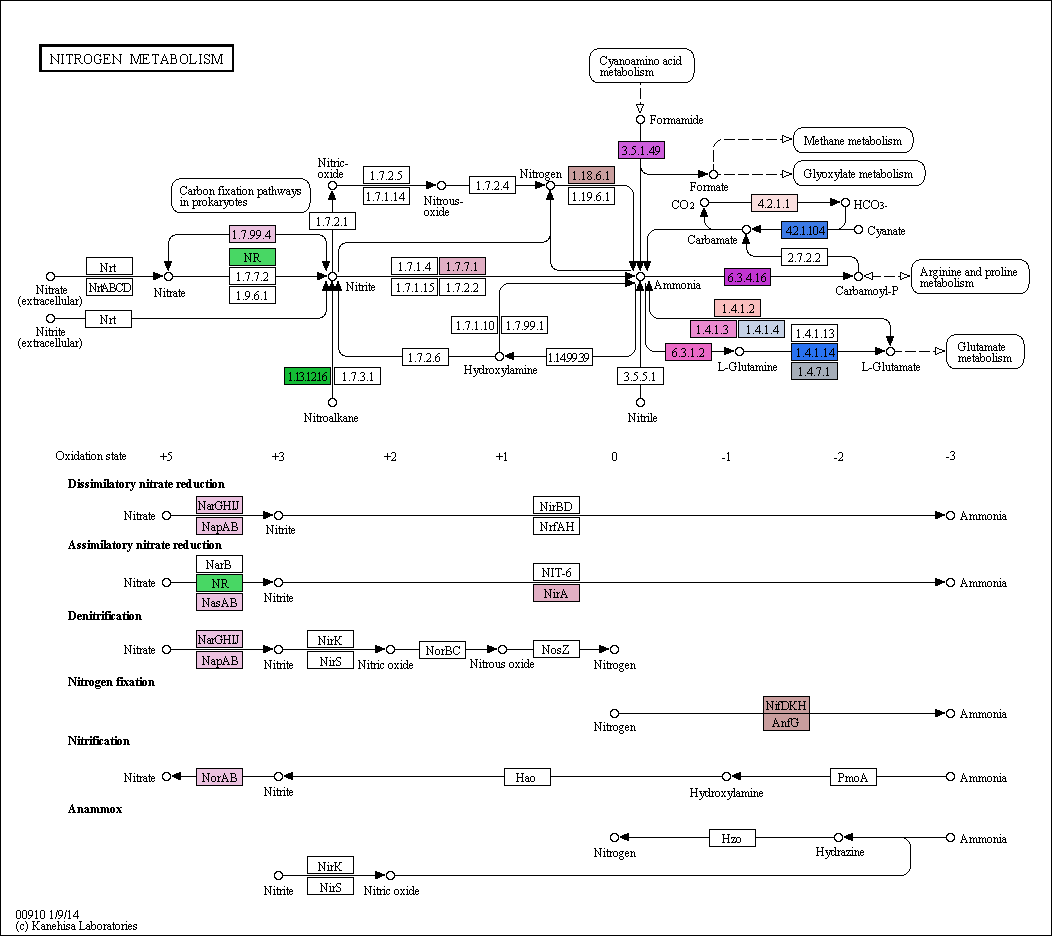

Supplement: Additional file 7: — KEGG pathway annotation. A zip compressed file with a list of KEGGs pathways, graphics in png format, and a file with a comparison with KEGGs pathways of potato and tomato. (ZIP 4361 kb) [file 12864_2016_2656_MOESM7_ESM.zip › Pathway representations/map00910_20150305160631.png]

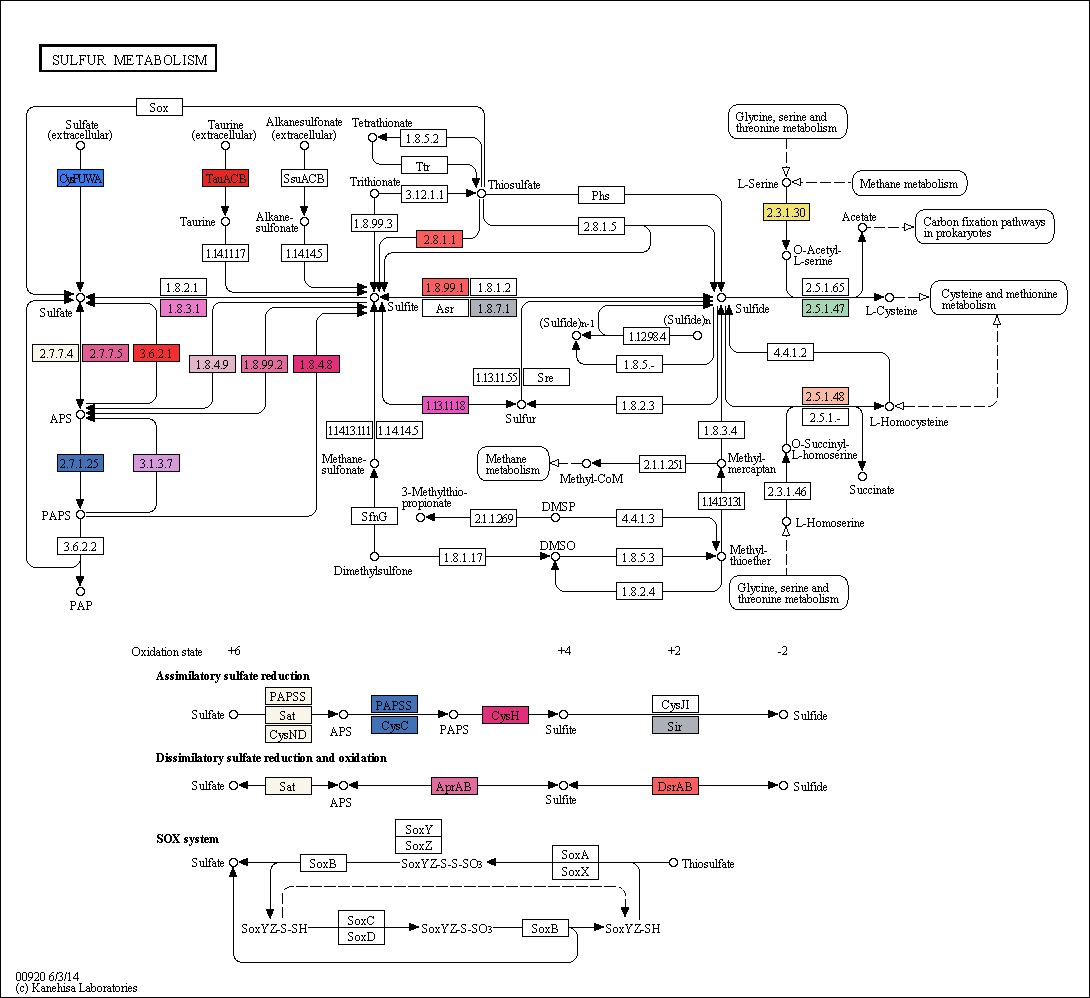

Supplement: Additional file 7: — KEGG pathway annotation. A zip compressed file with a list of KEGGs pathways, graphics in png format, and a file with a comparison with KEGGs pathways of potato and tomato. (ZIP 4361 kb) [file 12864_2016_2656_MOESM7_ESM.zip › Pathway representations/map00920_20150305160512.png]

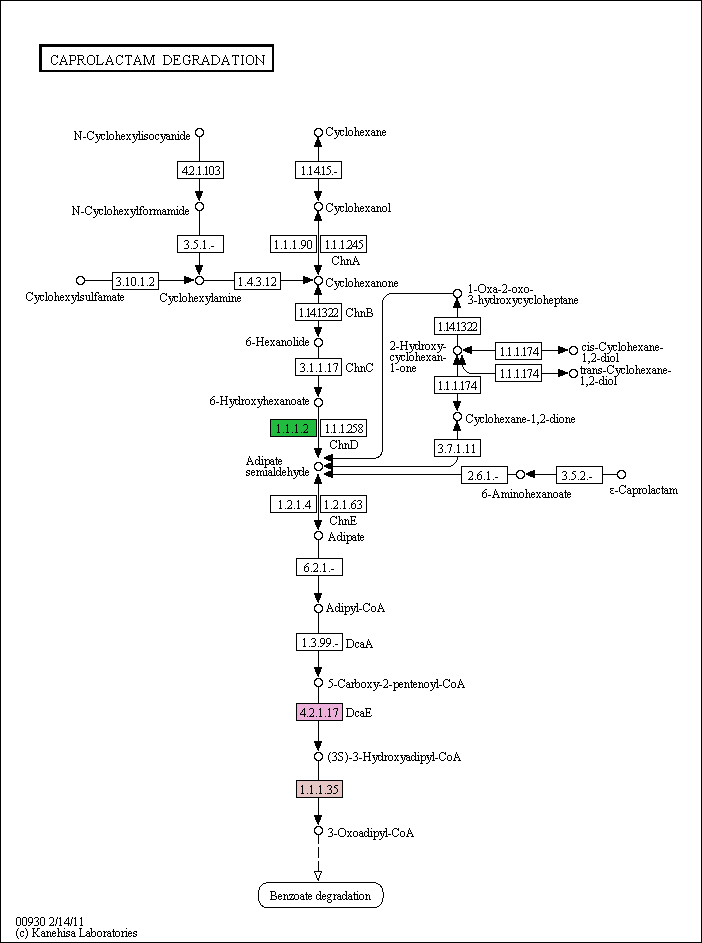

Supplement: Additional file 7: — KEGG pathway annotation. A zip compressed file with a list of KEGGs pathways, graphics in png format, and a file with a comparison with KEGGs pathways of potato and tomato. (ZIP 4361 kb) [file 12864_2016_2656_MOESM7_ESM.zip › Pathway representations/map00930_20150305160421.png]

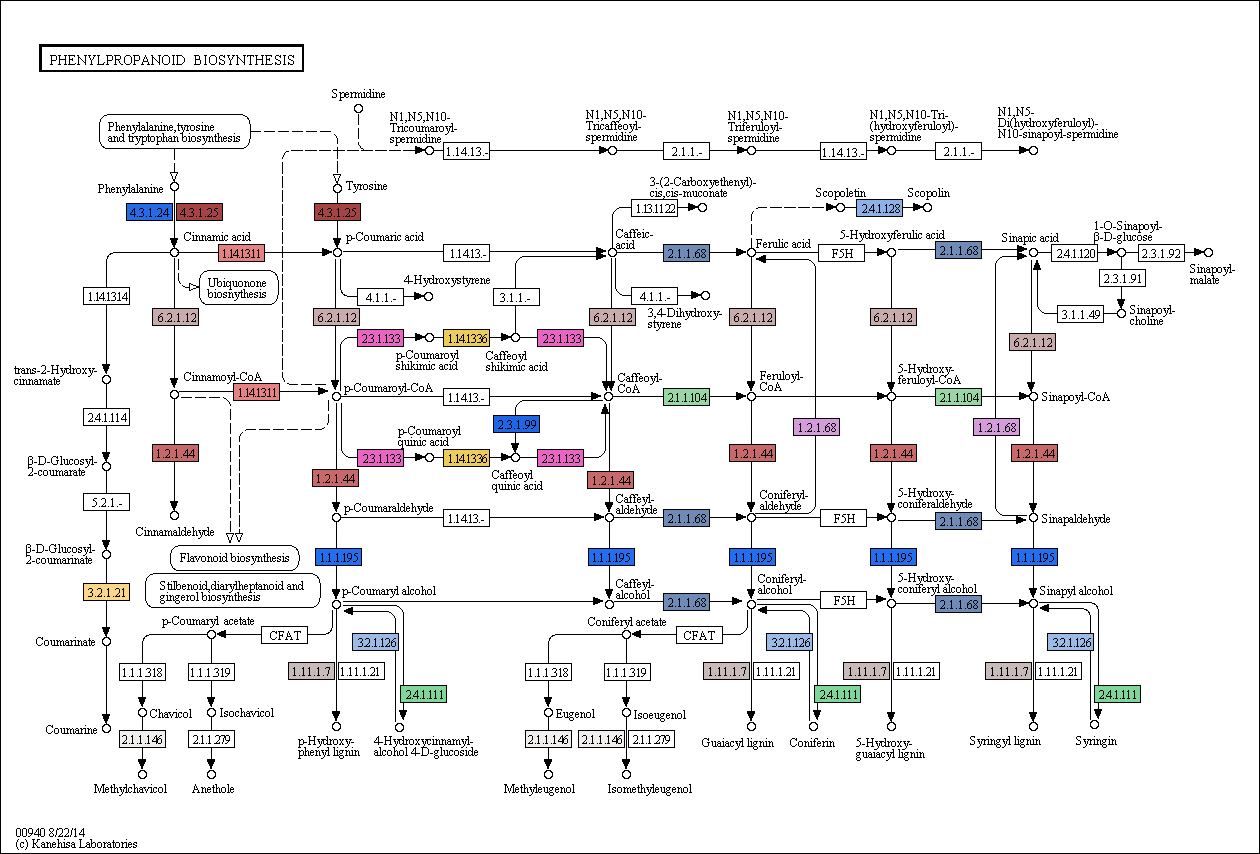

Supplement: Additional file 7: — KEGG pathway annotation. A zip compressed file with a list of KEGGs pathways, graphics in png format, and a file with a comparison with KEGGs pathways of potato and tomato. (ZIP 4361 kb) [file 12864_2016_2656_MOESM7_ESM.zip › Pathway representations/map00940_20150305161617.png]

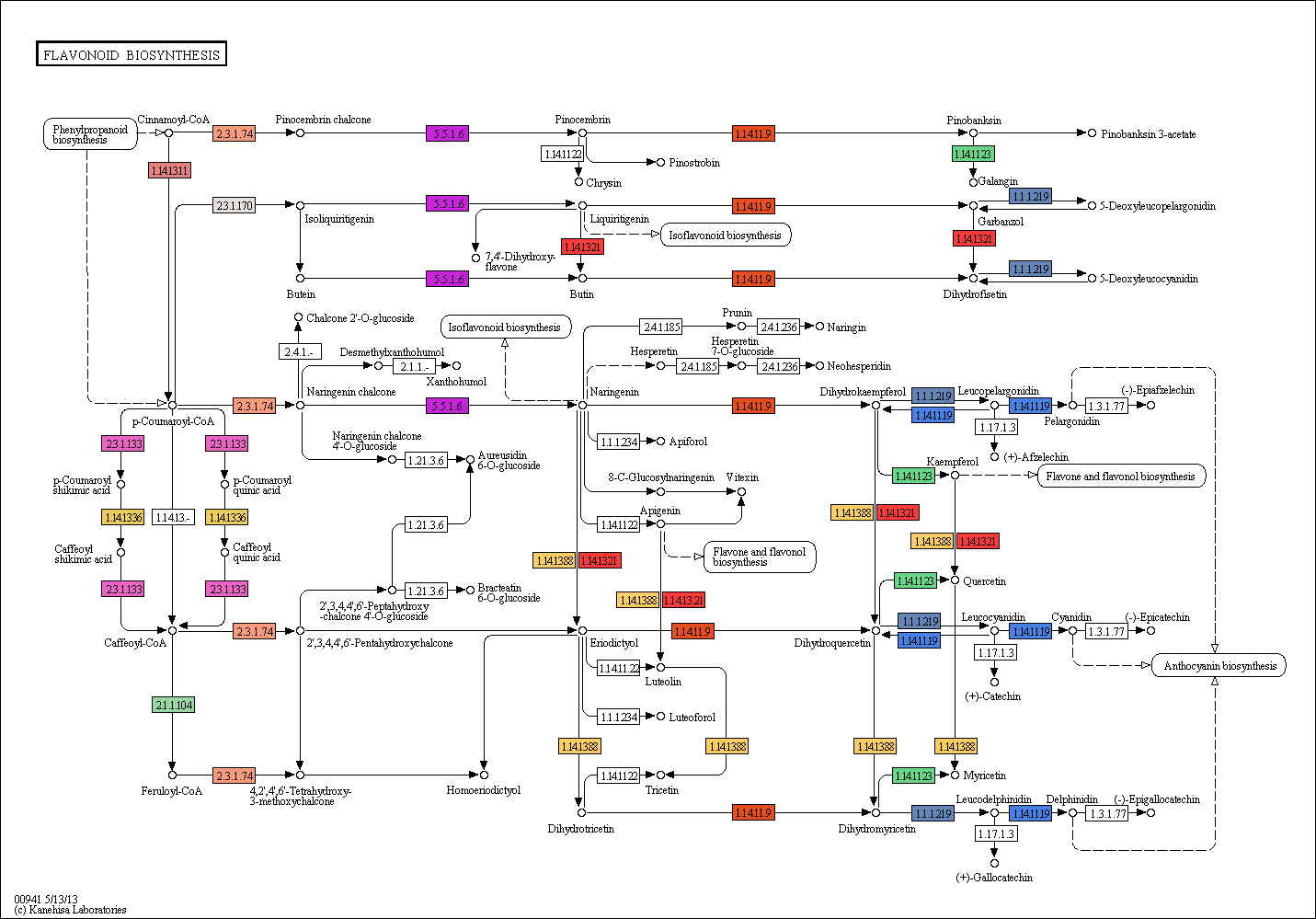

Supplement: Additional file 7: — KEGG pathway annotation. A zip compressed file with a list of KEGGs pathways, graphics in png format, and a file with a comparison with KEGGs pathways of potato and tomato. (ZIP 4361 kb) [file 12864_2016_2656_MOESM7_ESM.zip › Pathway representations/map00941_20150305161611.png]

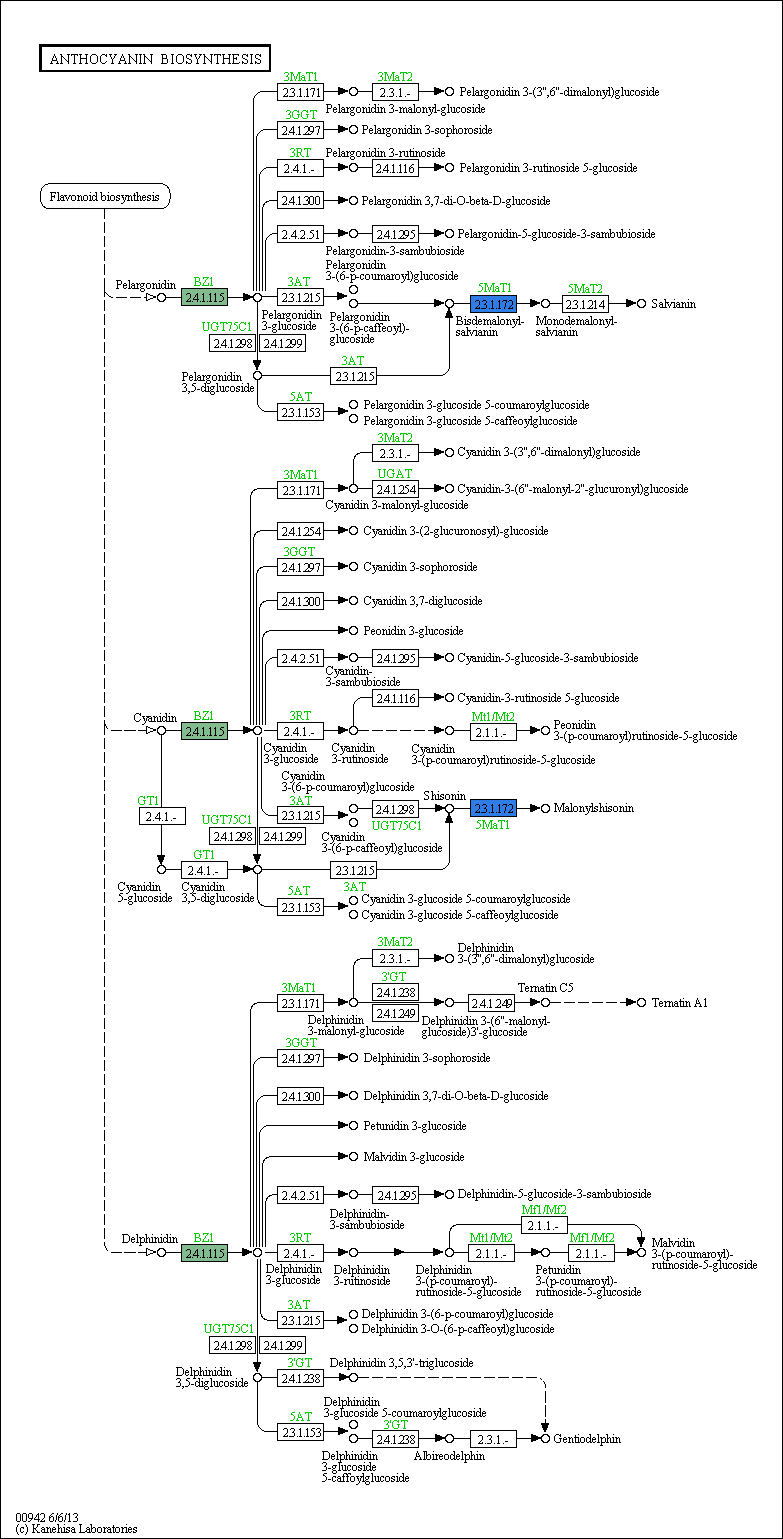

Supplement: Additional file 7: — KEGG pathway annotation. A zip compressed file with a list of KEGGs pathways, graphics in png format, and a file with a comparison with KEGGs pathways of potato and tomato. (ZIP 4361 kb) [file 12864_2016_2656_MOESM7_ESM.zip › Pathway representations/map00942_20150305161605.png]

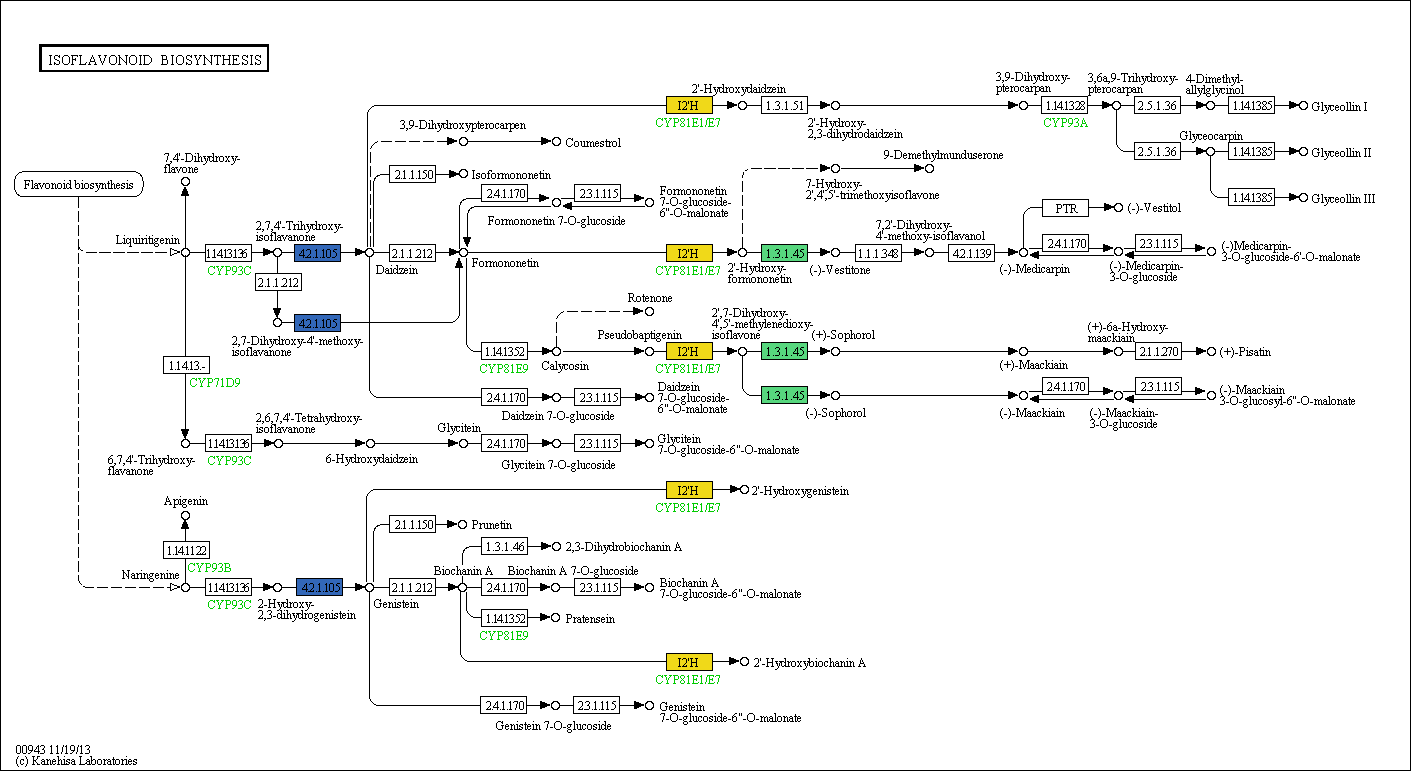

Supplement: Additional file 7: — KEGG pathway annotation. A zip compressed file with a list of KEGGs pathways, graphics in png format, and a file with a comparison with KEGGs pathways of potato and tomato. (ZIP 4361 kb) [file 12864_2016_2656_MOESM7_ESM.zip › Pathway representations/map00943_20150305161602.png]

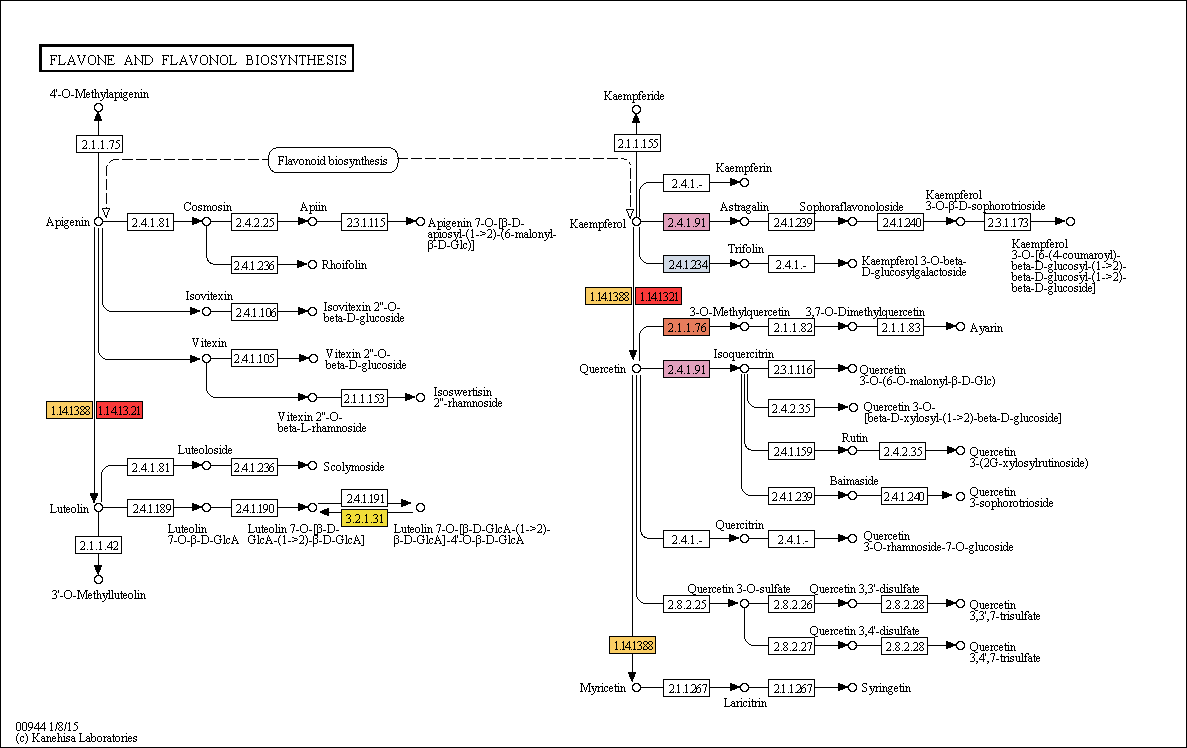

Supplement: Additional file 7: — KEGG pathway annotation. A zip compressed file with a list of KEGGs pathways, graphics in png format, and a file with a comparison with KEGGs pathways of potato and tomato. (ZIP 4361 kb) [file 12864_2016_2656_MOESM7_ESM.zip › Pathway representations/map00944_20150305161558.png]

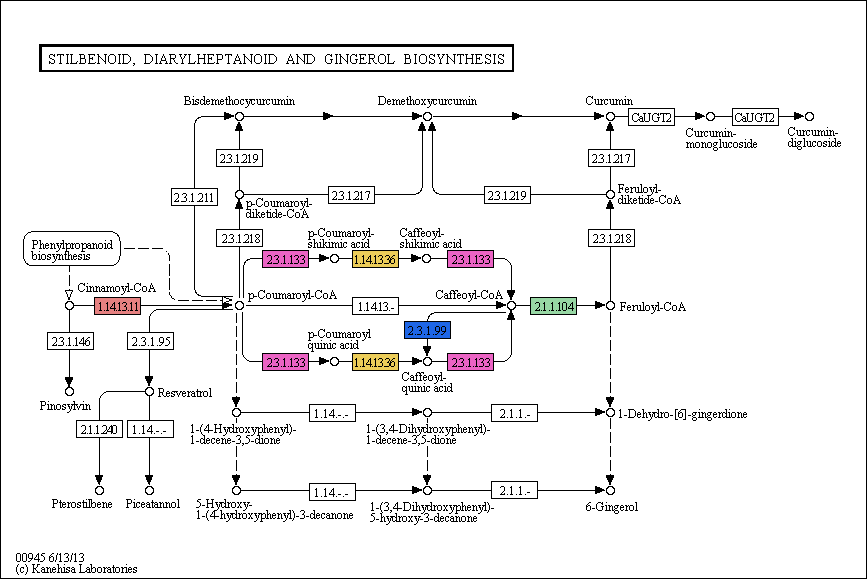

Supplement: Additional file 7: — KEGG pathway annotation. A zip compressed file with a list of KEGGs pathways, graphics in png format, and a file with a comparison with KEGGs pathways of potato and tomato. (ZIP 4361 kb) [file 12864_2016_2656_MOESM7_ESM.zip › Pathway representations/map00945_20150305161554.png]

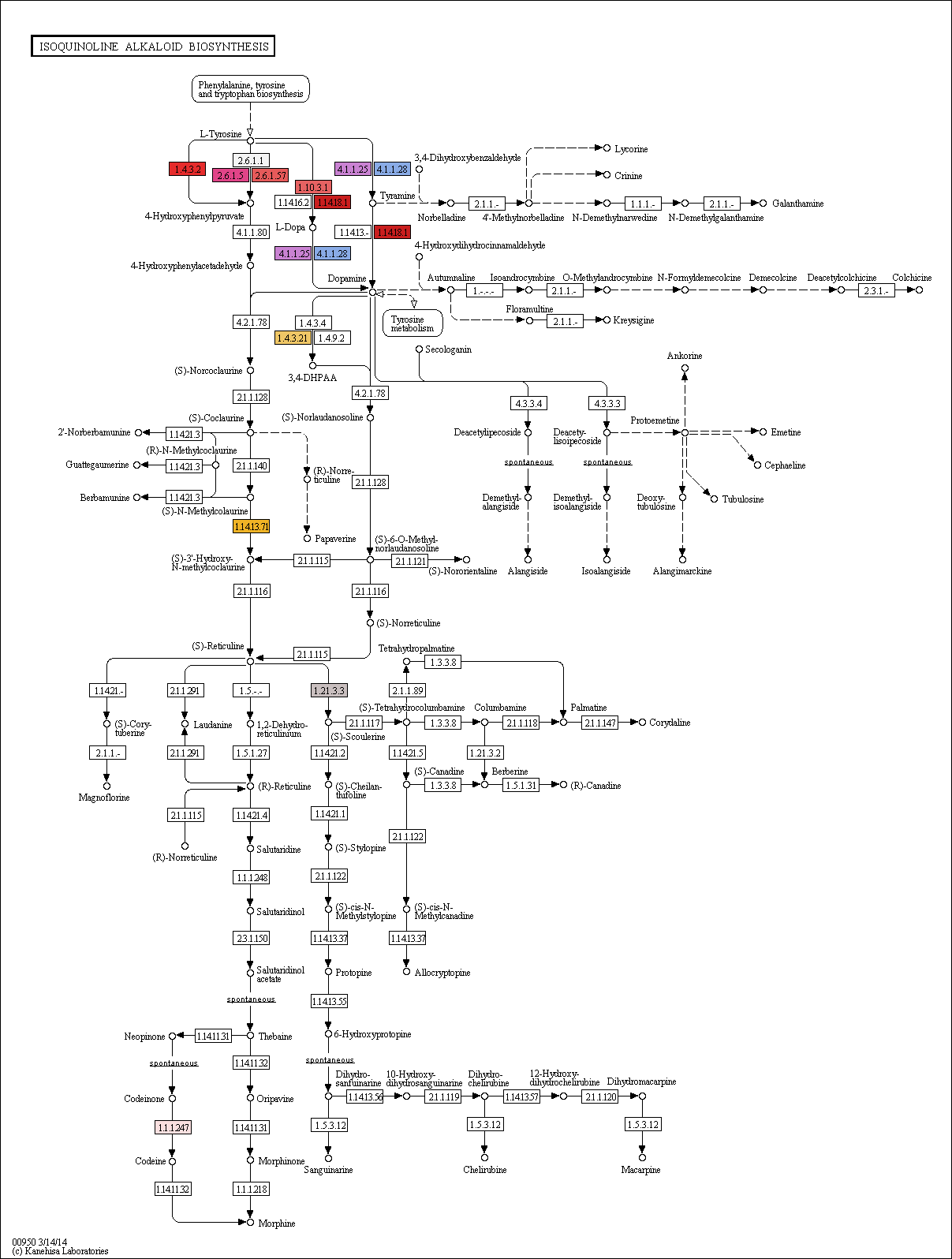

Supplement: Additional file 7: — KEGG pathway annotation. A zip compressed file with a list of KEGGs pathways, graphics in png format, and a file with a comparison with KEGGs pathways of potato and tomato. (ZIP 4361 kb) [file 12864_2016_2656_MOESM7_ESM.zip › Pathway representations/map00950_20150305161533.png]

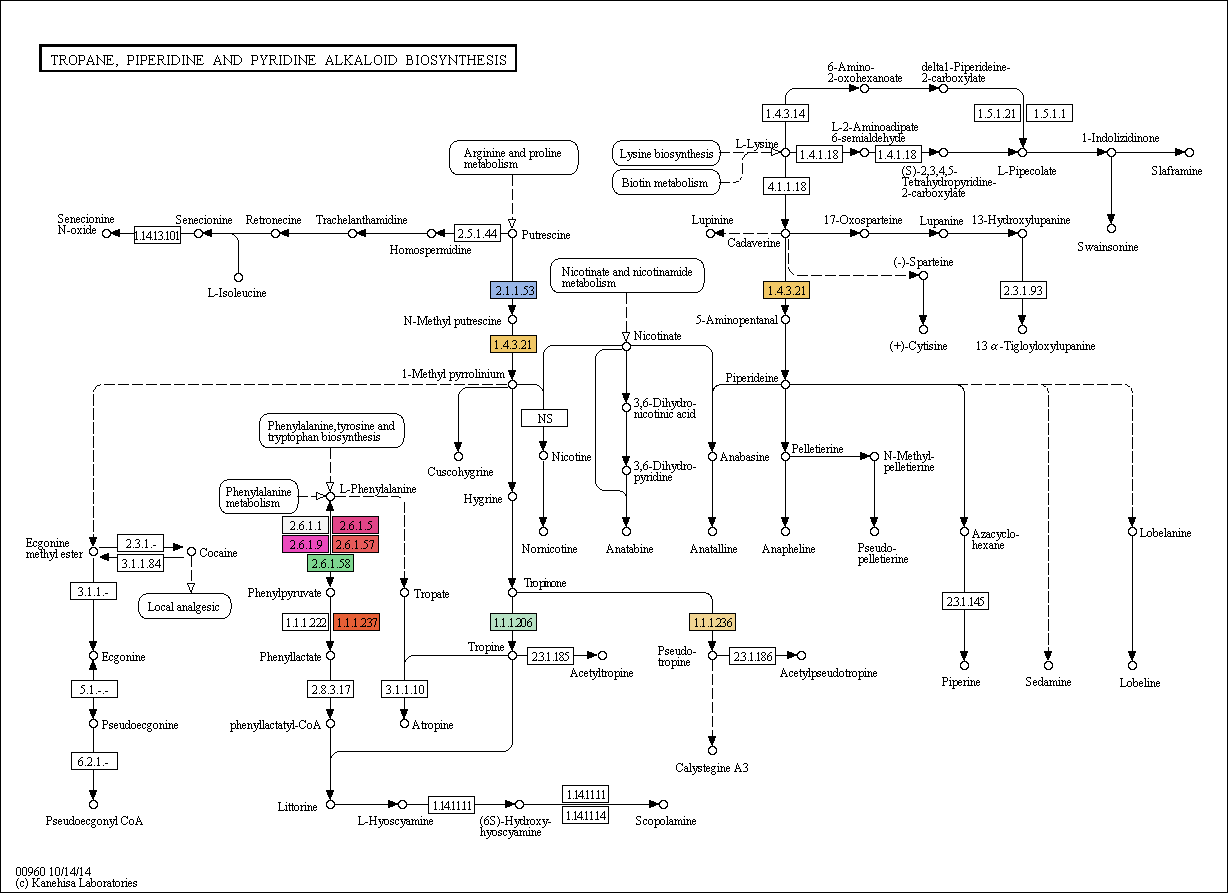

Supplement: Additional file 7: — KEGG pathway annotation. A zip compressed file with a list of KEGGs pathways, graphics in png format, and a file with a comparison with KEGGs pathways of potato and tomato. (ZIP 4361 kb) [file 12864_2016_2656_MOESM7_ESM.zip › Pathway representations/map00960_20150305161359.png]

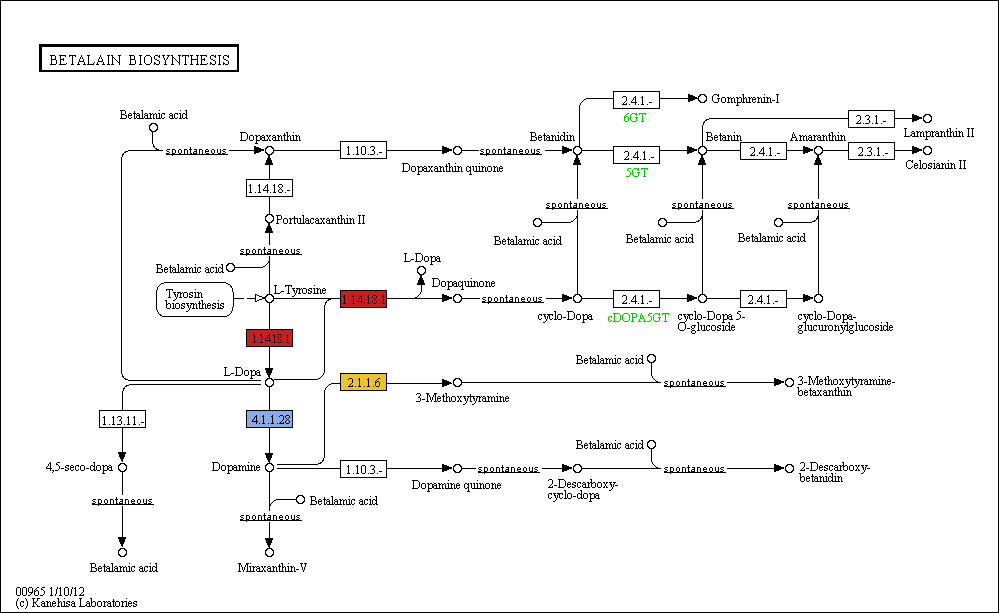

Supplement: Additional file 7: — KEGG pathway annotation. A zip compressed file with a list of KEGGs pathways, graphics in png format, and a file with a comparison with KEGGs pathways of potato and tomato. (ZIP 4361 kb) [file 12864_2016_2656_MOESM7_ESM.zip › Pathway representations/map00965_20150305161354.png]

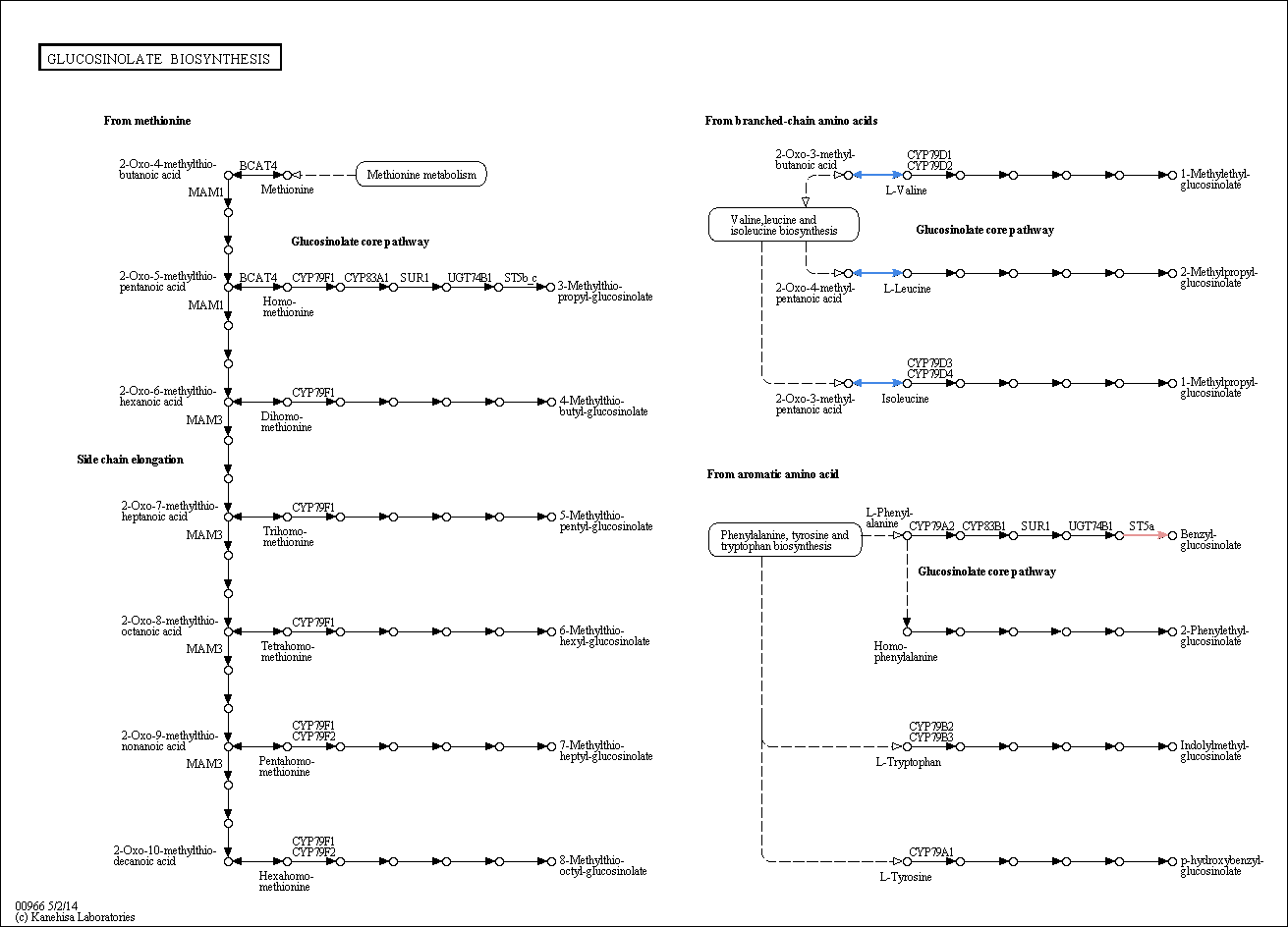

Supplement: Additional file 7: — KEGG pathway annotation. A zip compressed file with a list of KEGGs pathways, graphics in png format, and a file with a comparison with KEGGs pathways of potato and tomato. (ZIP 4361 kb) [file 12864_2016_2656_MOESM7_ESM.zip › Pathway representations/map00966_20150305161344.png]

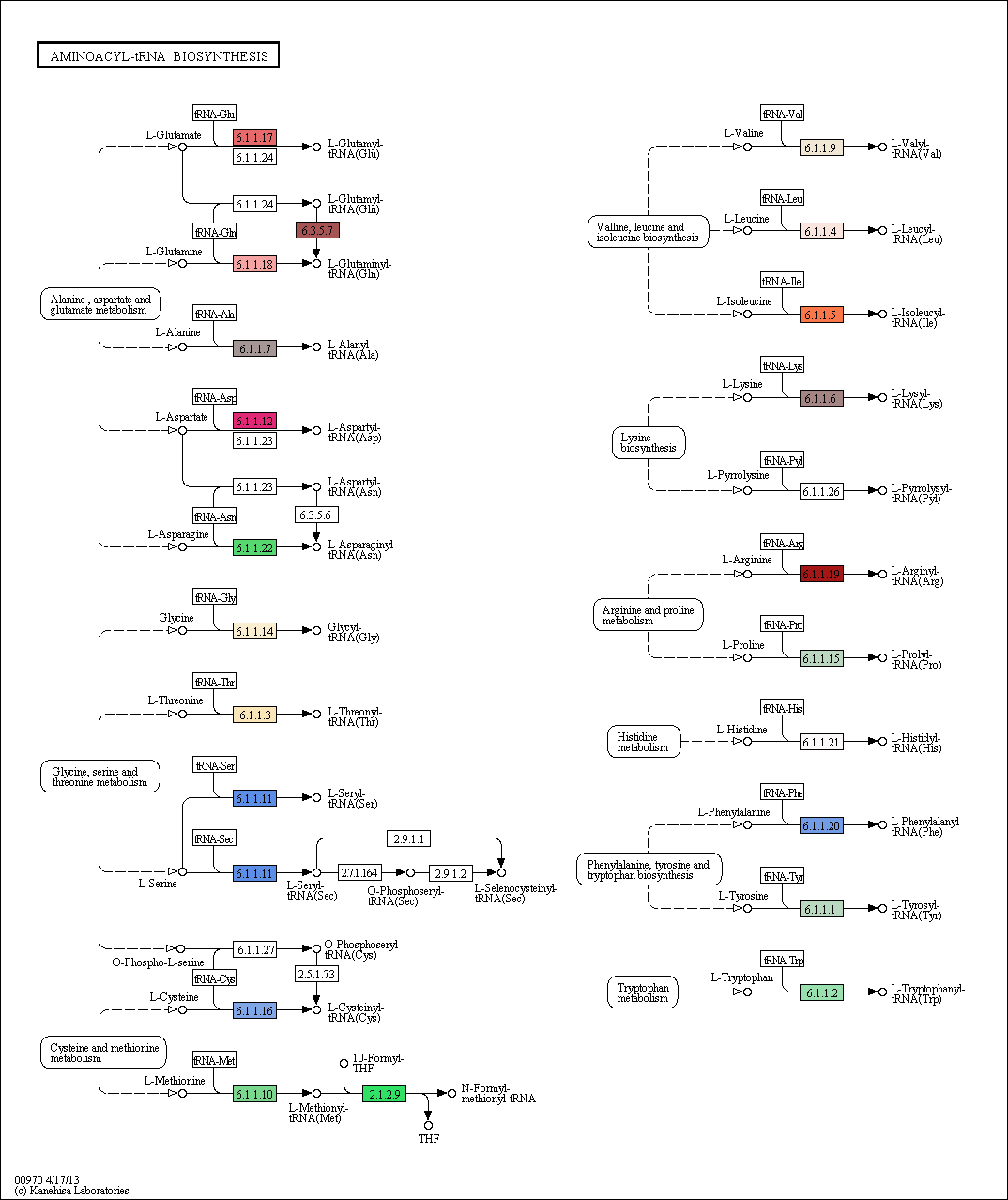

Supplement: Additional file 7: — KEGG pathway annotation. A zip compressed file with a list of KEGGs pathways, graphics in png format, and a file with a comparison with KEGGs pathways of potato and tomato. (ZIP 4361 kb) [file 12864_2016_2656_MOESM7_ESM.zip › Pathway representations/map00970_20150305161325.png]

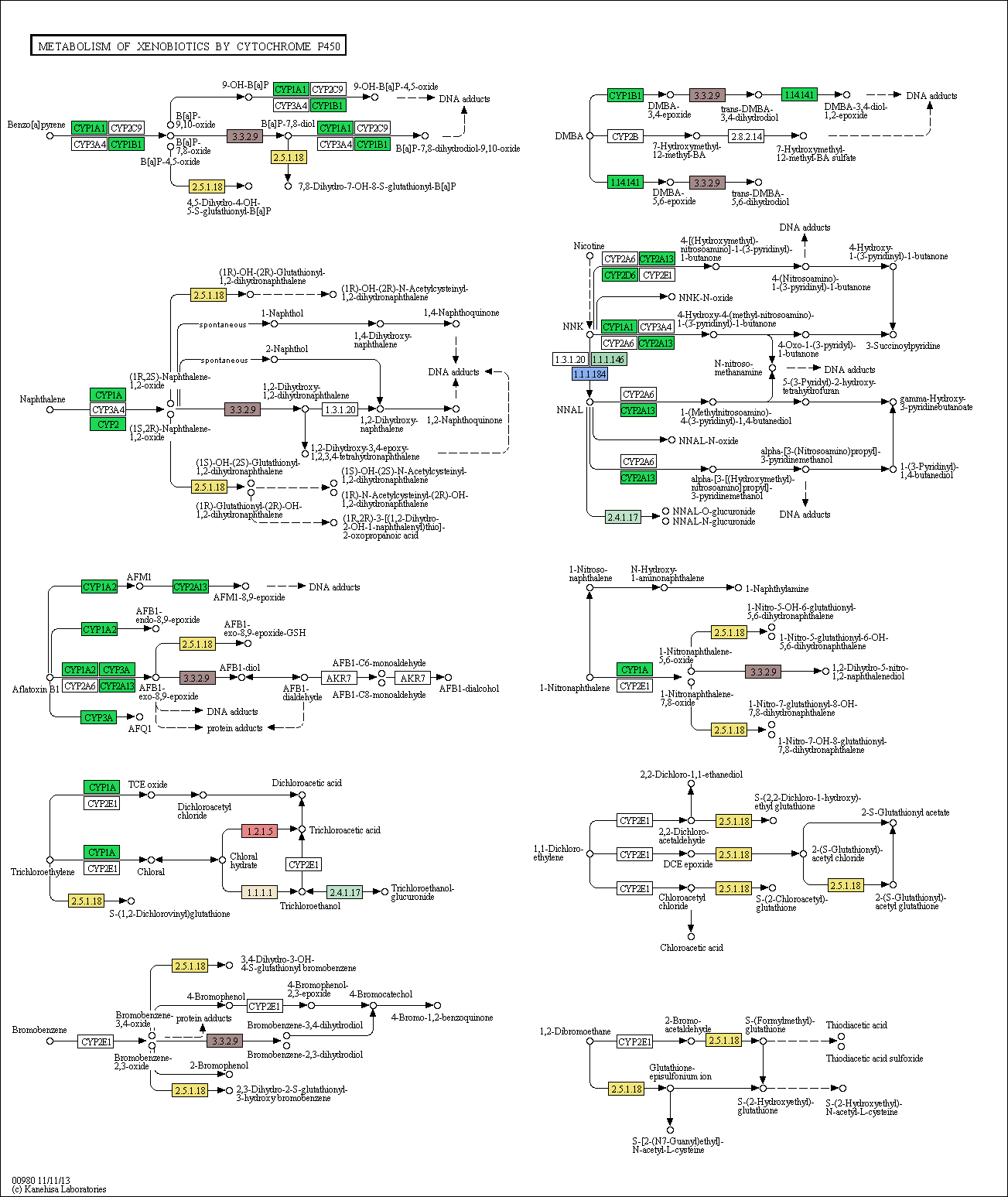

Supplement: Additional file 7: — KEGG pathway annotation. A zip compressed file with a list of KEGGs pathways, graphics in png format, and a file with a comparison with KEGGs pathways of potato and tomato. (ZIP 4361 kb) [file 12864_2016_2656_MOESM7_ESM.zip › Pathway representations/map00980_20150305161233.png]

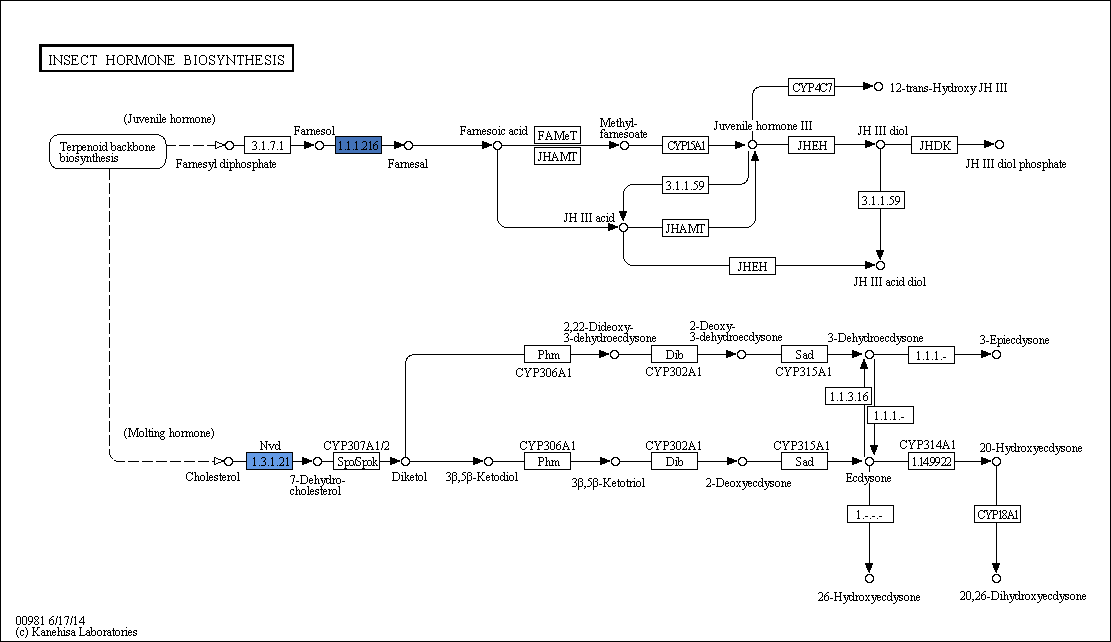

Supplement: Additional file 7: — KEGG pathway annotation. A zip compressed file with a list of KEGGs pathways, graphics in png format, and a file with a comparison with KEGGs pathways of potato and tomato. (ZIP 4361 kb) [file 12864_2016_2656_MOESM7_ESM.zip › Pathway representations/map00981_20150305161223.png]

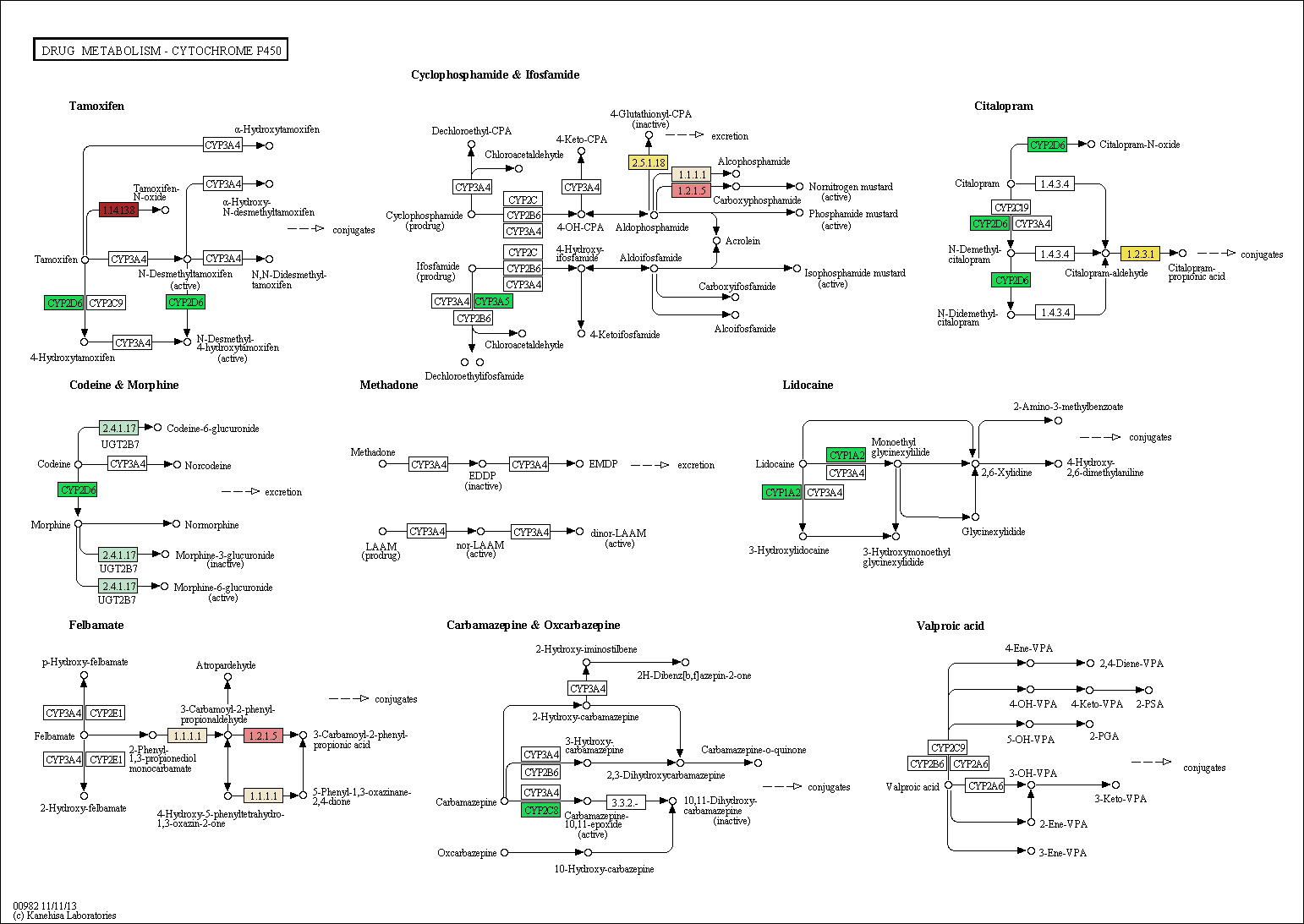

Supplement: Additional file 7: — KEGG pathway annotation. A zip compressed file with a list of KEGGs pathways, graphics in png format, and a file with a comparison with KEGGs pathways of potato and tomato. (ZIP 4361 kb) [file 12864_2016_2656_MOESM7_ESM.zip › Pathway representations/map00982_20150305161216.png]

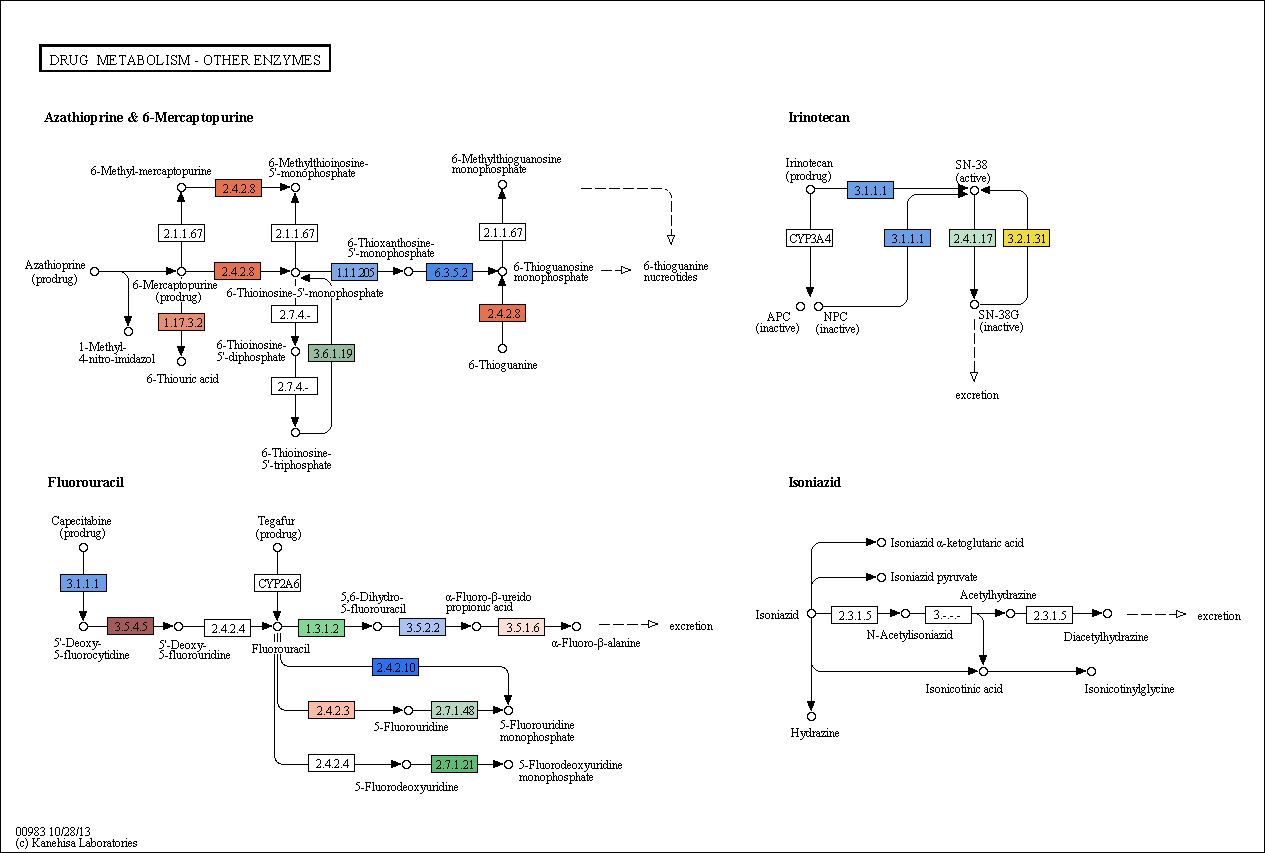

Supplement: Additional file 7: — KEGG pathway annotation. A zip compressed file with a list of KEGGs pathways, graphics in png format, and a file with a comparison with KEGGs pathways of potato and tomato. (ZIP 4361 kb) [file 12864_2016_2656_MOESM7_ESM.zip › Pathway representations/map00983_20150305161205.png]

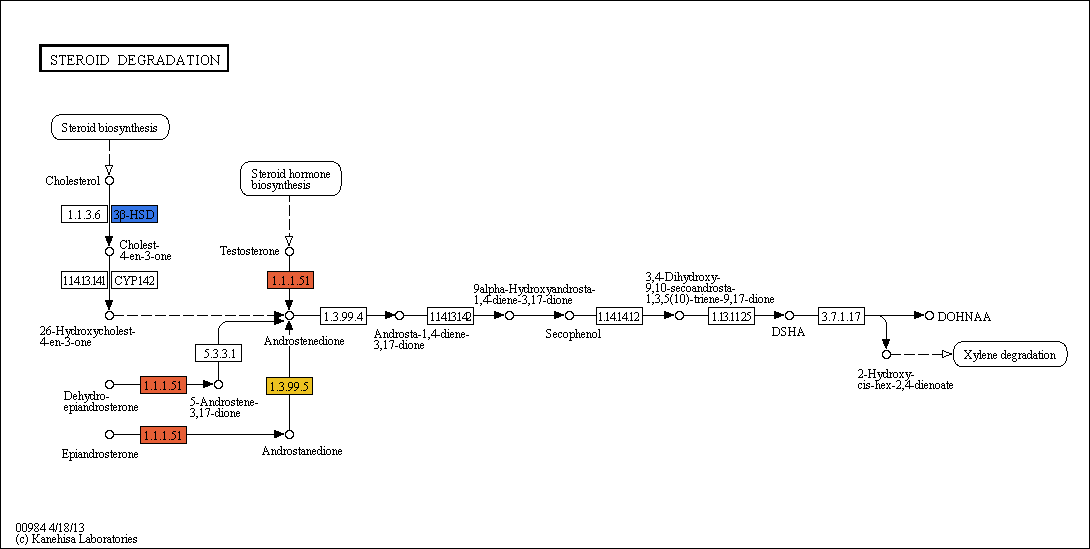

Supplement: Additional file 7: — KEGG pathway annotation. A zip compressed file with a list of KEGGs pathways, graphics in png format, and a file with a comparison with KEGGs pathways of potato and tomato. (ZIP 4361 kb) [file 12864_2016_2656_MOESM7_ESM.zip › Pathway representations/map00984_20150305161154.png]

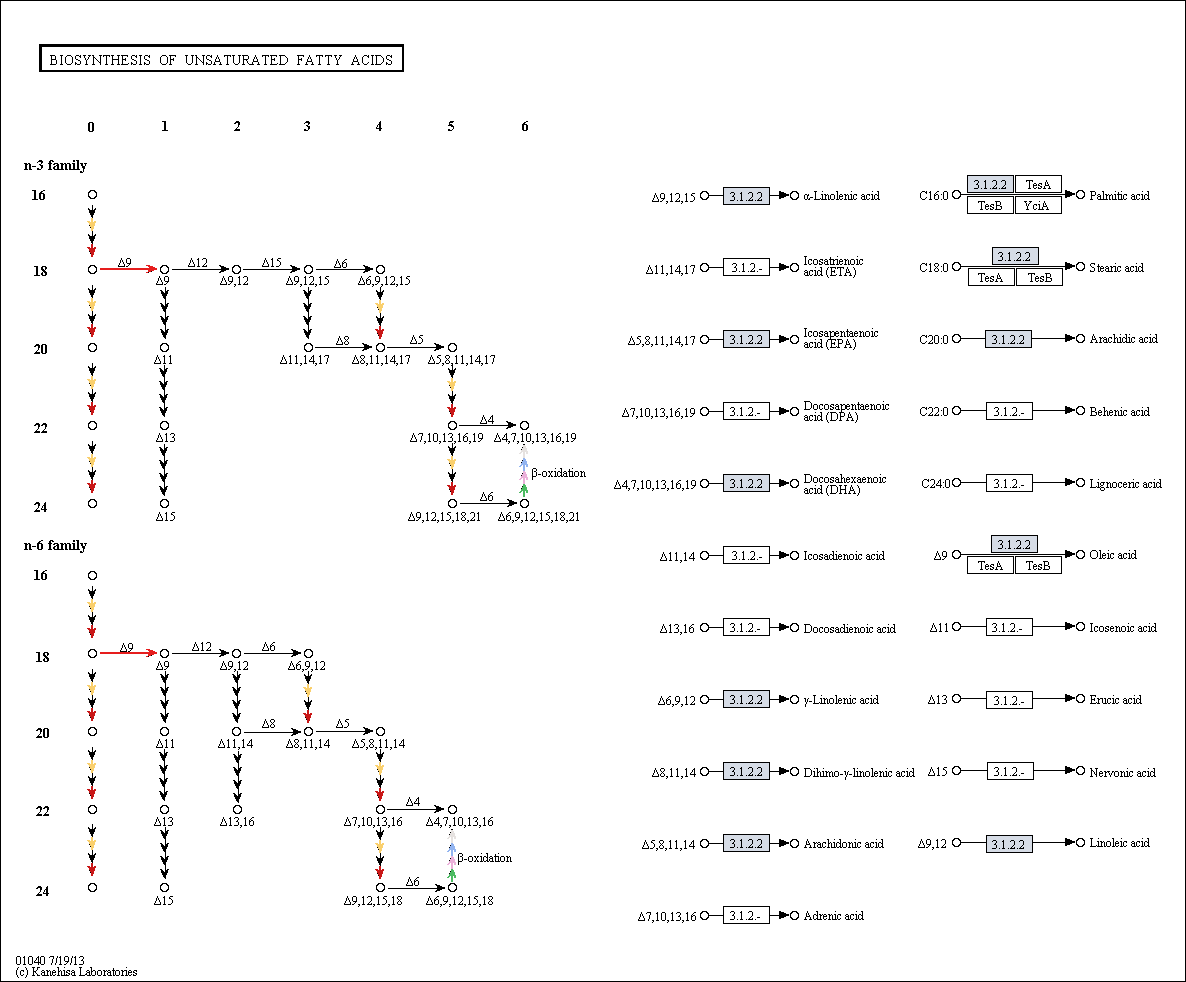

Supplement: Additional file 7: — KEGG pathway annotation. A zip compressed file with a list of KEGGs pathways, graphics in png format, and a file with a comparison with KEGGs pathways of potato and tomato. (ZIP 4361 kb) [file 12864_2016_2656_MOESM7_ESM.zip › Pathway representations/map01040_20150305161342.png]

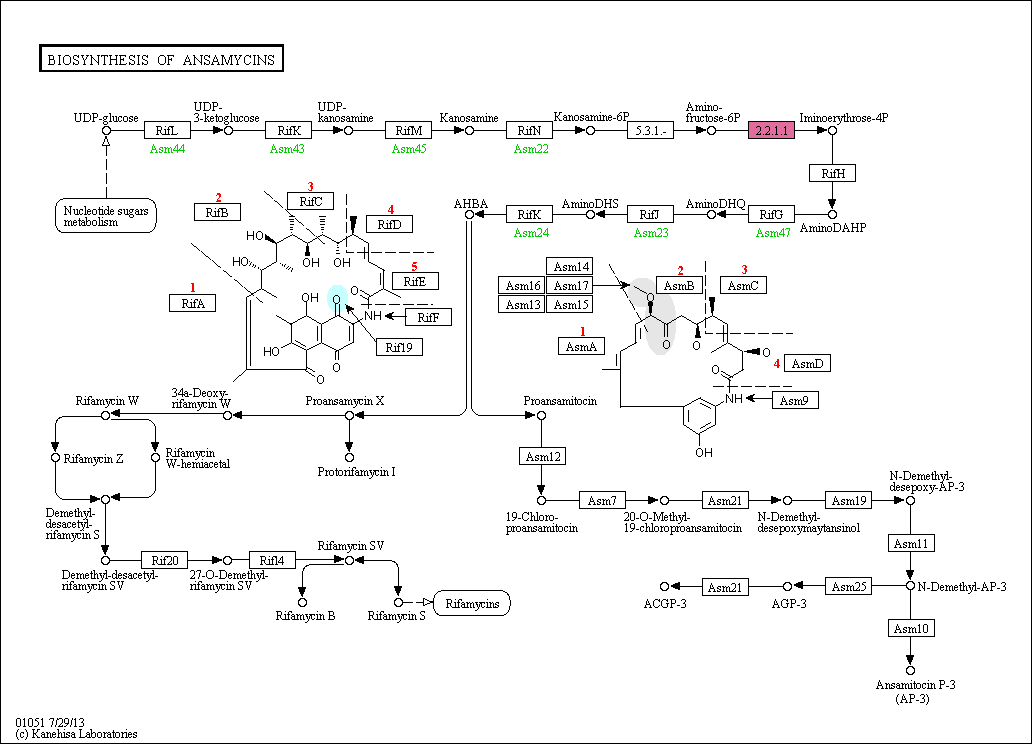

Supplement: Additional file 7: — KEGG pathway annotation. A zip compressed file with a list of KEGGs pathways, graphics in png format, and a file with a comparison with KEGGs pathways of potato and tomato. (ZIP 4361 kb) [file 12864_2016_2656_MOESM7_ESM.zip › Pathway representations/map01051_20150305161304.png]

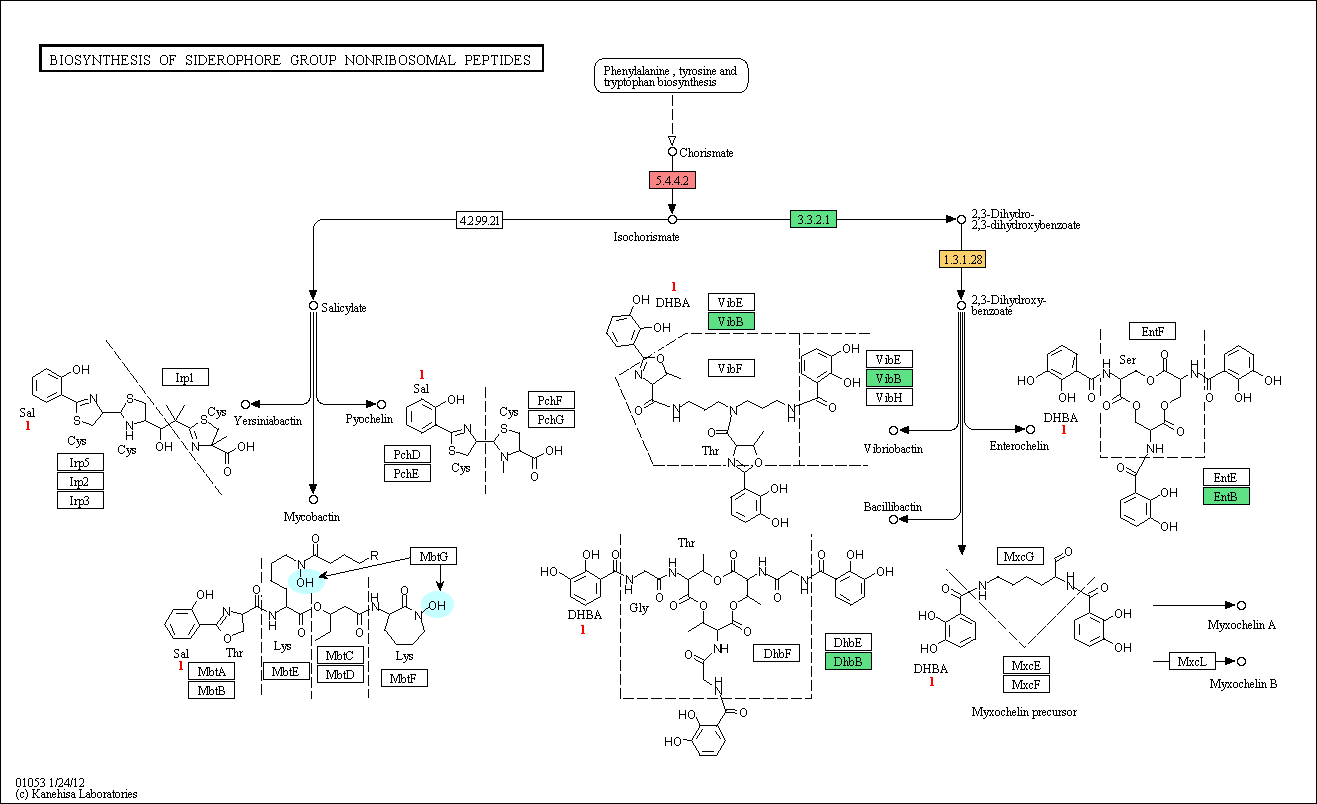

Supplement: Additional file 7: — KEGG pathway annotation. A zip compressed file with a list of KEGGs pathways, graphics in png format, and a file with a comparison with KEGGs pathways of potato and tomato. (ZIP 4361 kb) [file 12864_2016_2656_MOESM7_ESM.zip › Pathway representations/map01053_20150305161301.png]

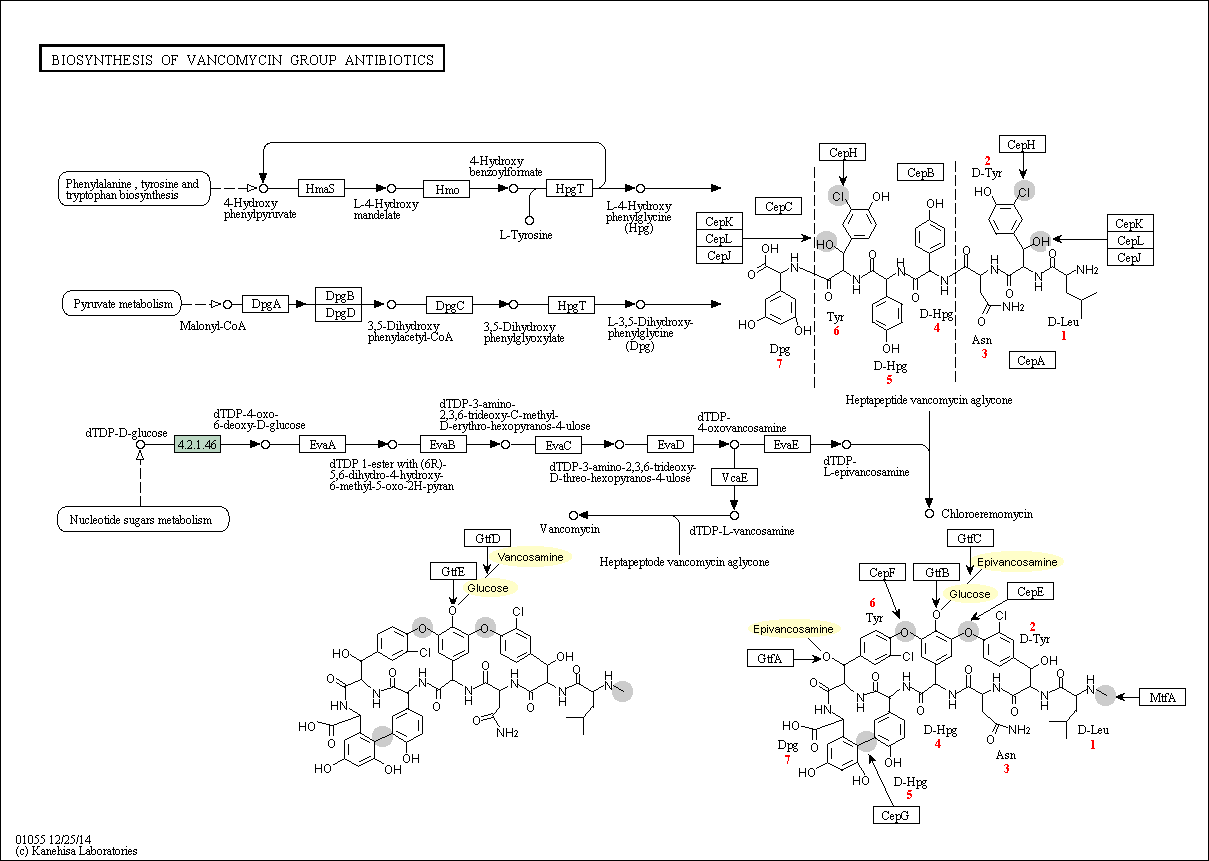

Supplement: Additional file 7: — KEGG pathway annotation. A zip compressed file with a list of KEGGs pathways, graphics in png format, and a file with a comparison with KEGGs pathways of potato and tomato. (ZIP 4361 kb) [file 12864_2016_2656_MOESM7_ESM.zip › Pathway representations/map01055_20150305161257.png]

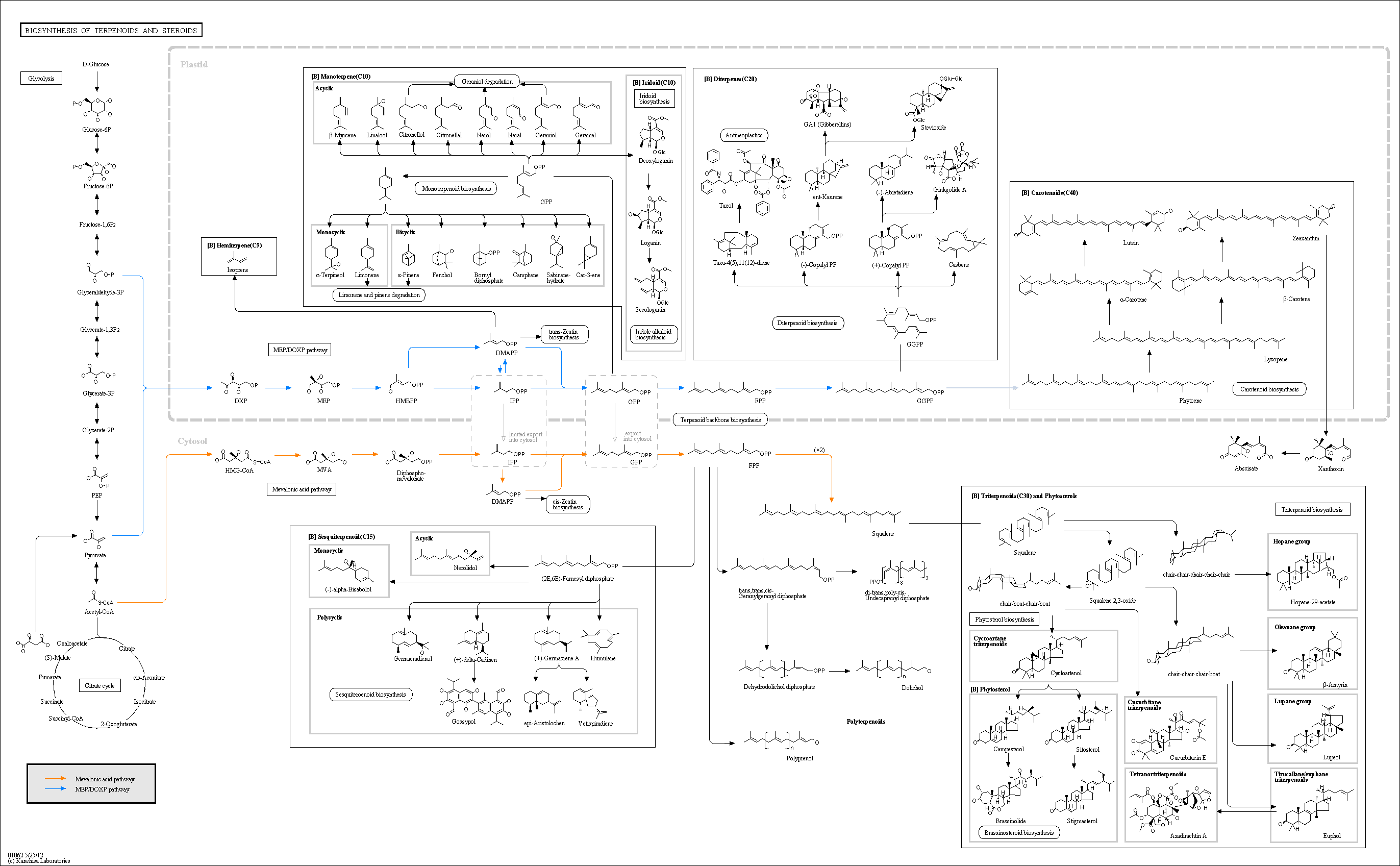

Supplement: Additional file 7: — KEGG pathway annotation. A zip compressed file with a list of KEGGs pathways, graphics in png format, and a file with a comparison with KEGGs pathways of potato and tomato. (ZIP 4361 kb) [file 12864_2016_2656_MOESM7_ESM.zip › Pathway representations/map01062_20150305161119.png]

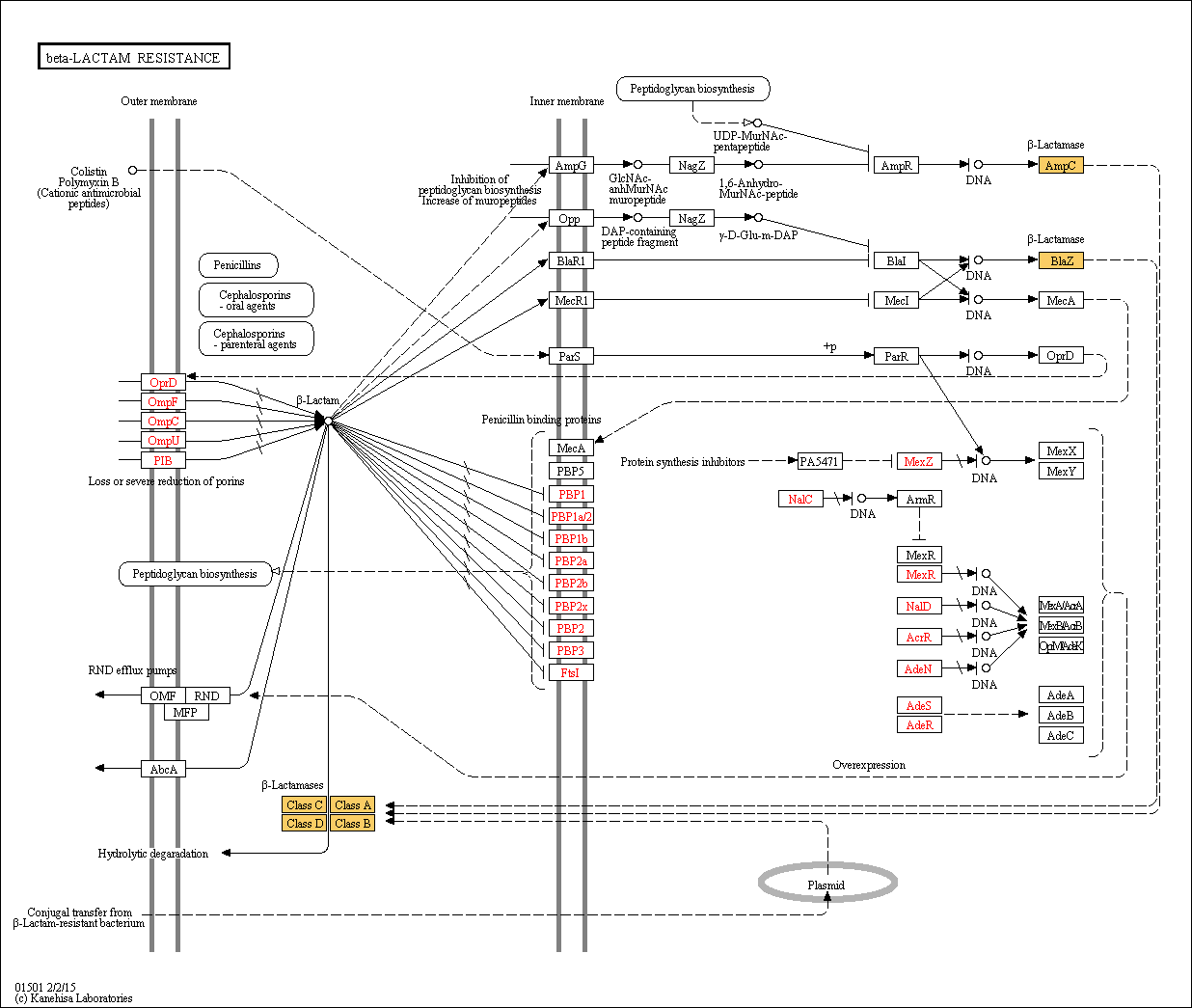

Supplement: Additional file 7: — KEGG pathway annotation. A zip compressed file with a list of KEGGs pathways, graphics in png format, and a file with a comparison with KEGGs pathways of potato and tomato. (ZIP 4361 kb) [file 12864_2016_2656_MOESM7_ESM.zip › Pathway representations/map01501_20150305161037.png]

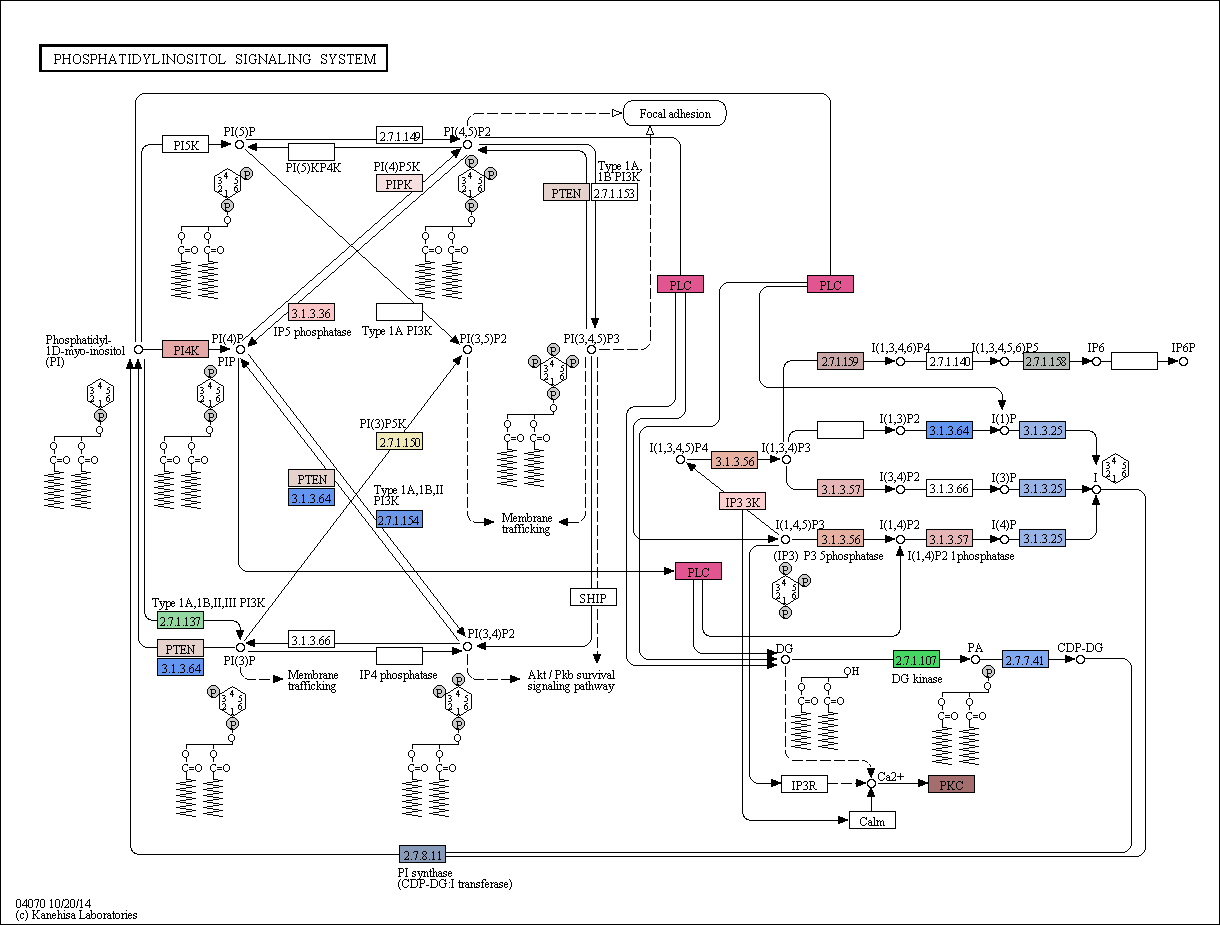

Supplement: Additional file 7: — KEGG pathway annotation. A zip compressed file with a list of KEGGs pathways, graphics in png format, and a file with a comparison with KEGGs pathways of potato and tomato. (ZIP 4361 kb) [file 12864_2016_2656_MOESM7_ESM.zip › Pathway representations/map04070_20150305160601.png]

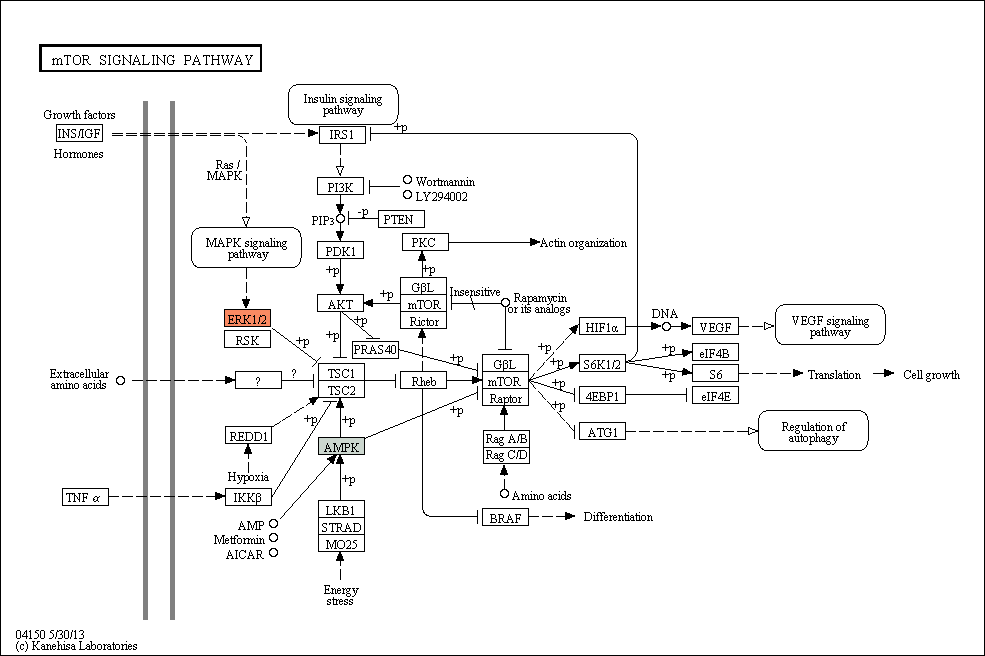

Supplement: Additional file 7: — KEGG pathway annotation. A zip compressed file with a list of KEGGs pathways, graphics in png format, and a file with a comparison with KEGGs pathways of potato and tomato. (ZIP 4361 kb) [file 12864_2016_2656_MOESM7_ESM.zip › Pathway representations/map04150_20150305161422.png]

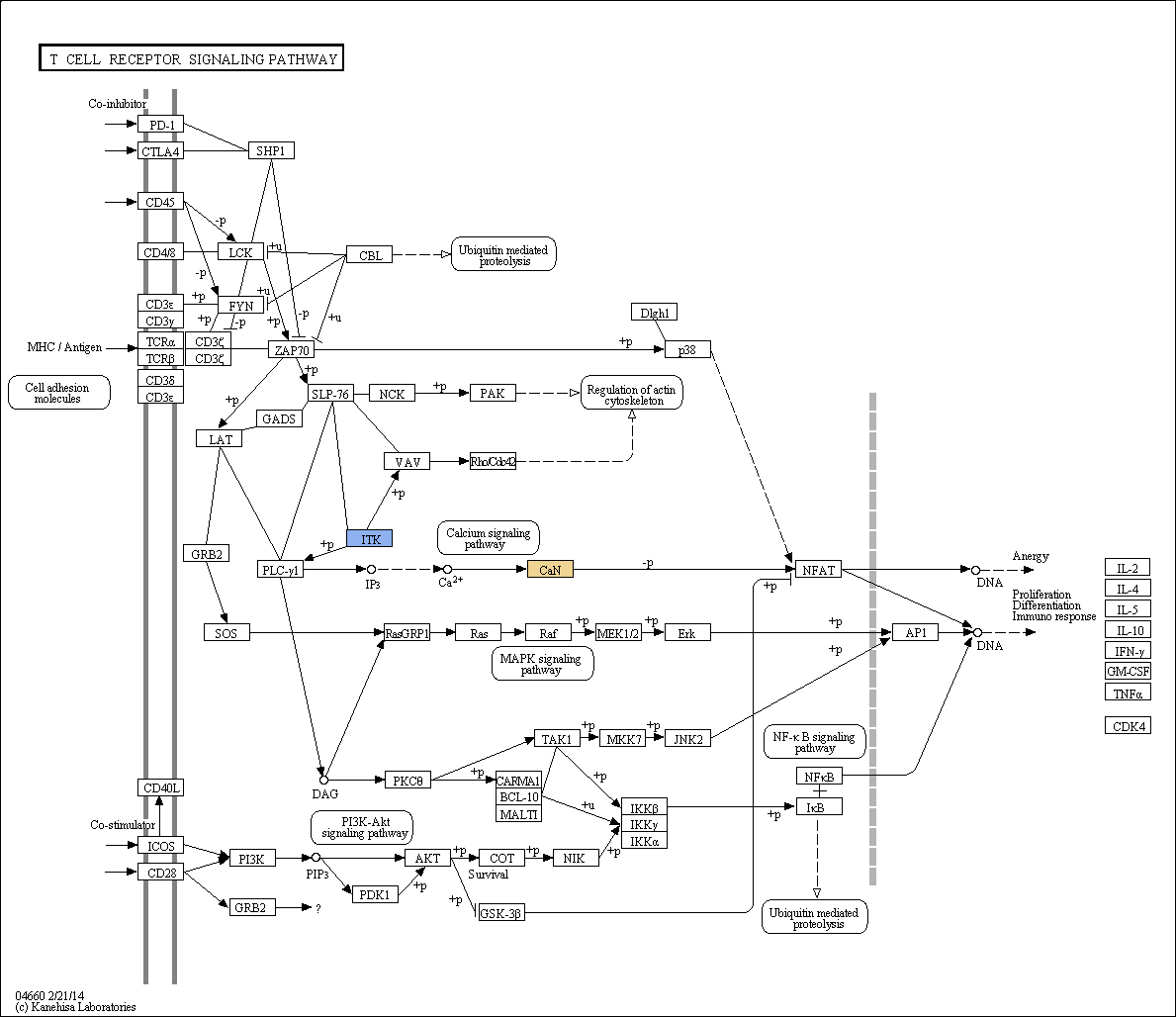

Supplement: Additional file 7: — KEGG pathway annotation. A zip compressed file with a list of KEGGs pathways, graphics in png format, and a file with a comparison with KEGGs pathways of potato and tomato. (ZIP 4361 kb) [file 12864_2016_2656_MOESM7_ESM.zip › Pathway representations/map04660_20150305160610.png]
