# Supplementary material for: The first de novo transcriptome of pepino (Solanum muricatum): assembly, comprehensive analysis and comparison with the closely related species S. caripense, potato and tomato
Source: BMC Genomics. 2016 May 4;17:321. doi: 10.1186/s12864-016-2656-8 (PMC4855764; doi:10.1186/s12864-016-2656-8)
Supplement: Additional file 8: Table S1. — Candidate genes list and features. A Word file with a description of the candidate genes used, the name of the origin sequences, the name of the pepino unigenes and the number of variants in these unigenes between pepino and S. caripense. (DOCX 26 kb) [file 12864_2016_2656_MOESM8_ESM.docx]

| Character description | Genes | Databases sequence name | Reference | Features | Pepino unigenes | Score (bits) | E value | Number SNVs muricatum / caripense |
| --- | --- | --- | --- | --- | --- | --- | --- | --- |
| Inflorescence | Anantha (an) | gi\|207083720\|gb\|FJ190668.1 | Lippman *et al.* 2008 | Gene - F-box protein | No hits | - | - | - |
|  | Compound inflorescence (S) | gi\|207083718\|gb\|FJ190667.1 | Lippman *et al.* 2008 | Transcription factor | SMUC52767_TC01 | 464 | e^-129^ | 1 |
| Fruit Stripes | Fruit Stripes | Solyc10g008160.2.1 | Lam Cheng, KL 2013 | Transcription factor | SMUC34152_TC01 | 688 | 0 | 20 |
| Fruit Shape | FAS | gi\|350539380\|ref\|NM_001247461.1 | Wang *et al.* 2015 | Intron-regulatory | SMUC25785_TC01 | 624 | e^-178^ | 5 |
|  | FAS family | Solyc01g010240.2.1 | Wang *et al.* 2015 | Intron-regulatory | SMUC27499_TC01 | 549 | e^-155^ | 1 |
|  |  | Solyc01g091010.2.1 | Wang *et al.* 2015 | Intron-regulatory | SMUC18209_TC01 | 664 | 0 | 2 |
|  |  | Solyc05g005240.1.1 | Wang *et al.* 2015 | Intron-regulatory | SMUC38925_TC01 | 361 | 8e^-99^ | 2 |
|  |  | Solyc05g012050.2.1 | Wang *et al.* 2015 | Intron-regulatory | SMUC27500_TC01 | 731 | 0 | 12 |
|  |  | Solyc06g073920.2.1 | Wang *et al.* 2015 | Intron-regulatory | SMUC09999_TC01 | 182 | e^-135^ | 4 |
|  |  | Solyc07g008180.2.1 | Wang *et al.* 2015 | Intron-regulatory | SMUC21560_TC01 | 868 | 0 | 1 |
|  |  | Solyc08g079100.2.1 | Wang *et al.* 2015 | Intron-regulatory | SMUC13041_TC01 | 726 | 0 | 11 |
|  |  | Solyc11g071810.1.1 | Wang *et al.* 2015 | Intron-regulatory | SMUC25785_TC01 | 584 | e^-167^ | 5 |
|  |  | Solyc12g009580.1.1 | Wang *et al.* 2015 | Intron-regulatory | SMUC21559_TC01 | 896 | 0 | 2 |
|  | Fw2.2 | Solyc02g090730.2.1 | Wang *et al.* 2015 | Promoter-regulatory | SMUC05517_TC01 | 739 | 0 | 0 |
|  | Fw3 | Solyc03g114940.2.1 | Wang *et al.* 2015 | Promoter-regulatory | SMUC09078_TC01 | 1707 | 0 | 13 |
|  | Tonneau-1 | gi\|77745510\|gb\|DQ222523.1 | Wang *et al.* 2015 | Gene-unknown function | SMUC10029_TC01 | 666 | 0 | 1 |
|  | Wuschel (LC) | Solyc02g083950.2.1 | Wang *et al.* 2015 | SNP in downstream-regulatory | No hits | - | - | - |
|  | OVATE | gi\|350535934\|ref\|NM_001247292.1 | Wang *et al.* 2015 | Premature stop | SMUC15651_TC01 | 906 | 0 | 4 |
|  | OVATE family | Solyc01g007800.2.1 | Wang *et al.* 2015 | Premature stop | SMUC16951_TC01 | 398 | e^-110^ | 1 |
|  |  | Solyc01g007810.1.1 | Wang *et al.* 2015 | Premature stop | SMUC26723_TC01 | 817 | 0 | 6 |
|  |  | Solyc02g072030.1.1 | Wang *et al.* 2015 | Premature stop | SMUC03225_TC01 | 775 | 0 | 0 |
|  |  | Solyc03g034100.2.1 | Wang *et al.* 2015 | Premature stop | SMUC39086_TC01 | 696 | 0 | 0 |
|  |  | Solyc03g120190.2.1 | Wang *et al.* 2015 | Premature stop | SMUC47616_TC01 | 509 | e^-143^ | 5 |
|  |  | Solyc03g120790.1.1 | Wang *et al.* 2015 | Premature stop | SMUC34033_TC01 | 1098 | 0 | 1 |
|  |  | Solyc04g080210.1.1 | Wang *et al.* 2015 | Premature stop | No hits | - | - | - |
|  |  | Solyc05g055220.1.1 | Wang *et al.* 2015 | Premature stop | SMUC35558_TC01 | 866 | 0 | 3 |
|  |  | Solyc06g074020.2.1 | Wang *et al.* 2015 | Premature stop | SMUC37289_TC01 | 414 | e^-115^ | 5 |
|  |  | Solyc06g082450.1.1 | Wang *et al.* 2015 | Premature stop | No hits | - | - | - |
|  |  | Solyc06g082460.1.1 | Wang *et al.* 2015 | Premature stop | SMUC36757_TC01 | 1602 | 0 | 4 |
|  |  | Solyc07g055240.1.1 | Wang *et al.* 2015 | Premature stop | SMUC55447 | 323 | 2e^-87^ | 0 |
|  |  | Solyc08g068170.1.1 | Wang *et al.* 2015 | Premature stop | SMUC62672_TC01 | 452 | e^-126^ | 0 |
|  |  |  | Wang *et al.* 2015 |  | SMUC69341_TC01 | 389 | e^-107^ | 0 |
|  |  | Solyc09g018200.1.1 | Wang *et al.* 2015 | Premature stop | No hits | - | - | - |
|  |  | Solyc09g082080.1.1 | Wang *et al.* 2015 | Premature stop | SMUC03298_TC01 | 519 | e^-146^ | 3 |
|  |  | Solyc10g076180.1.1 | Wang *et al.* 2015 | Premature stop | SMUC09834_TC01 | 605 | e^-172^ | 9 |
|  |  | Solyc10g082050.1.1 | Wang *et al.* 2015 | Premature stop | SMUC16957_TC01 | 626 | e^-179^ | 17 |
|  |  | Solyc10g082060.1.1 | Wang *et al.* 2015 | Premature stop | SMUC13781_TC01 | 373 | e^-102^ | 2 |
|  |  | Solyc10g083070.1.1 | Wang *et al.* 2015 | Premature stop | No hits | - | - | - |
|  |  | Solyc10g083080.1.1 | Wang *et al.* 2015 | Premature stop | No hits | - | - | - |
|  |  | Solyc10g083090.1.1 | Wang *et al.* 2015 | Premature stop | No hits | - | - | - |
|  |  | Solyc10g083100.1.1 | Wang *et al.* 2015 | Premature stop | No hits | - | - | - |
|  |  | Solyc11g006670.1.1 | Wang *et al.* 2015 | Premature stop | SMUC22499_TC01 | 424 | e^-118^ | 10 |
|  |  | Solyc11g068780.1.1 | Wang *et al.* 2015 | Premature stop | SMUC20635_TC01 | 474 | e^-133^ | 6 |
|  |  | SlOFP4-SL1.00sc02618_4.1.1 | Wang *et al.* 2015 | Premature stop | No hits | - | - | - |
|  |  | SlOFP11-Solyc06g073040 | Wang *et al.* 2015 | Premature stop | SMUC35427_TC01 | 389 | e^-107^ | 4 |
|  |  | SlOFP18-Solyc09g065350-LA1589 | Wang *et al.* 2015 | Premature stop | SMUC18168_TC01 | 644 | 0 | 14 |
|  |  | SlOFP24 | Wang *et al.* 2015 | Premature stop | No hits | - | - | - |
|  |  | SlOFP25 | Wang *et al.* 2015 | Premature stop | No hits | - | - | - |
|  |  | SlOFP31-SL1.00sc03540_201.1.1 | Wang *et al.* 2015 | Premature stop | SMUC11435_TC01 | 644 | 0 | 3 |
|  | POS1 | Solyc01g008880.1.1 | Wang *et al.* 2015 | Intron-regulatory | No hits | - | - | - |
|  | SlCCS52A | Solyc08g080080.2.1 | Wang *et al.* 2015 | Receptor activity | SMUC28194_TC01 | 2932 | 0 | 9 |
|  | Sl-IAA17 | gi\|365818542\|gb\|JN379444.1 | Wang *et al.* 2015 | Transcription factor | SMUC17629_TC01 | 494 | e^-139^ | 2 |
|  | SUN | gi\|350537692\|ref\|NM_001246864.1 | Wang *et al.* 2015 | Transposon insertion-regulatory | SMUC36402_TC01 | 702 | 0 | 1 |
|  |  |  | Wang *et al.* 2015 |  | SMUC44107_TC01 | 698 | 0 | 3 |
|  | SUN family | Solyc01g088250.2.1 | Wang *et al.* 2015 | Transposon insertion-regulatory | SMUC14769_TC01 | 2113 | 0 | 21 |
|  |  | Solyc02g077260.2.1 | Wang *et al.* 2015 | Transposon insertion-regulatory | SMUC22965_TC01 | 660 | 0 | 4 |
|  |  | Solyc02g087760.2.1 | Wang *et al.* 2015 | Transposon insertion-regulatory | SMUC00131_TC01 | 2591 | 0 | 6 |
|  |  | Solyc03g026110.2.1 | Wang *et al.* 2015 | Transposon insertion-regulatory | SMUC27600_TC03 | 2228 | 0 | 8 |
|  |  | Solyc03g083100.2.1 | Wang *et al.* 2015 | Transposon insertion-regulatory | SMUC15094_TC03 | 2147 | 0 | 13 |
|  |  | Solyc03g121760.2.1 | Wang *et al.* 2015 | Transposon insertion-regulatory | SMUC12740_TC01 | 2066 | 0 | 2 |
|  |  | Solyc04g016480.2.1 | Wang *et al.* 2015 | Transposon insertion-regulatory | SMUC11044_TC01 | 1949 | 0 | 12 |
|  |  | Solyc04g050050.2.1 | Wang *et al.* 2015 | Transposon insertion-regulatory | SMUC35209_TC01 | 1723 | 0 | 10 |
|  |  | Solyc04g081210.2.1 | Wang *et al.* 2015 | Transposon insertion-regulatory | SMUC19579_TC01 | 3316 | 0 | 4 |
|  |  | Solyc05g007130.2.1 | Wang *et al.* 2015 | Transposon insertion-regulatory | SMUC34112_TC01 | 2436 | 0 | 19 |
|  |  | Solyc06g052010.1.1 | Wang *et al.* 2015 | Transposon insertion-regulatory | SMUC11906_TC01 | 484 | e^-135^ | 17 |
|  |  | Solyc06g053450.2.1 | Wang *et al.* 2015 | Transposon insertion-regulatory | SMUC23218_TC01 | 2091 | 0 | 3 |
|  |  | Solyc06g066430.2.1 | Wang *et al.* 2015 | Transposon insertion-regulatory | SMUC00296_TC01 | 1877 | 0 | 8 |
|  |  | Solyc08g007920.1.1 | Wang *et al.* 2015 | Transposon insertion-regulatory | SMUC36731_TC01 | 478 | e^-134^ | 7 |
|  |  | Solyc08g007930.1.1 | Wang *et al.* 2015 | Transposon insertion-regulatory | SMUC36731_TC01 | 430 | e^-119^ | 7 |
|  |  | Solyc08g014280.2.1 | Wang *et al.* 2015 | Transposon insertion-regulatory | SMUC23217_TC01 | 2617 | 0 | 8 |
|  |  | Solyc08g062940.2.1 | Wang *et al.* 2015 | Transposon insertion-regulatory | SMUC01545_TC02 | 1463 | 0 | 25 |
|  |  | Solyc08g080470.2.1 | Wang *et al.* 2015 | Transposon insertion-regulatory | SMUC09378_TC01 | 2315 | 0 | 14 |
|  |  | Solyc08g083240.2.1 | Wang *et al.* 2015 | Transposon insertion-regulatory | SMUC27968_TC01 | 1296 | 0 | 12 |
|  |  | Solyc09g007410.2.1 | Wang *et al.* 2015 | Transposon insertion-regulatory | SMUC16887_TC01 | 1158 | 0 | 0 |
|  |  |  | Wang *et al.* 2015 |  | SMUC01030_TC01 | 729 | 0 | 6 |
|  |  | Solyc09g082560.2.1 | Wang *et al.* 2015 | Transposon insertion-regulatory | SMUC17161_TC01 | 2028 | 0 | 6 |
|  |  | Solyc10g005000.2.1 | Wang *et al.* 2015 | Transposon insertion-regulatory | SMUC25158_TC01 | 509 | e^-143^ | 10 |
|  |  | Solyc10g008790.2.1 | Wang *et al.* 2015 | Transposon insertion-regulatory | SMUC26226_TC01 | 920 | 0 | 8 |
|  |  | Solyc10g079240.1.1 | Wang *et al.* 2015 | Transposon insertion-regulatory | SMUC36402_TC01 | 846 | 0 | 1 |
|  |  |  | Wang *et al.* 2015 |  | SMUC44107_TC01 | 698 | 0 | 3 |
|  |  | Solyc10g084280.1.1 | Wang *et al.* 2015 | Transposon insertion-regulatory | SMUC01030_TC01 | 2383 | 0 | 6 |
|  |  | Solyc10g086060.1.1 | Wang *et al.* 2015 | Transposon insertion-regulatory | SMUC22487_TC01 | 2107 | 0 | 11 |
|  |  | Solyc11g071840.1.1 | Wang *et al.* 2015 | Transposon insertion-regulatory | SMUC34119_TC01 | 2242 | 0 | 11 |
|  |  | Solyc12g008520.1.1 | Wang *et al.* 2015 | Transposon insertion-regulatory | SMUC35488_TC01 | 1574 | 0 | 5 |
|  |  | Solyc12g014130.1.1 | Wang *et al.* 2015 | Transposon insertion-regulatory | SMUC01565_TC01 | 1203 | 0 | 4 |
|  |  | SlSUN2-Solyc01g009340 | Wang *et al.* 2015 | Transposon insertion-regulatory | SMUC14283_TC01 | 1400 | 0 | 15 |
|  |  | SlSUN4-Solyc01g097490 | Wang *et al.* 2015 | Transposon insertion-regulatory | No hits | - | - | - |
|  |  | SlSUN6-SL1.00sc00090_96 | Wang *et al.* 2015 | Transposon insertion-regulatory | SMUC37046_TC01 | 577 | e^-164^ | 1 |
|  |  | SlSUN9 | Wang *et al.* 2015 | Transposon insertion-regulatory | No hits | - | - | - |
|  | Wee | Solyc09g074830.2.1 | Wang *et al.* 2015 | Gene - Kinase | SMUC36376_TC01 | 2508 | 0 | 5 |
| Anthocyanins route | F3’5’H | gi\|395260\|emb\|X70824.1 | Zhang *et* *al.* 2014 | Gene - Hydroxylase | SMUC10708_TC01 | 856 | 0 | 17 |
|  | Acyltransferase-like | gi\|307746705\|dbj\|AB522638.1 | Zhang *et* *al.* 2014 | Gene - Acyltransferase | SMUC32453_TC01 | 1913 | 0 | 13 |
|  | 5GT | gi\|112806965\|dbj\|AB269922.1 | Zhang *et* *al.* 2014 | Gene - Glucosyltransferase | SMUC27109_TC01 | 1340 | 0 | 7 |
|  | ANS | gi\|112806963\|dbj\|AB269921.1 | Zhang *et* *al.* 2014 | Gene – Anthocyanidin synthase | SMUC02266_TC01 | 894 | 0 | 34 |
|  | DFR | gi\|112806961\|dbj\|AB269920.1 | Zhang *et* *al.* 2014 | Gene - Dihydroflavonol 4-reductase | SMUC01213_TC03 | 999 | 0 | 9 |
|  | F3H | gi\|112806959\|dbj\|AB269919.1 | Zhang *et* *al.* 2014 | Gene - Flavanone 3-hydroxylase | SMUC31526_TC01 | 932 | 0 | 12 |
|  | CHI | gi\|112806957\|dbj\|AB269918.1 | Zhang *et* *al.* 2014 | Gene - Chalcone isomerase | SMUC14738_TC01 | 498 | e^-140^ | 17 |
|  | CHS2 | gi\|112806945\|dbj\|AB269912.1 | Zhang *et* *al.* 2014 | Gene - Chalcone synthase | SMUC31744_TC01 | 575 | e^-163^ | 9 |
|  | CHS3 | gi\|112806943\|dbj\|AB269911.1 | Zhang *et* *al.* 2014 | Gene - Chalcone synthase | SMUC25143_TC01 | 714 | 0 | 9 |
|  | CHS1 | gi\|112806941\|dbj\|AB269910.1 | Zhang *et* *al.* 2014 | Gene - Chalcone synthase | SMUC16785_TC01 | 504 | e^-142^ | 0 |
|  | Acyltransferase-like | gi\|307746707\|dbj\|AB522639.1 | Zhang *et* *al.* 2014 | Gene - Acyltransferase | SMUC31737_TC01 | 1118 | 0 | 23 |
| Chlorogenic acid route | 4CL | SGN-U580976 Tomato 200607 | Gramazio *et al.* 2014 | Gene - 4-Coumarate-CoA ligase | SMUC26903_TC01 | 2466 | 0 | 13 |
|  | C3H | Solyc01g096670.2.1 | Gramazio *et al.* 2014 | Cytochrome P450 | SMUC23464_TC01 | 2524 | 0 | 1 |
|  | HTC | Solyc03g117600.2.1 | Gramazio *et al.* 2014 | Gene - Transferase | SMUC33401_TC01 | 2064 | 0 | 11 |
|  | HQT | Solyc07g005760.2.1 | Gramazio *et al.* 2014 | Gene - Transferase | SMUC29657_TC01 | 1709 | 0 | 24 |
| Saponines route | Egp#1-1 | gi\|58430475\|dbj\|AB182375.1 | Kohara *et al.* 2005 | Gene - Glycosyltransferase | SMUC18755_TC01 | 636 | 0 | 16 |
|  | Egp#1-4 | gi\|58430477\|dbj\|AB182376.1 | Kohara *et al.* 2005 | Gene - Glycosyltransferase | SMUC18755_TC01 | 508 | e^-142^ | 5 |
|  | Ptt#1-53 | gi\|58430479\|dbj\|AB182377.1 | Kohara *et al.* 2005 | Gene - Glycosyltransferase | SMUC29980_TC03 | 858 | 0 | 20 |
|  | Ptt21 | gi\|58430481\|dbj\|AB182378.1 | Kohara *et al.* 2005 | Gene - Glycosyltransferase | SMUC26561_TC01 | 1065 | 0 | 16 |
|  | Sgt1-1 | gi\|58430483\|dbj\|AB182379.1 | Kohara *et al.* 2005 | Gene - Glycosyltransferase | SMUC29980_TC01 | 555 | e^-157^ | 20 |
|  | Ptt#5-30 | gi\|58430485\|dbj\|AB182380.1 | Kohara *et al.* 2005 | Gene - Glycosyltransferase | SMUC29980_TC03 | 567 | e^-161^ | 20 |
|  | Sa#6-15 | gi\|58430487\|dbj\|AB182381.1 | Kohara *et al.* 2005 | Gene - Glycosyltransferase | SMUC18755_TC01 | 583 | e^-165^ | 5 |
|  | Sk#7-4 | gi\|58430489\|dbj\|AB182382.1\| | Kohara *et al.* 2005 | Gene - Glycosyltransferase | SMUC27168_TC01 | 640 | 0 | 0 |
|  | Sk#7-4 | gi\|58430491\|dbj\|AB182383.1 | Kohara *et al.* 2005 | Gene - Glycosyltransferase | SMUC26561_TC01 | 729 | 0 | 16 |
|  | Sk#7-5 | gi\|58430493\|dbj\|AB182384.1 | Kohara *et al.* 2005 | Gene - Glycosyltransferase | SMUC18755_TC01 | 559 | e^-158^ | 5 |
|  | SaGT4A | gi\|58430495\|dbj\|AB182385.1 | Kohara *et al.* 2005 | Gene - Glycosyltransferase | SMUC18755_TC01 | 1065 | 0 | 5 |
|  | SaGT4R | gi\|58430497\|dbj\|AB182386.1 | Kohara *et al.* 2005 | Gene - Glycosyltransferase | SMUC18755_TC01 | 837 | 0 | 5 |
|  | SaGT6 | gi\|58430499\|dbj\|AB182387.1 | Kohara *et al.* 2005 | Gene - Glycosyltransferase | SMUC27169_TC01 | 1618 | 0 | 14 |
| Sucrose accumulator | TIV1 | gi\|170361\|gb\|M81081.1 | Klann *et al.* 1992 | Gene – Acid invertase | SMUC10010_TC01 | 2769 | 0 | 25 |
