# Supplementary material for: The first de novo transcriptome of pepino (Solanum muricatum): assembly, comprehensive analysis and comparison with the closely related species S. caripense, potato and tomato
Source: BMC Genomics. 2016 May 4;17:321. doi: 10.1186/s12864-016-2656-8 (PMC4855764; doi:10.1186/s12864-016-2656-8)
Supplement: Additional file 9: — ClustalW alignment. Text file with multiple alignment from the concatenation of the five genes studied in pepino, tomato, potato, eggplant, pepper and tobacco. (DOCX 34 kb) [file 12864_2016_2656_MOESM9_ESM.docx]

CLUSTAL 2.1 multiple sequence alignment

tomato ATGGCAAGCATCACAGCTTCACAC---TTTGTGTCAAGAAGCTCAAATGTCCAAACT--- 54

potato ATGGCAAGCATCACAGCTTCACACCACTTTGTGTCAAGAAG---------CCAAACT--- 48

pepino ATGGCAAGCATCACAGCTTCACAC---TTTGTGTCAAGAAGCTCAAATGTCCAAACT--- 54

eggplant ATGGCAAGCATCACGGCTTCACAC---TTTGTGTCAACAAGATCAAATGTCCAAACT--- 54

Pepper ATGGCAAGCATCACAGCTTCACAC---TTTGTGTCGAGAAGCTCAAACGTCCGAGCTGGA 57

tobaco ATGGCAAGCATCACAGCTTCACAC---TTTGTGTCAAGAAGCTCAAATGTGTGCAGTGGA 57

**************.********* ********.* *** ... *

tomato ------TCACTAGACACCAAATCAACCTTGTCACAGATAGGACTCAGGAACCATACTCTG 108

potato ------TCACTAGACACCAAATCAACCTTGTCACAGATAGGACTCAGGAACCATACTCTG 102

pepino ------TCACTGGACACCAGACCAACCTTGTCACAGATAAGACTCAGGAACCATACTCTG 108

eggplant ------TCAGTAGACACCAAATCAGACTTGTCACAGATACGACTCAGGAATCATACTCTG 108

Pepper TCAACTTCAGTAGACACCAAAGCCAACTTGTCTCAGATAGGGCTGAGGAACCATACTGTG 117

tobaco GCAGCCTCTGTAGACACCAGAGCAAACTTGTCACAGATAGGGCTGAGGAACCATGCTCTG 117

**: *.*******.* *...******:****** *.** ***** ***.** **

tomato ACACACAATGGGTTAAGGGCTGTTAACAAGCTTGATGGGCTCCAATCAAGAACTAATACT 168

potato ACTCACAATGGTTTAAGGGCTGTTAACAAGCTTGATGGGCTCCAATCAAGAACTAATACT 162

pepino ACGCACAATGGGTTGAGGGCTGTTAACAAGCTTGATAGGCTCCAATCAAGAACTAATACT 168

eggplant ACTTACAATGGGTTGAGGGCTGTTAACAAGTTTCCTATGCTCCAAT------------CT 156

Pepper ACTCACAATGGGTTGAGGGCAGTTAACAAGGTTGATATGCTCCAATCAAGAACTAATGTT 177

tobaco ACTCACAATGGGTTGAGGGCTGTTAACAAGGTTGATATGCTGCAATCAAGAACTAATACT 177

** ******* **.*****:********* ** .*. *** **** *

tomato AAGGTAACACCCAAGATG------------AGATCCA---------CCAAG--------- 198

potato AAGGTAACACCCAAGATG------------GCATCCAGAACTGAGACCAAGAGACCTGGA 210

pepino AAGGTGACAGCCAAGATG------------GGATCCAGAACTGAGACCAGCAGGCCTGG- 215

eggplant AAGAAATCTGGCAAACAG------------GGATCCACAACTGAGACGAAGAGACCTGG- 203

Pepper AAGGTTACAGCCAAGAAGTCTGGCAAACAGGGATCCAGAACTGACACGAAGAGGCC---- 233

tobaco AAGGTGACAGCCAAGAAATCCAGTAAACAGGGATCCAGAACTGATACGGAGAGGCC---- 233

***.: :*: ***..:. . ***** * ..

tomato ---TCAGCTACCATTGTTTGTGGAAAGGGAATGAACTTGATCTTTGTGGGTACTGAGGTT 255

potato TGCTCAGCTACCATTGTTTGTGGAAAGGGAATGAACTTGATCTTTGTGGGTACTGAGGTT 270

pepino --ATCAGGTACCATTGTTTGTGGAAAGGGAATGAACTTGATCTTTGTGGGTACTGAGGTT 273

eggplant --ATCAGGTACCATTGTTTGTGCACAGGGAATGAACTTGATCTTTGTAGGAACTGAGGTG 261

Pepper --ATCAGGTACCATTGTTTGTGGAAAGGGGATGAACTTGATCTTTGTGGGAACTGAGGTG 291

tobaco --ATCAGGTACAATTGTTTGTGGAAAGGGGATGAACGTGATCCTTGTTGGAGCTGAGGTC 291

**** ***.********** *.****.****** ***** **** **:.*******

tomato GGTCCTTGGAGCAAAACTGGTGGACTAGGTGATGTTCTTGGTGGACTACCACCAGCCCTT 315

potato GGTCCTTGGAGCAAAACTGGTGGACTAGGTGATGTTCTTGGTGGACTACCACCAGCCCTT 330

pepino GGTCCTTGGAGCAAAACTGGTGGGCTAGGTGATGTTCTTGGTGGACTACCACCAGCTCTC 333

eggplant GGTCCTTGGAGCAAAACTGGTGGACTAGGGGATGTTCTTGGTGGACTACCATCAGCCCTG 321

Pepper GGTCCTTGGAGCAAAACTGGTGGACTAGGTGATGTTCTTGGTGGACTACCACCAGCCCTG 351

tobaco GGTCCTTGGAGCAAAACTGGCGGGTTAGGTGATGTTCTTGGTGGACTACCACCAGCCTTG 351

******************** **. **** ********************* **** *

tomato GCAGCCCGCGGACATCGGGTAATGACAATATCCCCCCGTTATGACCAATACAAAGATGCT 375

potato GCAGCCCGCGGACATCGGGTAATGACAATATCCCCCCGTTATGACCAATACAAAGATGCT 390

pepino GCAGCACGCGGACATCGGGTAATGACAATATCCCCCCGTTATGATCAATACAAAGATGCT 393

eggplant GCAGCTCGTGGACATCGGGTAATGACAATATCCCCCCGTTATGACCAATACAAAGATGCT 381

Pepper GCAGCCCGTGGACATCGGGTAATGACAATATCTCCCCGTTATGACCAATACAAAGATGCC 411

tobaco GCAGCCCGTGGACATCGCGTAATGACAGTATCTCCACGTTATGACCAATACAAAGATGCT 411

***** ** ******** *********.**** **.******** **************

tomato TGGGATACTAGCGTTGCCCTTGAGGTCAAAGTTGGAGACAGCATTGAAATTGTTCGCTTC 435

potato TGGGATACTAGCGTTGCGGTTGAGGTCAAAGTTGGAGACAGCATTGAAATTGTTCGTTTC 450

pepino TGGGATACTAGCGTTGCGGTTGAGGTCAAAGTTGGAGACAGCATTGAAATGGTTCGTTTC 453

eggplant TGGGATACTAGTGTTGCCATTGAGGTCAAAGTTGGAGACAGCATTGAAATTGTTCGTTTC 441

Pepper TGGGATACAAGCATTACGGTTGAGGTCAAAGTTGGAGACAACATCGAAGTTGTTCGTTTC 471

tobaco TGGGATACTAGCGTTGTCGTTGAGATCAAAGTTGGAGACAAAATTGAAATTGTTCGTTTC 471

********:** .**. *****.***************..** ***.* ***** ***

tomato TTTCACTGCTATAAACGTGGGGTTGATCGTGTTTTTGTTGACCACCCAATGTTCTTGGAG 495

potato TTTCACTGCTATAAACGTGGGGTTGATCGTGTTTTTGTTGACCACCCAATGTTCTTGGAG 510

pepino TTTCACTGTTATAAACGTGGGGTTGATCGTGTTTTTGTTGACCACCCAATCTTCTTGGAG 513

eggplant TTTCACTGCTATAAACGTGGGGTTGATCGTGTTTTTGTTGACCACCCAATGTTCTTGGAG 501

Pepper TTTCACTGCTATAAACGTGGGGTTGATCGCATTTTTGTTGACCACCCAATGTTCTTGGAG 531

tobaco TTTCACTGCTATAAACGTGGGGTTGATCGTGTTTTTGTTGACCACCCAATGTTCTTGGAG 531

******** ******************** .******************* *********

tomato AAAGTTTGGGGCAAAACTGGTTCAAAAATCTACGGCCCCAAAGCTGGACTGGATTATCTG 555

potato AAAGTTTGGGGCAAAACTGGTTCAAAAATCTATGGCCCCAAAGCTGGACTAGATTATCTG 570

pepino AAAGTTTGGGGCAAAACTGGTTCAAAAATCTATGGCCCCAAAGCTGGACAAGATTATTTG 573

eggplant AAAGTTTGGGGCAAAACTGGTTCAAAAATCTATGGCCCCAAAGCTGGACAAGATTATTTG 561

Pepper AGAGTTTGGGGCAAAACTGCTTCAAAAATCTATGGCCCCAAAGCTGGACAAGATTATTTG 591

tobaco AAAGTTTGGGGCAAAACTGCTTCAAAAATCTATGGCCCCAAAGCTGGAGAAGAATATTTG 591

*.***************** ************ *************** :.**:*** **

tomato GACAATGAACTTAGGTTCAGCTTGTTGTGTCAAGCAGCCCTAGAGGCACCTAGAGTTTTG 615

potato GACAATGAACTTAGGTTCAGCTTGTTGTGTCAAGCAGCCCTAGAGGCACCTAAAGTTTTG 630

pepino GACAATGAACTTAGGTTCAGCTTGATGTGTCAAGCAGCCCTAGAGGCACCTAGAGTTCTG 633

eggplant GACAATGAACTTAGATTCAGCCTGTTGTGTCAAGCAGCCCTAGAGGCACCCAGAGTTCTG 621

Pepper GACAATGAACTTAGGTTCAGCTTGTTGTGTCAAGCAGCACTAGAGGCACCTAGAGTTCTG 651

tobaco GACAATGAACTTAGGTTTAGCTTGTTGTGTCAAGCAGCTCTAGAAGCACCTAGAGTTCTG 651

**************.** *** **:************* *****.***** *.**** **

tomato AATTTGAACAGTAGCAACTACTTCTCAGGACCATATGGAGAGGATGTTCTCTTCATTGCC 675

potato AATTTGAACAGTAGCAACTACTTCTCAGGACCATATGGAGAGGATGTTCTCTTCATTGCC 690

pepino AATTTGAACTGTAGCAAATACTTCTCAGGACCATATGGAGAGGATGTTCTCTTCATTGCC 693

eggplant AATTTGAACTGCAGCAAATACTTCTCAGGACCATATGGAGAAGATGTTCTCTTCATTGCC 681

Pepper AATTTGAACTGCAGCAAAAGCTTCTCAGGACCATATGGAGAGGATGTTCTCTTCATTGCC 711

tobaco AATTTGAACTGCAGTGAATACTTCTCAGGACCATATGGAGAGGATGTTGTCTTCATTGCC 711

*********:* ** .*.:.*********************.****** ***********

tomato AATGATTGGCACACAGCTCTAATTCCCTGCTACTTAAAGTCAATGTACCAGTCCAGAGGA 735

potato AATGATTGGCACACAGCTCTCATTCCTTGCTACTTGAAGTCAATGTACCAGTCCAGAGGA 750

pepino AATGATTGGCACACAGCTCTCATTCCCTGCTACTTGAAGTCAATGTACCAGTCAAAAGGA 753

eggplant AATGATTGGCACACAGCTCTCATTCCCTGCTACTTGAAGTCAATGTACCAGTCAAGAGGA 741

Pepper AATGACTGGCACACAGCTCTCATTCCTTGCTACCTGAAGTCAATGTACCAATCTAAAGGA 771

tobaco AACGATTGGCACACTGCTCTCCTTCCCTGCTACCTGAAGTCGATGTACCAATCAAGAGGA 771

** ** ********:*****..**** ****** *.*****.********.** *.****

tomato ATCTATTTGAATGCCAAGGTCGCTTTCTGCATCCATAACATTGCCTACCAAGGCAGATTT 795

potato ATCTATTTGAATGCCAAGGTCGCTTTCTGCATCCATAACATTGCCTACCAAGGCCGATTT 810

pepino ATCTATTTGAATGCCAAGGTTGCTTTCTGCATCCACAACATTGCCTACCAAGGACGATTT 813

eggplant ATCTATTTGAATGCCAAGGTCGCTTTCTGCATCCATAACATTGCCTACCAAGGCCGATTT 801

Pepper ATCTATATGAATGCGAAGGTTGCTTTCTGCATCCATAACATTGCCTACCAAGGCCGATTT 831

tobaco ATCTATATGAATGCCAAGGTCGCTTTCTGCATCCATAACATTGCCTACCAAGGGCGATTT 831

******:******* ***** ************** ***************** .*****

tomato TCTTTCTCTGACTTCCCTCTTCTCAATCTTCCTGATGAATTCAGGGGTTCTTTTGATTTC 855

potato TCTTTCTCTGACTTCCCTCTTCTCAATCTTCCTGATGAATTCAGGGGTTCTTTTGATTTC 870

pepino TCTTTCTCCGACTTCCCTCTTCTCAATCTTCCTGATGAATTCAGGGGTTCTTTTGATTTC 873

eggplant ACTTTCTCTGACTTCCCTCTTCTCAATCTTCCTGATGAATTCAGGGGTTCTTTTGATTTC 861

Pepper TCTTTCTCAGACTTCTCTCTTCTCAATCTGCCTGATGAATTCAGGAGTTCTTTTGATTTC 891

tobaco GCTTTTTCAGACTTCTCTCTTCTCAATATGCCTGATGAATACAAGAGTTCTTTTGATTTC 891

**** ** ****** ***********.* **********:**.*.**************

tomato ATTGATGGATATGAGAAACCTGTTAAGGGTAGGAAAATCAACTGGATGAAGGCTGGGATA 915

potato ATTGATGGATATGAGAAGCCTGTTAAGGGTAGGAAAATCAACTGGATGAAGGCTGGGATA 930

pepino ATTGATGGATATGAAAAGCCTGTTAAGGGTAGGAAAATCAACTGGATGAAGGCTGGGATG 933

eggplant ATTGATGGACATGAAAAGCCTGTGAAAGGTAGGAAAATCAACTGGATGAAGGCTGGGATA 921

Pepper ATTGATGGATACGAAAAGCCTGTTAAGGGTAGGAAAATCAACTGGATGAAGGCTGGGATA 951

tobaco ATTGATGGATATGAAAAGCCTGTTAAGGGTAGGAAAATCAACTGGATGAAGGCTGGGATA 951

********* * **.**.***** **.********************************.

tomato TTAGAATCACATAGGGTGGTTACAGTAAGCCCATACTATGCCCAAGAACTTGTCTCTGCT 975

potato TTAGAATCACATAGGGTGGTTACAGTGAGCCCATACTATGCCCAAGAACTTGTCTCTGCT 990

pepino TTGGAATCACATAGGGTGGTTACAGTGAGCCCATACTATGCCCAAGAACTTGTCTCCGCT 993

eggplant TTAGAATCAGATAGGGTGGTTACAGTGAGCCCATACTATGCTCAAGAACTTGTCTCAGCT 981

Pepper TTAGAATCACATAGGGTGGTTACAGTGAGCCCATACTATGCCCAAGAACTTGTTTCCGGT 1011

tobaco TTGGAATCACATAGGGTGGTTACTGTGAGCCCATACTATGCTCAAGAACTTGTTTCCGGT 1011

**.****** *************:**.************** *********** ** * *

tomato GTTGACAAGGGTGTTGAATTGGACAGTGTCCTTCGTAAGACTTGCATAACTGGGATTGTG 1035

potato GTTGACAAGGGTGTTGAATTGGACAGTGTCCTTCGTAAGACTTGCATAACTGGGATTGTG 1050

pepino GTTGACAAGGGTGTTGAATTGGATAATGTCCTTCGTAAGACTGGCATAACTGGGATTGTG 1053

eggplant GTTGACAAGGGTGTTGAATTGGATAATGTCCTTCGTAAGACTACCATAACTGGTATTGTG 1041

Pepper GTTGACAAGGGTGTTGAATTGGATAACGTCCTTCGTAAGACTAGCATAACTGGGATTGTG 1071

tobaco GTTGACAAGGGTGTCGAACTGGATAACGTCCTTCGTAAGACTAGCATAACTGGGATTGTG 1071

************** *** **** *. *************** ********* ******

tomato AATGGCATGGATACACAAGAGTGGAACCCAGCGACTGACAAATACACAGATGTCAAATAC 1095

potato AATGGCATGGATACACAAGAGTGGAACCCAGCGACTGACAAATACACAGATGTCAAATAC 1110

pepino AATGGCATGGATACACAAGAGTGGAACCCAGCAACTGACAAATACACAGATGTCAAATAC 1113

eggplant AATGGCATGGATACACAAGAGTGGAACCCAGCAACTGACAAATACACAGATGTCAAATAT 1101

Pepper AATGGTATGGATACACAAGAGTGGAACCCAGCAACTGACAAATACACCGATGTCAAATAT 1131

tobaco AATGGCATGGATATTCAAGAGTGGAACCCAGCAACTGACAAATACACTGATGTCAAATAT 1131

***** ******* :*****************.************** ***********

tomato GATATAACCACTGTCATGGACGCAAAACCTTTACTAAAGGAGGCTCTTCAAGCAGCAGTT 1155

potato GATATAACCACTGTCATGGACGCAAAACCTTTACTAAAGGAGGCTCTTCAAGCAGCAGTT 1170

pepino GATATAACCACGGTCATGGATGCAAAACCTTTACTAAAGGAGGCTCTTCAAGCAGCAGTT 1173

eggplant GATATAACCACTGTCATGGATGCAAAGCCTTTACTAAAGGAGGCTCTTCAAGCAGCAGTT 1161

Pepper GATATAACCACTGTCATGGACGCAAAGCCTTTACTGAAGGAGGCTCTTCAAGCAGCAGTT 1191

tobaco GATATAACCACTGTCATGGACGCAAAACCTTTACTGAAGGAGGCTCTTCAAGCAGCAGTT 1191

*********** ******** *****.********.************************

tomato GGCTTGCCTGTTGACAAGAAGATCCCCTTGATTGGCTTCATCGGCAGACTTGAGGAGCAG 1215

potato GGCTTGCCTGTTGACAAGAAGATCCCTTTGATTGGCTTCATCGGCAGACTTGAGGAGCAG 1230

pepino GGCTTGCCTGTTGACAAGAAGATCCCTTTGATTGGCTTCATCGGCAGACTTGAGGAGCAG 1233

eggplant GGCTTGCCTGTTGACAGGAAGATCCCTTTGATTGGCTTCATCGGCAGACTTGAGGAGCAG 1221

Pepper GGCTTGCCTGTTGACAGGAACATCCCTTTGATTGGCTTCATCGGCAGACTTGAAGAGCAG 1251

tobaco GGCTTGCCTGTTGACAGGAAGATCCCTTTGATTGGATTCATTGGCAGACTTGAAGAGCAG 1251

****************.*** ***** ********.***** ***********.******

tomato AAAGGTTCAGATATTCTTGTTGCTGCAATTCACAAGTTTATCGAATTGGATGTTCAAATT 1275

potato AAAGGTTCAGATATTCTTGTTGCTGCAATTCACAAGTTCATCGGATTGGATGTTCAAATT 1290

pepino AAAGGTTCAGATATTCTTGTTGCTGCAATTCGCAAGTTCATTGGATTGGATGTTCAAATT 1293

eggplant AAAGGTTCAGATATTCTTGTTGCTGCAATTCACAAGTTCATCGGATTGGATGTTCAAATA 1281

Pepper AAAGGTTCAGACATTCTTGTTGCTGCAATTCACGAGTTCATCGGTTTGGATGTTCAAATA 1311

tobaco AAAGGTTCTGACATTCTTGTTGCTGCAATTCACAAGTTCATTGGATTGGATGTTCAAATA 1311

********:** *******************.*.**** ** *.:**************:

tomato GTAGTCCTTGGAACTGGCAAAAAGGAGTTTGAGCAGGAGATTGAACAGCTCGAAGTGTTG 1335

potato GTAGTCCTTGGAACTGGCAAAAAGGAGTTTGAGCAGGAGATTGAACAGCTCGAAGTGTTG 1350

pepino GTAGTCCTTGGAACTGGCAAAAAGGAGTTTGAGCAGGAGATTGAACAGCTCGAAGTGTTG 1353

eggplant GTAGTCCTTGGAACTGGAAAAAAGAAGTTCGAACAGGAGATTGAACAGCTCGAGGTGTTG 1341

Pepper GTAGTCCTTGGAACTGGCAAAAAGAAGTTCGAGCAGGAGATTGAACAGCTCGAAGTGTTG 1371

tobaco GTAGTCCTTGGAACTGGAAAAAAGGAGTTCGAACAGGAGATTGAACAGCTGGAAGTGTTG 1371

*****************.******.**** **.***************** **.******

tomato TACCCTAATAAAGCTAAAGGAGTGGCAAAATTCAATGTCCCTTTGGCTCACATGATCACT 1395

potato TACCCTAACAAAGCTAAAGGAGTGGCAAAATTCAATGTCCCTTTGGCTCACATGATCACT 1410

pepino TACCCTAACAAAGCTAAAGGAGTAGCAAAATTCAATGTCCCTTTGGCTCACATGATCACT 1413

eggplant TACCCTAACAAAGCTAAAGGAGTGGCAAAATTCAATGTCCCTTTGGCTCACATGATCACT 1401

Pepper TATCCTAACAAAGCTAAAGGCGTCGCAAAATTCAATGTCCCTCTGGCTCACATGATCACC 1431

tobaco TATCCTAACAAAGCTAAAGGAGTGGCAAAATTCAATGTCCCTTTGGCTCACATGATCACC 1431

** ***** ***********.** ****************** ****************

tomato GCTGGTGCTGATTTTATGTTGGTCCCAAGCAGATTTGAACCTTGTGGTCTCATTCAGTTA 1455

potato GCTGGTGCTGATTTTATGTTGGTTCCAAGCAGATTTGAACCTTGTGGTCTCATTCAGTTA 1470

pepino GCTGGTGCTGATTTTATGTTGGTTCCAAGCAGATTTGAACCTTGTGGTCTCATTCAGTTA 1473

eggplant GCTGGTGCTGATTTTATGTTGGTTCCAAGCAGATTTGAACCTTGTGGACTCATTCAGTTA 1461

Pepper GCTGGTGCTGATTTTATGTTGGTTCCAAGCAGATTTGAACCTTGTGGTCTCATTCAGTTG 1491

tobaco GCTGGTGCTGATTTTATGTTGGTTCCTAGCAGATTTGAACCTTGTGGTCTCATTCAGTTA 1491

*********************** **:********************:***********.

tomato CATGCTATGCGATATGGAACAGTGCCAATCTGTGCCTCAACTGGTGGACTTGTTGACACT 1515

potato CATGCTATGCGATATGGAACAGTGCCAATCTGTGCATCGACTGGTGGACTTGTTGACACT 1530

pepino CATGCTATGCGATATGGAACAGTGCCAATCTGTGCCTCGACTGGTGGACTTGTTGACACT 1533

eggplant CATGCTATGCGATATGGAACAGTGCCAATCTGTGCCTCGACTGGTGGACTTGTGGACACT 1521

Pepper CATGCTATGCGATATGGAACAGTGCCTATCTGTGCCTCAACTGGTGGACTTGTTGACACT 1551

tobaco CATGCTATGCGCTATGGAACAGTGCCAATCTGTGCCTCAACTGGTGGACTTGTTGACACT 1551

***********.**************:********.**.************** ******

tomato GTGAAAGAAGGCTATACTGGATTCCATATGGGAGCCTTCAATGTTGAATGCGATGTTGTT 1575

potato GTGAAAGAAGGCTATACTGGATTCCATATGGGAGCCTTCAATGTTGAATGCGATGTTGTT 1590

pepino GTGAAAGAAGGCTATACTGGATTCCATATGGGAGCCTTCAATGTTGAATGCGATGTTGTT 1593

eggplant GTGAAAGAAGGCTATACTGGATTCCATATGGGAGCCTTCAATGTTGAATGCGATGTTGTT 1581

Pepper GTGAAAGAAGGCTATACTGGGTTCCATATGGGAGCCTTCAATGTTGAATGTGATGTTGTT 1611

tobaco GTGAAAGAAGGCTACACTGGATTCCATATGGGAGCCTTCAACGTTGAATGCGATGTCGTT 1611

************** *****.******************** ******** ***** ***

tomato GACCCAGATGATGTGCTTAAGATAGTAACAACAGTGTCAAGAGCTCTTGCAGTCTATGGC 1635

potato GACCCAGCTGATGTGCTTAAGATAGTAACAACAGTTGCTAGAGCTCTTGCAGTCTATGGC 1650

pepino GACCCTGCTGATGTGCTTAAGATAGTAACAACAGTTGCTAGAGCTCTTGCAGTCTATGGC 1653

eggplant GACCCAGCTGATGTGCTTAAGATAGAAACAACAGTTGCTAGAGCTCTTGCAGTCTATGGC 1641

Pepper GATCCAGCTGATGTGCTTAAGATAGTAACAACAGTTACTAGAGCTCTTGCAGCCTATGGC 1671

tobaco GACCCAGCTGATGTGCTTAAGATAGTAACAACAGTTACTAGAGCTCTTGAAGTTTATGGC 1671

** **:*.*****************:********* *:**********.** ******

tomato ACCCTTGCGTTTGCTGAGATGATAAAAAATTGCATGTCAGAGGAGCTCTCTTGGAAGGAA 1695

potato ACCCTCGCATTTGCTGAGATGATAAAAAATTGCATGTCAGAGGAACTCTCCTGGAAGGAA 1710

pepino ACCCTCTCGTTTGCTGAGATGATAAAAAATTGCATGTCAGAGGAGCTCTCCTGGAAGGAA 1713

eggplant ACCCTCGCATTTGCTGAGATGATAAAAAATTGCATGTCAGAGGAGCTCTCCTGGAAGGAA 1701

Pepper ACCCTTGCGTTTGCTGAGATGATCAAAAACTGCATGTCAGAGGAACTCTCCTGGAAGGAA 1731

tobaco ACCCTCGCTTTTGCTGAGATGATCAAAAACTGCATGTCACAGGAGCTCTCCTGGAAGGAA 1731

***** * **************.***** ********* ****.***** *********

tomato CCTGCCAAGAAATGGGAGACGTTGCTATTGGGCTTAGGAGCTTCTGGCAGTGAACCCGGT 1755

potato CCTGCCAAGAAATGGGAGACATTGCTATTGGGCTTAGGAGCTTCTGGCAGTGAACCCGGT 1770

pepino CCTGCCAAGAAATGGGAGACATTGCTATTGGGCTTAGGAGCTGCTGGCAGTGAACCCGGT 1773

eggplant CCTGCCAAGAAATGGGAGACATTGTTATTGGGCTTAGGAGCTGCTGGCAGTGAACCCGGT 1761

Pepper CCCGCCAAGAAATGGGAGACATTGCTATTGGGCTTAGGAGCTACTGGAGGTGAACCTGGT 1791

tobaco CCTGCCAAGAAATGGGAGACGTTGCTATTGAGCTTAGGAGCTGCTGGCAGTGAAGCCGGT 1791

** *****************.*** *****.*********** ****..***** * ***

tomato GTCGAAGGGGAAGAAATCGCTCCACTTGCCAAGGAAAATGTAGCCACTCCCT-AATGAAG 1814

potato GTTGAAGGGGAAGAAATCGCTCCACTTGCCAAGGAAAATGTAGCCACTCCCTAAATGAAG 1830

pepino GATGAAGGGGAAGAAATCGCTCCACTTGCCAAGGAAAATGTAGCCACTCCCTAAATGAAG 1833

eggplant GTTGACGGGGAAGAAATCGCACCACTTGCCAAGGAAAATGTAGCCACTCCCTAAATGAAG 1821

Pepper GTTGAAGGGGAAGAAATCGCCCCGCTTGCCAAGGAAAATGTAGCCACTCCCTAAATGAAG 1851

tobaco GTTGAAGGGGATGAAATCGCCCCACTTGCCAAGGAAAATGTGGCCACTCCTTAAATGAAG 1851

*: **.*****:******** **.*****************.******** * *******

tomato GTTGTTGAAGTTCTTCACATGAATGGAGGAAATGGAGACATTAGCTATGCAAATAATTCC 1874

potato CTTGTTGAAGTTCTTCACATGAATGGAGGAATTGGAGACATTAGCTATGCAAACAATTCT 1890

pepino GTTGTTGAAGTTCTTCACATGAATGGAGGAAATGGAGACATTAGCTATGCAAACAATTCC 1893

eggplant GTTGTTGAAGTTCTTCACATGAATGGAGGAAATGGAGACATTAGCTATGCAAATAATTCT 1881

Pepper GTTGTTGAAGTTCTTCACATGAATGGAGGAAATGGAGACGTTAGCTATGCAAACAATTCT 1911

tobaco GTTGTTGAAGTTCTTCACATGAATGGAGGAAATGGAGACATTAGCTATGCAAAAAATTCC 1911

******************************:*******.************* *****

tomato TTGGTTCAGAAAAAGGTGATTCTCATGACAAAGCCAATAAGAGATCAAGCCATAAGTGAT 1934

potato TTGGTTCAGAGAAAGGCCATTCTCATGACAAAGCCAATAATAGAACAAGCCATGCGTGAT 1950

pepino TTGGTTCAGAAAAAGGTGATTCTCATGACAAAGCCAATAACAGATCAAGCCATAAGTGAT 1953

eggplant TTGGTTCAGAGAAAGGTGATTTTCATGACAAAGCCAATAACAGATGAAGCCATAAGTGAT 1941

Pepper TTGGTTCAGAGAAAGGTGATTCTCATGACAAAGCCAATAACTGAGGAAGCCATAAGTGAT 1971

tobaco TTGGTTCAGCAAAAGGTAATTCTCATGACAAAGCCAATAACAGAGCAAGCCATAACTGAA 1971

*********..***** *** ****************** :** *******.. ***:

tomato CTCTATTGTAACCTATTCCCAGAAACATTATACATTGCTGATTTGGGTTGTTCTTCTGGA 1994

potato CTTTACTGCAGCCTTCTCCCAAAAAACTTATGCATTGCTGATTTGGGTTGTTCCTCTGGA 2010

pepino CTCTATTGCAACCTCTTCCCAGAAACCTTATGCATTGCTGATTTGGGTTGTTCTTCTGGA 2013

eggplant CTCTACTGCAACCTCATCCCAGAAACCATATGCATTGCTGATTTAGGTTGTTCTTCTGGA 2001

Pepper CTTTACTGCAGCCTCTTACCAGAAACGTTATGCATTGCTGATTTGGGCTGTTCTTCTGGA 2031

tobaco CTCTACTGCAGTCTCTTCCCACAAAACTTATGCATTGCGGATTTGGGTTGTTCCTATGGA 2031

** ** ** *. ** *.*** ***. :***.****** *****.** ***** *.****

tomato GCGAACACTTTTTTAGTGGTATCAGAACTTGTTAAGGTCATTGAAAAAGAACGAAAAAAA 2054

potato GCGAACACTTTCTTGGTGGTATCAGAGCTTGCTAAAACCATTGAGAAAGAACGAAAAAAG 2070

pepino GCGAACACTTTCTTAGTGGTATCAGAACTTGTGAAAATCATTGAAAAAGAACGAAAGAAA 2073

eggplant GCGAACACTTTTCTAGTGGTGTCAAATTTTGTCAAAATCATCGAGAAGGAACGAAAAAAT 2061

Pepper GCTAACACTTTCTTGGTGGTATCAGAGCTTGTTAAAGTCGTTGAAAAAGAACGAAAAAAA 2091

tobaco GCTAATACTTTCATAGTAGTATCAGAGCTCGTTAAAATCGTTGAAAAAGAACGGAAGAAG 2091

** ** ***** *.**.**.***.* * * **.. *.* **.**.*****.**.**

tomato CACGATCTACAATCACCAGAGTTTTATTTTCACTTCAATGATCTCCCTGGAAATGATTTT 2114

potato CATGGATTTCAATCACCAGAATTTCATTTTCTCTTTAATGATCTTCCTAGCAATGATTTT 2130

pepino CACAATCTTCAATCACCAGAGTTTTATTTTCGCTTCAATGATCTCCCTGGCAATGATTTT 2133

eggplant CATGGAGTTCAATCGCCAGAGTTTCATTTTAACTTCAACGATCTCCCTGGCAATGATTTT 2121

Pepper CACAAACTTCAATCTCCAGAGTTTTATTTTCGCTTCAATGACCTTCCTGGCAATGATTTT 2151

tobaco CACGGGTTTCAGTCACCAGAGTTTCATTTTAACTTCAATGATCTTCCTGGCAATGACTTT 2151

** .. *:**.** *****.*** *****. *** ** ** ** ***.*.***** ***

tomato AACGCGATTTTTCGATCGTTAGGGGAATTTGAACAAAATTTGAAAAAACAAATTGGAGAA 2174

potato AACACTATTTTTCAATCATTGGGAGAATTTGAACATGATTTGAGAAAGCAATTTGGAGAA 2190

pepino AACGCGATTTTTCAATCGTTAGGGGAATTTGAACACAATTTGAGAAATCAAATTGGAGAA 2193

eggplant AACACGATTTTTCGATCATTGGGAGAATTTGAACAAAATTTGAGAAGCCAAATTGGTAAA 2181

Pepper AACGTGATTTTTCAGTCATTGGGAGAATTTGAACAAGATTTGAGAAACCAAACTGGAGAA 2211

tobaco AATACCATTTTCCAGTCATTGGATGTATTTCAACAAGATTTGAGAAAGCAAATAGGAGAA 2211

** . ***** *..**.**.*. *:**** **** .******.**. ***: :**:.**

tomato GAACTTGGTCCATGTTTTTTTAGTGGTGTGGCTGGTTCATTTTATTCTAGACTTTTTCCA 2234

potato GGATTTGGTCCATGTTTTTTTAGTGGAGTGTCTGGTTCATTTTATACTAGACTTTTTCCT 2250

pepino GGACTTGGTCCATGTTTTTTTAGTGGTGTGGCTGGTTCATTTTATGCTAGACTTTTTCCT 2253

eggplant GAACTTGGTTCATGTTTTTTCAGTGGTGTGCCTGGTTCATTTTATACCAGACTTTTTCCT 2241

Pepper GAACTTGGTCCATGTTTTTTTAGTGGAGTCCCTGGTTCATTTTATACTCGACTTTTTCCT 2271

tobaco AAGTTTGGTCCATGCTTTTTTAGTGGAGTGCCTGGTTCATTTTATACTAGACTTTTCCCT 2271

... ***** **** ***** *****:** ************** * .******* **:

tomato TCAAAAAGTTTGCATTTTGTTCACTCATCTTATAGTCTTATGTGGCTATCTCAAGTTCCT 2294

potato TCAAAGAGTTTACATTTTGTTCACTCCTCTTATAGTATCCATTGGCTAGCTCAGGTTCCT 2310

pepino TCAAAAAGTTTGCATTTTGTTCACTCCTCTTATAGTCTCATGTGGCTATCACAAGTTCCT 2313

eggplant TCGAAGAGTTTGCATTTTATTCACTCTTCTTATAGTCTCATGTGGCTATCTCAAGTTCCT 2301

Pepper TCAAAAAGTCTGCATTTTGTTCACTCCAGTTATAGTCTCATGTGGCTATCTCAAGTTCCT 2331

tobaco TCCAACAGTTTGCACTTTGTTCACTCCAGTTACAGTCTCATGTGGCTATCTCAAGTTCCT 2331

** ** *** *.** ***.******* : *** ***.* .: ****** *:**.******

tomato AATTTAATTGAAAAGAACAAGGGGAATATTTACATGGCAAGTACAAGTCCACCAAGTGTT 2354

potato AATTTCATTGAGAAGAACAAGGGGAATATTTACATGTCAAGTACAAGTCCACCAAGTGTT 2370

pepino AATTTAATTGAAAAGAACAAGGGGAATATTTACATGGCAAGTACAAGTCCACCAAGTGTT 2373

eggplant AATTTCCTTGAAAAGAACAAGGGGAATATTTACATGTCAAGTACAAGTCCACCAAGTATT 2361

Pepper AATTTGATTGAAAAGAACAAGGGGAATATTTACATGTCAAGTACAAGTCCACCAAGTGTT 2391

tobaco GATGCAGTTGAGAATAACAAGGGGAACATTTACATGGCAAGTACAAGTCCGCCAAGTGTA 2391

.** ****.** *********** ********* *************.******.*:

tomato ATAAAAGCATATTACAAGCAATATGAAAAAGATTTTTCAATTTTTTTGAAATATCGTTCG 2414

potato ATAAAAGCATACTACAAACAATATGAAAATGATTTTTCAAATTTTCTGAAATATCGTTCA 2430

pepino ATAAAAGCATATTATAAGCAATATGAAAAAGATTTTTCAATTTTTTTGAAATATCGTTCA 2433

eggplant ATAAAAGCATACTACAAGCAATTTGGAAAAGATTTTTCAAATTTTTTGAAATATCGTTCA 2421

Pepper ATAAAAGCATATTACAAGCAATATGGAAAAGATTTTACAAATTTTCTGAAATATCGTTCT 2451

tobaco ATTAAAGCATACTACAAACAATATGAAAAAGATTTTTCCAATTTTCTCAAGTATCGTTCA 2451

**:******** ** **.****:**.***:******:*.*:**** * **.********

tomato GAAGAATTGATGAAAGGTGGAAAAATGGTATTAACATTTTTAGGAAGAGAAAGTGAAGAT 2474

potato GAGGAATTGATGAAAGGTGGAAAAATGGTATTAACATTTTTAGGAAGAGAAAGTGATGAT 2490

pepino GAAGAATTGATGAAAGGTGGAAAAATGGTATTAACATTTTTAGGAAGAGAAAGTGAAGAT 2493

eggplant GAGGAATTGATGAAAGGTGGAAAAATGGTATTAACATTTTTAGGAAGAGAAAGTGAAGAT 2481

Pepper GAAGAATTGATGAAAGGTGGGAAAATGGTATTAACATTTTTAGGAAGAGAAAATGAAGAT 2511

tobaco GAAGAATTGGTGAAAGGTGGGAAGATGGTTTTAACATTTTTAGGAAGAGAAAGTGAGGAT 2511

**.******.**********.**.*****:**********************.*** ***

tomato CCTTTTAGCAAAGAATGTTGTTATATTTGGGAGCTTTTATCCATGGCTCTTAATGAATTG 2534

potato CCTTCTAGCAAAGAAGGTTGTTATATCTGGGAGCTTCTAGGAATGGCCCTTAATGAATTG 2550

pepino CCTTCTAGCAAAGAATGTTGTTATATTTGGGAGCTTTTATCCATGGCTCTTAATGAATTG 2553

eggplant CCATCTAGCAAAGAATGTTGTTATATTTGGGAGCTTTTATCCATGGCCCTTAATGAATTG 2541

Pepper CCCTCTAGCAAAGAATGTTGCTATATTTGGGAACTTTTATCCATGGCCCTTAATGAATTG 2571

tobaco CCAACTAGCAAAGAATGCTGCTATATTTGGGAGCTTTTAGCCATGGCACTCAATGAGTTG 2571

** : ********** * ** ***** *****.*** ** .***** ** *****.***

tomato GTTTTAGAGGGATTGATTGAAGAAGAGAAAGTGGATTCATTTAATATTCCTCAATATACA 2594

potato GTTATTGAGGGATTAATAGAAGAAGAGAAAGTGGATTCATTCAACATTCCTAATTATACA 2610

pepino GTTTTAGAGGGATTGATAGAAGAAGAGAAAGTGGATTCATTTAACATTCCTCAATATACA 2613

eggplant GTTTTAGAGGGATTGATAGAAGAAGATAAAGTGGATTCATTTAACATTCCTCAATATACA 2601

Pepper GTTGTTGAGGGATTGATAGAAGAAGAGAAATTGGATGCCTTCAACATTCCTCAATATACA 2631

tobaco GTTGTAGAGGGATTGATAGAAGAAGAGAAAGTGGACTCATTCAACATTCCTCAATATACA 2631

*** *:********.**:******** *** **** *.** ** ******.*:******

tomato CCATCACAAGGAGAAGTGAAATATGTAGTTGATAAAGAAGGTTCATTCACTATAAATAAA 2654

potato GCATCACCATCAGAAGTGAAGTACTTAGTTGATAAAGAAGGTTCATTCACTATTAATAAA 2670

pepino CCATCACCAGCAGAAGTGAAGTACATAGTTGATAAAGAAGGTTCATTCACAATTAATAGA 2673

eggplant CCATCACCAGGAGAAGTGAAGTGTATAGTTGAAAAGGAAAGTTCATTCATTATTAATAGA 2661

Pepper CCATCACCAGCAGAAGTGAAGTACATAGTTGAGAAAGAAAATTCATTCACCATTAATAGA 2691

tobaco CCATCACCAGAAGATGTGAAATACGCAGTTGAGAAGGAGGGATCATTCACCATTAATCAA 2691

******.* ***:*****.*. ****** **.**...:******* **:***..*

tomato TTGGAAACTACAAGAGTTCATTGGAATAATGCTTCTAAT---AATATTGAAAATATTAAT 2711

potato TTGGAAACTACAAGAGTCCATTGGAATTATGCTTCTAATACTAATAATGAGAATATTTAT 2730

pepino TTGGAAACTACAAGAGTCCATTGGAATAATGCTTCTAAT---AATAATGAAAATATTAAT 2730

eggplant TTGGAAACTACAAGAGTCCATTGGAATAATGCTTCTAAT---AATAATGAAAATATCAAT 2718

Pepper TTGGAAGCTACAAGAATCCATTGG---AATGCTTCTAAT---------GATCATATTAAT 2739

tobaco TTGGAAGCTACAAGAGTCCAATGG---AATGCTTGTAAT---------GAGAACCACAAA 2739

******.********.* **:*** :****** **** ** .* .: :*:

tomato AATGATG---GTTACAATGTGTCAAAGTGCATGAGAGCTGTGGCTGAGCCTTTGCTTCTG 2768

potato AATAATGGTGGTTACAATTTATCAAGGGCCATAAGAGCTGTGGCTGAGCCTTTGCTTGTG 2790

pepino ATTAATGATGGTTACAATGTGTCAAGGTGCATGAGAGCTGTGGCTGAGCCTTTGCTTATG 2790

eggplant GATGGTG---GTTACAATGTGGCAAGGTGCATGAGAGCTGTGGCTGAGCCTTTGCTTGTG 2775

Pepper GGTG------GTTATAATGTGTCAAGGTGCATGAGAGCTGTGGCTGAACCTTTACTTGTT 2793

tobaco AATGGTG---GTTACAGTGTCTCAAGGTGTATGAGAGCTGTGGCTGAGCCTTTGCTTGTG 2796

. *. **** *.* * ***.* **.**************.*****.*** *

tomato AGCCAATTTGATCCAAAATTGATTGATTTAGTCTTCCAAAAGTATGAAGAGATTGTCTCC 2828

potato AGCCAATTTGATCCAAAATTGATGGATTTAATCTTCCAAAAATATGAAGAGATTGTTTCC 2850

pepino AGCCAATTTGATCCAAAATTGATTGATTTAGTCTTCCAAAAATATGAAGAGATTGTTTCT 2850

eggplant AGCCAATTTGATCCCAAATTGATGGATTTAGTGTTTGAAAAATATGAAGAGATTGTTTCC 2835

Pepper AGCCAATTTGGTCCAAAATTGATGGATTTAGTGTTCCAAAAATATGAAAAAATT------ 2847

tobaco AGCCAATTTGGCGAGGAATTAATGGATTTAGCATTCCACAAATACGAAGAGATTATATCT 2856

**********. . .****.** ******. ** *.**.** ***.*.***

tomato AAGTGTATGGCTAAAGAGGATACTGAGTTTATAAATGTTACTGTCTCCTTGACCAAGAAA 2888

potato GACTGCATGTCCAAAGAGAAAACCGAGTTTATAAATATCATAGTCTCCTTGACCAAAAAA 2910

pepino GAGTGCATGGCTAAAGAGAAAACCGAGTTTATAAATGTCACCGTCTCCTTGACCAAGAAA 2910

eggplant GAGTGCATGACTAAGGAGAAAACCGAGTTTATAAATGTCACTGTCTCATTGACCAAGAAA 2895

Pepper ---------------------------ATTATAAATGCCACTGTCTCATTGACCAAGAAA 2880

tobaco GAGTGCATGTCTAAAGAGCAAACTGAGTTTACAAATGTCACTGTCTCATTGACCAAAAGA 2916

:*** ****. * *****.********.*.*

tomato AAATAAATGTCGACTACTGTAGGCCAAGTCATTCGTTGCAAAGCTGCTGTGGCATGGGAA 2948

potato ACCTAAATGTCGACTACTGTAGGCCAAGTCATTCGTTGCAAAGCTGCTGTGGCATGGGAG 2970

pepino AAGTAAATGTCGACTACTGTAGGCCAAGTTATTCGTTGCAAAGCTGCTGTGGCATGGGAG 2970

eggplant AAATAAATGTCAAACACTGTTGGGCAAGTCATTCGTTGCAAAGCTGCTGTGGCATGGGAA 2955

Pepper AAATAAATGTCAAGCACTATAGGACAAGTCATTCGTTGCAAAGCTGCTGTGGCATGGGAG 2940

tobaco AATTAAATGTCAAGCACTGTCGGGCAAGTCATTCGTTGCAAAGCTGCTGTGGCATGGGAG 2976

*. ********.* ***.* ** ***** *****************************.

tomato GCTGGTAAGCCATTAGTGATGGAGGAAGTAGATGTTGCTCCTCCACAGAAAATGGAAGTT 3008

potato GCTGGTAAGCCATTAGTGATGGAGGAAGTGGATGTTGCTCCTCCACAGAAAATGGAAGTT 3030

pepino GCTGGTAAGCCATTAGTGATCGAGGAAGTGGATGTTGCACCTCCACAAAAAATGGAAGTT 3030

eggplant GCTGGAAAGCCATTGGTGATTGAAGAAGTGGAAGTGGCACCTCCACAGAAAATGGAAGTT 3015

Pepper GCTGGGAAGCCATTAGTGATAGAGGAAGTGGAAGTGGCACCTCCACAAAAAATGGAAGTT 3000

tobaco GCAGGGAAGCCATTAGTGATGGAAGAAGTGGAGGTGGCACCTCCACAAAAAATGGAAGTT 3036

**:** ********.***** **.*****.** ** **:********.************

tomato CGTCTTAAGATCCTCTATACTTCACTCTGTCATACTGATGTATACTTCTGGGAAGCTAAG 3068

potato CGTCTTAAGATCCTCTACACTTCACTCTGTCATACTGATGTATACTTCTGGGAAGCTAAG 3090

pepino CGTCTTAAGATTATCTACACTTCACTCTGTCATACTGATGTCTATTTCTGGGAAGCTAAG 3090

eggplant CGTCTTAAGATCCTTTACACTTCACTCTGTCATACTGATGTCTACTTCTGGGAAGCTAAG 3075

Pepper CGTCTTAAGATTCTCTACACTTCTCTATGTCATACTGATGTCTACTTCTGGGAAGCTAAG 3060

tobaco CGTCTTAAGATCCTCTACACTTCTCTCTGCCATACTGATGTCTACTTCTGGGAAGCAAAG 3096

*********** .* ** *****:**.** ***********.** ***********:***

tomato TGTTGTCAGGTCAAAATCCAGTCTTTCCTCGAATTCTTGGACATGAAGCAGCAGGGTATG 3128

potato --------GGTCAAAATCCAGTCTTTCCTAGAATTCTTGGACATGAAGCAGCAGG----G 3138

pepino --------GGACAAAATCCGGTCTTTCCTCGAATTCTTGGACATGAAGCAGCAGG----T 3138

eggplant --------GGCCAAAATCCAGTCTTTCCTCGAATTCTTGGACATGAAGCATCAGG----G 3123

Pepper --------GGCCAAAATCCAGTCTTTCCTCGAATTCTTGGACATGAAGCAGCAGG----G 3108

tobaco --------GGCCAAAATCCAGTCTTTCCTCGAATTCTTGGACATGAAGCAGCAGG----G 3144

** ********.*********.******************** ****

tomato ATTGTGGAGAGTGTTGGAGAGGGAGTAACAGACCTTGCACCAGGAGACCATGTTCTTCCT 3188

potato ATTGTGGAGAGTGTTGGAGAGGGAGTAACAGAACTTGCACCAGGAGACCATGTTCTTCCT 3198

pepino ATTGTGGAGAGTGTTGGAGAGGGAATAACAGAACTTGCACCAGGAGACCATGTTCTTCCT 3198

eggplant ATTGTGGAGAGTGTTGGAGAGGGAGTAACAGAACTTGCACCAGGAGACCATGTTCTTCCT 3183

Pepper ATTGTGGAGAGTGTTGGGGAGGGAGTTATAGAACTTGCACCAGGGGATCATGTTCTTCCT 3168

tobaco ATTGTGGAGAGTGTTGGAGAGGGAGTAACAGAACTTGCACCAGGAGACCATGTTTTGCCT 3204

*****************.******.*:* ***.***********.** ****** * ***

tomato GTCTTTACAGGGGAATGTAAAGATTGTGCTCACTGTAAATCTGAAGAAAGCAATATGTGT 3248

potato ATCTTTACAGGAGAATGCAAAGATTGTGCTCACTGCAAATCTGAAGAAAGCAACATGTGC 3258

pepino GTCTTTACAGGGGAATGCAAAGAATGTGCTCACTGCAAATCGGAAGAAAGCAATATGTGC 3258

eggplant GTCTTTACAGGGGAATGCAAAGATTGCGCTCACTGTAAATCTGAAGAAAGCAATATGTGC 3243

Pepper GTCTTTACAGGGGAATGCAAAGATTGTTCTCATTGTAAATCAGAAGAAAGCAATATGTGT 3228

tobaco GTGTTCACAGGGGAATGTAAAGATTGTGCTCACTGCAAATCGGAAGAAAGCAATATGTGT 3264

.* ** *****.***** *****:** **** ** ***** *********** *****

tomato AGCCTCTTAAGGATTAACACTGACAGGGGAGTGATGCTTAATGATGGAAAATCAAGATTT 3308

potato AGTCTCTTGAGGATTAACACTGACAGGGGAGTGATGATTAATGATGGACAATCAAGGTTT 3318

pepino AGCCTCTTGAGGATCAACACTGACAGGGGAGTGATGATTCATGATGGACAATCAAGGTTT 3318

eggplant AGCCTCTTAAGGATCAACACTGACAGGGGAGTGATGATTCATGATGGCAAGTCAAGGTTT 3303

Pepper AGCCTCTTAAGGATTAACACCGACAGGGGAGTGATGATTCATGATGGACAATCAAGATTT 3288

tobaco AGCCTCTTAAGGATTAACACTGATAGGGGAGTAATGATTCAAGATGGACAATCAAGATTT 3324

** *****.***** ***** ** ********.***.**.*:*****..*.*****.***

tomato TCCATCAATGGAAACCCCATTTACCATTTTGTTGGGACCTCTACTTTTAGTGAGTACACC 3368

potato TCCATCAATGGAAAGCCCATTTACCATTTTGTTGGGACCTCCACTTTTAGTGAGTACACT 3378

pepino TCCATCAATGGAAAACCTATTTACCATTTTGTTGGGACCTCCACTTTTAGTGAGTACACT 3378

eggplant TCCATCAATGGGAAGCCCATTTACCATTTTGTTGGGACCTCCACTTTTAGTGAGTACACT 3363

Pepper TCAATCAATGGGAAGCCCATTTACCATTTTGTTGGGACCTCCACTTTTAGTGAATATACT 3348

tobaco TCCATAAATGGAAAGCCTATTTACCATTTTGTTGGGACTTCTACCTTTAGTGAGTACACT 3384

**.**.*****.** ** ******************** ** ** ********.** **

tomato GTGGTTCATGTTGGATGTGTTGCAAAAATTAACCCTCTTGCTCCTCTTGACAAAGTATGT 3428

potato GTGGTTCATGTTGGATGTGTCGCCAAAATTAACCCCCTTGCTCCTCTTGACAAAGTATGT 3438

pepino GTGGTTCATGTTGGATGTGTTGCGAAAATTAACCCCCTTGCTCCTCTTGACAAAGTATGT 3438

eggplant GTCGTTCATGTTGGATGTCTCGCCAAAATTAACCCCGTTGCTCCACTTGATAAAGTATGT 3423

Pepper GTGGTTCATGTTGGATGTGTCGCGAAAATTAACCCCCTTGCTCCTCTTGACAAAGTATGT 3408

tobaco GTGGTTCATGTTGGTTGTGTTGCTAAAATCAACCCCCTTGCTCCTCTTGACAAAGTTTGT 3444

** ***********:*** * ** ***** ***** *******:***** *****:***

tomato GTCCTTAGTTGTGGAATTTCGACAGGCCTTGGAGCAAGTTTGAATGTTGCTAAACCAACA 3488

potato GTCCTTAGTTGTGGAATCTCGACAGGCCTTGGAGCAACTTTGAATGTTGCTAAACCAACA 3498

pepino GTCCTTAGTTGTGGAATCTCGACAGGCCTTGGAGCAACTTTGAATGTTGCTAAACCAACA 3498

eggplant GTGCTTAGCTGTGGAATTTCGACAGGCCTTGGTGCCACACTGAATGTTGCTAAACCGAAA 3483

Pepper GTCCTTAGTTGTGGAATCTCGACAGGCCTTGGTGCAACTTTGAATGTTGCTAAACCAACA 3468

tobaco GTCCTCAGTTGTGGAATCTCTACAGGTCTTGGTGCAACTTTGAATGTGGCTAAACCAACA 3504

** ** ** ******** ** ***** *****:**.* : ******* ********.*.*

tomato AAAGGCTCAAGTGTGGCTATATTTGGACTAGGAGCTGTAGGCCTCGCGGCTGCAGAAGGA 3548

potato AAAGGCTCAAGTGTGGCTATTTTTGGACTAGGAGCTGTAGGCCTTGCAGCCGCAGAAGGA 3558

pepino AAAGGTTCAAGTGTGGCTATATTTGGACTAGGGGCTGTAGGCCTTGCTGCTGCAGAAGGA 3558

eggplant AAAGGTTCAAGTGTGGCTATATTTGGACTAGGGGCTGTAGGCCTTGCAGCTGCAGAAGGA 3543

Pepper AAAGGCTCAAGTGTGGCTATATTTGGACTAGGGGCTGTAGGCCTTGCTGCTGCAGAAGGA 3528

tobaco AAAGGATCAAGTGTGGCTATATTTGGACTGGGGGCTGTAGGCCTTGCTGCTGCAGAAGGA 3564

***** **************:********.**.*********** ** ** *********

tomato GCCAGAATTGCTGGTGCCTCGAGGATAATTGGTGTTGATTTAAATGCTAGTAGATTTGAG 3608

potato GCCAGAATTGCTGGTGCTTCAAGGATAATTGGTGTTGATTTAAATGCTAGTAGATTTGAG 3618

pepino GCAAGAATTGCTGGTGCTTCAAGGATAATTGGTGTTGATTTAAATGCTAGCAGATTTGAG 3618

eggplant GCAAGAATTGCTGGTGCTTCAAGGATAATTGGTGTTGATTTAAATGCTAGCAGATTTGAG 3603

Pepper GCAAGAATTGCTGGTGTGTCAAGGATAATTGGTGTTGATTTAAATGCTAGCAGATTTGAG 3588

tobaco GCAAGAATTGCTGGTGCTTCAAGGATAATTGGTGTTGATTTAAATGCTAGCAGATTTGAG 3624

**.************* **.***************************** *********

tomato CAAGCTAAGAAATTTGGTGTGACAGAGTTTGTGAACCCAAAGGACTATAGTAAACCAGTT 3668

potato CAAGCTAAGAAATTTGGTGTGACAGAGTTTGTGAACCCAAAGGACTATAGTAAACCAGTT 3678

pepino CTAGCTAAGAAATTTGGTGTGACAGAGTTTGTGAACCCAAAGGACCACAGTAAACCAGTT 3678

eggplant CAAGCTAAGAAATTTGGTGTGACTGAATTTGTGAATCCAAAGGATTATAGTAAACCAGTT 3663

Pepper CAAGCTAAGAAATTTGGTGTGACAGAGTTTGTGAATCCAAAGGATCATAGTAAACCAGTT 3648

tobaco TTAGCTAAGAAATTTGGTGTGACAGAGTTTGTGAATCCTAAGGATCATAATAAACCAATT 3684

:*********************:**.******** **:***** * *.*******.**

tomato CAAGAGGTAATTGCTGAGATGACTGATGGCGGAGTCGATAGGAGTGTGGAATGTACTGGT 3728

potato CAAGAGGTAATTGCTGAGATGACTGATGGTGGAGTCGATAGGAGTGTGGAATGTACTGGC 3738

pepino CAAGAGGTAATTGCTGAGATGACTGATGGCGGAGTCGATAGGAGTGTGGAATGTACTGGC 3738

eggplant CAAGAGGTAATTGCTGAGATGACTGATGGCGGAGTTGATAGGAGTGTGGAATGTACTGGC 3723

Pepper CAAGAGATAATTGCTGAGATGACTGATGGCGGAGTCGATAGGAGTGTGGAATGTACTGGC 3708

tobaco CAAGAGGTAATTGCTGAGATGACTGATGGCGGAGTAGACAGGAGTGTGGAATGTACAGGC 3744

******.********************** ***** ** *****************:**

tomato CACATTGATGCTATGATTTCAGCATTTGAATGTGTCCATGATGGCTGGGGAGTGGCGGTT 3788

potato CATATTGATGCTATGATTTCAGCATTTGAGTGTGTCCATGATGGCTGGGGAGTGGCTGTA 3798

pepino CATATTGATGCTATGATTTCAGCATTTGAATGTGTCCATGATGGGTGGGGAGTGGCTGTT 3798

eggplant CATATTGATGCTATGATTTCAGCATTTGAATGTGTCCATGATGGCTGGGGAGTGGCTGTT 3783

Pepper CATATTGATGCTATGATTTCAGCATTTGAATGTGTTCATGATG----------------- 3751

tobaco CACATTGATGCTATGATTTCAGCATTTGAATGTGTCCATGATGGTTGGGGAGTGGCTGTT 3804

** **************************.***** *******

tomato CTTGTTGGTGTACCCCATAAAGAAGCTGTGTTCAAGACACATCCTCTGAACTTTTTGAAT 3848

potato CTTGTTGGAGTACCCCATAAAGAAGCTGTGTTCAAGACACACCCTATGAACTTTTTGAAT 3858

pepino CTTGTTGGAGTACCCCATAAAGAAGCTGTGTTCAAGACAAACCCTATGAACTTTTTGAAT 3858

eggplant CTTGTTGGAGTGCCCCATAAAGAAGCTGTGTTCAAGACACAGCCTATGAACTTTTTGAAC 3843

Pepper ------------------TATGTTTCTGTCATCAA--AATTTCCTTAGTTATATGT---- 3787

tobaco CTTGTTGGAGTACCACATAAAGAAGCTGTATTCAAGACACACCCTATGAAGTTGTTGAAT 3864

:*:*:: **** :**** .* : *** :*:: *: *

tomato GAACGGACTCTCAAAGGAACCTTCTTTGGAAACTACAAACCTCGTTCGGATATTCCTTGT 3908

potato GAAAGGACTCTCAAAGGAACCTTCTTTGGAAACTACAAACCTCGTTCGGATATTCCTTCT 3918

pepino GAAAGGACTCTCAAAGGAACCTTCTTTGGAAACTACAAACCTCGTTCGGATATTCCTTCT 3918

eggplant GAAAGGACTCTGAAAGGAACCTTCTTTGGAAACTACAAGCCCCGTTCGGATATTCCTTGT 3903

Pepper ----------------------TCTATTACAACATTAATCTTG------ATGAAACTTGT 3819

tobaco GAAAGGACTCTCAAGGGGACATTCTTTGGAAACTACAAGCCCCGTTCTGATCTTCCTTCT 3924

***:* ..***:: ** * ** ::.*** *

tomato GTTGTTGAGAAATACATGAACAAAGAACTTGAATTGGAGAAATTCATCACTCATACACTT 3968

potato GTTGTTGAGAAATACATGAATAAAGAACTTGAATTGGAGAAATTCATCACTCATACACTT 3978

pepino GTTGTTGAGAAATACATGAACAAAGAACTTGAATTGGAGAAGTTCATCACTCATACACTC 3978

eggplant GTTGTTGAGAAATACATGAACAAAGAACTTGAATTGGAGAAGTTCATCACTCATACAATC 3963

Pepper ----------AATTTATTTTCAGG--------GTTGGGGAG------------------- 3842

tobaco GTCGTCGAGAAATACATGAACAAGGAACTTGAATTGGAGAAGTTCATCACTCACAAACTC 3984

***: ** :: *.. .****.**.

tomato CCATTTGCTGAAATCAATAAGGCTTTCGATTTAATGCTGAAGGGAGAAGGCCTTCGTTGC 4028

potato CCATTTGCTGAAATCAATAAGGCTTTTGATTTAATGTTGAAGGGAGAAGGCCTTCGTTGC 4038

pepino CCATTTGCTCAAATCAATAAGGCTTTTGATTTAATGTTGAAGGGAGAAGGCCTTCGTTGC 4038

eggplant CCATTTTCTCAAATCAACAAGGCTTTTGATTTAATGTTGAAGGGAGAAGGCCTTCGTTGC 4023

Pepper -------------------TGGCTG-----TTCTTGTTGGAG------------------ 3860

tobaco CCATTTGCTGAAATCAACAAGGCATTTGATTTAATGTTGAAGGGAGAAGGCCTTCGTTGC 4044

:***: **.:** **.**

tomato ATCATCACCATGGCGGACTAAATGACTTTAACACTTCAATCATCAGCTTCTTTTATCAAT 4088

potato ATCATCACCATGGAGGACTAAATGACTTTAACACTTCAATCATCAGCTTCTTTTATCAAT 4098

pepino ATCATCACCATGGAGGACTAAATGACTTTAACACTTCAATCATCAACTTCTTTAATCAAT 4098

eggplant ATCATCACCATGGAGGACTAAATGACACTAACACTTCAATCATCAACTTCTTTTATCAAT 4083

Pepper ---TACCCCAT--------AAATGACTTTAACACTCCAATCATCAACTTCTTTTATCAAT 3909

tobaco ATCATCAACATGGAGGACTAAATGACTTTAACACTTCGTTCATCAACTTCTTTTATTAAT 4104

::*..*** *******: ******* *.:******.*******:** ***

tomato TTCAAAGAAACCAAAGGTGTTAAAACACCTGATGAGTTCTTAGGAATGGTTTCTTTTGCA 4148

potato TTCAAAGAAACCAAAGGTGTTAAAGCACCTGATGAGTTCTTAGGAATGGTTTCTTTTGCA 4158

pepino TTCAAAGAAACCAAAGGTGTTAAAACACCTGATGAGTTCTTAGGAATGGTTTCTTTTGCA 4158

eggplant TTGAAAGACACCAGAGGTGTTAAAACACCTGATGATTTCTTAACCATGGTTTCTTTTGTA 4143

Pepper TTAAAAGAAACCAAAGGTGTTAAAACACCTGATGATTTCTTAAGCATGGTTTCTTTTGCA 3969

tobaco ATAAAAGAAACCAAAGGTGTTAAAACACCTGATGATTTCTTAGGCATGGTTTCTTATACA 4164

:* *****.****.**********.********** ******. .**********:*. *

tomato CAAGCCAAGCCATCATCATGTCGGCTAGTTGCGAAAAGTTCGATGCAAGAAGCTCAACTC 4208

potato CAAGCCAAGCCATCAT---GCCGGCTAGTCGCGAAAAGTTCGATGCAAGAAGCTCAACTC 4215

pepino CAAGCCAAGCCATCAT---GCCGGCTAGTTGCGAAAAGTTCGATGCAAGAAGCTCAACTC 4215

eggplant CAAACCAAGCCATCTT---GCCGTCTAGTCGCGAAGAGTTCGATGCAAGAAGCTCAACTC 4200

Pepper CAATCCAAGCCATCTT---GCCGTCTCGTAGCAAAAAGCTCGATGCAAGAAGCTCAACTC 4026

tobaco CAAACCAAGCCATCTT---GTCGTCTCATAACAAAGAATTCAATGCAAGAAGCTCACCTC 4221

*** **********:* * ** **..* .*.**.*. **.**************.***

tomato TCCCATGAGAGAATCATGGAAGTGAGGAAAATTGAGAAAAGAGAGAAGCTACATGAGTTA 4268

potato TCCCATGAGAGAATCATGGAAGTGAAGAAAATTGAGAAAAGAGAGAAGCTACATGAGTTA 4275

pepino TCCCATGAGAGAATCATGGAAGTGAGGAAAACTGAGAAAAGAGAGAAGCTACATGAGTTA 4275

eggplant TCTCATGAGAGAATCATGGAAGTGAGGAAAAAT---------GAGAAGCTATATGAGCTA 4251

Pepper TCCCATGAGACAATCATGGAAGGGAGGAAAAATGAGAAAAGAGAGAAGCTACATGAGTTA 4086

tobaco TCTCATGAGAGAGTTATCGTT---------AATGAGAAAAGAGAAAAACTTCATGTGCTA 4272

** ******* *.* ** *:: * * **.**.**: ***:* **

tomato ACAGCTAATCATAGCAATAGTAGTACAAGGGTACCTGTTTTTGTGATGCTTCCACTTGAC 4328

potato CCAGCTAATCACAGCAATAGAAGTACAAGGGTACCTGTTTTTGTGATGCTTCCACTTGAC 4335

pepino ATAGCTAATCACAGCAATAGTAGTACAAGGGTACCTGTTTTTGTGATGCTTCCACTTGAC 4335

eggplant ACAGATAATCACAGCAATAGTAGTACAAAGGTACCTGTTTTTGTGATGCTTCCACTTGAC 4311

Pepper ACAGCTAGTCACAGCAATAGCAGTAGAAAGGTACCGGTTTTTGTGATGCTCCCACTTGAC 4146

tobaco ACAACTACTCACAGTAATAGTAGTACAAAGGTACCTGTTTTTGTGATGTTGCCACTTGAT 4332

. *..** *** ** ***** **** **.****** ************ * ********

tomato ACCATGACTATGGGAGGGAACTTGAACAGGCCACGAGCGATGAATGCGAGTTTGATGGCG 4388

potato ACCATGACTATGGGAGGGAACTTGAACAGGCCACGAGCGATGAATGCGAGTTTGATGGCG 4395

pepino ACCATGACCATGGGAGGGAACTTGAACAGGCCACGAGCGATGAATGCGAGTTTGATGGCG 4395

eggplant ACCATGACTATGGGAGGGAACTTGAACAGGCCACGAGCGATGAATGCGAGTTTGATGGCC 4371

Pepper ACCATGACCATGGGAGGGAACTTGAACAAGCCACGAGCGATGAACGCGAGTTTGATGGCC 4206

tobaco ACTATGACTATGGGAGGGAACTTGAACAAGCCACGAGCGATGCACGTGAGTTTGATGGCG 4392

** ***** *******************.*************.* * ************

tomato TTGAAAAGTTCTGGAGCTGAAGGGGTGATGGTGGATGCTTGGTGGGGATTGGTGGAGAAA 4448

potato TTGAAAAGTTCTGGAGCTGAAGGGGTGATGGTGGATGCTTGGTGGGGATTGGTGGAGAAA 4455

pepino TTGAAAAGTTCTGGAGCTGAAGGGGTTATGGTGGATGCTTGGTGGGGCTTGGTGGAGAAA 4455

eggplant TTGAAAAGTTCTGGAGTTGAAGGGGTCATGGTGGATGCTTGGTGGGGATTGGTGGAGAAA 4431

Pepper TTGAAAAGTTCTGGAGCTGAAGGGGTTATGGTGGATGCTTGGTGGGGATTGGTAGAGAAA 4266

tobaco TTGAAAAGTGCTGGAGTTGAAGGAGTAATGGTAGATGCTTGGTGGGGTTTGGTAGAAAAA 4452

********* ****** ******.** *****.************** *****.**.***

tomato GATGGACCTTTGAAGTATAATTGGGAAGGATATGCTGAACTTGTAAATATGTGTCGAGAA 4508

potato GATGGACCTTTGAAGTATAATTGGGAAGGATATGCTGAGCTTGTAAAGATGTGTCAAGAA 4515

pepino GATGGACCTTTGAAGTATAATTGGGAAGGATATGCTGAACTTGTAAAGATGTGTCAAGAA 4515

eggplant GATGGACCTTTGAAGTATAATTGGGAAGGATATGCTGAACTTGTAAAGATGTGTCAAGAA 4491

Pepper GATGGACCGTTGAAGTATAACTGGGAAGGGTATGCTGAACTTGTCAAGATGTGTCAAGAA 4326

tobaco GATGGACCTTTGAAGTATAACTGGGAAGGTTATGCTGAACTTGTTAAGATGTGTAAAGAA 4512

******** *********** ******** ********.***** ** ******..****

tomato CATGGGTTGAAGCTTCAAGTTGTCATGTCTTTTCATCAGTGTGGAGGAAATGTTGGAGAC 4568

potato CATGGATTGAAGCTTCAAGTTGTCATGTCTTTTCATCAGTGTGGAGGAAATGTTGGAGAT 4575

pepino CATGGATTGAAGCTTCAAGTTGTCATGTCTTTTCATCAGTGTGGAGGAAATGTTGGAGAC 4575

eggplant CATGGCTTGAAGCTTCAAGTTGTCATGTCTTTTCATCAGTGTGGAGGAAATGTTGGAGAC 4551

Pepper CATGGCTTGAAGCTTCAAGTTGTCATGTCTTTTCATCAGTGTGGAGGAAATGTTGGAGAT 4386

tobaco CATGGCTTGAAACTTCAGATTGTTATGTCTTTTCATCAGTGTGGAGGAAATGTTGGAGAC 4572

***** *****.*****..**** ***********************************

tomato TCCTGCAGTATTCCTCTACCTCCATGGGTACTCGAAGAAATCAGTAAGAATCCTGACCTT 4628

potato TCTTGCAGTATTCCTCTACCTCCATGGGTACTTGAAGAAATCAGCAAGAATCCTGACCTT 4635

pepino TCTTGCAGTATTCCTCTACCTCCATGGGTACTTGAAGAAATCAGCAAGAATCCTGACCTT 4635

eggplant TCCTGCAGTATTCCTCTACCTCCATGGGTACTTGAAGAAATCAGCAAGAATCCTGACCTT 4611

Pepper TCTTGCAGTATTCCTCTACCTCCATGGGTACTTGAAGAAATCAGCAAGAATCCTGACCTT 4446

tobaco TCTTGCAGTATTCCTCTACCTCCATGGGTACTTGAAGAAATCAGCAAGAATCCTGACCTT 4632

** ***************************** *********** ***************

tomato GTCTACACAGATAGATCAGGCCGGAGAAATCCCGAGTATCTATCCTTAGGTTGTGATATG 4688

potato GTCTACACAGATAGATCAGGCCGGAGAAATCCTGAGTATCTATCCTTAGGTTGTGATATG 4695

pepino GTCTACACAGATAGATCAGGCCGGAGAAATCCTGAGTATCTATCCTTAGGTTGTGATATG 4695

eggplant GTCTACACAGATAGATCAGGCCGGAGAAATCCCGAATACATATCCTTAGGTTGTGATATG 4671

Pepper GTCTACACAGATAGATCAGGCCGGAGAAATCCTGAGTATATATCCTTAGGTTGTGATATG 4506

tobaco GTCTACACAGATAGATCAGGCCGGAGAAATCCTGAGTATATTTCCTTAGGTTGTGATCAG 4692

******************************** **.** .*:***************.:*

tomato TTACCAGTACTCAAAGGAAGAACACCTATTCAAGTATACACTGACTATATGAGGAGCTTC 4748

potato TTACCAGTACTCAAAGGAAGAACACCTATTCAAGTATACACCGACTATATGAGGAGCTTC 4755

pepino TTACCAGTACTCAAAGGAAGAACACCTATTCAAGTATACGCCGACTATATGAGGAGCTTC 4755

eggplant GTACCAGTACTCAAAGGAAGAACACCCATTCAAGCATACACAGACTATATGAGGAGCTTC 4731

Pepper TTGCCAGTACTCAAAGGAAGAACACCCATTCAAGTATACGCCGACTATATGAGGAGCTTC 4566

tobaco TTACCAGTACTCAAAGGAAGAACACCTATTCAGGTCTATACTGACTTTATGAAGAGCTTC 4752

*.*********************** *****.* .** .* ****:*****.*******

tomato AGAGAGAGATTCAACAATTACTTGGGAAACATCATAGTGGAAATCCAAGTGGGAATGGGT 4808

potato AGAGAAAGATTCAACGATTACTTGGGAAACGTCATAGTGGAAATCCAAGTGGGAATGGGC 4815

pepino AGAGAAAGATTTAACGATTACTTGGGAAACGTCATAGTGGAAATTCAAGTGGGAATGGGT 4815

eggplant AAAGAAAGATTCAACGATTATCTGGGAAACGTCATAGTGGAAATTCAAGTGGGAATGGGT 4791

Pepper AGAGAAAGATTCAACGATTATTTGGGAAATGTCATAGTGGAAATTCAAGTGGGAATGGGT 4626

tobaco AGAGAAAGATTCAGCAATTACTTGGGAGATGTCATAGTGGAGATTCAAGTGGGAATGGGT 4812

*.***.***** *.*.**** *****.* .**********.** **************

tomato CCTTGTGGAGAGCTAAGATACCCAGCCTATCCAGAAAGCAATGGTACATGGAGGTTTCCT 4868

potato CCTTGTGGAGAGCTAAGATATCCAGCCTATCCCGAAAGCAATGGTACATGGAGGTTTCCT 4875

pepino CCTTGTGGGGAGCTAAGATATCCAGCCTATCCAGAAAGCAATGGTACATGGAGGTTTCCA 4875

eggplant CCTTGTGGGGAGCTAAGATATCCATCCTATCCAGAAAGCAATGGTACATGGAGGTTTCCT 4851

Pepper CCTTGTGGGGAGCTAAGATATCCAGCCTACCCAGAAAGCAATGGTACATGGAGGTTTCCT 4686

tobaco CCTTGTGGGGAACTCAGATATCCATCTTATCCAGAAAGCAATGGTACATGGAGGTTTCCT 4872

********.**.**.***** *** * ** **.**************************:

tomato GGAATTGGAGAATTCCAATGCTATGACAAGTACATGAGAGCTTCATTGGCGGCAGCAGCC 4928

potato GGAATTGGAGAATTCCAATGCTATGACAAGTACATGGGAGCTTCATTGGCAGCAGTGGCC 4935

pepino GGAATTGGGGAATTCCAATGCTATGACAAGTACATGAGAGCTTCATTGGCCGCAGCCGCC 4935

eggplant GGAATTGGTGAATTCCAATGCTATGACAAGTACATGAGAGCATCATTGGCAGCAGCCGCC 4911

Pepper GGAATTGGAGAATTCCAATGCTATGACATGTACATGAGAGCTTCACTAGCAGCAGCAGCC 4746

tobaco GGAATTGGAGAATTCCAGTGCTATGACAAGTACATGAGAGCTTCACTATCAGCAGCCGCC 4932

******** ********.**********:*******.****:*** *. * **** ***

tomato AAGGCAACTGGAAAGGATGACTGGGGCCAGGGAGGGCCTCATGATTCTGGGCAGTACAAC 4988

potato AAGGCAGCTGGAAAGGATGACTGGGGCCAGGGAGGGCCTCATGATTCTGGGAAGTACAAC 4995

pepino AAAGCAGCTGGAAAGGATGACTGGGGCCAGGGAGGGCCTCATGATTCTGGGCAGTACAAC 4995

eggplant AAGGCAGCTGGAAAGGATGACTGGGGCCAGGGAGGGCCTCATGACTCTGGGCAGTACAAT 4971

Pepper AAGGCAGCTGGAAAGGATGATTGGGGCCGGGGAGGGCCTCATGACTCTGGGCAGTACAAT 4806

tobaco AAGGCGGCCGGAAAAGATGACTGGGGCCAGGGAGGGCCTCATGACTCCGGACAGTACAAT 4992

**.**..* *****.***** *******.*************** ** **..*******

tomato CAGTTTCCCGAGGATACTGGATTTTTCCAACGGGATGGAACATGGAATAGTGACTATGGA 5048

potato CAGTTTCCTGAGGATACTGGATTTTTCCAGAGGGATGGAACATGGAACAGTGAATATGGA 5055

pepino CAGTTTCCCGAGGATACTGGATTTTTCCAGAGGGATGGAACATGGAACAGTGAATATGGA 5055

eggplant CAGTTTCCTGAGGATACTGGATTTTTCCGAAGGGATGGAACATGGAACAGTGAATATGGA 5031

Pepper CAGTTTCCCGAGGATACTGGATTTTTTCAAAGGGACGGAACATGGAACAGTGAATATGGA 4866

tobaco CAATTTCCTGAGGATACTGGATTTTTTCAAAGAGATGGAACATGGAACAGTGAATATGGA 5052

**.***** ***************** *...*.** *********** *****.******

tomato CAGTTCTTCCTAGAGTGGTATTCAGGAAAGCTACTGGAGCATGGTGACAGAATACTAGCA 5108

potato CAGTTCTTCCTAGAGTGGTATTCAGGAAAGCTACTGGAGCATGGTGACAGAATAGTAGCA 5115

pepino CAGTTCTTCCTAGAGTGGTATTCAGGAAAGCTACTGGAGCATGGTGACAGAATACTAGCA 5115

eggplant CAGTTCTTCCTTGAGTGGTATTCAGGAATGCTACTGGAGCATGGTGACAGAATCCTAGCA 5091

Pepper CAGTTCTTCCTAGAGTGGTATTCAGGAAAACTACTAGAGCATGGAGACAGGATCCTAGCA 4926

tobaco CAGTTCTTCCTAGAGTGGTATTCAGGAAAGCTAGTGGAGCACGGCGACAGAATCCTAGCA 5112

***********:****************:.*** *.***** ** *****.**. *****

tomato GCAGGAGAAAGTATATACCAAGGAACTGGGGCTAAACTATCTGGAAAGATAGCTGGAATT 5168

potato GCAGGAGAAAGTATATACCAAGGAACTGGGGCTAAACTATCTGGAAAGGTAGCTGGGATT 5175

pepino GCAGGAGAAAGTATATACCGAGGAACTGGGGCTAAACTATCTGGAAAGGTAGCTGGGATT 5175

eggplant GCAGCAGAAAGTATATACCAAGGAACTGGGGCTAAACTATCCGGAAAGGTAGCTGGGATT 5151

Pepper GCAGGAGAAAGTATATACCAAGGAACTGGGGCTAAGCTATCTGGAAAGGTAGCTGGGATT 4986

tobaco GCAGCAGAGGGTATATATCAAGGAACTGGGGCTAAACTATCTGGAAAGGTAGCTGGAATT 5172

**** ***..******* *.***************.***** ******.*******.***

tomato CATTGGCATTACAATACTAGATCACATGCTGCAGAGTTAACAGCAGGATATTATAATACA 5228

potato CATTGGCATTACAATACTAGATCACATGCTGCAGAGTTAACTTCAGGATATTATAATACA 5235

pepino CATTGGCATTACAATACTAGATCACATGCTGCAGAGTTAACAGCAGGATATTATAATACA 5235

eggplant CATTGGCATTACAATACTAGATCACATGCTGCAGAGTTAACAGCAGGATATTATAATACA 5211

Pepper CATTGGCATTACAATACTAGATCACATGCTGCAGAGTTAACAGCAGGATATTATAATACA 5046

tobaco CATTGGCATTACAATACTAGATCACATGCTGCGGAGTTAACAGCAGGATACTATAATACA 5232

********************************.********: ******* *********

tomato AGACACAGAGATGGTTATCTACCTATAGCACGTATGTTAGCGAAACACGGTGTTGTACTT 5288

potato AGACACAGAGATGGTTATCTACCTATAGCACGTATGTTAGCGAAACATGGTGCTGTACTG 5295

pepino AGACACAGAGATGGCTATCTACCTATAGCACGTATGTTAGCAAAACATCGTGTTGTACTT 5295

eggplant AGACACAGAGACGGCTATCTACCTATAGCACGTATGTTAGCGAAACATCGTGCTGTACTA 5271

Pepper AGACACAGAGACGGCTATCTACCTATAGCACGTATGTTAGCGAAACATCGAGTTGTACTT 5106

tobaco AGACACACAAATGGCTATCTACCTATAGCACGTATGTTCGCCAAACATCGTGTTGTGTTT 5292

******* *.* ** ***********************.** ***** *:* ***. *

tomato AACTTTACATGTATGGAAATGAGGGATGGTGAACAGCCCCAGAGTGCAAACTGCTCACCA 5348

potato AACTTTACATGTATGGAAATGAGGGATGGTGAACAGCCCCAGAGTGCAAACTGTTCACCA 5355

pepino AACTTTACATGTATGGAAATGAGGGATGGTGAACAGCCCCAGAGTGCAAACTGCTCACCA 5355

eggplant AACTTTACATGTATGGAAATGAAGGATGGTGAACAGCCCCAGAGTGCAAACTGCTCACCA 5331

Pepper AACTTTACATGCATGGAAATGAGGGATGGTGAACAGCCCCATAGTGCAAACTGCTCACCA 5166

tobaco AACTTTACATGTATGGAAATGAGGGATGGTGAACAGCCCCAGAGTGCAAACTGCTCACCA 5352

*********** **********.****************** *********** ******

tomato GAAGGCTTAGTTCGACAAGTTAAAACTGCAGCTAGAACTGCTGAAGTAGAACTTGCTGGA 5408

potato GAAGGCTTAGTTCGACAAGTTAAAACTGCAGCTAGAACTGCTGAAGTAGAACTTGCTGGA 5415

pepino GAAGGCTTAGTTCGACAAGTTAAAACTGCAGCGAGAACTGCTGAAGTAGAACTTGCTGGA 5415

eggplant GAAGGCTTGGTTAGACAAGTTAAAACTGCAGCCAAAACTGCTGGAGTAGAACTTGCTGGA 5391

Pepper GAAGGCTTAGTTCGACAAGTTAAAACTGCAGCGAGAACTGCTGGAGTAGAACTTGCTGGA 5226

tobaco GAAGGCTTAGTTCGACAAGTAAAAAACGCAACTACAACTGCTGAAGTAGAACTTGCCGGA 5412

********.***.*******:****. ***.* * ********.************ ***

tomato GAAAATGCTCTAGAAAGGTATGATGGAGGAGCATTTTCTCAAGTTTTGGCAACAAGCATG 5468

potato GAAAATGCTCTAGAAAGGTATGATGGAGGAGCATTTTCTCAAGTTTTGGCAACAAGCATG 5475

pepino GAAAATGCTCTAGAAAGGTATGATGGAGGAGCATTTTCTCAAGTTTTGGCAACAAGCATG 5475

eggplant GAAAATGCCCTAGAAAGGTATGACGGAGGAGCATTCTCTCAAGTATTGGCAACAAGCATG 5451

Pepper GAAAATGCTCTAGAAAGGTACGATGTAGGAGCCTTTTCTCAAGTTTTGGCAACAAGCATG 5286

tobaco GAAAATGCTCTAGAGAGGTATGATGGAGGAGCGTATTCTCAAGTTTTGGCAACAAGCAGA 5472

******** *****.***** ** * ****** *: ********:************* .

tomato TCAGATTCTGGAAATGGATTGAGTGCATTTACATTCTTACGGATGAACAAACGGTTGTTT 5528

potato TCAAATTCTGGAAATGGATTGAGTGCATTTACATTCTTGCGAATGAACAAACGGTTGTTT 5535

pepino TCAGATTCTGGAAATGGATTGAGTGCATTTACATTCTTGCGAATGAACAAACGGTTGTTT 5535

eggplant TCAGATTCTGGAAATGGATTGAGTGCATTTACATTCTTGCGAATGAACAAGCGGTTGTTT 5511

Pepper TCAGATTCTGGAAATGGGTTGAGTGCATTTACATTCTTGCGAATGAACAAACGGTTGTTT 5346

tobaco TCAGATTCCGGAAATGGATTGAGTGCATTCACATTCTTGAGAATGAACAAGCGGTTGTTT 5532

***.**** ********.*********** ********..*.********.*********

tomato GAGCCAGAAAATTGGCGGAATCTAGTGCAATTTGTGAAGAGCATGTCGGAAGGAGGTCGA 5588

potato GAGCCAGAAAATTGGCGGAATCTAGTGCAATTTGTGAAGAGCATGTCTGAAGGAGGTCGA 5595

pepino GAGCCAGAAAATTGGCGGAATCTAGTGCAATTCGTGAAGAGCATGTCGGAAGGAGGTCGA 5595

eggplant GAGCCAGAAAATTGGAGAAATCTAGTGCAATTCGTGAAGAGCATGTCGGAAGGAGGTCGA 5571

Pepper GAGCCAGAAAACTGGCGGAATCTAGTGCAATTCATAAAGAGCATGTCGGAAGGAGGTCGA 5406

tobaco GAGCCAGAAAATTGGCGGAATCTAGTGCAATTCGTGAAAAACATGTCAGATGGAGGTCGA 5592

*********** ***.*.************** .*.**.*.****** **:*********

tomato AATGCTACCCTTCCAGAGTGTGACTCAAGCAGGACAGACCTCTATGTAAGATTTATCAAA 5648

potato AATGCTAGCCTTCCAGAGTGTGACTCAAGCAGGACAGACCTCTATGTAAGATTTATCAAA 5655

pepino AAGGCTAGCCTTCCAGAGTGTGACTCAAGCAGGACAGATCTCTATGTAAGATTTATCAAA 5655

eggplant AATGCTAGCCTTCCAGAGTGTGACTCGAGCAGGACAGACCTCTATGTAAGATTTATCAAA 5631

Pepper AATGCTAGCCTTCCAGAGTGCGACTCGAGCAGGACAAACCTCTATGTAAGATTTATCAAA 5466

tobaco AATGCTACACTTCCAGAGTGTGACTCAAGCAGGACAGACCTCTATGTACATTTTGTCAAA 5652

** **** .*********** *****.*********.* *********..:***.*****

tomato GAGAGCCATTCTAAGAAAGCTACAGAGGTTGCAGTAGTGTAAATGACAACATTTCATACT 5708

potato GAGAGTCATTCTAAGAAAGCTACAGAGGTTGCAGTAGTGTAAATGACAACATTTCATACT 5715

pepino GAGAGTCATTCTAAGAAAGCTACAGAGGTTGCAGTAGTGTAAATGACAACATTTCATAGT 5715

eggplant AAGAGTCATTCTAAGGAAGCTACAGAGGTTGCAGTAGTGTAAATGACAAAATTTCATAGT 5691

Pepper GAAAGTCATTCTAAGAAAGCTACCGAAGTTGCAGTAGTGTAAATGACAACAATTCATAGT 5526

tobaco AAGAGTCATTCTAAGAAAACTACAGAGGTTGCACTAGTGTAAATGACAACATTTCATAGT 5712

.*.** *********.**.****.**.****** ***************.*:****** *

tomato AGCTTAGTTTACATTTTGTATGTGCTACTTCTTTTTTCACTTCCATTAAGTATCACATCT 5768

potato ACCTTAGTTTACATCTTGTATGTGCTACTTCTTTTTTCACTTCCATTAAGTATCACATCT 5775

pepino ACCTTAGTTTACATTTTGTATGTGCTACTTCTTTTTTCACTTCCATTAAGTATCACATCT 5775

eggplant AGCTTAGTTTACATTTTGTATGTGTTACTTCTTTTTTCACTTCCATTAAGCATCACATCA 5751

Pepper ACCTTTGTTTACATTCTACATGTGTTGCTTCTTTTTTCACTTCCATTAAGGATCACATCA 5586

tobaco ACCTTTGTTTACATTCTACATGTGCTACTTCTTTGTTCACTTCCATTAAATATCACATCT 5772

* ***:******** *. ***** *.******* **************. ********:

tomato TCAGCAAGAACAGAAGCTGAATCTCTTCTCAAATGGAAGAGTAATTTACCTACTACTTCT 5828

potato TCAGCAAGAACAGAAGCTGAATCTCTTGTCAAATGGAAGAGAAATTTACCTTCTACTTCT 5835

pepino TCAGCAAGAACAGAAGCTGAATCTCTTGTCAAATGGAAGAACAATTTACCTTCTACTTCT 5835

eggplant TCAGCAAGAAGAGAAGCTGAATCTCTTGTGAAATGGAAGAGTAATTTAGCTTCAACTTCT 5811

Pepper TCAGCAAGAACAGAAGCTGAAGCTCTTGTCAAATGGAAAAGTAACTTACCTTCTACTTCT 5646

tobaco TCAGCAAGAACTGAAGCTGAAGCTCTAATCAAATGGAAGAGTAACTTATCTCCTATTTCT 5832

********** :********* ****: * ********.*. ** *** ** *:* ****

tomato TTCTTAGATTCATGGTCCATTTCGAATCTCGAAAACTTGTGCAATTGGACATCTATTGTG 5888

potato TTCTTGGACACATGGTCCATTTCGAATCTCGAAAACTTGTGCAATTGGACATATATTGTG 5895

pepino TTCTTGGATTCATGGTCCATTTCCAATCTCAAAAACTTGTGTAATTGGACATCTATTGTA 5895

eggplant TTGTTGGATTCATGGTCCATTTCGAATGTCGAAAACTTGTGCAATTGGACATGTATTGTC 5871

Pepper TTCTTGGATTCATGGTCCATTTCCAATCTAAGAAACTTGTGCAATTGGACATCTATTGTG 5706

tobaco TTTTTGGATTCTTGGTCCATTTCCAATCTCAGAAACTTGTGTAATTGGACAGCTATTGTT 5892

** **.** :*:*********** *** *...********* ********* ******

tomato TGTAATGTTGGTGGAACAATTTCAGTGATCAATCTTTCTGATGCAGCCCTCTCAGGCTCA 5948

potato TGTAATGATGGTGGAACAATTTCTGAGATCAATCTTTCTGATGCAGCCCTCTCAGGCACA 5955

pepino TGCAATGCTGGTGGAACAATTTCTGAGATCAATCTCTCTGATGCAGACATCTCAGGCTCA 5955

eggplant TGTAATGCTGGTGGCAGAATTTCTGAGATCAATCTTTCTGATTCAGGCCTCTCAGGCTCT 5931

Pepper TGCAATGCTGGTGGAACCATTTCTGAGATCAATCTTTCTAATGCAGGCCTCTCAGGCTCA 5766

tobaco TGCAATACTGGTGGAACACTTTCTGAGATCAATCTTTCTGATGCAACCCTCTCTGGCTCT 5952

** ***. ******.* ..****:*:********* ***.** **. *.****:***:*:

tomato CTTGATCACCTTGATTTCACTTCATTTCCGAGTCTCGTGAATTTCAATCTTAATCAAAAT 6008

potato CTTGATCACCTTGATTTCACTTCATTTCCGAGTCTCGTGAATTTCAATCTTAATGGAAAT 6015

pepino CTTGATCAGCTTGATTTCACTTCATTTCCAAGTCTTGTGAGTTTCAATCTTAATGGAAAC 6015

eggplant CTTGATCACCTTGACTTCACTTCATTTCCGAGTCTCGTGAGTTTCAATGTCAATGGGAAT 5991

Pepper CTTGATCAGCTTGATTTCACTTCATTTCCGAGTCTCACGAGTTTCAATCTCAATACCAAC 5826

tobaco CTTGATCAGCTTGATTTTACTTCATTCCTGAACCTCACTCGTTTTAATCTCAATGGCAAT 6012

******** ***** ** ******** * .*. ** . ..*** *** * *** **

tomato AACTTCAGTGGATCGATACCTTCGAGTATTGGCAATGCCTCATTGCTCACTTTCTTGGAC 6068

potato AACTTCAGTGGATCGATACCTTCGAGTATTGGCAATGCCTCATTGCTCACATTCTTGGAC 6075

pepino AACTTCAGTGGATCGATACCTTCGAGTATTGGCAATGCCTCATTGCTCACTTTCTTGGAC 6075

eggplant AACTTTAGTGGATCGATACCTTGGAGTATTGGCAATGCATCTAGGCTAACTTTCCTGGAC 6051

Pepper AACTTCAGTGGATCGATACCTTCGAGTATTGGCAATGGGTCTATGCTCACTTTCTTGGAC 5886

tobaco AACTTCAGTGGATCGATACCTTCGAATATTGGCAATGTCTCTATGCTCACTTTCTTGGAC 6072

***** **************** **.*********** **:: ***.**:*** *****

tomato TTAAGTAACAACATCTTGTCAGGTATCATACCTGAAGAGATTGGAAAGTTGAATCAACTT 6128

potato CTGAGTAACAATATCTTATCAGGTGTCATACCTGAAGAGATTGGAAAATTGAATCAACTT 6135

pepino TTGAGTAACAATATCTTGTCAGGAGTCATACCTGAAGAGATTGGGAAGTTGACTCAACTT 6135

eggplant CTGAGTAACAATATCTTGTCAGGTGTCATACCTGAAGAGATGGGGAAGTTGACTCATCTT 6111

Pepper CTGAGTAACAACATCTTGGAAGGTGTCATACCAGAAGAGATTGGGAAGTTAACACGACTT 5946

tobaco CTGAGTAACAATATCTTGGAAGGTGTCATACCAGAAGAGATTGGGAAGTTAACACAACTT 6132

*.******** *****. .***:.*******:******** **.**.**.*.:*.:***

tomato GAGTATCTGAGTTTTTATAACAACAATATCACCGGTGTGATTCCGTATCAGATTAGCAAT 6188

potato GAGTATCTGAGTTTTTATAACAACAATATCGAAGGTGTGATGCCGTATCAGATTAGCAAT 6195

pepino GAATATCTAAGTTTTTATAACAACAATATCGAAGGTGTGATTCCGTATCAGATTAGCAAT 6195

eggplant GAATATATCAGCTTCTATAACAACAATATCGAAGGTGTTATACCGTATCAAATTAGCAAT 6171

Pepper GAATATCTCAGTATGATAAACAACAATATCAATGGTGCTATTCCCTATCAAATTAGCAAC 6006

tobaco GAATATCTCAGTTTTTATAACAACAATTTAAATGGTGTTATTCCCTATCAAATTAGCAAT 6192

**.***.* ** :* :::*********:*... **** ** ** *****.********

tomato CTGCAGAAGCTAATGCACTTGGATGTTGGATCAAATTACTTAGAAACTCCTGATTGGTTG 6248

potato CTGCAGAAGGTAATGCACTTGGATCTTGGATCAAATTTCTTAGAAACTCCTGATTGGTTG 6255

pepino CTGCAGAAGGTATGGCATTTGGATCTTGGATCAAATTTCTTAGAAACTCCTGATTGGTCT 6255

eggplant CTTCAGAAAGTATGGTACTTGGATTTTGGATCAAATTACTTAGAAACTCCTGATTGGTCT 6231

Pepper CTTCAGAAGGTATGGTACTTGGATCTTGGATCAAATTACTTAGAAACTCCTGATTGGTCT 6066

tobaco CTTCAGAAGGTAAGGTACTTGGATCTTGGATCAAATTACTTAGAAACTCCTGATTGGTCT 6252

** *****. **: * * ****** ************:********************

tomato AAGATGAGAAGTATGCCTATGTTGAAGTATCTAAGCTTTGGTTACAATGAACTGAGGTTA 6308

potato AAGATGAGAAATATGCCTGTGTTGACGTATCTAAGCTTTGGTTATAATGAACTGAGGTTA 6315

pepino AAGTTGAGGAATATGCCTATGTTGAAGTATCTAAGCTTTGGTTATAATGAACTGAGGTTA 6315

eggplant AAGTTGAGGAATATGCCTATGCTGAAGCATCTAAGCTTTGGCTATAATGAACTGAGGTCA 6291

Pepper AAAATGAAGAATATGCCTATGTTGGCACATTTAAGCTTTGGTTACAATGAACTGACCTTA 6126

tobaco AAATTGAGGAATATGCCTCTGTTGGCACATCTGAGCTTTGGTTACAATGAATTGAGGTTA 6312

**.:***..*.******* ** **... ** *.******** ** ****** *** * *

tomato GAATTCCCTGAATTCATACTCCGTTGTCATAATCTAACATATCTTGATTTATCGATAAAT 6368

potato GAATTCCCTGAATTCGTACTTCGTTGTCATAATCTAACATATCTTGATTTATCGATAAAT 6375

pepino GAATTCCCTGAATTCGTACTCCGTTGTCATAATCTGACTTATCTTGATTTGTCCATAAAC 6375

eggplant GAATTCCCTGAATTCGTTCTCCGTTGCCATAATTTGACTTATCTTAATTTGTCGATAAAC 6351

Pepper GAATTTCCTGAATTTGTACTCCGTTGCCATAATTTGACTTACCTTGATTTGTCGACAAAC 6186

tobaco GAATTCCCTGAATTTGTACTCCGTTGCCATAATCTGACTTACCTCGATATATCTTTAAAC 6372

***** ******** .*:** ***** ****** *.**:** ** .**:*.** : ***

tomato CATTTCAATGGTTCGATTCCTGAAACAGTATTTACCAACTTGATCAACCTTGAAACACTT 6428

potato CATTTCAATGGTTCGATTCCTGAAACAGTATTTACCAACTTGATCAACCTTGAAAGACTT 6435

pepino CATTTGAATGGTTCGATTCCTGAAACAGTATTTACCAACTTGAACAGACTTGAAACACTT 6435

eggplant CATTTGAATGGTTCAATCCCTGAAACAGTATTCAGTAACTTGAACAACCTTGAAAGACTC 6411

Pepper CATTTTAATGGTTCAATCCCAGAAACAGTATTCACCAACTTAGACAAGCTCGAACGCCTC 6246

tobaco CATTTGAATGGTTCAATCCCAGAAACAGTATTCACCAACTTAGACAAGCTTGAGTACCTT 6432

***** ********.** **:*********** * *****..:**. ** **. .**

tomato AATCTTTCATCTAATTCATTTCAAGGTTCATTGTCACCAAATTTTAACAACTTGTCTAAG 6488

potato AATCTTTCATCTAATTCATTTCAAGGTTCATTGTCACCAAATTTTACTAAGTTGTCTAAG 6495

pepino AATCTTTCATCTAATTCATTTCAAGGTTCATTGTCACCAAATTTTACAAAGTTGTCTAAG 6495

eggplant GATCTTTCATCTAATTTGTTTCAAGGTTCATTGTCACCAAATTTTACCAAGTTGTCCAAG 6471

Pepper AATCTTTCATCTAATTCATTTCAAGGTCCATTGTCACCAAATCTTAGCAAGTTGTCCAAG 6306

tobaco AATCTTTCATCCAATTCATTTGAAGGTTTATTGTCACCAAATTTTACCAAGTTGTCCAAG 6492

.********** **** .*** ***** ************* *** ** ***** ***

tomato TTGAAAGAACTTCAGCTAGGTGGTAACATGTTTTCTGGTTTAATTCCTGATGAAATTGGC 6548

potato TTAAAAGAACTTCAGCTAGGTGTTAACATGTTTTCTGGTTTAATTCCTGATGAAATTGGC 6555

pepino TTAAAAGAACTTCAGCTAGGTGTGAACATGTTTTCTGGCTTAATACCTGATGAAATTGGC 6555

eggplant TTAAAAGAACTTCAGCTAGGTGTTAACATGTTTTCAGGATTAATCCCTGATGAAATTGGC 6531

Pepper ATAAAAGAACTTCAGCTAGGTGTTAACCAGTTTTCCGGCTTAATTCCTGATGAAATTGGC 6366

tobaco TTAAAAGAACTTCGGATTGGTCTTAACATGTTTTCTGGCCTAATTCCTGATGAAATTGGT 6552

:*.**********.*.*:*** ***.:****** ** **** **************

tomato TTGATCACTAGTCTTGAAGTTGTTGTACTTAACAGCAATTCATTCGAAGGAATGATTCCA 6608

potato TTGATTACTAGTCTTGAAGTTCTTGTACTTTTCAACAATTCATTTGAAGGAAAGATTCCA 6615

pepino TTGATCACTAGTCTTGAAGTTCTTGTACTTTTCAACAATTCATTTGAAGGAAAGATTCCA 6615

eggplant TTGATCACTAGTCTTGAAATTCTTGTACTTTTCAACAATTCATTTCAAGGAAAGATTCCA 6591

Pepper TTGATCACTAGTCTTGAAGTTCTTGTACTTTTCAACAATTCAATTCAAGGAAACATTCCA 6426

tobaco TTGATCACTAGTCTTGAAGTTGTTGTACTTTTCAACAATTCATTTCTAGGAAATATTCCA 6612

***** ************.** ********::**.*******:* :*****: ******

tomato TCTTCTATAGGTAGACTCATAAATCTTCAAAGGTTGGATCTTCGAACGAACAGTTTGAAT 6668

potato TCTTCTATAGGTAGACTCATAAATCTTCAAAAGTTGGATCTTCGAAAGAACGATTTGAAT 6675

pepino TCTTCTATAGGTAGACTCATAAATCTTCACCAGTTGGATCTTCGAAAGAACGATTTGAAT 6675

eggplant TCTTCTATAGGTAGATTGATAAATCTTCAACAATTGGATCTTCGGAAGAACGATTTGAAT 6651

Pepper TCTTCTATAGGCAGACTTAGAAATCTTCAACACCTGGATCTTCGAAAGAACGGTTTCAAT 6486

tobaco TCTTCTATAGGAAGACTCACAAATCTTCAACAACTAGACCTTCGAAAGAATCGTTTGAAT 6672

*********** *** * * *********... *.** *****.*.*** .*** ***

tomato TCAACTATTCCTTCTGAGCTTGGCTTTTGTACTAAACTCAATTACTTAGCTCTAGCTGAA 6728

potato TCAACTATTCCTTCTGAGCTTGGCTTTTGTACTAAACTCACTCTCTTAGCTCTAGCAGAA 6735

pepino TCAACTATTCCTTCTGAGCTTGGCCTTTGTACTAAACTCACTTACTTGGCTCTAGCAGAG 6735

eggplant TCAACCATTCCTTCTGAGCTTGGCCTTTGTACTAAACTAACTATCTTGGCTCTAGCAGAA 6711

Pepper TCAACCATTCCTTCTGAACTTGGCCTTTGTACTAAACTCGCTGTCTTGGCTCTAGCAGAA 6546

tobaco TCAACCATTCCTTCTGAGCTTGGCCTGTGTACTAACCTCACCGTCTTGGCTTTAGCAGAG 6732

***** ***********.****** * ********.**... :***.*** ****:**.

tomato AATGACCTGCAAGGGTCATTGCCTTTATCATTCTCCTCTCTTACAAAGTTATCTGAATTG 6788

potato AATGACCTGCAAGGGTCATTGCCTTTATCATTCTCCTCTCTTGCAAAGTTATCTGATTTG 6795

pepino AACTCCCTGCAAGGACCATTGCCTCCATCATTCTCCTCTCTTACCAAGTTATCTGATTTG 6795

eggplant AATGCCCTGCAAGGACCAATGCCTCCATCATTCTCTTCTCTTACCAAGTTATCTAATTTG 6771

Pepper AATTCCCTGCAAGGACCATTGCCTCCATCCTTCTCTTCTCTTACCAAGTTATCGGAATTG 6606

tobaco AATTTCTTGCAAGGACCATTGCCTCCATCTTTCTCTTCTCTGACCAAGTTATCTGATTTG 6792

** * *******. **:***** *** ***** ***** .*.******** .*:***

tomato GGGTTGTCTGATAATAGTCTTTCTGGTGAAATCTCATCGAATTTAATCACCAATTGGACT 6848

potato GGGTTGTCTGATAATAGTCTTTCTGGTGAAATCTCATCGAATTTCATCACCAATTGGACT 6855

pepino GGGTTGTCTGATAATAGTCTTTCTGGTGAAATATCATCAAATCTCATCACTAATTGGACT 6855

eggplant GGGTTGTCCGATAATTTTCTGTCCGGTGAAATCTCATCAAATTTCATCACTAATTGGACA 6831

Pepper GGGTTGTCCGATAACATTCTTTCTGGTGAAATCTCATCAAATTTCATCACAAATTGGACT 6666

tobaco GGGTTGTCTTCTAATATTCTTTCTGGTGAGATCTCAACATATTTCATCACCAATTGGACT 6852

******** .*** : *** ** *****.**.***:*.:** *.***** ********:

tomato GAGTTGACATCTCTGCAGCTTCAAAACAATTCCTTTACGGGGAAAATTCCACCCGAAACC 6908

potato GAGTTGACATCTTTGCAGCTTCAAAACAATATGTTTACAGGGAAAATCCCACCCGAAACC 6915

pepino GAGTTGACATCTTTGCAGCTTCAAAACAATATCTTTACAGGGAAAATTCCACACGAAATC 6915

eggplant GAACTGATATCTTTGCAGCTTCAAAACAATTCCTTTACGGGGAAAATTCCATTGGAAACC 6891

Pepper GAATTGGCATCTTTGCAGCTTCAAAACAATTCTTTTACTGGGAAAATTCCACCTGAAACC 6726

tobaco GAGCTGACATCTTTGCAGCTTCAAAACAATTCCTTTACTGGGAATATTCCATCTGAAATC 6912

**. **. **** *****************: ***** *****:** *** **** *

tomato ACTCAATTGACAAACCTTGAATATCTTTACCTCTATCATAACAAGTTCACTGGTTCCATT 6968

potato AGTCAATTGACAAACCTTGTATATCTTTACCTCTATCATAACAATTTCACTGGTTCCATT 6975

pepino AGTCAGTTGACAAACCTTGCATATCTTTACCTCTTTCATAACAATTTCACTGGTTCCATT 6975

eggplant AGTCAGTTGACAAACATCACTTATCTTTACCTCTTTCATAACAATTTCACTGGTTCCATT 6951

Pepper AGTCAGTTGACAAACCTCAACTTTCTTTACCTCTTTCATAACAATTTCACTGGTTCCATT 6786

tobaco AGTCAGTTGAAAAGCCTCAAATATCTTTTCCTCTTTCATAACAATTTCACTGGTCCCATT 6972

* ***.****.**.*.* . *:*****:*****:********* ********* *****

tomato CCCTATCTGATTGGGAACTTGCAAAACTTGTTAGAGTTAGATTTGTCTGATAACCAGCTT 7028

potato CCCTATCAGATTGGGAACTTGCAAAACTTGTTGGATTTAGACTTTTCTGATAACCAGCTT 7035

pepino CCCTATCAGATTGGGAACTTGCAAAACTTGCTGGAGCTAGATTTTTCTGATAACCAGCTT 7035

eggplant CCCTATCAGATAGGGAACTTGGTAAACTTGTTGGAGCTAGATTTCTCTGATAACCAGCTT 7011

Pepper CCCTCTCAGATTGGTAACTTACAAAACTTGTTGGAGCTAGATTTCTCTGACAACCAGCTT 6846

tobaco CCCTCTGAGATTGGAGACTTGCAAAACTTGTTGGAGCTAGATTTCTCTGACAACCAGCTT 7032

****.* :***:** .****. :******* *.** **** ** ***** *********

tomato TCAGGCATAATACCTCCAACCATTGGAAATCTGACCAATCTAAAGACGTTGCACCTTTTC 7088

potato TCAGGCATAATACCTCCAACCATTGGAAATCTGACCAATCTAAAGATGTTGCAACTTTTT 7095

pepino TCAGGCACAATACCTCCAACCATTGGAAATCTGACCAATCTAAAGACGTTGCAACTTTTT 7095

eggplant TCAGGCACAATACCTCCAACCATTGGAAATCTGACCAATCTAAAGACGTTGCAACTTTTT 7071

Pepper TCAGGCACAATACCTCCAACCATTGGAAATCTGACCAATCTAACGACGTTGCATCTTTTT 6906

tobaco TCAGGAACAATACCTCCATCCATTGGAAACTTAACCAATCTAACAACGTTGCATCTTTTT 7092

*****.* **********:********** *.**********..* ****** *****

tomato CGCAACAATCTCAGTGGAACCATTCCTCCTGAGATTGGAAAATTGATATTCCTTGAAAGC 7148

potato CGCAACAATCTCAGTGGAACCATTCCTCCTGAGATTGGAAAATTGATATCCCTTGAAACC 7155

pepino CGCAACAATCTCAGTGGAACCATTCCTTCTGAGATTGGAAAATTGATATCCCTTCAAACC 7155

eggplant CGCAACAATCTCAGTGGAACCATTCCTTCTGAGATTGGAAAATTGACATCCCTTCAAACT 7131

Pepper CGCAACAATCTTAGTGGAACCATTCCTCCTGAGATTGGGAAATTGATATCCCTTCAGACC 6966

tobaco CGCAATGTTCTCAGTGGAACCATTCCTCCTGAGATTGGGAAATTGACATCTCTTCAGATC 7152

***** .:*** *************** **********.******* ** *** *.*

tomato ATCGACATCAACACCAACCGACTAAGTGGTGAGCTGCCGGACAGCATTTCTGACCTCAGT 7208

potato ATCGACATCAATACCAACCGACTAAGTGGTGAGCTGCCGGACAGCATTTCTGACCTCAGT 7215

pepino ATTGACATCAACACCAACCAACTAAGTGGTGAGCTGCCGGTCAGCATTTCTGACCTCAGT 7215

eggplant CTCGACATCAACACCAACCGACTAAGTGGTGAGCTGCCGGACAGCATTTCTGACCTCAGT 7191

Pepper CTCGACATCAACACCAACCGACTAAGTGGTGAGCTGCCGGATAGCATTTCTGACCTCAGT 7026

tobaco CTTGATATCAATACCAACCGACTTAGTGGTGAGCTGCCGGACATCATTTCTGACCTCAGC 7212

.* ** ***** *******.***:****************: * ***************

tomato GCACTGACGATTATTTCTGTATATACTAATGATTTCTCAGGCAGTGTGCCTAAGGACTTT 7268

potato GAGCTGAAGTTTCTTTCTGTATATACTAATGATTTCTCAGGCAGTGTCCCTAAGGACTTT 7275

pepino GATCTGAGGCTTCTTTCTGTGTATACTAATAATTTCTCAGGCAGTGTCCCCAAGGACTTT 7275

eggplant AGTCTAAACCTTCTTTCTGTATATACTAATAATTTCTCAGGCAGTGTCCCTAAGGACTTT 7251

Pepper GAGCTGAAGCTTATTTCTTTATATACTAATAATTTCTCAGGCAGCGTCCCTAAGGACTTT 7086

tobaco AGTCTGAACCTTTTTTTTGTATATAGTAATAATATCTCGGGCAGTGTTCCTGAGGATTTT 7272

. **.* ** *** * *.**** ****.**:****.***** ** ** .**** ***

tomato GGAAAGAATAGTCCTCCATTGTCAAGTGTCAGCTTTGCAAATAATAGTTTCACTGGTGAA 7328

potato GGAAAGAACAGTCCTCAATTGTCAAGTGCCAGCTTTGCAAATAACAGCTTCACTGGTGAA 7335

pepino GGAAAGAACAGTCCTCTATTGTCAAGTGTCAGTTTTGCAAATAATAGTTTCACTGGTGAA 7335

eggplant GGAAAGAACAGTCCTCTATTGTCTAATGTTGGCTTGGCAAATAACAGCTTCACTGGTGAA 7311

Pepper GGAAAGAACAGTCCTCTATTGTCAAGTGCCAGCTTTGCAAACAACACCTTCACTGGCGAA 7146

tobaco GGAAAAAAAAGTCCTCAATTGTCCAGTGTCAGCTTTTCAAATAACAGCTTCAGTGGTGAA 7332

*****.** ******* ****** *.** .* ** **** ** * **** *** ***

tomato CTACCTGCTGGATTGTGTAGCCCAAAT---CTTAAGGAGTTGACGATAAACGGGAACAAA 7385

potato CTACCTGCTGGATTGTGTAGCCCGAAT---CTTGAGGAGTTGACGATAAATGGGAACAAA 7392

pepino TTACCTGCTGGATTGTGTAGCCAGAAT---CTTGAGGAGTTGACGATAAATGGGAACAAG 7392

eggplant CTACCTCCTGGATTATGTAGCCGAAAT---CTTGAGGAGTTGACAATAAATGGGAACAAA 7368

Pepper CTACCTCCTGGATTATGTAACCAGAAT---CTTGAGGAGTTGACAATAAATGGGAACAAA 7203

tobaco CTGCCTCCTGGATTATGTAGCCAGTTTGCTCTTGAGGAGTTGACAATAAATGGCAACAAA 7392

*.*** *******.****.** .::* ***.**********.***** ** *****.

tomato TTCAGTGGGAAGTTACCAGATTGTTTGAAGAACTGCACGCTGCTAACCCGAGTACGGCTT 7445

potato TTCAGTGGGAAGTTACCAGATTGCTTGAAGAACTGCACACTTCTAAGACGAGTACGGCTT 7452

pepino TTCAGTGGGAAATTACCAGATTGCTTGAAGAACTGCACACTGCTAAGACGAGTACGGCTT 7452

eggplant TTCAGTGGGAAGTTACCAGATTGCTTGAAGAACTGCACAAAGCTAAGACGAGTACGGCTT 7428

Pepper TTCAGTGGGAAGTTACCAGATTGCTTGAAGAACTGCACAGGACTAAAGCGAGTACGGCTT 7263

tobaco TTCAGTGGAAAGTTACCAGATTGCTTGAAGAATTGCACAGAGCTAAAAAGAATAAGGCTT 7452

********.**.*********** ******** *****. **** .**.**.*****

tomato GAAGGCAACAATTTATCTGGTAATCTTGCAGATGCATTCGGAGTGCACCCGAATCTTGTT 7505

potato GAAGGCAACAATTTATCTGGTAATCTTGCAGATGCATTTGGAGTGCACCCGAAACTTGTT 7512

pepino GAAGGCAACAATTTATCTGGTAATCTTGCAGAGGCATTTGGAGTGCACCCGAATCTTGTT 7512

eggplant GAAGGCAACAATTTATCTGGTAATCTTGCGGAGGCATTTGGAGTGCACCCGAATCTTGTT 7488

Pepper GAAGGCAACAATTTTTCTGGTAATCTTGCAGAGGCATTTGGAGTGCACCAGAATCTTGTT 7323

tobaco GAAGGCAACAACTTATCTGGTAATCTTGCAGAGGCATTTGGCGTGCACCCGAATCTTGAT 7512

*********** **:**************.** ***** **.*******.***:****:*

tomato TTCCTTTCTCTTAGTGACAACCAACTTTCAGGTGAACTCTCACCTAACTGGGGGAAATGT 7565

potato TTCCTTTCTCTTAGTGACAACCAACTTTCAGGTGAACTCTCACCTGACTGGGGGAAATGT 7572

pepino TTCCTTTCTCTTAGTGACAACCAACTTTCAGGTGAACTCTCACCTGACTGGGGGAAGTGT 7572

eggplant TTCCTTTCTCTTAGTGACAACCAGTTTTCAGGTGAACTCTCACCTGACTGGGGGAAATGT 7548

Pepper TTCCTTTCTCTTAGTGACAACCAACTTTCAGGTGAACTGTCACCTGACTGGGGGAAATGT 7383

tobaco TTCCTTTCTCTCATTGACAACCAATTTTCAGGTGAACTCTCACCTGAATGGGGGAAATGT 7572

*********** * *********. ************* ******.*.********.***

tomato GATAGTCTCACTAATCTAAGAATGGATGGAAACAAATTTTCTGGTGTAATCCCAGCTGAG 7625

potato GAAAATCTCACAAGTCTAAGAATGGATGGAAACAAATTTTCTGGTGTAATCCCAAGTGAG 7632

pepino GAAAATCTCACAAGTCTAAGAATGGATGGAAACAAATTTTCTGGTGTAATCCCAGCTGAG 7632

eggplant GAAAATCTCACAAGTCTAAGAATGGATGGAAACAAAATTTCTGGTGTAATTCCAGCTGAG 7608

Pepper GAAAAACTCACAAGTCTAAGAATGCATGGAAACAAATTTTCTGGTGTAATCCCAGCTGAG 7443

tobaco GAAAAACTCACAAGTCTAAGAATGGATGGAAACAAAATTTCTGGTGTGATCCCAGCTGAG 7632

**:*.:*****:*.********** ***********:**********.** ***. ****

tomato CTAGGGAACCTGAGAGCGTTGCGCATGTTAGCTTTGGAAGGAAATGAATTGACTGGTGAG 7685

potato CTAGGGAACCTGAGAGCGTTGCGCGTGTTAGCTTTGGAAGGAAATGAATTGACTGGTGAA 7692

pepino CTAGGGAACCTGAGAGCGTTGCGCATGTTAACTTTGGAAGGAAATGAATTGACTGGTGAA 7692

eggplant CTAGGGAATCTGAGCGAGCTGCGCATGTTAACTTTGGAAGGAAATGAATTAACTGGTGAA 7668

Pepper CTAGGGAATCTGAGAGAGCTGCGCATGTTAACTTTGGAAAGAAATGAATTGACTGGTGAA 7503

tobaco CTAGGGAATCTGAGGGAGCTGCGCGTGTTAACTTTGGAAGGAAATGAATTGACCGGTGAA 7692

******** ***** *.* *****.*****.********.**********.** *****.

tomato ATTCCTTCTGAACTGGGAAGGTTAGACCTGCTCTTCAATCTCAGCTTGAGCAAAAACAAT 7745

potato ATTCCCTCTGAACTGGGAAGGTTAGACCTGCTCTATAATCTCAGCTTGAGTAAAAACAAT 7752

pepino ATTCCTTCTGAACTGGGAAGGTTAGACCTGCTCTACAATCTCAGCTTGAGCAAAAACAAT 7752

eggplant ATTCCTTCCAAATTGGGAAGGTTAGGCCTGCTCTACAATCTTAGCTTGAGCAGAAACAAT 7728

Pepper ATTCCTTCTGAACTAGGAAGGTTAGGCCTGCTCTTCAATCTCAGCTTGAGTGAAAACAAT 7563

tobaco ATTCCTTCTGAACTGGCAAAGTTAGGCCAGCTCTACAATCTCAGCTTGAGCAAAAACAAT 7752

***** ** .** *.* **.*****.**:*****: ***** ******** ..*******

tomato CTTACTGGAGGCATCCCTCAGTCTATTGGCAATTTAACTAATCTCCAGTATCTTGATTTG 7805

potato CTTACTGGAGGCATCCCTCAGTCTGTTGGCAATTTAACTAAGCTCCAGTATCTTGATTTG 7812

pepino CTTACAGGAGGCATCCCTCAGTCTGTTGGCAATTTAACTAAGCTCCAGTATCTTGATTTG 7812

eggplant CTTACAGGAAGCATCCCTCAGTCTATTGGCAATTTAACTAATCTCCAGCATCTTGATTTG 7788

Pepper CTCACAGGAGGCATCCCTCAGTCAGTTGGCAATTTAACTAAGCTCCAGCATCTTGATTTG 7623

tobaco CTTACAGGAGGCATCCCTCAGTCTGTTGGAAATTTAACTAAGCTCCAGTATCTTGACTTG 7812

** **:***.*************:.****.*********** ****** ******* ***

tomato TCAACAAACGAGTTAAGTGGTAACATACCAGTAGATCTTGGGAAGTGTGATCGTCTGTTG 7865

potato TCGACAAACAAGTTAAGTGGTAACACACCAGTAGATCTTGGGAAGTGTGAGAGCCTGTTG 7872

pepino TCAACAAACAAGTTAAGTGGTAACATACCAGCAGATCTTGGGAAGTGTGAGAGCCTGTTG 7872

eggplant TCAGCAAACAAGTTAAGTGGTAACATACCAGTAGATCTTGGGAAGTGCGAGCGCCTGTTG 7848

Pepper TCGACAAACAAGTTAAGTGGTAACATACCAGTAGAACTTGGGAAGTGTGACAGCCTGTTG 7683

tobaco TCAACAAACAAGTTAAGTGGTAACATACCGGTAGATGTTGGGAAGTGCGAGAGACTTTTG 7872

**..*****.*************** ***.* ***: ********** ** .* ** ***

tomato AGCTTGAATCTTGGCAACAACTCATTATCAGGTGGCATTCCATCTGACCTTGGCAATTTG 7925

potato AGCTTGAATCTTGGCAACAACTCATTATCAGGTGGCATTCCATCTGACCTTGGTAATTTG 7932

pepino AGCTTGAATCTTGGAAACAACTCATTATCAGGTGGCATTCCATCTGACCTTGGCAATTTG 7932

eggplant AGCTTGAATCTTGGTAACAACTCATTATCAGGTGGCATTCCCTCTGAACTTGGCAATTTG 7908

Pepper AGCTTGAATCTTGGCAACAACTTATTATCAGGTGGTATTCCCTCTGAACTTGGCAATTTG 7743

tobaco AGCTTGAATCTTGGCAACAACTCATTATCAGGTGGTATTCCCTCTGAACTTGGAAATTTG 7932

************** ******* ************ *****.*****.***** ******

tomato ATGCAGTTGAGTATTCTTTTGGACCTAAGTAACAATTCGCTAACGGGAACCATTCCACAA 7985

potato ATGCAGTTGAGTATTCTTTTGGACTTGAGTGGCAATTCACTAACGGGAACCATTCCACAA 7992

pepino ATGCAGTTGAGTATTCTTTTGGACTTAAGTGGCAATTCACTAACGGGAACTATTCCACAA 7992

eggplant ATGCAGTTGAGTATTCTTTTGGACCTAAGTGGCAATTCACTAACGGGAACCATTCCACAA 7968

Pepper ATGCAGTTGAGTATTCTTTTGGACATAAGTGGCAATTCACTAACGGGAACCATTCCACAA 7803

tobaco ATGGGGTTGAGTATTCTTTTGGACCTCAGTGGCAATTCGCTATCGGGAACAATCCCACAA 7992

*** .******************* * ***..******.***:******* ** ******

tomato AACTTGGCCAAACTCACCTCATTAATGCATCTCAACCTCTCACATAACAACCTCTCAGGT 8045

potato AACTTGGCCAAACTCACCTCATTAATGCATCTCAACCTCTCACATAACAACCTCTCAGGT 8052

pepino AACTTGGCCAAGCTCACCTCATTAATGCATCTCAACCTCTCCCATAACAACCTCTCAGGT 8052

eggplant AACTTGGCCAAGCTCACCTCATTAATGAATCTCAACCTCTCACACAACAACCTCTCGGGT 8028

Pepper AACCTGGCTAAGCTCACCTCATTAATGCATCTCAACCTCTCACATAACAACTTCTCAGGT 7863

tobaco AACTTGGCCAAGCTTACCTCATTGGAGGATCTCAACCTCTCACATAACAACCTCTCAGGT 8052

*** **** **.** ********..:* *************.** ****** ****.***

tomato AGAATTCCTCCAGCATTATCTCAGATGATCAGTCTTCAGGAGATGGATTTTTCCTACAAT 8105

potato AGAATTCCTCCAGCATTATCTCAGATGATCAGTCTTCAGGAGATGGATTTTTCCTACAAT 8112

pepino AGAATTCCTCCAACATTATCTCAGATGATCAGTCTTCAGGAGATGGATTTTTCCTACAAT 8112

eggplant AGAATTCCTCCAGCGTTATCTCGCATGATCAGTCTTCAAGAGATGGACTTTTCGTACAAT 8088

Pepper AGAATTCCTCCAGCATTATCTCGCATGATCAGTCTTCAGGAGATGGATTTTTCATACAAC 7923

tobaco AGAATTCCTCCGTCATTATCTCGCATGGTCAGTCTTCAGGAAATGGACTTTTCCTACAAC 8112

***********. *.*******. ***.**********.**.***** ***** *****

tomato GAGTTTTCTGGACCAATCCCAACCGATGGAGTATTTCAAAGAGCACCCGCTAGATCTTTT 8165

potato GAGTTTTCTGGACCAATCCCAACCGATGGAGTATTTCAAAGAGCACCCGCTAGATCTTTT 8172

pepino GAGTTTTCTGGACCAATTCCAACCGATGGAATATTTCAAGGAGCAACTGCTAGATCATTT 8172

eggplant GAGTTTTCTGGGCCAATTCCAACTGATGGAATATTTCGAGGAGCACTGGCTAGATCTTTT 8148

Pepper AAGTTTTCTGGACCAATTCCAACCGATGGAGTATTTCAAGGAGCAACCGCTAGATCTTTT 7983

tobaco GAGTTTTCAGGACCAATTCCAACCGATGGAGTATTTCAAGGAGCGGCTGCTAGATCTTTT 8172

.*******:**.***** ***** ******.******.*.****. ********:***

tomato CTTGGGAACTCTGGTTTGTGTGGAAATATAGAAGGATTATCCTCATGTAATTTGGATACT 8225

potato CTTGGAAACTCTGGTTTGTGTGGAAATGTAGAGGGATTATCCTCATGTAATTTGGATACT 8232

pepino CTTGGAAACTCTGGTTTGTGTGGAAATGTTGAAGGATTATCCTCATGTAATTTGGATAGC 8232

eggplant CTTGGAAACTCTGGTTTGTGTGGAAATGTAGAAGGATTATCCTCATGTAATTCGGATACG 8208

Pepper CTTGGGAACTCTGGTTTGTGTGGAAATGTAGAAGGATTATCCTCATGTAATTTGGATACC 8043

tobaco CTTGGAAACTCTGGTTTGTGTGGAAATGTAGAAGGATTATCCTCGTGTAATTTGGCTACC 8232

*****.*********************.*:**.***********.******* **.**

tomato CCCAATGACAAGTCCAGAAATAATAATCAAAAGATCCTCATTGCGGTACTTGTACCGGTG 8285

potato CCCAATGACAAGTCCAGAAATAATAATCAAAAGATCCTCATTGGGGTACTTGTACCGGTG 8292

pepino CCAAATGACAAGTCCAGAAATAATAATCAAAAGATTCTCATTGGGGTACTTGTACCGGTG 8292

eggplant CCAAATGACAAGTCTGGAAACAAGAAGCAAAAGGTCCTCATTGCGGTACTTGTACCTGTC 8268

Pepper CCGAATGAGAAGTCTAAAAATAAGAATCAAAAGGTCCTCATTGGGGTACTTGTACCCGTG 8103

tobaco CCGGATGAGAAGTCCAGAAACAAGAATCAAAAGGTTCTTATTGGGGTACTTGTACCGGTG 8292

** .**** ***** ..*** ** ** ******.* ** **** ************ **

tomato GTCAGCCTCATACTTTTAGCAATCCTGTTTGTTGCATGTCTCGTGTCTCGAAGGAAGGCT 8345

potato GTCAGCCTCATACTTTTGGCAATCCTGTTTGTTGCATGTCTCGTGTCTCGAAGGAAGGCT 8352

pepino GTCAGCCTCATACTTCTGGCAATCCTGTTTGTTGCATGTCTCGTGTCTCGGAGGAAGGCT 8352

eggplant GGCAGCCTCATACTTTTGGCAATCCTGTTTGTTGCATGTCTTGTGTCTCGAAGGAAGGCT 8328

Pepper GCCAGCCTCATACTTTTGGCAATCCTGTTTGTTGCATGTCTTGTGTCTCGAAGGAAGGCT 8163

tobaco GCCTGCCTCATACTTTTGGCAATCATTTTTGTTGCATGCCTTGTATCTCGAAGGAAGGCT 8352

* *:*********** *.******.* *********** ** **.*****.*********

tomato AAGCAATATGATGAGGAGATCAAAGCTAGCCAGGTACATGAAAATACAGAATCTCTGATT 8405

potato AAGCAATATGATGAGGAGATCAAAGCTAGCCAGATACATGAAAATACAGAATCTCTGATT 8412

pepino AAGCAATATGATGAGGAGATCAAAGCTAGCCAGATGCATGAAAATTCAGAATCTCTGATT 8412

eggplant AAGCAATATGATTTGGAGATCAAAGCTAGCCAGATGCATGAAAATTCAGAATCTCTGATT 8388

Pepper AAGCAATATGATGAGGAGATCAAAGCTAGCCAGATGGATGAAAATTCGGAATCTCTGATT 8223

tobaco AAGCAATACGATGAGGAGATCAAAGCCAGCCAGATGTATGAGAATTCAGAGTCTCTCATT 8412

******** *** :************ ******.*. ****.***:*.**.***** ***

tomato TGGGAAAGAGAAGGGAAGTTTACATTTGGTGACATAGTGAAAGCTACTGAAGATTTCAGT 8465

potato TGGGAAAGAGAAGGGAAGTTTACATTTGGTGACATAGTGAAAGCTACTGAAGATTTCAGT 8472

pepino TGGGAAAGAGAAGGCAAGTTCACATTTGGCGACATAGTGAAAGCTACTGAAGATTTCAGT 8472

eggplant TGG------GATGCTTTGTTTACATTTGGTGACATAGTGAAAGCTACTGAGGATTTCAGT 8442

Pepper TGGGAAAGAGAAGGGAAGTTCACATTTGGTGACATAGTGAAAGCTACTGAAGAATTCAGT 8283

tobaco TGGGAAAGAGAAGGGAAGTTTACATTTGGTGACATTGTGAAAGCTACTGAAGATTTCAAT 8472

*** **:* ::*** ******** *****:**************.**:****.*

tomato GAAAAGAACTGCATTGGAAGAGGAGGCTTTGGAACTGTCTATAAAGCTGTTTTGCCCTCT 8525

potato GAAAAGAACTGCATTGGAAGAGGAGGCTTTGGAAGTGTCTATAAAGCTGTTTTGCCCTCT 8532

pepino GAAAAGAACTGCATTGGAAGAGGAGGCTTTGGAAGTGTCTATAAAGCTGTTTTGCCCTCT 8532

eggplant GAAAAGAACTGCATTGGAAGAGGAGGCTTTGGAAGTGTTTATAAAGCTGTTTTGCCCTCT 8502

Pepper GAAAAGAACTGCATTGGAAGAGGAGGCTTTGGAAGTGTCTATAAAGCTGTTTTACCCTCT 8343

tobaco GAGAAAAACTGCATTGGAAGAGGAGGCTTTGGAAGTGTTTATAGAGCAATTTTGCCATCT 8532

**.**.**************************** *** ****.***:.****.**.***

tomato GGGCAGATTGTTGCAGTCAAAAGACTCCACATGTCAGACTCAAGTGACATTCCATTGACT 8585

potato GGGCAGATTGTTGCAGTCAAAAGACTCAACATGTCAGACTCAAGTGATATTCCATTGACT 8592

pepino GGACAGATTGTTGCAGTCAAAAGGCTCAACATGTCAGACTCGAGTGATATTCCATTGACA 8592

eggplant GGACAGATTGTTGCAGTCAAAAGACTCAACATGTCAGACTCGAGTGACATTCCATTGACA 8562

Pepper GGGCAGATAGTTGCAGTCAAAAGACTCAACATGTCAGACTCGAGTGACATTCCATTGACA 8403

tobaco GGGCAGGTTGTTGCAGTCAAAAGACTCAACATGTCAGACTCAAGTGACATTCCTTTAACA 8592

**.***.*:**************.***.*************.***** *****:**.**:

tomato AATCGTCGAAGCTTTGAGAATGAGATTAGAACTTTGACAGAGGTGAGACACAGGAACATA 8645

potato AATCGTCGAAGCTTTGAGAATGAGATTAGAACTTTGACAGAGGTGAGACACAGGAATATA 8652

pepino AATCGTCGAAGCTTTGAGAATGAGATTAGAACTTTGACAGAGGTGAGACACAGGAATATA 8652

eggplant AATCGTCGAAGCTTTGAGAATGAGATCAGAACTCTGACAGAGGTGAGACACAGGAATATA 8622

Pepper AATCGTCGAACCTTTGAGAATGAGATTAAAACGTTGACAGAGGTAAGACACAGGAATATA 8463

tobaco AGTCGTCGAAGCTTTGAGAATGAGATTAGAACTTTGACAGAGGTGAGACACAGGAATATA 8652

*.******** *************** *.*** **********.*********** ***

tomato ATTAAGCTCTTTGGTTACTGTTCTAAGAATGGGTGCATGTACTTGGTTTATGAGTATATA 8705

potato ATTAAGCTCTTTGGTTACTGTTCCAAGAATGGATGCATGTACTTGGTTTATGAGTACATA 8712

pepino ATTAAGCTCTTTGGTTACTGTTCCAAGAATGGGTGCATGTACTTGGTTTATGAGTATATA 8712

eggplant ATTAAGCTCTTTGGTTACTGTTCCAAGAATGGGTGCATGTACTTGGTTTATGAGTATATA 8682

Pepper ATTAAGCTCTTTGGTTACTGTTCCAAGAATGGGTGCATGTACTTGGTTTATGAGTATATA 8523

tobaco ATCAAGCTCTTTGGTTACTGTTCCAAGAATGGGTGCATGTACTTGGTTTATGAGTATATA 8712

** ******************** ********.*********************** ***

tomato GAAAGAGGTAGCCTGGGGAAAGTTCTATATGACAATGATATGGGAATGGAACTTGGATGG 8765

potato GAAAGAGGTAGCCTGGGGAAAGTTCTATATGACAATGAGATGGGAATGGAACTTGGATGG 8772

pepino GAAAGAGGTAGCCTGGGGAAAGTTCTATATGACAATGAAATGGGAATGGAACTTGGATGG 8772

eggplant GAAAGAGGTAGCCTAGGGAAAGTTCTATATGACAACGATATGGTAATGGAACTTGGATGG 8742

Pepper GAAAGAGGTAGCCTGGGTAAAGTTCTATATGACAAGGAGATGGGACTGGAACTTGGATGG 8583

tobaco GAAAAAGGTAGCCTCGGGAAAGTTCTGTATGACAGTGAGATGGTAACGGAACTGGGGTGG 8772

****.********* ** ********.*******. ** **** *. ****** **.***

tomato GGTACAAGAGTGAAAATTGTGCAAGGAATAGCTCATGCACTCGCTTACTTGCATCATGAC 8825

potato GGTACAAGAGTGAAAATTGTGCAAGGAATAGCTCATGCACTCGCTTACTTGCATCATGAC 8832

pepino GGTACAAGAGTGAAAATTGTGCAAGGAATAGCTCATGCACTCGCTTACTTGCACCATGAC 8832

eggplant GGTACAAGAGTGAAAATTGTTCAAGGAATAGCTCATGCACTCGCGTACTTGCACCATGAC 8802

Pepper GATACAAGAGTGAAAATTGTGCAAGGAATAGCTCATGCACTTGCTTACTTGCACCATGAC 8643

tobaco GGCACAAGAGTGAAAATTGTGCAAGGAATAGCACATGCACTTGCTTACTTGCACCATGAC 8832

*. ***************** ***********:******** ** ******** ******

tomato TGCTCTCCGCCCATCGTTCACCGTGATGTATCATTGAATAACATTTTGCTTGAATCAGAA 8885

potato TGCTCTCCGCCCATTGTTCACCGTGATGTATCATTGAATAACATTTTGCTTGAATCAGAA 8892

pepino TGCTCTCCGCCCATTGTTCACCGTGATGTATCACTGAATAACATTTTGCTTGAGTCAGAA 8892

eggplant TGCTCTCCACCCGTTGTGCACCGTGATGTATCATTGAACAACATTTTGCTCGAGTCAGAA 8862

Pepper TGCTCTCCACCCATTGTGCACCGTGATGTATCACTGAATAACATTTTGCTTGAGTCAGAA 8703

tobaco TGCTCCCCACCCATCGTGCACCGTGATGTATCGCTGAATAACATCTTGCTTGAGTCAGAG 8892

***** **.***.* ** **************. **** ***** ***** **.*****.

tomato TTCGGGCCACGACTCTCTGACTTTGGCACAGCAAAGTTGTTAGCTTCCGACTCATCAAAT 8945

potato TTCGAGCCACGACTCTCTGACTTTGGCACAGCAAAGTTGTTAGCTTCAGACTCATCAAAT 8952

pepino TTCGAGCCACGACTCTCTGACTTTGGCACAGCAAAGTTGTTAGCTTCAGATTCATCAAAT 8952

eggplant TTCGAGCCACGGCTGTCTGACTTTGGCACAGCAAAGTTGTTAGCTTCAGAATCATCAAAT 8922

Pepper TTTGAGCCACGACTCTCTGACTTTGGCACAGCGAAGCTGTTAGCTTCAGATTCATCAAAT 8763

tobaco TTCGAGCCACGACTCTCTGACTTTGGCACAGCAAAGCTGCTAGCTTCAGAATCATCAAAT 8952

** *.******.** *****************.*** ** *******.** *********

tomato TGGACCACAGTTGCTGGTTCTTATGGCTATATGGCACCAGAGCTTGCACTTACCATGCGT 9005

potato TGGACCACAGTTGCTGGTTCTTATGGCTATATGGCACCAGAGCTTGCACTTACCATGCGT 9012

pepino TGGACCACAGTTGCAGGTTCTTATGGATATATGGCACCAGAGCTTGCACTTACCATGCGT 9012

eggplant TGGACCACAGTTGCTGGTTCTTATGGCTATATGGCACCAGAGCTTGCACTTACCATGCGT 8982

Pepper TGGACCACAGCTGCAGGTTCTTATGGCTATATGGCACCAGAGCTTGCACTTACCATGCGT 8823

tobaco TGGACCTCAGTTGCTGGTTCTTATGGCTACATGGCACCAGAGCTTGCATTTACCATGCGC 9012

******:*** ***:***********.** ****************** **********

tomato GTTACAGAAAAGTGTGATGTCTATAGTTTTGGAGTCGTAGCGATGGAGACTATGATGGGA 9065

potato GTTACAGAAAAGTGTGATGTTTATAGTTTTGGAGTCGTAGCGATGGAGACTATGATGGGA 9072

pepino GTTACAGAAAAGTGTGATGTTTATAGTTTTGGAGTCATTGCGATGGAGACTATGATGGGA 9072

eggplant GTTACAGAAAAATGTGATGCTTACAGTTTTGGAGTCGTGGCGATGGAGGTTATGATGGGA 9042

Pepper GTTACAGAAAAGTGTGATGTTTATAGTTTTGGAGTGGTGGCGATGGAGACTATGATGGGA 8883

tobaco GTTACAGAAAAATGTGATGTTTATAGTTTTGGAGTTGTGGCTATGGAGATTATGATGGGA 9072

***********.******* ** *********** .* ** ******. **********

tomato AGGCATCCAGGGGAGCTTTTAACTTCATTATCAGCATCAACAACATTGTCTCCGGAAATA 9125

potato AGGCATCCAGGGGAGCTTTTAACTTCATTATCAGCATCAACAACATTGTTTCCAGAAATA 9132

pepino AGGCATCCAGGGGAGCTTTTAACTTCACTATCAGCATCAACAACATTGTCTTCGGAAATA 9132

eggplant AAGCATCCAGGGGAGCTTTTAACTTCATTATCAGCATCAACAACATTATCTTCAGAAATA 9102

Pepper AGACATCCAGGGGAGCTTTTAACTTCATTATCAGCAGCAACAACATTGTCTTCAGAAATA 8943

tobaco AGGCATCCAGGGGAGCTTTTAACCTCATTATCAGCAGCAACAACTTTATCATCGGAAATT 9132

*..******************** *** ******** *******:**.* : *.*****:

tomato CTTTTGAAGGATGTTCTTGACCAAAGACTTCCTCCTCCCACCGGCCACTTGGCAGAGGCA 9185

potato CTTTTGAAGGATGTTCTTGACCAAAGACTTCCACCTCCCACAGGCCACTTGGCAGAGGCA 9192

pepino CTTTTGAAGGATGTTCTTGACCAAAGACTTCCACCTCCCACCGGCCACTTGGCAGAGGCA 9192

eggplant CTTTTGAAGGATGTTCTTGACCAAAGGCTTTCACCTCCCACTGGTCACTTGGCAGAGGCA 9162

Pepper CTTTTGAAGGATGTTCTTGACCAAAGACTTGCACCTCCCTCAGGCCACTTGGCAGAGGAA 9003

tobaco CTTTTGAAGGATGTTCTTGACCAAAGACTTCAACCGCCCACTGGCCACTTGGCAGAAGCA 9192

**************************.*** .:** ***:* ** ***********.*.*

tomato GTGGTTTTTGTGATCACGATTGCCTTGGCATGTACACGTACCACGCCTGAATCGCGACCA 9245

potato GTGGTTTTTGTGATCACGATTGCCTTGGCATGTACACGTACCACTCCTGAATCGCGACCA 9252

pepino GTGGTTTTTGTAATCACAATTGCCTTGGCATGTACACGTACCACTCCTGAATCACGACCA 9252

eggplant GTAGTTTTTGTAATTACAATTGCCTTAGCATGTACACGTACCACTCCTGAGTCACGACCA 9222

Pepper GTGGTTTTTGTGATCACGATTGCATTGGCATGTACTCGTACCACTCCTGAGTCGCGACCA 9063

tobaco GTGGTTTTTGTTATCACGATTGCCTTTGCATGCACACGTACCACTCCTGAGTCACGACCA 9252

**.******** ** **.*****.** ***** **:******** *****.**.******

tomato ACCATGCGTTCTGTAGCACAAGAATTGTCTGTTCAGACTTTGCCTTACCTTCCACAGCCA 9305

potato ACCATGCGTTCTGTAGCACAAGAATTGTCTGTTCAGACTTTGCCTTACCTTCCACAGCCA 9312

pepino ACCATGCGTTCTGTAGCACAAGAATTGTCTGTTCAGACTTTGCCTTACCTTCCACAGCCA 9312

eggplant ACCATGCGTTCTGTAGCACAAGAATTGTCTGTTAAGACTTTGCCTTACCTTCCACAGCCA 9282

Pepper ACCATGCGTTTTGTAGCACAAGAATTATCTGCTAAGACTTTGCCTTACCTTCCACAGCCA 9123

tobaco ACCATGCGTTCTGTAGCACAAGAATTATCTGCTCAGACTTTGCCTTACCTTCCACAGCCA 9312

********** ***************.**** *.**************************

tomato CTGGGGACAATAGAAATGAGCAAACTAACAAGTTTTCAGAAATAATG 9352

potato TTGGGGACAATAGAAATGAGCAAACTAACAAGTTTTCAGAAATAA-- 9357

pepino TTGGGGACAATAGAAATGAGCAAACTAACAAGTTTTCAGAAATAATG 9359

eggplant TTGGGGACAATAGAAATGAGCAAACTAACAAGTTTTCAGAAATAA-- 9327

Pepper TTGGGGACAATAGAAATGAGCAAGCTAACAAGTTTCCAGAAATAG-- 9168

tobaco TTGGGGTCAATAGAAGTGAGCAAACTAACAAGTTTCCAGAAATAG-- 9357

*****:********.*******.*********** ********.
