# Supplementary material for: The first de novo transcriptome of pepino (Solanum muricatum): assembly, comprehensive analysis and comparison with the closely related species S. caripense, potato and tomato
Source: BMC Genomics. 2016 May 4;17:321. doi: 10.1186/s12864-016-2656-8 (PMC4855764; doi:10.1186/s12864-016-2656-8)
Supplement: Additional file 12: — Computational steps, software and their parameters used. A Word file with a description of the software used for the assembly process and annotation. (DOCX 16 kb) [file 12864_2016_2656_MOESM12_ESM.docx]

**Clean reads (using ngs_crumbs)**

1.- Quality trims:

trim_quality (default options)

2.- Filter by length

filter_by_length --paired_reads -n 70

3.- Filter illumina oligos

filter_by_blast_short --paired_reads -l GATCGGAAGAGCACACGTCT -l ACACTCTTTCCCTACACGACGCTCTTCCGATCT -l AATGATACGGCGACCACCGAGATCTACACTCTTTCCCTACACGACGCTCTTCCGATCT -l GTGACTGGAGTTCAGACGTGTGCTCTTCCGATCT -l ACACTCTTTCCCTACACGACGCTCTTCCGATCT -l GATCGGAAGAGCACACGTCTGAACTCCAGTCAC -l GTGACTGGAGTTCAGACGTGTGCTCTTCCGATCT -l CAAGCAGAAGACGGCATACGAGATCGTGATGTGACTGGAGTTC -l CAAGCAGAAGACGGCATACGAGATACATCGGTGACTGGAGTTC -l CAAGCAGAAGACGGCATACGAGATGCCTAAGTGACTGGAGTTC -l CAAGCAGAAGACGGCATACGAGATTGGTCAGTGACTGGAGTTC -l CAAGCAGAAGACGGCATACGAGATCACTGTGTGACTGGAGTTC -l CAAGCAGAAGACGGCATACGAGATATTGGCGTGACTGGAGTTC -l CAAGCAGAAGACGGCATACGAGATGATCTGGTGACTGGAGTTC -l CAAGCAGAAGACGGCATACGAGATTCAAGTGTGACTGGAGTTC -l CAAGCAGAAGACGGCATACGAGATCTGATCGTGACTGGAGTTC -l CAAGCAGAAGACGGCATACGAGATAAGCTAGTGACTGGAGTTC

4.- clean virus(muri)

filter_by_bowtie2 --paired_reads -i pep_virus_index

**Assembly**

1.- Trinity

Trinity.pl --seqType fq --JM 30G --left muri.1.fastq.gz --right muri.2.fastq.gz --output assembly --CPU 40 --full_cleanup

2.- Cap3

- cap3 Trinity.fasta -o 200 -p 99 > cap3.stdout
- python rename_cap3_contigs_to_trinity_subcomponents.py -ace Trinity.fasta.cap.ace -o Trinity.fasta.cap.contigs.renamed Trinity.fasta.cap.contigs
- cat Trinity.fasta.cap.contigs.renamed Trinity.fasta.cap.singlets > trinity_no_redundancy.fasta

3- Complexity filter (ngs_crumbs)

- filter_by_complexity trinity_no_redundancy.fasta -e trinity_no_redundancy_uncomplex.fasta -o trinity_no_redundancy_complex.fasta

4.- Split trinity subclusters (homemade script using blast)

- trinity_split_subcomp_by_transblast.py trinity_no_redundancy_complex.fasta -o trinity_no_redundancy_complex_clustered_by_trans_blast.fasta

5.-Filtering low expresed transcripts (using RSEM)

- /usr/local/biology/trinityrnaseq/util/RSEM_util/run_RSEM_align_n_estimate.pl  --transcripts trinity_no_redundancy_complex_clustered_by_trans_blast.fasta --seqType fq --left muri_1.fastq.gz --right muri_2.fastq.gz --output_dir rsem -- --num-threads 40 &> run_rsem.stdout
- /usr/local/biology/trinityrnaseq/util/filter_fasta_by_rsem_values.pl --rsem_output=rsem/RSEM.isoforms.results --fasta=trinity_no_redundancy_complex_clustered_by_trans_blast.fasta --isopct_cutoff=1.00 --output=trinity_no_redundancy_complex_clustered_by_trans_blast_low_express_filtered.fasta

**Annotation**

Using ngs_backbone:

We have created an ngs_backbone project using trinity assembly output as input of the project:

The used configuration file content:

[General_settings]

   tmpdir = '/home/santi/analises/muri/muri/tmp'

   project_name = 'muricatum'

   project_path = '/home/santi/analises/muri/muri'

   threads = 24

[Other_settings]

   default_sanger_quality = 20

   java_memory = 4096

   picard_path = '/usr/local/biology/picard-tools'

   gatk_path = '/usr/local/biology/gatk'

[Annotation]

[[go_annotation]]

       blast_database = 'nr'

       java_memory = 2048

       create_dat_file = False

       blast2go_path = '/usr/local/biology/b2g4pipe/blast2go.jar'

       b2g_properties_file = '/usr/local/biology/b2g4pipe/b2gPipe.properties'

[[ortholog_annotation]]

               ortholog_databases = ['tomato_pep', 'potato_pep']

[[description_annotation]]

       description_databases = ['swissprot', 'tomato_pep', 'arabidopsis_pep', 'uniref90']

[[Cdna_intron_annotation]]

                   genomic_db = '/srv/databases/blast/S_lycopersicum_chromosomes.2.50.fa'

       genomic_seq_file = '/srv/databases/blast/S_lycopersicum_chromosomes.2.50.fa'

[[orf_annotation]]

       estscan_matrix = '/usr/local/biology/estscan_matrix/Arabidopsis_thaliana.smat'

[blast]

[['nr']]

path    = '/srv/databases/blast/nr'

species = 'all'

[['swissprot']]

path    = '/srv/databases/blast/uniprot_sprot'

species = 'all'

[['uniref90']]

path    = '/srv/databases/blast/uniref90'

species = 'all'

[['arabidopsis_pep']]

path    = '/srv/databases/blast/tair10_pep'

species = 'arabidopsis'

kind    = 'prot'

[['tomato_pep']]

path    = '/srv/databases/blast/ITAG2.4_proteins.fasta'

species = 'tomato'

kind    = 'prot'

[['cucumber']]

path = '/srv/databases/blast/Cucumber_v2i.pep'

species = 'cucumber'

kind = 'prot'

[['calabaza_v1']]

path = '/srv/databases/blast/cucurbita_pepo_transcriptome_comav_v1'

species = 'calabaza'

kind = 'nucl'

[['potato_pep']]

path    = '/srv/databases/blast/Stuberosum_proteins.ITAG1.wfunct.fasta'

species = 'potato'

kind    = 'prot'
